# Activation of the ecdysone receptor agonism AOP (AOP4) in *Drosophila* S2 cells: a mechanistic *in vitro* model

**DOI:** 10.1016/j.namjnl.2026.100147

**Published:** 2026-10-13

**Authors:** Rebeka Đarmati, David Gómez-Blanco, Samantha Hughes, Paul L Carmichael, Bruno Campos, Nico W Van den Brink

**Affiliations:** a Wageningen University and Research, Droevendaalsesteeg 4, Wageningen, PB, 6708, Netherlands; b Unilever, Colworth House, Colworth Road, Bedford, MK44 1LQ, UK

**Keywords:** Adverse Outcome Pathway (AOP), *Drosophila melanogaster* cells, *In vitro* screening assay, Ecdysone Receptor agonism, New Approach Methodologies (NAMs)

## Abstract

A next generation environmental risk assessment encompasses a more mechanistic approach to understanding toxicity by employing New Approach Methodologies (NAMs). Despite the essential role invertebrates have in maintaining the ecosystem, invertebrate specific traits are often overlooked in development of NAMs. Here we investigate the potential of the *Drosophila melanogaster* S2 cell line to serve as an insect specific *in vitro* screening assay coupled to the ecdysone receptor agonism Adverse Outcome Pathway (AOP4). The ability of the S2 cells to portray the initial Key Events (KE) of the AOP4 was tested by studying expression profiles of transcription factors involved in early developmental regulation. The results show a dose and time dependent response to ecdysone exposure which is in line with gene expression profile seen *in vivo* and described in the AOP4. These findings lay the foundation for further development of the invertebrate specific cell-based assay that can be used for early screening of chemicals and offers a mechanistic method for hazard identification contributing to a more relevant and robust mechanistic based risk assessment.

## Highlights

• Drosophila S2 cells mimic the ecdysone cascade observed *in vivo*.
• S2 cells show key events of the ecdysone receptor agonism adverse outcome pathway.
• S2 cells are a promising model for insect specific *in vitro* assay development.

## Graphical abstract

**Figure:**
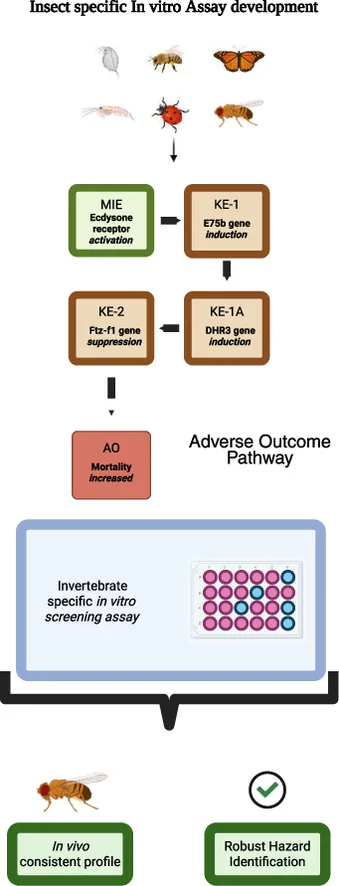

## Introduction

### Species specific environmental risk assessment

Invertebrates have an integral function in ecosystems, providing services like pollination, soil aeration, and carbon cycling. Yet, the increasing variety of chemicals entering the environment potentially threatens the biodiversity necessary for functional ecosystems (Wagner et al., 2021; Wan et al., 2025). Traditional Environmental Risk Assessment (ERA) relies on *in vivo* assays in a small set of species and focuses on apical endpoints rather than mechanisms of toxicity. Given the vast diversity of insect species, ERA protocols can be time-consuming, resource-intensive, and depend on interspecies extrapolation, emphasizing the need for early screening assays and New Approach Methodologies (NAMs) to streamline risk assessment and improve its mechanistic underpinning. NAMs incorporate *in vitro, in chemico*, and *in silico* approaches to provide more mechanistic and species-specific assessments. However, most NAM development focuses on humans and higher vertebrates, often neglecting invertebrate-specific traits. Wan et al. (2025) summarize results from over a thousand scientific papers, showing that pesticide risks to non-target invertebrates are often underestimated, in part due to limited mechanistic understanding of Adverse Outcome Pathways (AOPs) and Molecular Initiating Events (MIEs).​ 

To address this gap, we developed an *in vitro* assay targeting a critical invertebrate developmental pathway, the Ecdysone Receptor (EcR) signaling axis. The assay is anchored to the EcR agonism AOP (AOP4;Song, et al., 2017), providing a mechanistic framework for an invertebrate-specific screening system. S2 cell-based assays have already been proposed for detecting EcR agonists in pesticide development (Pinto et al., 2019), but they generally lack quantitative characterization of the underlying molecular pathways. EcR agonists are used as insect growth regulators (IGRs) that interfere with normal development by targeting endocrine pathways. Although IGRs are often considered highly specific, the high level of conservation of endocrine signaling across insect species suggests that non-target organisms may be more vulnerable to IGRs than assumed (Crane et al., 2022).​ 

### Molecular pathways underlying development in Drosophila melanogaster

Insects must molt to grow. Molting consists of detachment of the old exoskeleton, formation of the new exoskeleton, shedding of the old cuticle (ecdysis), and tanning. This process is initiated by ecdysone, a steroid hormone controlling insect development. The active form, 20-hydroxyecdysone (20E, hereafter “ecdysone”), is released by the prothoracic gland in pulses throughout the life cycle, triggering developmental transitions (Riddiford, 1993). *In vivo*, an ecdysone pulse activates the EcR-USP receptor dimer, inducing early transcription factors that belong to the nuclear receptor superfamily and control downstream genes required for completion of a developmental molt (Sullivan and Thummel, 2003). Within the ecdysis cascade, early and early-late genes E75b and Dhr3 are rapidly induced after an ecdysone pulse (Ashburner et al., 1972; Kageyama et al., 1997). E75b dimerizes with Dhr3, constraining Dhr3 activity and delaying activation of downstream targets (White et al., 1997). As ecdysone levels fall and E75b is downregulated, Dhr3 is released, inducing later genes such as Ftz-f1 and providing temporal control over ecdysis.

### Exogenous ecdysone receptor agonism

The adverse effects of exogenous EcR agonism on development and survival are captured in AOP4 ((Song, Daniel L. Villeneuve, et al., 2017); Fig. 1, aopwiki.org). In this AOP, the Molecular Initiating Event (MIE) is binding of an EcR agonist to the receptor, followed by induction of early genes (KE1 and KE1a). Under adverse conditions, such as continuous exposure to an EcR-active insecticide, the agonist remains bound, expression of later genes is suppressed (KE2), and the gene cascade is interrupted. This suppression prevents release of ecdysis-triggering hormone (KE3), blocking shedding of the old cuticle (KE4–KE8) while a new cuticle forms underneath, ultimately leading to incomplete ecdysis and mortality as the Adverse Outcome (AO).

**Fig. 1:**
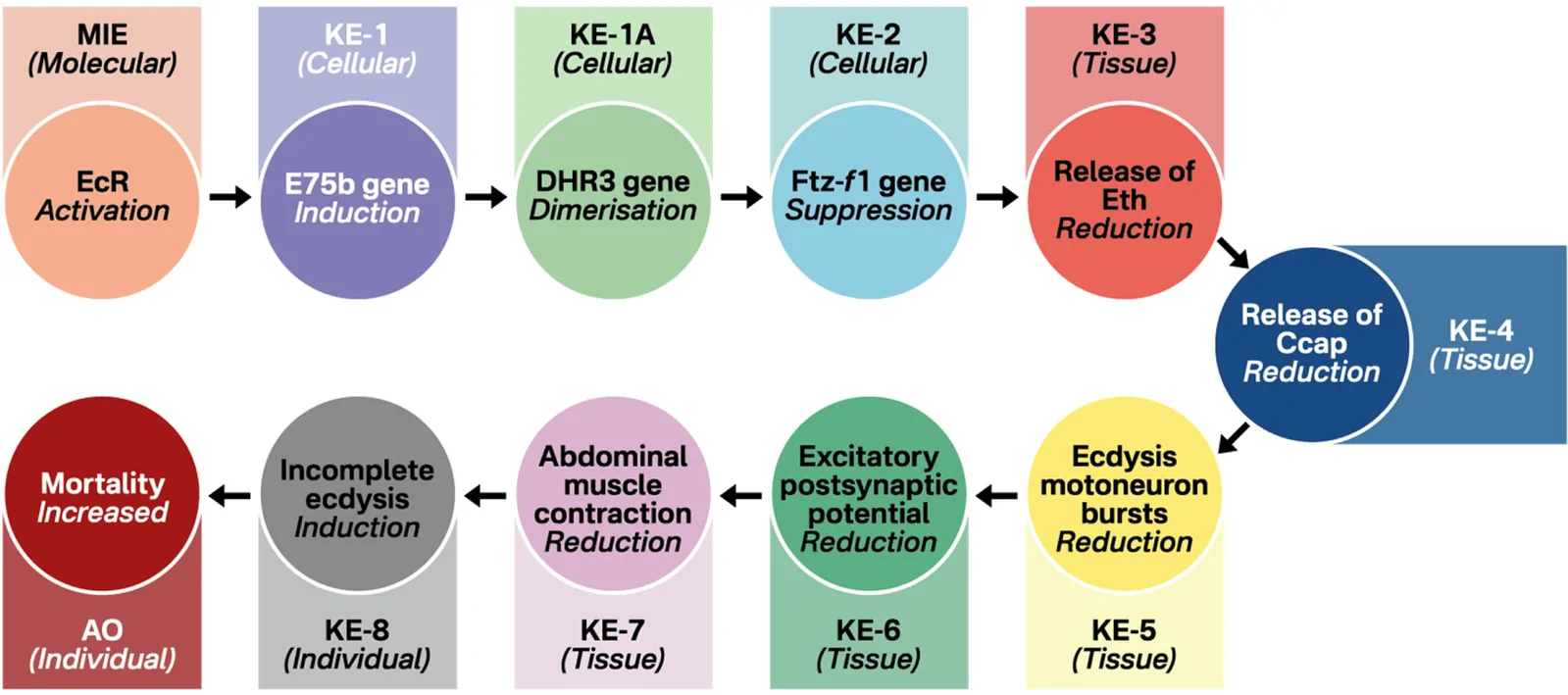
Ecdysone receptor agonism leading to incomplete ecdysis associated mortality Adverse Outcome Pathway (AOP4) adapted from Song et al. (2017). Abbreviations MIE: Molecular Initiating Event, KE: Key Event; AO: Adverse Outcome; EcR: Ecdysone Receptor; E75b: early gene triggered by EcR, a transcription factor; Dhr3: Drosophila Hormone Receptor 3, early-late gene, a transcription factor; Ftz-f1: fushi tarazu transcription factor 1, late gene; Eth: Ecdysis triggering hormone; Ccap: Crustacean cardioactive peptide, enables neuropeptide receptor binding activity.

In this study, we characterize the temporal and dose-response profiles of the ecdysis gene cascade in the Drosophila S2 cell line under physiologically relevant (pulsed) and adverse (continuous) exposure conditions. Our aim is to establish a proof-of-concept *in vitro* screening assay for EcR-disrupting chemicals that can be applied to additional compounds to support more robust evaluation of developmental toxicity. We also profile Brc, E75a, and EcR isoforms in S2 cells to explore the broader AOP network and identify additional key events relevant to EcR-mediated disruption.

Cell-based *in vitro* assays, once well characterized, offer reproducibility and scalability for high-throughput chemical screening at molecular level. In addition to AOP4, several AOPs with the same MIE (AOP342, AOP343, AOP606) are under development (aopwiki.org, October 2025), reflecting growing interest in EcR-mediated pathways. EcR isoforms (EcRA, EcRB1, EcRB2) and early genes Broad Complex (Brc) and E75a contribute to stage- and tissue-specific responses to ecdysone in *Drosophila melanogaster*, suggesting that additional EcR-related AOPs may be defined in parallel to AOP4 (William S. Talbot et al., 1993; Emery et al., 1994; Cakouros et al., 2004)

## Methods

### Cell culturing

Schneider’s Drosophila (S2) cells (ATCC; Manassas, VA, USA) were cultured in Schneider’s medium (Gibco, Fisher Scientific, Landsmeer, NL) supplemented with 10% heat-inactivated fetal bovine calf serum (FCS; Fisher Scientific, Landsmeer, NL) and 1% penicillin-streptomycin (Gibco, Fisher Scientific, Landsmeer, NL) at 25 °C. The same medium was used in all experiments. Cells were passaged twice a week. Cell viability and density were determined using a Cellometer K2 Fluorescent Cell Counter (Nexcelom Bioscience, Lawrence, MA, USA).

### Cytotoxicity assay

A cytotoxicity assay was performed to determine the sublethal concentrations of ecdysone for the 8 and 16 h exposure period. Cells were seeded in a white 96-well plate at a density of 40 000 cells in 100 µL of complete Schneider’s medium per well 24 h prior to exposure. Exposure medium was prepared in a 24-well plate using fresh Schneider’s medium and a range of concentration of ecdysone (Enzo Life Sciences, Lausen, Switzerland) previously prepared in Dimethyl Sulfoxide (DMSO; Merck, Darmstadt, Germany) (3 µM, 1 µM, 0.3 µM, 0.1 µM, 0.03 µM, 0.01 µM, 0.003 µM). A solvent control (0.5% DMSO), medium control and positive control (0.5% Triton X) were all included. Cell viability was assessed using the RealTime-Glo™ MT Cell Viability Assay (Promega, Leiden, the Netherlands), according to the producer’s manual, and measuring luminescence using the spectrophotometer (Tecan, Giessen, the Netherlands).

### Temporal and concentration-based gene expression profiling

The gene expression profile of S2 cells was investigated upon two ecdysone exposure scenarios (Pulsed and Continuous) in a time and concentration dependent manner (Fig. 2). The exposure medium was prepared in fresh Schneider’s medium by adding ecdysone dissolved in DMSO, at target final concentrations with a final DMSO concentration of 0.5%. The experiments included a solvent control of 0.5% DMSO in Schneider’s medium. For the time dependent gene expression, a target concentration of 0.1 µM of ecdysone was used and tested all time points (Fig. 2). For the concentration-based profiling, ecdysone concentrations used were: 3 µM, 1 µM, 0.3 µM, 0.1 µM, 0.03 µM, 0.01 µM, and 0.003 µM and all samples were assessed only after 8 h.

**Fig. 2:**
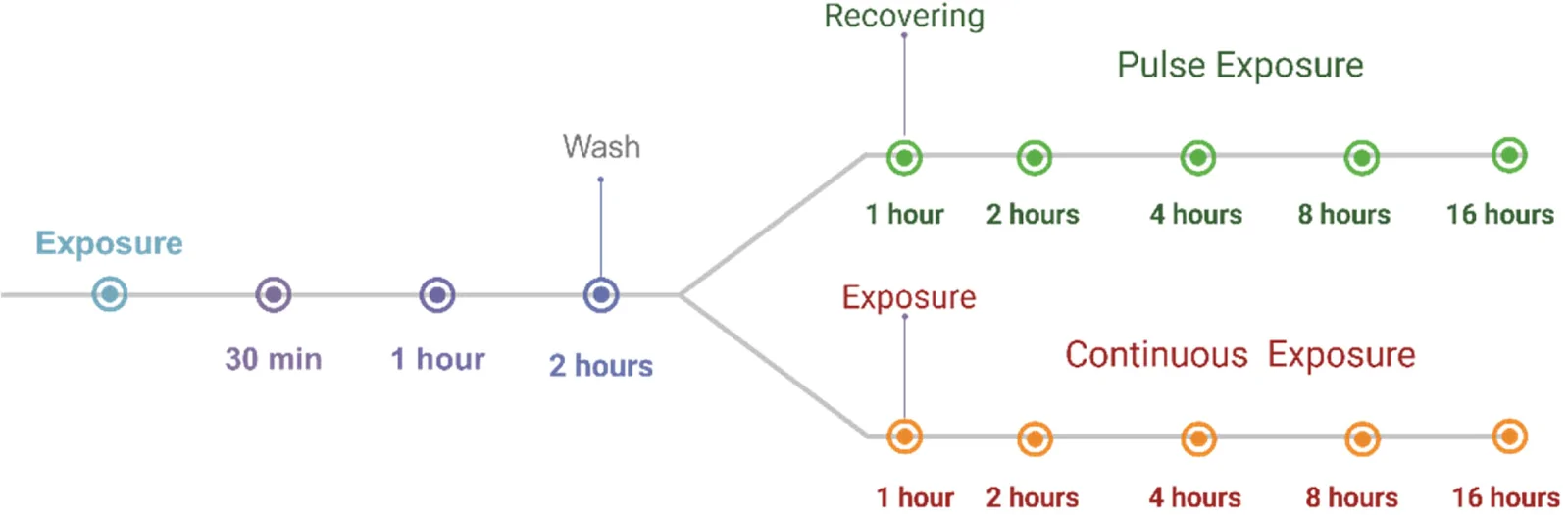
Conceptual representation of experiment set-up including a relevant (pulse exposure) and adverse (continuous exposure) scenario. The dots represent sampling moments after initial exposure, expressed as time after exposure. At the 2-hour point, a washing is also done prior to cells undergoing one of the 2 scenarios. The timeline is then split into two parts, pulse exposure scenario (top) and continuous scenario (bottom). In the pulse exposure scenario the dots represent the sampling moments expressed as recovery time or time since wash. In the continuous scenario the dots represent the sampling moments expressed as time after repeated exposure. Created in BioRender. https://BioRender.com/mbf7nls.

Cells were seeded in a 12-well plate at a density of 1 × 10^6^ cells per well in 1 mL of conditioned Schneider’s medium and incubated at 25 °C for 24 h prior to exposure. Cells were exposed to 1 mL per well of exposure medium, either ecdysone or solvent control. Then, the plates were incubated for a further 2 h. After incubation, cells were washed with phosphate-buffered saline (PBS; Gibco, Fisher Scientific, Landsmeer, NL) as follows. The entire content of each well was aspirated and transferred to a 2 mL Eppendorf tube. The tubes were centrifuged at 1000 rcf for 6 min. The supernatant was removed, and the cells were resuspended in 1 mL of PBS. The tubes were centrifuged again under the same conditions. The supernatant was removed, and the cells were resuspended in 1 mL of fresh Schneider’s medium. The wells were washed with 1 mL PBS prior to reseeding the cells. Cells were reseeded into the appropriate wells and exposed to 1 mL of either ecdysone or solvent control. After the incubation period, the cells were sampled by aspirating the entire contents of the well and transferring to a 2 mL Eppendorf tube. Then, 350 µL of RLT lysis buffer (Qiagen, Hilden, Germany) was added to each well. The tubes were centrifuged at 2000 rcf for 4 min, the supernatant removed, and the cells were resuspended in RLT lysis buffer aspirated from the corresponding well. The tubes were snap-frozen in liquid nitrogen and stored at −80 °C until further analysis.

### RNA isolation and RT-qPCR analysis

RNA isolation was performed using the RNeasy kit (Qiagen, Hilden, Germany) according to the manufacturer’s protocol. RNA quantity was measured using an Infinite® M Nano spectrophotometer (Tecan, Giessen, the Netherlands). The average RNA yield was 268 ng/µL with the yield range 89–589 ng/µL. The RNA quality was determined by using spectrophotometric measurements in a 260/280 ratio. A 260/280 ratio between 1.8 and 2.15 indicates low DNA contamination and good RNA quality. Reverse transcription was done using 1000 ng of RNA with iScript™ gDNA Clear cDNA Synthesis Kit (Bio-Rad Laboratories B.V., Veenendaal, the Netherlands) according to the manufacturer’s protocol and performed in 96 well plates using a BioRad PTC Tempo 96 Thermal cycler (Bio-Rad Laboratories B.V., Veenendaal, the Netherlands. Primers for quantitative PCR analysis were designed using NCBI Primer Blast software and ordered from Biolegio (Nijmegen, the Netherlands) (Table S1 in supplementary material). qPCR analysis was performed on a CFX Opus 384 Real-Time PCR System (Bio-Rad Laboratories B.V., Veenendaal, the Netherlands) in 10 µL reactions using 5 µL of iQ™ SYBR® Green Supermix (BioRad Laboratories B.V., Veenendaal, the Netherlands), 1 µL of specific primers, at a final concentration between 0.1 and 1 µM, 2 µL of DNase free water, and 2 µL of 64x diluted cDNA. For each primer pair, a calibration curve made of a 4x serial dilution of the sample pool including 5 concentrations in duplicate, was run on the same plate. The thermal cycling profile used is a two-step with melt curve, as follows: 95.0 °C for 3 min, 95.0 °C for 10 s, 60.0 °C for 30 s + Plate Read, repeated for 40 cycles, and Melt Curve 65.0 to 95.0 °C, increment 0.5 °C every 5 s + Plate Read. The transcription of the following target genes was analyzed: EcR (isoforms A, B1, B2), E75a, E75b, Brc, Dhr3, Ftz-f1. The expression level of target genes was corrected for the expression of a reference gene, Gapdh1. Quality control for qPCR included melting curves to determine the specificity of the primers and no-template control, calibration curves for primer efficiency (90–110%). Further data analysis was done by averaging the CT values of two replicates following the Livak method (Livak and Schmittgen, 2001).

### Statistical analysis

The cell viability data was analyzed in Excel. The raw luminescence values given by the spectrophotometer (Tecan, Giessen, the Netherlands) were used to determine the cell viability as a percentage of the solvent control. The data is presented as bar graphs created in GraphPad Prism 9 (GraphPad Software, San Diego, *CA*, USA).

For gene expression data, statistical analysis was undertaken in RStudio (Posit Software, PBC, Boston, MA, USA) using ddCT values instead of fold change (Yuan et al., 2006). Firstly, the normality of data was checked with a qq-plot. To determine if exposure scenarios had a significant effect on gene expression per time-point, five *t*-tests were performed for each target gene. We considered the unadjusted p-values with p < 0.05 indicating significance. The output values are summarized in table in Supplementary materials. To present gene expression data we plotted fold change values in bar graphs or dose-response curves made in GraphPad Prism 9 (GraphPad Software, San Diego, *CA*, USA).

## Results

### Cytotoxicity of ecdysone

First, cell viability after exposure to a range of concentrations of ecdysone for 8 and 16 h was assessed (Fig. 3). As viability never dropped below 50% compared to solvent control, this clearly shows the ecdysone concentrations used in this range were not toxic to the cells. Therefore, this allowed for the investigation of sublethal effects of prolonged ecdysone exposure Fig. 3.

**Fig. 3:**
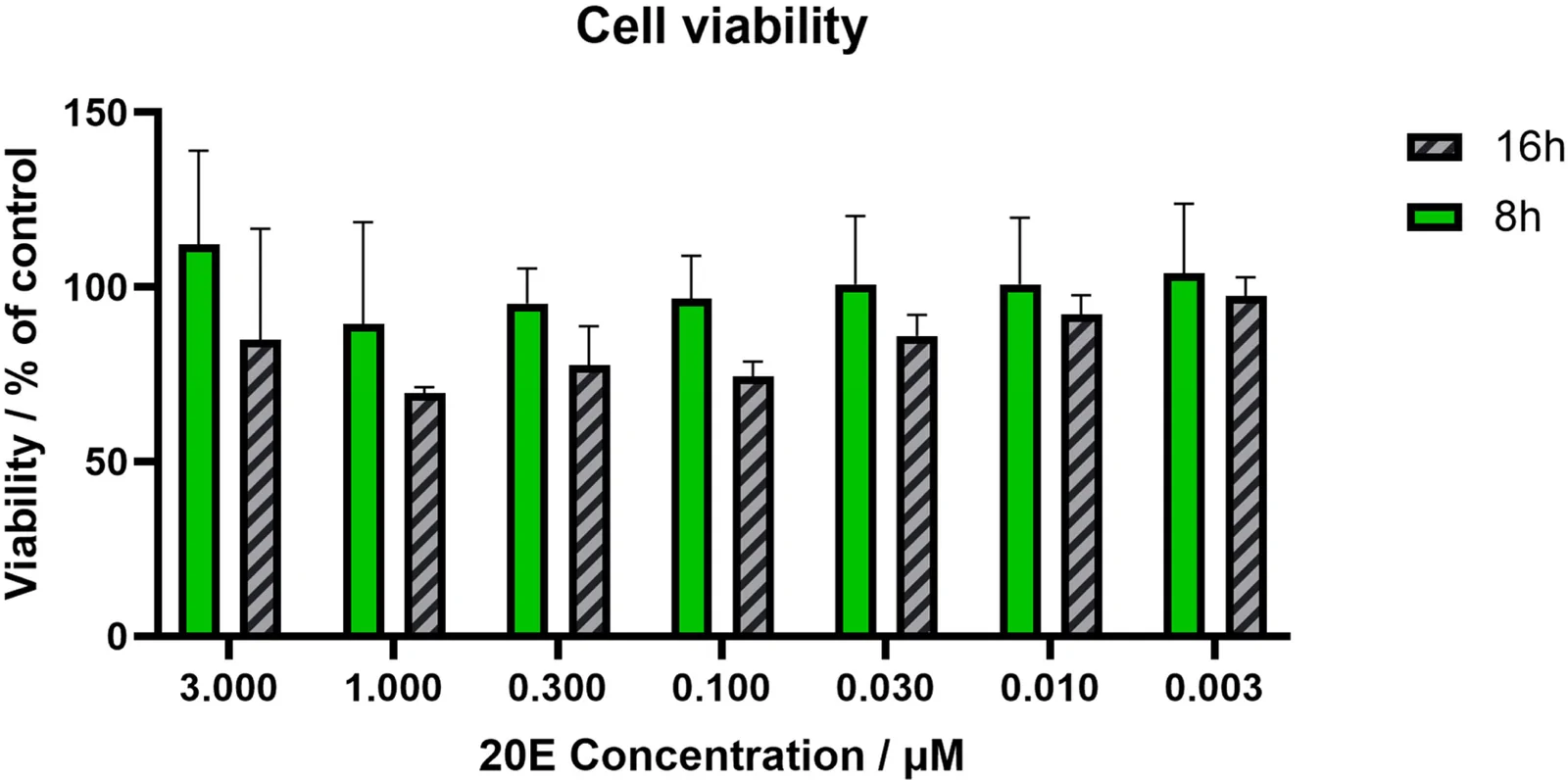
Cell viability of S2 cells after exposure to the following concentrations of ecdysone: 3 µM, 1 µM, 0.3 µM, 0.1 µM, 0.03 µM, 0.01 µM, and 0.003 µM. Viability assessed MT Cell Viability Assay. Viability is shown as average with error bars in percentage of the solvent control (0.5% DMSO. (N = 3).

### Gene expression profile in S2 cells

#### Validation of ecdysis gene expression cascade in the S2 cells under pulsed exposure

Ecdysone induced S2 cells express genes encoding the key transcription factors E75b, Dhr3 and Ftz-f1 form the backbone of the ecdysis cascade. The aim of this experiment was to mimic an ecdysone pulse as it occurs *in vivo*. Our findings show that expression of E75b is high during the 2 h of exposure, and then decreases after ecdysone clearance (Fig. 4A). The expression of Dhr3 steadily declined over 4 h post ecdysone clearance (Fig. 4B). The expression of Ftz-f1 remained low for the duration of the experiment (Fig. 4C). However, 8 h after the exposure, there is a slight induction of Ftz-f1, suggesting a delayed response to ecdysone exposure.

**Fig. 4:**
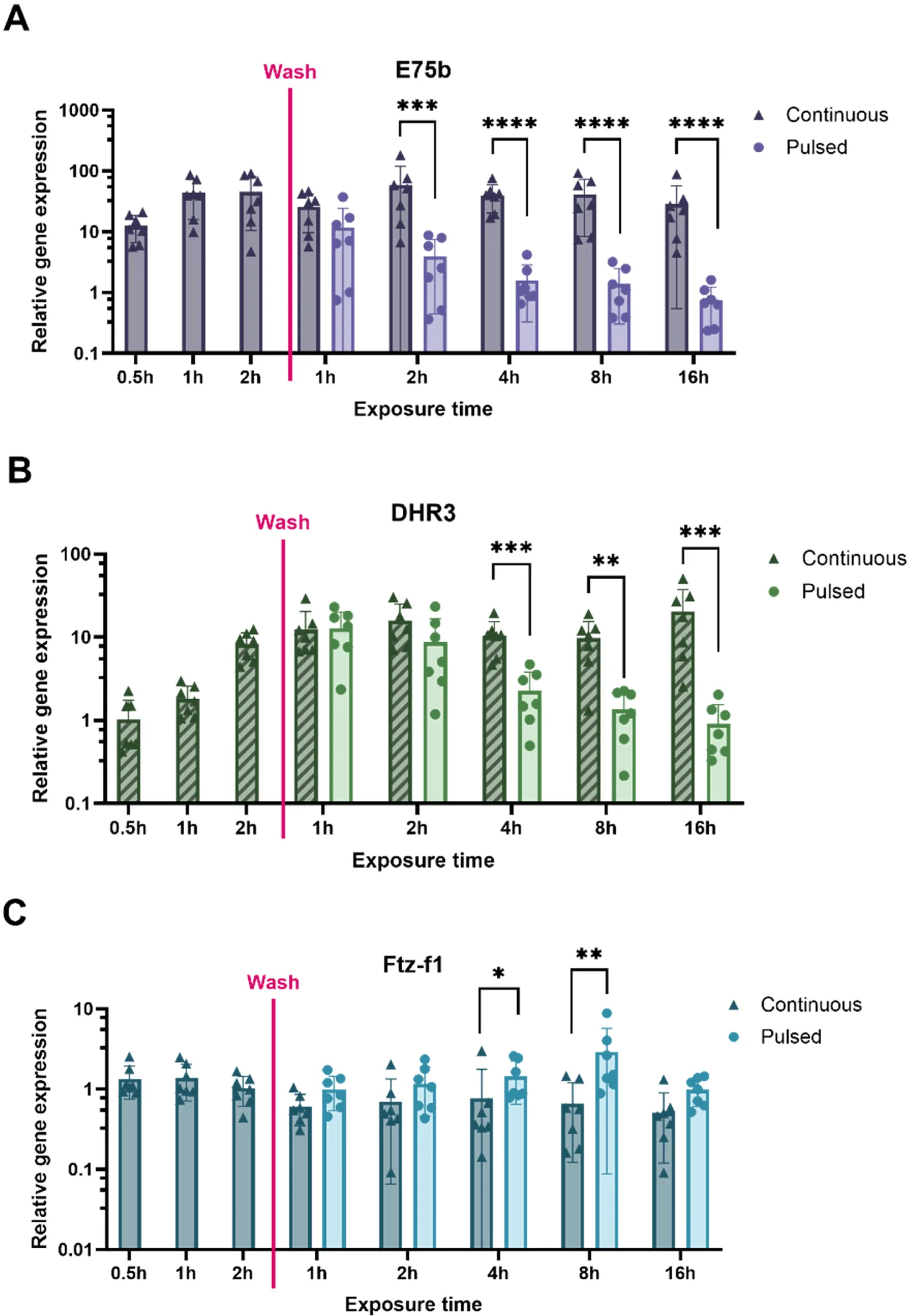
Graphs illustrating relative gene expression in S2 cells for the three key targets of the Ecdysis genetic cascade under two exposure scenarios, pulsed (dot) and continuous (triangle): A) E75b, B) Dhr3, C) Ftz-f1. The first three time points before the red “Wash” line (0.5 h, 1 h and 2 h) represent time after exposure to 0.1 µM of 20E. The time-points after the red “Wash” line represent time after washing with the two bars representing gene expression under two conditions: continuous exposure to 0.1 µM of 20E (continuous exposure), and the recovery condition, cells treated with growth medium only (pulsed exposure). Statistical significance of comparison between pulsed and continuous exposure per timepoint is represented with stars as per legend: * (p < 0.05), ** (p < 0.01), *** (p < 0.001), **** (p < 0.0001), N = 3.

#### Ecdysone receptor agonism AOP in S2 cells under continuous exposure

Upon continuous exposure scenario, S2 cells mimic the initial KEs of the EcR agonism AOP (AOP4). The gene expression for the continuous exposure is plotted next to the pulse scenario data in Fig. 4, which clearly shows significant differences between the pulsed and continuous exposure scenarios. The expression levels of E75b and Dhr3 remain relatively high in the continuous exposure scenario as compared to the pulse scenario. Although a significant induction of Ftz-f1 in the pulsed scenario is lacking, a significant downregulation was apparent in the continuous scenario.

#### Dose dependent gene expression in S2 cells

Fig. 5 shows dose-response curves per gene. Fig. 5A shows a dose-response relationship between ecdysone concentration and the expression of E75b in a continuous exposure scenario with an EC50 of 0.96 µM. The same can be seen for the expression of Dhr3 in Fig. 5B, with an EC50 of 0.9 µM. There was no expression of E75b and Dhr3 under the pulsed exposure scenario as the reading was 8 h after ecdysone was removed from the system leading to a background expression of the genes. The expression of Ftz-f1 varies slightly depending on the exposure concentration, however, the overall low expression levels do not yield a robust EC50 value (Fig. 5C).

**Fig. 5:**
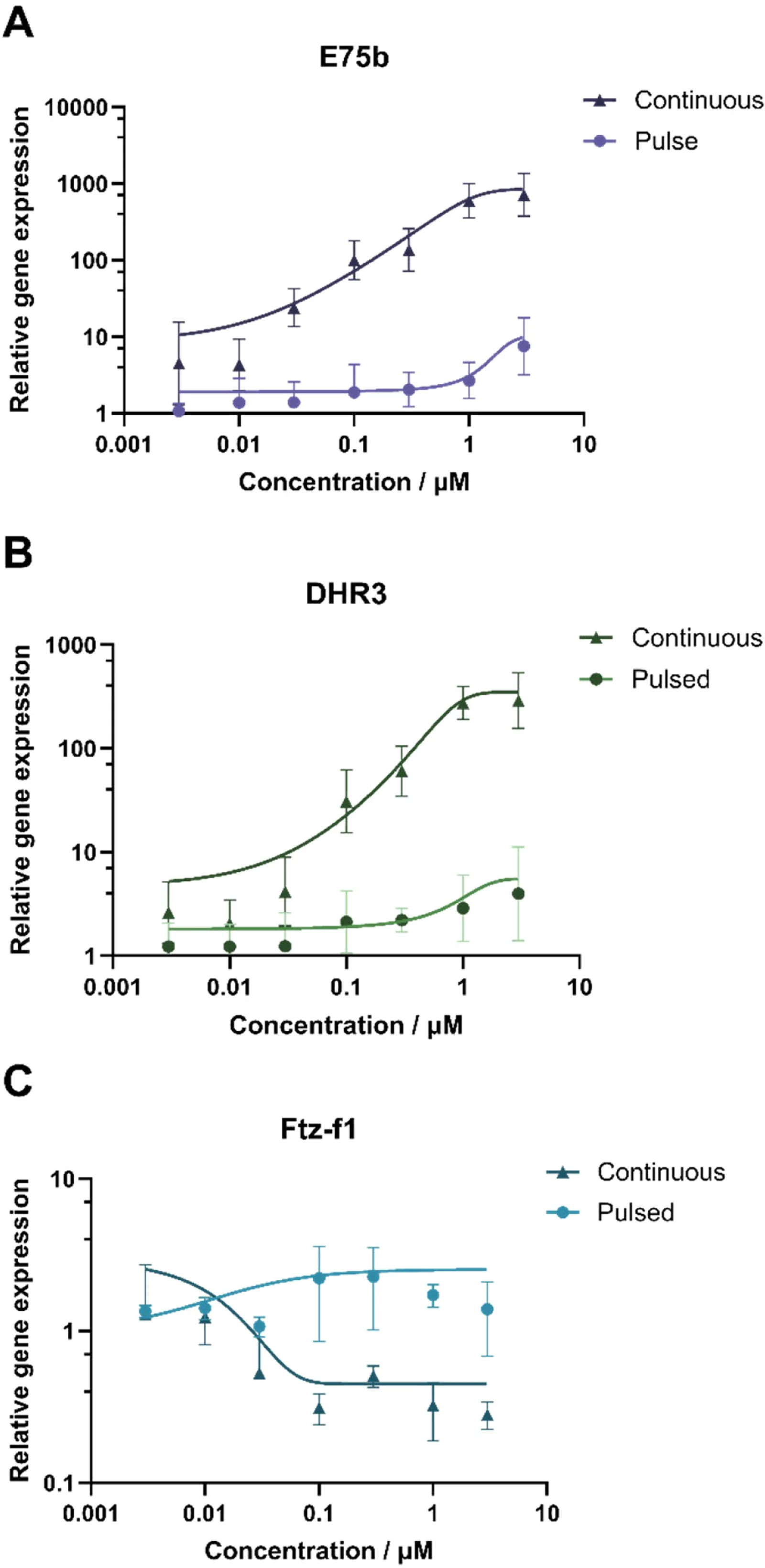
Graphs illustrating relative gene expression in S2 cells for the three key targets of the Ecdysis genetic cascade upon 8 hour exposure to a series of ecdysone concentrations under two exposure scenarios, pulsed (dot) and continuous (triangle): A) E75b, B) Dhr3, C) Ftz-f1. N = 3.

### Expression profiles of EcR isoforms and Brc and E75A in S2 cells

To further investigate the expression profile in ecdysone-induced S2 cells, a qPCR analysis of EcR isoforms and Brc and E75A using the same samples was done. Transcriptional analysis of the ecdysone receptors (Fig. 6) indicated EcRB1 playing the most important role in the S2 cells as it was induced within the first two hours of exposure, while the expression decreased following the removal of ecdysone (Fig. 6A). The continuous exposure profile exhibits an increased expression of EcRB1, suggesting the receptor remains induced for the duration of experiment (Fig. 6A). EcRA exhibits an interesting expression profile as it shows no activity until 18 h of exposure (2 h of initial exposure + 16 h after washing) (Fig. 6B). On the other hand, EcRB2 expression was below the limit of detection for all time-points (data not shown).

**Fig. 6:**
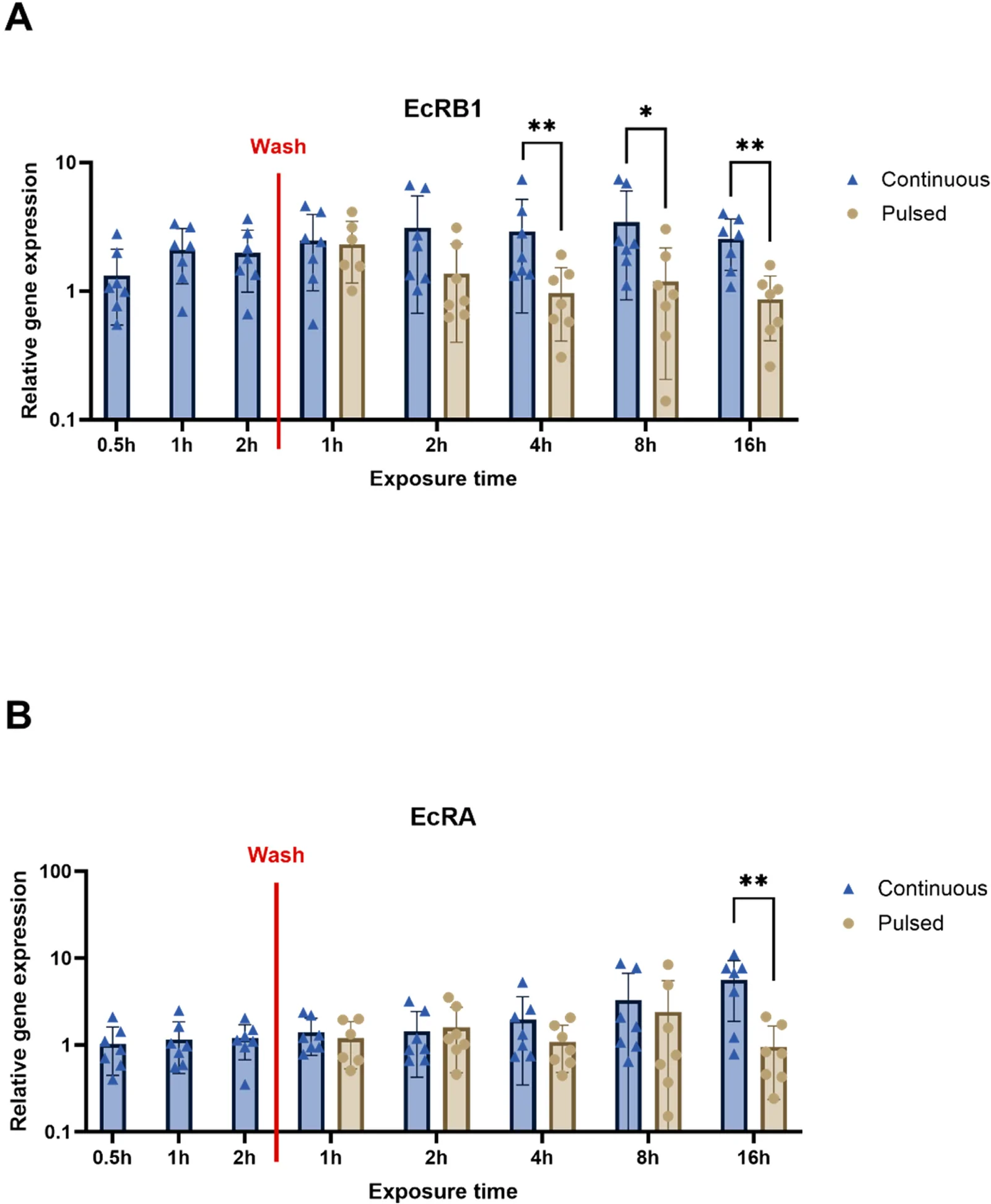
Graphs illustrating relative gene expression in S2 cells for EcR isoforms under two exposure scenarios, pulsed (blue, dot) and continuous (yellow, triangle): A) EcRA, B) EcRB1. The first three time points before the red “Wash” line (0.5 h, 1 h and 2 h) represent time after exposure to 0.1 µM of 20E. The time-points after the red “Wash” line represent time after washing with the two bars representing gene expression under two conditions: continuous exposure to 0.1 µM of 20E (continuous exposure), and the recovery condition, cells treated with growth medium only (pulsed exposure). Statistical significance of comparison between pulsed and continuous exposure per timepoint is represented with stars as per legend: * (p < 0.05), ** (p < 0.01), *** (p < 0.001). N = 3.

Furthermore, Broad Complex (Brc) and E75a are additional early genes of the ecdysis cascade and portray similar expression profiles (Fig. 7A and B respectively). Both genes are induced by ecdysone within the first hour of exposure and are downregulated within the first 2 h after ecdysone clearance. On the other hand, the continuous exposure scenario reveals both E75A and Brc remain induced while ecdysone is present. While these genes serve a different purpose in the process of development, the expression profiles are similar as seen for E75b and Dhr3 (as shown in Fig. 7) indicating the presence of alternative KEs in a parallel AOP.

**Fig. 7:**
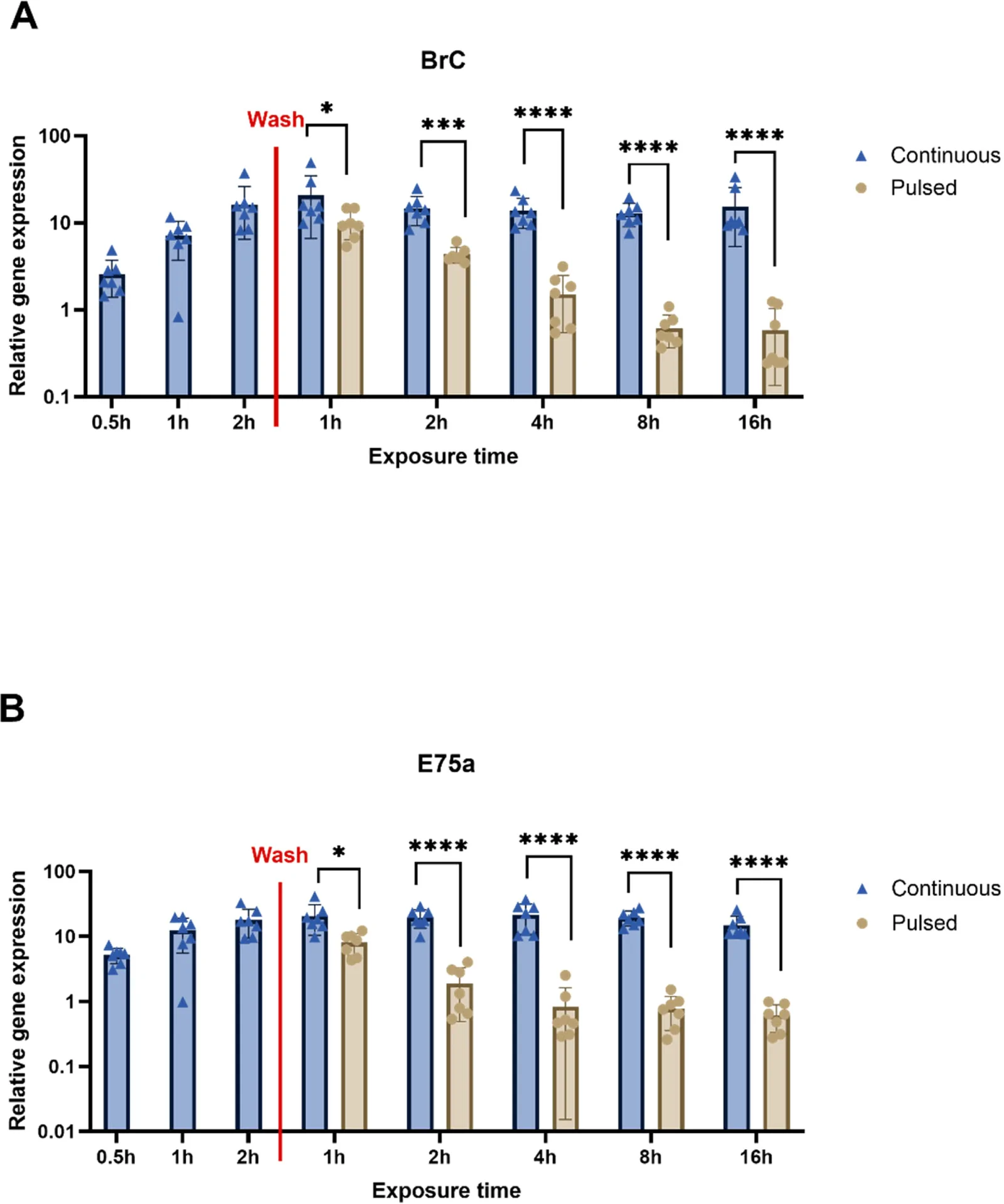
Graphs illustrating relative gene expression in S2 cells for additional targets of the Ecdysis genetic cascade under two exposure scenarios, relevant (blue, dot) and continuous (yellow, triangle): A) Brc, B) E75A. The first three time points before the red “Wash” line (0.5 h, 1 h and 2 h) represent time after exposure to 0.1 µM of 20E. The time-points after the red “Wash” line represent time after washing with the two bars representing gene expression under two conditions: continuous exposure to 0.1 µM of 20E (continuous exposure), and the recovery condition, cells treated with growth medium only (pulsed exposure). Statistical significance of comparison between pulsed and continuous exposure per timepoint is represented with stars as per legend : * (p < 0.05), ** (p < 0.01), *** (p < 0.001), **** (p < 0.0001), N = 3.

## Discussion

Strong societal pressure, the increasing number and variety of chemicals in the environment and the need for a more robust and protective risk assessment are leading the paradigm shift towards the mechanistic based, animal-free approaches in the Next Generation Risk Assessment of chemicals (NRC, 2007; Carmichael, 2022; Rivetti and Campos, 2023). Even though human health risk assessment has been at the center of NAM development, there is an increase in research dedicated to employing NAMs in environmental risk assessment (Basu et al., 2025). The initial efforts focused on reducing and replacing vertebrates testing which resulted in OECD test guideline (TG nr. 249) for the use of rainbow trout gill cell line in risk assessment of chemicals in aquatic environments and opened avenues for further development of the NAMs to replace fish testing (OECD, 2021; Emberley-Korkmaz et al., 2025). Although often underrepresented, the shortcomings of risk assessment for invertebrate specific traits are heavily discussed, emphasizing the need for faster and more protective tools (He et al., 2023; Goodsell et al., 2025). Developing cell-based assays anchored in AOPs offers substantial benefits for mechanistically robust safety assessments (Burden et al., 2015; LaLone et al., 2017). By integrating mechanistic information from AOPs, these assays allow mapping the causal chain from a molecular initiating event (MIE) through key events (KEs) to adverse outcomes (AO), improving biological relevance, cross-species extrapolation and predictive power of toxicity analyses (Burden et al., 2015; Paul Friedman et al., 2020). Furthermore, cell-based *in vitro* assays coupled to AOPs can serve together in a battery of tests for early screening of chemicals, thereby expediting the process of risk assessment while providing greater mechanistic accuracy. The same *in vitro* screening assays also support development of new chemicals under the EU framework “Safe and sustainable by design” (Abbate et al., 2024) contributing to reducing the chemical pressures on the environment.

This paper discusses the potential of the *Drosophila* S2 cell line coupled to AOP4 to serve as a screening assay. The applicability domain is in early screening of novel pesticides, and other chemicals for their ability to trigger AOP4 in non-target, economically important species (Song, et al., 2017). The aim of this study was to investigate the initial KEs of the EcR agonism AOP (AOP4) in the S2 cell line. Our results have suggested that S2 cells have the molecular capacity to follow the natural ecdysis cascade when exposed to a peak of ecdysone and portray the initial KEs of the AOP when continuously exposed. These events show a time and concentration-based relationship adding to the molecular underpinning needed to support the use of S2 cells as *in vitro* screening assays for toxicology.

### Ecdysis gene expression cascade and AOP4 in the S2 cells

The experiments included in this study have confirmed that ecdysone-induced S2 cells express the key factors of ecdysis gene cascade in a time and concentration dependent manner, as seen *in vivo* (Sullivan and Thummel, 2003). The high level of temporal regulation of gene expression *in vivo* contributes to a perfectly timed developmental transition, and this process is preserved in the S2 cell line. To mimic the ecdysone pulse in the presented experiments, cells were exposed to ecdysone for a short period of time. It was found that the early gene, E75b, portrays a fast response both to the presence and the absence of ecdysone, in line with literature (Bernardo et al., 2014; Sullivan and Thummel, 2003). Furthermore, the early-late Dhr3 gene is already expressed after two hours of exposure to ecdysone, however it only reaches peak expression, and becomes active, one hour after ecdysone clearance, corresponding to the downregulation of E75b. This dynamic has been studied *in vivo* as a key factor in temporal control of metamorphosis (Sullivan and Thummel, 2003). E75b inhibits Dhr3 by direct protein-protein interaction at the promotor region of Ftz-f1 (White et al., 1997). In addition, compared to E75b, Dhr3 exhibits a more gradual decrease in expression, returning to base levels only four hours after ecdysone clearance (Fig. 8) (Mazina et al., 2015). Our experimental setup did not allow for significant Ftz-f1 upregulation, despite the successful downregulation of E75b. This is possibly due to a variety of contributing factors that could be missing from the S2 cells or potentially the self-repressive nature of the Ftz-f1 that was shown *Drosophila* prepupal stage (Woodard et al., 1994; Borsos et al., 2015). *In vivo*, the Ftz-f1 transcripts necessary for embryonic development are maternally deposited (Ohno and Petkovich, 1993), which may be a reason for the lack of Ftz-f1 induction observed in these experiments, as S2 is an embryonic cell line. On the other hand, it is possible to induce Ftz-f1 gene expression in S2 cells by using charcoal treated media, which can aid in clearing hormones from the cell-based system (Vorobyeva et al., 2012; Sikora et al., 2016). Even though the cell media in this study were not treated with charcoal, ecdysone was successfully removed from the system as seen by the rapid downregulation of E75b and Dhr3. Nevertheless, it is important to consider that the removal of ecdysone may have been sufficient for downregulation of the early genes, but not enough to inhibit the repression of Ftz-f1. Furthermore, higher Ftz-f1 induction can be achieved by using S2 cells transfected with plasmids containing the Ftz-f1 promotor and/or coding regions which can enhance expression compared to the not transfected cells used in this research (Dubrovsky et al., 2011; Bernardo and Dubrovsky, 2012). Despite these shortcomings, this experiential set-up was successfully able to mimic the ecdysis cascade until Ftz-f1 expression.

**Fig. 8:**
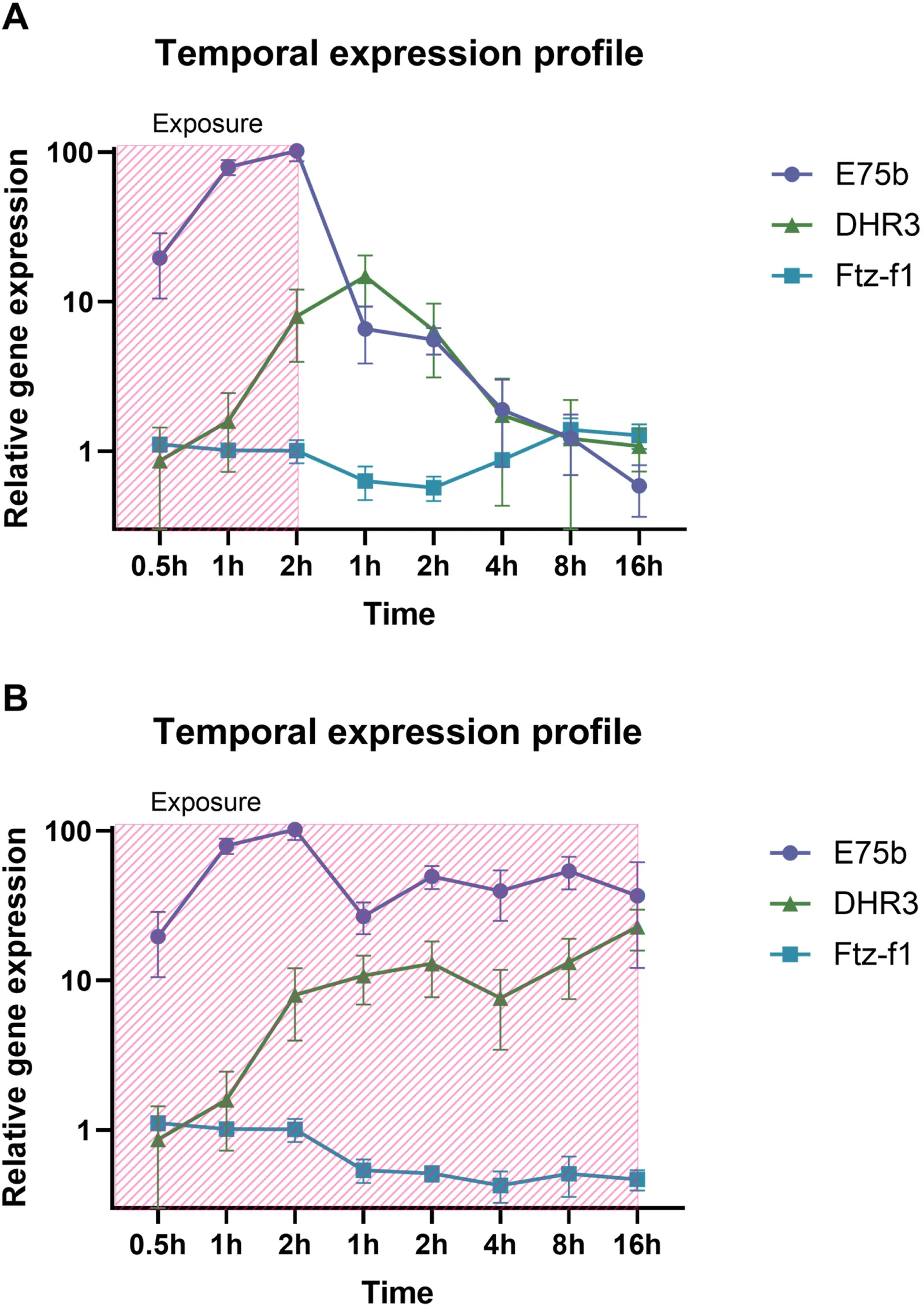
Temporal expression profile of E75b, Dhr3 and Ftz-f1 for pulsed (A) and continuous (B) exposure scenario. Striped (pink) area marks the exposure period. The relative genes expression is shown on a log10 scale on the x axis. The time is shown in hours, from left to right, after the first 2 h the cells are washed and (A) allowed to recover (1–16 h) in the pulsed scenario, or (B) exposed again (1–16 h).

To explore the applicability of S2 cells as model system for early KEs of AOP4, cells were again exposed to ecdysone to create a continuous exposure profile. The results show that both E75b and Dhr3 remain induced for the duration of the experiment (Fig. 8B). When compared to the pulsed exposure profile, there are significant differences in expression across time points, and a clear trend of expression upregulation of both E75b and Dhr3 as designated in KE1 and KE1A. The continuous exposure scenario successfully triggers the KEs as described by Song et al. (2017). Moreover, in the AOP4, KE2 is characterized by Ftz-f1 suppression (Song et al., 2017). Although the initial pulsed-exposure setup did not significantly increase Ftz-f1 expression, the continuous exposure scenario resulted in significant Ftz-f1 downregulation, aligning with the predicted directionality for KE2. *In vivo*, silencing or suppression of βFtz-f1 leads to severe molting defects, including arrested development, double mouthparts, and failure of ecdysis, supporting the biological plausibility of this key event sequence (Cho et al., 2014).

Furthermore, the induction of E75b and Dhr3 exhibits a dose dependent relationship upon continuous exposure to ecdysone. This suggests the potential for quantifying Key Event Relationships and thus strengthens the applicability of the proposed AOP framework (Ankley et al., 2010; Song et al., 2017). On the other hand, in the pulsed scenario, the same genes remain downregulated 8 h after clearance, independent of the dose of ecdysone. This implies that ecdysone can still dissociate from the receptor even after exposure to high concentrations.

### Expression profile of EcR isoforms, Brc and E75a

EcR activation is identified as the MIE of the EcR agonism AOP and has been studied previously employing reporter gene assays paired to Ecdysone Response Elements (Dela Cruz et al., 2000; Zotti et al., 2013; Pinto et al., 2019). In this paper the gene expression profile of three EcR isoforms under the two exposure conditions was analyzed to investigate the dynamics of EcR regulation in S2 cells. The results show only EcRB1 is expressed immediately after ecdysone exposure, with expression slowly reduced to a fluctuating low level when ecdysone is removed from the system. In the continuous exposure scenario, EcRB1 remains expressed for the duration of exposure, while EcRA appears to be induced only 16 h after repeated exposure. The predominant expression of EcRB1 in ecdysone induced S2 cells enables the strong transcriptional response to ecdysone, confirmed by others (Cruz et al., 2000; Ruff et al., 2009; Kovalenko et al., 2019). Furthermore, Gradilla et al. (2011) found that ubiquitylation of EcRA is needed for protein regulation which may in turn activate a feedback mechanism to induce gene transcription, potentially explaining the late EcRA induction seen in our results. In addition, F. Dela Cruz et al. (2000) found the isoforms bind ecdysone with similar affinities, which opens further questions on the regulation of EcR isoform expression. *In vivo*, the EcR isoforms contribute to a tissue specific response to ecdysone that enables development. For example, EcRA is generally responsible for differentiation of adult tissue during metamorphosis, while EcRB1 and EcRB2 play a role in larval tissue regression (neuronal tissue) but also proliferation in muscle tissue (Davis et al., 2005; Talbot et al., 1993; Truman and Riddiford, 2023). Therefore, EcR has the ability to induce a myriad of genetic pathways depending on the cell type and developmental stage.

In the efforts to understand the molecular events occurring in S2 cells exposed to ecdysone under the two exposure scenarios, the analysis was expanded to two additional targets: E75a and Brc. The results showed that both E75a and Brc remain upregulated throughout the exposure period and are back to background levels upon ecdysone clearance. As both genes play an important role in the ecdysone response acting as transcription factors this opens up possibilities for developing new AOPs with the same MIE and thereby broadening the applicability of S2 cells in early screening of chemicals.

## Conclusion

The aim of this study was to investigate the ability of the S2 cell line to portray the early KEs of the AOP4. The results indicate that the ecdysis genetic cascade is preserved in the S2 cells and if continuously exposed to ecdysone, the potential adverse scenario, the KEs as described in the AOP, are maintained to a certain degree. In addition, this research sheds light on the expression patterns of EcR isoforms and two additional targets (E75a and Brc) important for the aforementioned cascade in the S2 cells. Exploring the molecular network regulating insect development emphasizes the importance of the proposed EcR agonism AOP while suggesting that complementary AOPs with the same MIE may further advance our understanding. Although limited to the initial KEs, S2 cells present opportunities for the further development of this *in vitro* screening tool. Demonstrating the initial KEs of the EcR agonism AOP in the S2 cells opens a new line of investigation targeting the relative potency of an array of EcR agonists. This next step is important for building the body of evidence that can eventually lead to adoption of the model system in early toxicity assessment supporting the development of safe chemicals.

## Funding

This work was supported by Unilever Safety, Environmental and Regulatory Sciences.

## CRediT authorship contribution statement

**Rebeka Đarmati:** Writing – original draft, Methodology, Investigation, Formal analysis, Data curation, Conceptualization. **David Gómez-Blanco:** Writing – review & editing, Methodology, Data curation. **Samantha Hughes:** Writing – review & editing, Conceptualization. **Paul L Carmichael:** Writing – review & editing. **Bruno Campos:** Writing – review & editing, Methodology, Funding acquisition, Conceptualization. **Nico W Van den Brink:** Writing – review & editing, Methodology, Funding acquisition, Conceptualization.

## Declaration of competing interest

The authors declare that they have no known competing financial interests or personal relationships that could have appeared to influence the work reported in this paper.

## Data Availability

Data will be made available on request.

## References

[bib0001] Abbate E. (2024). Safe and Sustainable by Design Chemicals and Materials-Methodological Guidance.

[bib0002] Ankley G.T. (2009). Adverse outcome pathways: a conceptual framework to support ecotoxicology research and risk assessment. Env. Toxicol. Chem..

[bib0003] Ashburner M., Chihara C., Meltzer P., Richards G. (1972). Temporal Control Puffing Act. Polytene Chromosomes.

[bib0004] Basu N., Knapen D., LaLone C., Villeneuve D. (2025). Progress on new approach methods (NAMs) in ecotoxicology. Env. Toxicol. Chem.

[bib0006] Bernardo T.J., Dubrovsky E.B. (2012). The Drosophila juvenile hormone receptor candidates methoprene-tolerant (MET) and germ cell-expressed (GCE) utilize a conserved LIXXL motif to bind the FTZ-F1 nuclear receptor. J. Biol. Chem..

[bib0005] Bernardo T.J., Dubrovskaya V.A., Xie X., Dubrovsky E.B. (2014). A view through a chromatin loop: insights into the ecdysone activation of early genes in Drosophila. Nucleic Acids Res..

[bib0007] Borsos B.N. (2015). Acetylations of ftz-F1 and histone H4K5 are required for the fine-tuning of ecdysone biosynthesis during Drosophila metamorphosis. Dev. Biol..

[bib0008] Burden N. (2015). Adverse outcome pathways can drive non-animal approaches for safety assessment. J. Appl. Toxicol..

[bib0009] Cakouros D., Daish T.J., Kumar S. (2004). Ecdysone receptor directly binds the promoter of the *Drosophila* caspase *dronc*, regulating its expression in specific tissues. J. Cell Biol..

[bib0010] Carmichael P. (2022). Ready for regulatory use: nAMs and NGRA for chemical safety assurance. ALTEX.

[bib0011] Cho K-H (2014). Secretory competence in a gateway endocrine cell conferred by the nuclear receptor βftz-F1 enables stage-specific ecdysone responses throughout development in Drosophila. Dev. Biol.

[bib0012] Crane M. (2022). Commentary: assessing the endocrine disrupting effects of chemicals on invertebrates in the European Union. Env. Sci. Eur..

[bib0014] Davis M.B., Carney G.E., Robertson A.E., Bender M. (2005). Phenotypic analysis of EcR-A mutants suggests that EcR isoforms have unique functions during Drosophila development. Dev. Biol.

[bib0013] Dela Cruz F., Kirsch D., Heinrich J. (2000). Transcriptional activity of Drosophila melanogaster ecdysone receptor isoforms and ultraspiracle in Saccharomyces cerevisiae. J. Mol. Endocrinol.

[bib0015] Dubrovsky E.B. (2011). The drosophila FTZ-F1 nuclear receptor mediates juvenile hormone activation of E75A gene expression through an intracellular pathway. J. Biol. Chem..

[bib0016] Emberley-Korkmaz S. (2025). Cytotoxicity of 19 pesticides in rainbow trout gill, liver, and intestinal cell lines. Env. Toxicol. Chem.

[bib0017] Emery I.F., Bedian V., Guild G.M. (1994). Differential expression of Broad-Complex transcription factors may forecast tissue-specific developmental fates during Drosophila metamorphosis. Development.

[bib0018] Goodsell R.M. (2025). Moving towards better risk assessment for invertebrate conservation. Ecography.

[bib0046] Gradilla Ana-Citlali, Mansilla Alicia, Ferrús Alberto (2011). Isoform-specific regulation of a steroid hormone nuclear receptor by an E3 ubiquitin ligase in Drosophila melanogaster. Genetics.

[bib0019] He X. (2023). Insect cell-based models: cell line establishment and application. Insectic. Screen. Toxicol. Res. Insects..

[bib0020] Kageyama Y., Masuda S., Hirose S., Ueda H. (1997). Temporal regulation of the mid-prepupal gene FTZ-F1: dhr3 early late gene product is one of the plural positive regulators. Genes Cells.

[bib0021] Kovalenko E.V., Mazina M.Y., Krasnov A.N., Vorobyeva N.E. (2019). The Drosophila nuclear receptors EcR and ERR jointly regulate the expression of genes involved in carbohydrate metabolism. Insect. Biochem. Mol. Biol..

[bib0022] LaLone C.A. (2017). Advancing the adverse outcome pathway framework—An international horizon scanning approach. Env. Toxicol. Chem..

[bib0045] Livak Kenneth J., Schmittgen Thomas D. (2001). Analysis of relative gene expression data using real-time quantitative PCR and the 2−δδct method. Methods.

[bib0023] Mazina M.Y. (2015). Early-late genes of the ecdysone cascade as models for transcriptional studies. Cell Cycle.

[bib0024] NRC. 2007. Toxicity testing in the 21st century: a vision and a strategy. 10.17226/11970.

[bib0025] OECD (2021).

[bib0026] Ohno C.K., Petkovich M. (1993). FTZ-F1β, a novel member of the Drosophila nuclear receptor family. Mech. Dev.

[bib0027] Paul Friedman K. (2020). Utility of *In vitro* bioactivity as a lower bound estimate of *In vivo* adverse effect levels and in risk-based prioritization. Toxicol. Sci..

[bib0028] Pinto C.P.G., Rickes L.N., Zotti M.J., Grutzmacher A.D. (2019). Compared activity of agonist molecules towards ecdysone receptor in insect cell-based screening system. Arq. Inst. Biol. (Sao Paulo).

[bib0029] Riddiford L.M. (1993). The Development of Drosophila melanogaster.

[bib0030] Rivetti C., Campos B. (2023). Establishing a NexGen, mechanism-based environmental risk assessment paradigm shift: are we ready yet?. Integr Env. Assess Manag..

[bib0031] Ruff H., Tremmel C., Spindler-Barth M. (2009). Transcriptional activity of ecdysone receptor isoforms is regulated by modulation of receptor stability and interaction with Ab- and C-domains of the heterodimerization partner ultraspiracle. Arch. Insect. Biochem. Physiol.

[bib0032] Sikora M.J., Johnson M.D., Lee A V, Oesterreich S. (2016). Endocrine response phenotypes are altered by charcoal-stripped serum variability. Endocrinology.

[bib0033] Song Y., Villeneuve Daniel L. (2017). Ecdysone receptor agonism leading to lethal molting disruption in arthropods: review and adverse outcome pathway development. Env. Sci. Technol..

[bib0035] Sullivan A.A., Thummel C.S. (2003). Temporal profiles of nuclear receptor gene expression reveal coordinate transcriptional responses during drosophila development. Mol. Endocrinol..

[bib0036] Talbot William S., Swyryd E.A., Hogness D.S. (1993). Drosophila tissues with different metamorphic responses to ecdysone express different ecdysone receptor isoforms. Cell.

[bib0037] Truman J.W., Riddiford L.M. (2023). Drosophila postembryonic nervous system development: a model for the endocrine control of development. Genetics.

[bib0038] Vorobyeva N.E. (2012). SAYP and Brahma are important for “repressive” and “transient” Pol II pausing. Nucleic. Acids Res..

[bib0039] Wagner D.L. (2021). Insect decline in the Anthropocene: death by a thousand cuts. Proc. Natl. Acad. Sci..

[bib0040] Wan N-F (2025). Pesticides have negative effects on non-target organisms. Nat. Commun.

[bib0041] White K.P., Hurban P., Watanabe T., Hogness D.S. (1997). Coordination of Drosophila metamorphosis by two ecdysone-induced nuclear receptors. Sci. (1979).

[bib0042] Woodard C.T., Baehrecke E.H., Thummel C.S. (1994). A molecular mechanism for the stage specificity of the Drosophila prepupal genetic response to ecdysone. Cell.

[bib0043] Yuan J.S., Reed A., Chen F., Stewart C.N. (2006). Statistical analysis of real-time PCR data. BMC Bioinform..

[bib0044] Zotti M.J. (2013). A cell-based reporter assay for screening for EcR agonist/antagonist activity of natural ecdysteroids in Lepidoptera (Bm5) and Diptera (S2) cell cultures, followed by modeling of ecdysteroid-EcR interactions and normal mode analysis. Pestic. Biochem. Physiol.

