# Supplementary material for: Activation of the ecdysone receptor agonism AOP (AOP4) in Drosophila S2 cells: a mechanistic in vitro model
Source: NAM J. 2026 Oct 13;2:100147. doi: 10.1016/j.namjnl.2026.100147 (PMC13721100; doi:10.1016/j.namjnl.2026.100147)
Supplement: Supplementary file 1 [file mmc1.docx]

# Supplementary material

### S1: Primer sequences used for RT-qPCR analysis

Table S1 Primer sequences used for RT-qPCR analysis

| E75A | forward | CACCACCAGCTCCAGAACAG |
| --- | --- | --- |
|  | reverse | GGCGGTAGTTTCAGGGCATT |
| E75B | forward | CAACAGCGATGAGGATTGCC |
|  | reverse | ACGCGCATAGCGTATCTTCT |
| Drosophila Hormone Receptor 3 | forward | TCAAAGCAACGTCCAATCGC |
|  | reverse | TAATGCACGCCGGATGACTT |
| Fushi tarazu f1 | forward | TTGCCACGAGGCTGGAATAG |
|  | reverse | TGTGCCAGAATCGAAGCACAA |
| Ecdysone Receptor isoform A | forward | CCGCGACTTGATGTTACCGA |
|  | reverse | TAGCTGTGATTCTGCGAGCC |
| Ecdysone Receptor isoform B1 | forward | CGCGCAGACAACCATCATTC |
|  | reverse | GCCAACGCTCCCAGATTAGT |
| Ecdysone Receptor isoform B2 | forward | GCAGAACTGGCGCACTATATC |
|  | reverse | TGCTCTTCTTCGCATCGCA |
| Broad complex | forward | GTGCAATCCGTGCAACAAGA |
|  | reverse | ATATTGCACACGGGCTCCTT |
| Gapdh1 | forward | TGGATCTTACCGTCCGCTTG |
|  | reverse | GCGTCGAACACAGACGAATG |

### S2: Statistics per gene for the two exposure conditions over time

| **Target gene: E75b** |  |  |  |  |  |  |
| --- | --- | --- | --- | --- | --- | --- |
| Time point | t-value | df | p-value | 95% CI lower | 95% CI upper | Bonferroni-adjusted p-value |
| 1 h | 1.93 | 9.49 | 0.0834 | -0.281 | 3.79 | 0.417 |
| 2 h | 4.35 | 11.8 | 0.000999 | 2.00 | 6.05 | 0.00499 |
| 4 h | 10.6 | 11.4 | 0.000000300 | 3.80 | 5.78 | 0.00000150 |
| 8 h | 6.70 | 11.6 | 0.0000259 | 3.21 | 6.32 | 0.0000129 |
| 16 h | 7.56 | 11.0 | 0.0000114 | 3.53 | 6.43 | 0.0000570 |

| **Target gene: Dhr3** |  |  |  |  |  |  |
| --- | --- | --- | --- | --- | --- | --- |
| Time point | t-value | df | p-value | 95% CI lower | 95% CI upper | Bonferroni-adjusted p-value |
| 1 h | -3.44 | 10.6 | 0.0807 | -1.09 | 1.18 | 1 |
| 2 h | -3.79 | 9.45 | 0.0838 | -0.200 | 2.67 | 0.419 |
| 4 h | -3.26 | 9.96 | 0.000743 | 1.29 | 3.54 | 0.00371 |
| 8 h | -2.95 | 12.0 | 0.00112 | 1.39 | 4.31 | 0.00560 |
| 16 h | -3.79 | 9.67 | 0.000118 | 2.63 | 5.61 | 0.000591 |

| **Target gene: Ftz-f1** |  |  |  |  |  |  |
| --- | --- | --- | --- | --- | --- | --- |
| Time point | t-value | df | p-value | 95% CI lower | 95% CI upper | Bonferroni-adjusted p-value |
| 1 h | 0.834 | 11.7 | 0.0662 | -1.44 | 0.0542 | 0.331 |
| 2 h | -1.60 | 10.5 | 0.139 | -2.34 | 0.375 | 0.693 |
| 4 h | -2.39 | 9.44 | 0.0395 | -2.71 | -0.0827 | 0.198 |
| 8 h | -3.27 | 11.9 | 0.00670 | -3.57 | -0.715 | 0.0335 |
| 16 h | -2.43 | 8.80 | 0.0387 | -2.35 | -0.0791 | 0.193 |

| **Target gene: EcRA** |  |  |  |  |  |  |
| --- | --- | --- | --- | --- | --- | --- |
| Time point | t-value | df | p-value | 95% CI lower | 95% CI upper | Bonferroni-adjusted p-value |
| 1 h | -0.403 | 9.38 | 0.727 | -0.668 | 1.31 | 1 |
| 2 h | -0.336 | 11.9 | 0.822 | -1.39 | 1.13 | 1 |
| 4 h | -0.596 | 11.2 | 0.172 | -0.351 | 1.74 | 0.859 |
| 8 h | -0.861 | 10.4 | 0.276 | -0.866 | 2.73 | 1 |
| 16 h | -1.86 | 10.6 | 0.00213 | 1.11 | 3.81 | 0.0107 |

| **Target gene: EcRB1** |  |  |  |  |  |  |
| --- | --- | --- | --- | --- | --- | --- |
| Time point | t-value | df | p-value | 95% CI lower | 95% CI upper | Bonferroni-adjusted p-value |
| 1 h | -1.03 | 10.7 | 0.954 | -1.14 | 1.08 | 1 |
| 2 h | -1.26 | 11.6 | 0.0712 | -0.109 | 2.26 | 0.356 |
| 4 h | -1.24 | 12.0 | 0.00990 | 0.437 | 2.59 | 0.0495 |
| 8 h | -1.46 | 10.8 | 0.0260 | 0.249 | 3.19 | 0.130 |
| 16 h | -1.21 | 11.4 | 0.00257 | 0.701 | 2.56 | 0.0129 |

| **Target gene: Brc** |  |  |  |  |  |  |
| --- | --- | --- | --- | --- | --- | --- |
| Time point | t-value | df | p-value | 95% CI lower | 95% CI upper | Bonferroni-adjusted p-value |
| 1 h | 2.28 | 10.2 | 0.0451 | 0.0241 | 1.78 | 0.225 |
| 2 h | 7.05 | 8.18 | 0.00000812 | 1.15 | 2.26 | 0.000481 |
| 4 h | 8.28 | 10.1 | 0.000000802 | 2.48 | 4.31 | 0.0000400 |
| 8 h | 17.2 | 11.9 | 0.0000000000842 | 3.86 | 4.98 | 0.00000000421 |
| 16 h | 10.4 | 11.0 | 0.00000481 | 3.84 | 5.89 | 0.00000240 |

| **Target gene: E75a** |  |  |  |  |  |  |
| --- | --- | --- | --- | --- | --- | --- |
| Time point | t-value | df | p-value | 95% CI lower | 95% CI upper | Bonferroni-adjusted p-value |
| 1 h | 4.01 | 11.9 | 0.00178 | 0.600 | 2.03 | 0.00888 |
| 2 h | 7.59 | 8.08 | 0.0000604 | 2.57 | 4.82 | 0.000302 |
| 4 h | 9.63 | 10.6 | 0.00000140 | 3.83 | 6.11 | 0.00000699 |
| 8 h | 13.5 | 8.08 | 0.000000804 | 3.99 | 5.64 | 0.00000402 |
| 16 h | 14.4 | 10.5 | 0.000000295 | 3.97 | 5.41 | 0.00000148 |

| sample id | replicate | experiment number | Treatment | exposure time/hours | recovery time/hours | Avg Cq | st.dev Cq | corrected Cq (max cq 35) | Refrence gene Cq | delta Cq | Avg. solvent control delta Cq | st.dev solvent control delta Cq | delta delta Cq | 2^ddCq |
| --- | --- | --- | --- | --- | --- | --- | --- | --- | --- | --- | --- | --- | --- | --- |
| PR1hA0.1R1 | 1 | 1 | 0.1 µM 20E | 1 | NA | 26,13 | 0,02089 | 26,13 | 21,28 | 4,85 | 11,16 | 0,965829 | -4,39 | 20,91 |
| PR1hA0.1R2 | 2 | 1 | 0.1 µM 20E | 1 | NA | 28,26 | 0,00129 | 28,26 | 23,08 | 5,19 | 11,16 | 0,965829 | -4,23 | 18,79 |
| PR1hA0.1R3 | 3 | 1 | 0.1 µM 20E | 1 | NA | 36,54 | 1,24381 | 35,00 | 24,59 | 10,41 | 11,16 | 0,965829 | -3,46 | 10,99 |
| PR1hB0.1R1 | 1 | 1 | 0.1 µM 20E | 1 | NA | 29,57 | 0,14238 | 29,57 | 24,31 | 5,26 | 11,16 | 0,965829 |  |  |
| PR1hB0.1R2 | 2 | 1 | 0.1 µM 20E | 1 | NA | 28,76 | 0,38295 | 28,76 | 23,36 | 5,40 | 11,16 | 0,965829 |  |  |
| PR1hB0.1R3 | 3 | 1 | 0.1 µM 20E | 1 | NA | 36,57 | 0,16753 | 35,00 | 26,08 | 8,92 | 11,16 | 0,965829 |  |  |
| PR1hCSR1 | 1 | 1 | 0.1 µM 20E | 1 | NA | 37,17 | 0,00000 | 35,00 | 23,96 | 11,04 | 11,16 | 0,965829 |  |  |
| PR1hCSR2 | 2 | 1 | 0.1 µM 20E | 1 | NA | 35,34 | 1,63820 | 35,34 | 24,23 | 11,12 | 11,16 | 0,965829 |  |  |
| PR1hCSR3 | 3 | 1 | 0.1 µM 20E | 1 | NA | 33,22 | 1,84902 | 33,22 | 22,68 | 10,54 | 11,16 | 0,965829 |  |  |
| PR2hA0.1R1 | 1 | 1 | 0.1 µM 20E | 2 | NA | 26,03 | 0,09343 | 26,03 | 21,39 | 4,64 | 11,16 | 0,965829 | -6,10 | 68,75 |
| PR2hA0.1R2 | 2 | 1 | 0.1 µM 20E | 2 | NA | 28,49 | 0,03464 | 28,49 | 24,00 | 4,49 | 11,16 | 0,965829 | -5,86 | 58,20 |
| PR2hA0.1R3 | 3 | 1 | 0.1 µM 20E | 2 | NA | 29,40 | 0,30653 | 29,40 | 24,36 | 5,04 | 11,16 | 0,965829 | -1,49 | 2,81 |
| PR2hB0.1R1 | 1 | 1 | 0.1 µM 20E | 2 | NA | 28,77 | 0,34167 | 28,77 | 23,91 | 4,86 | 11,16 | 0,965829 |  |  |
| PR2hB0.1R2 | 2 | 1 | 0.1 µM 20E | 2 | NA | 28,28 | 0,31111 | 28,28 | 23,48 | 4,80 | 11,16 | 0,965829 |  |  |
| PR2hB0.1R3 | 3 | 1 | 0.1 µM 20E | 2 | NA | 30,36 | 0,67995 | 30,36 | 25,26 | 5,10 | 11,16 | 0,965829 |  |  |
| PR2hCSR1 | 1 | 1 | 0.5% DMSO | 2 | NA | 34,87 | 1,04142 | 34,87 | 23,16 | 11,72 | 11,16 | 0,965829 |  |  |
| PR2hCSR2 | 2 | 1 | 0.5% DMSO | 2 | NA | 36,54 | 0,00000 | 35,00 | 24,31 | 10,69 | 11,16 | 0,965829 |  |  |
| PR2hCSR3 | 3 | 1 | 0.5% DMSO | 2 | NA | 33,75 | 0,04970 | 33,75 | 22,03 | 11,72 | 11,16 | 0,965829 |  |  |
| PR30minA0.1R1 | 1 | 1 | 0.1 µM 20E | 0,5 | NA | 26,71 | 0,26915 | 26,72 | 20,05 | 6,67 | 11,16 | 0,965829 | -6,41 | 84,99 |
| PR30minA0.1R2 | 2 | 1 | 0.1 µM 20E | 0,5 | NA | 30,46 | 0,05462 | 30,46 | 24,01 | 6,45 | 11,16 | 0,965829 | -6,51 | 91,07 |
| PR30minA0.1R3 | 3 | 1 | 0.1 µM 20E | 0,5 | NA | 36,15 | 0,00000 | 35,00 | 24,45 | 10,55 | 11,16 | 0,965829 | -6,09 | 68,04 |
| PR30minB0.1R1 | 1 | 1 | 0.1 µM 20E | 0,5 | NA | 30,70 | 0,50500 | 30,70 | 23,83 | 6,87 | 11,16 | 0,965829 |  |  |
| PR30minB0.1R2 | 2 | 1 | 0.1 µM 20E | 0,5 | NA | 30,88 | 0,16317 | 30,89 | 23,49 | 7,40 | 11,16 | 0,965829 |  |  |
| PR30minB0.1R3 | 3 | 1 | 0.1 µM 20E | 0,5 | NA | 35,39 | 0,00000 | 35,39 | 25,28 | 10,11 | 11,16 | 0,965829 |  |  |
| PR30minCSR1 | 1 | 1 | 0.5% DMSO | 0,5 | NA | 34,36 | 0,00000 | 34,36 | 23,56 | 10,80 | 11,16 | 0,965829 |  |  |
| PR30minCSR2 | 2 | 1 | 0.5% DMSO | 0,5 | NA | 35,25 | 0,99541 | 35,25 | 24,18 | 11,07 | 11,16 | 0,965829 |  |  |
| PR30minCSR3 | 3 | 1 | 0.5% DMSO | 0,5 | NA | 33,96 | 1,27104 | 33,96 | 23,03 | 10,93 | 11,16 | 0,965829 |  |  |
| R16hCSR1 | 1 | 1 | 0.5% DMSO | 18 | NA | 34,34 | 0,00000 | 34,34 | 23,69 | 10,65 | 11,16 | 0,965829 |  |  |
| R16hCSR2 | 2 | 1 | 0.5% DMSO | 18 | NA | 35,34 | 0,00000 | 35,34 | 24,22 | 11,13 | 11,16 | 0,965829 |  |  |
| R16hCSR3 | 3 | 1 | 0.5% DMSO | 18 | NA | 32,14 | 0,09990 | 32,14 | 21,52 | 10,62 | 11,16 | 0,965829 |  |  |
| R1hCSR1 | 1 | 1 | 0.5% DMSO | 3 | NA | 33,33 | 1,23050 | 33,34 | 22,56 | 10,78 | 11,16 | 0,965829 |  |  |
| R1hCSR2 | 2 | 1 | 0.5% DMSO | 3 | NA | 35,51 | 0,00000 | 35,51 | 24,66 | 10,85 | 11,16 | 0,965829 |  |  |
| R1hCSR3 | 3 | 1 | 0.5% DMSO | 3 | NA | 33,35 | 0,45068 | 33,35 | 23,21 | 10,14 | 11,16 | 0,965829 |  |  |
| R2hCSR1 | 1 | 1 | 0.5% DMSO | 4 | NA | 33,85 | 0,24336 | 33,85 | 22,76 | 11,09 | 11,16 | 0,965829 |  |  |
| R2hCSR2 | 2 | 1 | 0.5% DMSO | 4 | NA | 35,98 | 0,66578 | 35,00 | 23,95 | 11,05 | 11,16 | 0,965829 |  |  |
| R2hCSR3 | 3 | 1 | 0.5% DMSO | 4 | NA | 33,08 | 1,03187 | 33,08 | 22,52 | 10,57 | 11,16 | 0,965829 |  |  |
| R4hCSR1 | 1 | 1 | 0.5% DMSO | 6 | NA | 32,96 | 0,13681 | 32,96 | 23,01 | 9,95 | 11,16 | 0,965829 |  |  |
| R4hCSR2 | 2 | 1 | 0.5% DMSO | 6 | NA | 36,70 | 0,17962 | 35,00 | 24,21 | 10,79 | 11,16 | 0,965829 |  |  |
| R4hCSR3 | 3 | 1 | 0.5% DMSO | 6 | NA | 30,93 | 0,80456 | 30,94 | 22,52 | 8,42 | 11,16 | 0,965829 |  |  |
| R8hCSR1 | 1 | 1 | 0.5% DMSO | 10 | NA | 34,46 | 0,81036 | 34,46 | 22,68 | 11,78 | 11,16 | 0,965829 |  |  |
| R8hCSR2 | 2 | 1 | 0.5% DMSO | 10 | NA | 35,35 | 0,00000 | 35,35 | 23,87 | 11,48 | 11,16 | 0,965829 |  |  |
| R8hCSR3 | 3 | 1 | 0.5% DMSO | 10 | NA | 35,12 | 2,98357 | 35,12 | 22,68 | 12,44 | 11,16 | 0,965829 |  |  |
| RC16h0.1R1 | 1 | 1 | 0.1 µM 20E | 18 | NA | 29,08 | 0,14463 | 29,08 | 24,39 | 4,69 | 11,16 | 0,965829 | -6,46 | 88,22 |
| RC16h0.1R2 | 2 | 1 | 0.1 µM 20E | 18 | NA | 30,76 | 0,39342 | 30,76 | 24,72 | 6,04 | 11,16 | 0,965829 | -5,11 | 34,64 |
| RC16h0.1R3 | 3 | 1 | 0.1 µM 20E | 18 | NA | 28,04 | 0,03846 | 28,04 | 21,62 | 6,42 | 11,16 | 0,965829 | -4,73 | 26,60 |
| RC1h0.1R1 | 1 | 1 | 0.1 µM 20E | 3 | NA | 29,25 | 0,09986 | 29,25 | 23,63 | 5,63 | 11,16 | 0,965829 | -5,53 | 46,12 |
| RC1h0.1R2 | 2 | 1 | 0.1 µM 20E | 3 | NA | 30,06 | 0,33561 | 30,06 | 23,92 | 6,14 | 11,16 | 0,965829 | -5,01 | 32,25 |
| RC1h0.1R3 | 3 | 1 | 0.1 µM 20E | 3 | NA | 31,64 | 0,66578 | 31,64 | 25,41 | 6,23 | 11,16 | 0,965829 | -4,92 | 30,32 |
| RC2h0.1R1 | 1 | 1 | 0.1 µM 20E | 4 | NA | 28,92 | 0,60873 | 28,92 | 23,64 | 5,28 | 11,16 | 0,965829 | -5,88 | 58,72 |
| RC2h0.1R2 | 2 | 1 | 0.1 µM 20E | 4 | NA | 29,12 | 0,06975 | 29,13 | 24,21 | 4,92 | 11,16 | 0,965829 | -6,24 | 75,44 |
| RC2h0.1R3 | 3 | 1 | 0.1 µM 20E | 4 | NA | 28,84 | 0,65579 | 28,84 | 25,19 | 3,65 | 11,16 | 0,965829 | -7,51 | 181,96 |
| RC4h0.1R1 | 1 | 1 | 0.1 µM 20E | 6 | NA | 28,53 | 0,64253 | 28,53 | 23,62 | 4,91 | 11,16 | 0,965829 | -6,24 | 75,83 |
| RC4h0.1R2 | 2 | 1 | 0.1 µM 20E | 6 | NA | 29,73 | 0,00367 | 29,73 | 24,12 | 5,60 | 11,16 | 0,965829 | -5,55 | 46,90 |
| RC4h0.1R3 | 3 | 1 | 0.1 µM 20E | 6 | NA | 30,99 | 0,65219 | 30,99 | 25,08 | 5,91 | 11,16 | 0,965829 | -5,25 | 37,98 |
| RC8h0.1R1 | 1 | 1 | 0.1 µM 20E | 10 | NA | 29,12 | 0,49696 | 29,12 | 24,04 | 5,08 | 11,16 | 0,965829 | -6,08 | 67,51 |
| RC8h0.1R2 | 2 | 1 | 0.1 µM 20E | 10 | NA | 27,61 | 0,01646 | 27,61 | 22,99 | 4,62 | 11,16 | 0,965829 | -6,53 | 92,55 |
| RC8h0.1R3 | 3 | 1 | 0.1 µM 20E | 10 | NA | 31,02 | 0,91758 | 31,02 | 25,73 | 5,29 | 11,16 | 0,965829 | -5,86 | 58,16 |
| RR16h0.1R1 | 1 | 1 | 0.1 µM 20E | 2 | 16 | 34,70 | 0,28067 | 34,71 | 23,69 | 11,02 | 11,16 | 0,965829 | -0,14 | 1,10 |
| RR16h0.1R2 | 2 | 1 | 0.1 µM 20E | 2 | 16 | 36,68 | 1,40401 | 35,00 | 24,53 | 10,47 | 11,16 | 0,965829 | -0,69 | 1,61 |
| RR16h0.1R3 | 3 | 1 | 0.1 µM 20E | 2 | 16 | 33,38 | 0,15175 | 33,39 | 21,85 | 11,53 | 11,16 | 0,965829 | 0,38 | 0,77 |
| RR1h0.1R1 | 1 | 1 | 0.1 µM 20E | 2 | 1 | 29,93 | 0,14108 | 29,93 | 21,60 | 8,33 | 11,16 | 0,965829 | -2,83 | 7,10 |
| RR1h0.1R2 | 2 | 1 | 0.1 µM 20E | 2 | 1 | 31,39 | 0,02609 | 31,40 | 23,95 | 7,45 | 11,16 | 0,965829 | -3,71 | 13,08 |
| RR1h0.1R3 | 3 | 1 | 0.1 µM 20E | 2 | 1 | 33,06 | 0,00561 | 33,06 | 24,60 | 8,47 | 11,16 | 0,965829 | -2,69 | 6,45 |
| RR2h0.1R1 | 1 | 1 | 0.1 µM 20E | 2 | 2 | 29,19 | 0,22069 | 29,19 | 20,57 | 8,62 | 11,16 | 0,965829 | -2,54 | 5,81 |
| RR2h0.1R2 | 2 | 1 | 0.1 µM 20E | 2 | 2 | 32,43 | 0,06168 | 32,43 | 24,27 | 8,16 | 11,16 | 0,965829 | -2,99 | 7,97 |
| RR2h0.1R3 | 3 | 1 | 0.1 µM 20E | 2 | 2 | 32,55 | 0,25455 | 32,56 | 24,53 | 8,03 | 11,16 | 0,965829 | -3,13 | 8,75 |
| RR4h0.1R1 | 1 | 1 | 0.1 µM 20E | 2 | 4 | 30,83 | 0,52069 | 30,84 | 21,74 | 9,10 | 11,16 | 0,965829 | -2,06 | 4,17 |
| RR4h0.1R2 | 2 | 1 | 0.1 µM 20E | 2 | 4 | 34,34 | 0,54199 | 34,34 | 23,44 | 10,89 | 11,16 | 0,965829 | -0,26 | 1,20 |
| RR4h0.1R3 | 3 | 1 | 0.1 µM 20E | 2 | 4 | 34,31 | 1,14148 | 34,31 | 24,36 | 9,95 | 11,16 | 0,965829 | -1,21 | 2,31 |
| RR8h0.1R1 | 1 | 1 | 0.1 µM 20E | 2 | 8 | 30,80 | 0,96806 | 30,80 | 20,95 | 9,85 | 11,16 | 0,965829 | -1,31 | 2,48 |
| RR8h0.1R2 | 2 | 1 | 0.1 µM 20E | 2 | 8 | 33,88 | 0,36599 | 33,89 | 23,19 | 10,70 | 11,16 | 0,965829 | -0,46 | 1,37 |
| RR8h0.1R3 | 3 | 1 | 0.1 µM 20E | 2 | 8 | 35,46 | 0,00000 | 35,47 | 24,48 | 10,99 | 11,16 | 0,965829 | -0,17 | 1,12 |
| PR1h0.1R1 | 1 | 2 | 0.1 µM 20E | 1 | NA | 29,33162 | 0,122173 | 29,33 | 22,27 | 7,06 | 12,36 | 1,143979 | -5,30 | 39,34 |
| PR1h0.1R2 | 2 | 2 | 0.1 µM 20E | 1 | NA | 29,06675 | 0,032673 | 29,07 | 22,09 | 6,98 | 12,36 | 1,143979 | -5,38 | 41,64 |
| PR1hCSR1 | 1 | 2 | 0.1 µM 20E | 1 | NA | 35,40215 | 1,689734 | 35,00 | 22,01 | 12,99 | 12,36 | 1,143979 |  |  |
| PR1hCSR2 | 2 | 2 | 0.1 µM 20E | 1 | NA | 34,85009 | 1,032164 | 34,85 | 22,33 | 12,52 | 12,36 | 1,143979 |  |  |
| PR2h0.1R1 | 1 | 2 | 0.1 µM 20E | 2 | NA | 29,19005 | 0,549948 | 29,19 | 21,82 | 7,37 | 12,36 | 1,143979 | -4,99 | 31,73 |
| PR2h0.1R2 | 2 | 2 | 0.1 µM 20E | 2 | NA | 29,42458 | 0,060458 | 29,42 | 21,66 | 7,77 | 12,36 | 1,143979 | -4,59 | 24,09 |
| PR2hCSR1 | 1 | 2 | 0.5% DMSO | 2 | NA | 34,22463 | 0,681694 | 34,22 | 22,09 | 12,13 | 12,36 | 1,143979 |  |  |
| PR2hCSR2 | 2 | 2 | 0.5% DMSO | 2 | NA | 35,41745 | 1,020607 | 35,00 | 22,06 | 12,94 | 12,36 | 1,143979 |  |  |
| PR30min0.1R1 | 1 | 2 | 0.1 µM 20E | 0,5 | NA | 31,48436 | 0,015575 | 31,48 | 23,06 | 8,43 | 12,36 | 1,143979 | -3,93 | 15,29 |
| PR30min0.1R2 | 2 | 2 | 0.1 µM 20E | 0,5 | NA | 31,19795 | 0,109973 | 31,20 | 22,21 | 8,99 | 12,36 | 1,143979 | -3,37 | 10,35 |
| PR30minCSR1 | 1 | 2 | 0.5% DMSO | 0,5 | NA | 35,68952 | 2,590948 | 35,00 | 21,97 | 13,03 | 12,36 | 1,143979 |  |  |
| PR30minCSR2 | 2 | 2 | 0.5% DMSO | 0,5 | NA | 35,62704 | 1,796431 | 35,00 | 22,57 | 12,43 | 12,36 | 1,143979 |  |  |
| R16hCSR1 | 1 | 2 | 0.5% DMSO | 18 | NA | 35,53661 | 0,94598 | 35,00 | 22,90 | 12,10 | 12,36 | 1,143979 |  |  |
| R16hCSR2 | 2 | 2 | 0.5% DMSO | 18 | NA | 33,61221 | 0 | 33,61 | 22,21 | 11,41 | 12,36 | 1,143979 |  |  |
| R1hCSR1 | 1 | 2 | 0.5% DMSO | 3 | NA | 31,93405 | 0,430989 | 31,93 | 23,14 | 8,79 | 12,36 | 1,143979 |  |  |
| R1hCSR2 | 2 | 2 | 0.5% DMSO | 3 | NA | 33,56943 | 1,083709 | 33,57 | 22,39 | 11,18 | 12,36 | 1,143979 |  |  |
| R2hCSR1 | 1 | 2 | 0.5% DMSO | 4 | NA | 35,15659 | 2,161031 | 35,00 | 22,43 | 12,57 | 12,36 | 1,143979 |  |  |
| R2hCSR2 | 2 | 2 | 0.5% DMSO | 4 | NA | 34,20983 | 1,138493 | 34,21 | 22,18 | 12,03 | 12,36 | 1,143979 |  |  |
| R4hCSR1 | 1 | 2 | 0.5% DMSO | 6 | NA | 34,9373 | 1,379592 | 34,94 | 22,44 | 12,50 | 12,36 | 1,143979 |  |  |
| R4hCSR2 | 2 | 2 | 0.5% DMSO | 6 | NA | 35,443 | 0 | 35,00 | 22,27 | 12,73 | 12,36 | 1,143979 |  |  |
| R8hCSR1 | 1 | 2 | 0.5% DMSO | 10 | NA | 35,63962 | 0,790506 | 35,00 | 22,66 | 12,34 | 12,36 | 1,143979 |  |  |
| R8hCSR2 | 2 | 2 | 0.5% DMSO | 10 | NA | 34,78991 | 0,635566 | 34,79 | 22,63 | 12,16 | 12,36 | 1,143979 |  |  |
| RC16h0.1R1 | 1 | 2 | 0.1 µM 20E | 18 | NA | 30,39323 | 1,584564 | 30,39 | 22,14 | 8,25 | 12,36 | 1,143979 | -4,11 | 17,22 |
| RC16h0.1R2 | 2 | 2 | 0.1 µM 20E | 18 | NA | 29,99937 | 0,293544 | 30,00 | 22,13 | 7,87 | 12,36 | 1,143979 | -4,49 | 22,47 |
| RC1h0.1R1 | 1 | 2 | 0.1 µM 20E | 3 | NA | 30,74067 | 2,332272 | 30,74 | 22,31 | 8,43 | 12,36 | 1,143979 | -3,93 | 15,21 |
| RC1h0.1R2 | 2 | 2 | 0.1 µM 20E | 3 | NA | 29,52669 | 0,119643 | 29,53 | 22,58 | 6,95 | 12,36 | 1,143979 | -5,41 | 42,55 |
| RC2h0.1R1 | 1 | 2 | 0.1 µM 20E | 4 | NA | 29,82877 | 2,682989 | 29,83 | 22,23 | 7,60 | 12,36 | 1,143979 | -4,76 | 27,16 |
| RC2h0.1R2 | 2 | 2 | 0.1 µM 20E | 4 | NA | 29,37558 | 0,412771 | 29,38 | 22,70 | 6,68 | 12,36 | 1,143979 | -5,68 | 51,43 |
| RC4h0.1R1 | 1 | 2 | 0.1 µM 20E | 6 | NA | 29,24744 | 1,563533 | 29,25 | 22,21 | 7,04 | 12,36 | 1,143979 | -5,32 | 39,88 |
| RC4h0.1R2 | 2 | 2 | 0.1 µM 20E | 6 | NA | 29,28167 | 0,092415 | 29,28 | 22,33 | 6,96 | 12,36 | 1,143979 | -5,40 | 42,36 |
| RC8h0.1R1 | 1 | 2 | 0.1 µM 20E | 10 | NA | 29,95294 | 1,559199 | 29,95 | 22,22 | 7,73 | 12,36 | 1,143979 | -4,63 | 24,77 |
| RC8h0.1R2 | 2 | 2 | 0.1 µM 20E | 10 | NA | 30,0787 | 0,48372 | 30,08 | 22,50 | 7,58 | 12,36 | 1,143979 | -4,78 | 27,48 |
| RR16h0.1R1 | 1 | 2 | 0.1 µM 20E | 2 | 16 | 36,07076 | 0,049569 | 35,00 | 22,06 | 12,94 | 12,36 | 1,143979 | 0,58 | 0,67 |
| RR16h0.1R2 | 2 | 2 | 0.1 µM 20E | 2 | 16 | 34,31728 | 0,753777 | 34,32 | 21,29 | 13,02 | 12,36 | 1,143979 | 0,66 | 0,63 |
| RR1h0.1R1 | 1 | 2 | 0.1 µM 20E | 2 | 1 | 31,82634 | 0 | 31,83 | 24,69 | 7,14 | 12,36 | 1,143979 | -5,22 | 37,35 |
| RR1h0.1R2 | 2 | 2 | 0.1 µM 20E | 2 | 1 | 30,82068 | 0,176508 | 30,82 | 22,55 | 8,27 | 12,36 | 1,143979 | -4,09 | 17,02 |
| RR2h0.1R1 | 1 | 2 | 0.1 µM 20E | 2 | 2 | 34,26088 | 2,3477 | 34,26 | 23,24 | 11,02 | 12,36 | 1,143979 | -1,34 | 2,54 |
| RR2h0.1R2 | 2 | 2 | 0.1 µM 20E | 2 | 2 | 33,78656 | 1,031187 | 33,79 | 22,25 | 11,53 | 12,36 | 1,143979 | -0,83 | 1,77 |
| RR4h0.1R1 | 1 | 2 | 0.1 µM 20E | 2 | 4 | 35,32358 | 1,848253 | 35,00 | 22,47 | 12,53 | 12,36 | 1,143979 | 0,17 | 0,89 |
| RR4h0.1R2 | 2 | 2 | 0.1 µM 20E | 2 | 4 | 34,65726 | 0,349448 | 34,66 | 22,09 | 12,57 | 12,36 | 1,143979 | 0,21 | 0,87 |
| RR8h0.1R1 | 1 | 2 | 0.1 µM 20E | 2 | 8 | 33,60601 | 1,863607 | 33,61 | 22,92 | 10,69 | 12,36 | 1,143979 | -1,67 | 3,19 |
| RR8h0.1R2 | 2 | 2 | 0.1 µM 20E | 2 | 8 | 35,1461 | 1,13765 | 35,00 | 22,18 | 12,82 | 12,36 | 1,143979 | 0,46 | 0,73 |
| PR1h0.1R1 | 1 | 3 | 0.1 µM 20E | 1 | NA | 29,15732 | 0,156825 | 29,16 | 21,66 | 7,50 | 10,80 | 1,602679 | -3,30 | 9,85 |
| PR1h0.1R2 | 2 | 3 | 0.1 µM 20E | 1 | NA | 28,88454 | 0,232599 | 28,88 | 22,08 | 6,81 | 10,80 | 1,602679 | -3,99 | 15,92 |
| PR1hCSR1 | 1 | 3 | 0.1 µM 20E | 1 | NA | 27,8849 | 0,130137 | 27,88 | 21,26 | 6,62 | 10,80 | 1,602679 |  |  |
| PR1hCSR2 | 2 | 3 | 0.1 µM 20E | 1 | NA | 33,40109 | 3,870248 | 33,40 | 21,94 | 11,46 | 10,80 | 1,602679 |  |  |
| PR2h0.1R1 | 1 | 3 | 0.1 µM 20E | 2 | NA | 30,81807 | 0,498393 | 30,82 | 22,27 | 8,55 | 10,80 | 1,602679 | -2,25 | 4,75 |
| PR2h0.1R2 | 2 | 3 | 0.1 µM 20E | 2 | NA | 29,81578 | 0,458966 | 29,82 | 22,90 | 6,91 | 10,80 | 1,602679 | -3,89 | 14,79 |
| PR2hCSR1 | 1 | 3 | 0.5% DMSO | 2 | NA | 37,1148 | 1,934262 | 35,00 | 21,14 | 13,86 | 10,80 | 1,602679 |  |  |
| PR2hCSR2 | 2 | 3 | 0.5% DMSO | 2 | NA | 35,74901 | 1,205562 | 35,00 | 22,37 | 12,63 | 10,80 | 1,602679 |  |  |
| PR30min0.1R1 | 1 | 3 | 0.1 µM 20E | 0,5 | NA | 30,43391 | 0,203708 | 30,43 | 22,13 | 8,30 | 10,80 | 1,602679 | -2,50 | 5,66 |
| PR30min0.1R2 | 2 | 3 | 0.1 µM 20E | 0,5 | NA | 30,63162 | 0,082263 | 30,63 | 22,43 | 8,20 | 10,80 | 1,602679 | -2,60 | 6,07 |
| PR30minCSR1 | 1 | 3 | 0.5% DMSO | 0,5 | NA | 33,95064 | 2,593599 | 33,95 | 22,05 | 11,90 | 10,80 | 1,602679 |  |  |
| PR30minCSR2 | 2 | 3 | 0.5% DMSO | 0,5 | NA | 35,5807 | 0,613977 | 35,00 | 21,65 | 13,35 | 10,80 | 1,602679 |  |  |
| R16hCSR1 | 1 | 3 | 0.5% DMSO | 18 | NA |  | 0 | 35,00 | 24,97 | 10,03 | 10,80 | 1,602679 |  |  |
| R16hCSR2 | 2 | 3 | 0.5% DMSO | 18 | NA |  | 0 | 35,00 | 24,64 | 10,36 | 10,80 | 1,602679 |  |  |
| R1hCSR1 | 1 | 3 | 0.5% DMSO | 3 | NA |  | 0 | 35,00 | 26,16 | 8,84 | 10,80 | 1,602679 |  |  |
| R1hCSR2 | 2 | 3 | 0.5% DMSO | 3 | NA | 38,7373 | 0,540007 | 35,00 | 24,71 | 10,29 | 10,80 | 1,602679 |  |  |
| R2hCSR1 | 1 | 3 | 0.5% DMSO | 4 | NA | 37,02096 | 0 | 35,00 | 25,86 | 9,14 | 10,80 | 1,602679 |  |  |
| R2hCSR2 | 2 | 3 | 0.5% DMSO | 4 | NA | 38,49614 | 0 | 35,00 | 25,37 | 9,63 | 10,80 | 1,602679 |  |  |
| R4hCSR1 | 1 | 3 | 0.5% DMSO | 6 | NA | 36,59633 | 0 | 35,00 | 26,51 | 8,49 | 10,80 | 1,602679 |  |  |
| R4hCSR2 | 2 | 3 | 0.5% DMSO | 6 | NA | 37,4142 | 0,347981 | 35,00 | 23,63 | 11,37 | 10,80 | 1,602679 |  |  |
| R8hCSR1 | 1 | 3 | 0.5% DMSO | 10 | NA | 38,10729 | 1,245775 | 35,00 | 25,82 | 9,18 | 10,80 | 1,602679 |  |  |
| R8hCSR2 | 2 | 3 | 0.5% DMSO | 10 | NA | 36,19829 | 0 | 35,00 | 23,53 | 11,47 | 10,80 | 1,602679 |  |  |
| RC16h0.1R1 | 1 | 3 | 0.1 µM 20E | 18 | NA | 30,60951 | 0,258986 | 30,61 | 22,76 | 7,85 | 10,80 | 1,602679 | -2,95 | 7,71 |
| RC16h0.1R2 | 2 | 3 | 0.1 µM 20E | 18 | NA | 31,33009 | 0,09079 | 31,33 | 22,70 | 8,63 | 10,80 | 1,602679 | -2,17 | 4,51 |
| RC1h0.1R1 | 1 | 3 | 0.1 µM 20E | 3 | NA | 31,96685 | 0,199319 | 31,97 | 24,27 | 7,69 | 10,80 | 1,602679 | -3,11 | 8,62 |
| RC1h0.1R2 | 2 | 3 | 0.1 µM 20E | 3 | NA | 32,744 | 1,575444 | 32,74 | 24,45 | 8,30 | 10,80 | 1,602679 | -2,51 | 5,68 |
| RC2h0.1R1 | 1 | 3 | 0.1 µM 20E | 4 | NA | 30,49161 | 0,968813 | 30,49 | 23,46 | 7,03 | 10,80 | 1,602679 | -3,77 | 13,64 |
| RC2h0.1R2 | 2 | 3 | 0.1 µM 20E | 4 | NA | 31,53394 | 0,949313 | 31,53 | 23,47 | 8,06 | 10,80 | 1,602679 | -2,74 | 6,68 |
| RC4h0.1R1 | 1 | 3 | 0.1 µM 20E | 6 | NA | 30,32917 | 0,933772 | 30,33 | 23,81 | 6,52 | 10,80 | 1,602679 | -4,28 | 19,49 |
| RC4h0.1R2 | 2 | 3 | 0.1 µM 20E | 6 | NA | 30,82389 | 0,893092 | 30,82 | 24,12 | 6,70 | 10,80 | 1,602679 | -4,10 | 17,11 |
| RC8h0.1R1 | 1 | 3 | 0.1 µM 20E | 10 | NA | 30,30299 | 0,331976 | 30,30 | 22,51 | 7,79 | 10,80 | 1,602679 | -3,01 | 8,06 |
| RC8h0.1R2 | 2 | 3 | 0.1 µM 20E | 10 | NA | 31,23096 | 0,592377 | 31,23 | 23,33 | 7,90 | 10,80 | 1,602679 | -2,90 | 7,47 |
| RR16h0.1R1 | 1 | 3 | 0.1 µM 20E | 2 | 16 | 35,93892 | 0 | 35,00 | 22,20 | 12,80 | 10,80 | 1,602679 | 1,99 | 0,25 |
| RR16h0.1R2 | 2 | 3 | 0.1 µM 20E | 2 | 16 | 34,2717 | 0 | 34,27 | 21,39 | 12,88 | 10,80 | 1,602679 | 2,08 | 0,24 |
| RR1h0.1R1 | 1 | 3 | 0.1 µM 20E | 2 | 1 | 34,58286 | 0,477556 | 34,58 | 23,37 | 11,22 | 10,80 | 1,602679 | 0,42 | 0,75 |
| RR1h0.1R2 | 2 | 3 | 0.1 µM 20E | 2 | 1 | 34,42156 | 1,013573 | 34,42 | 23,64 | 10,78 | 10,80 | 1,602679 | -0,02 | 1,01 |
| RR2h0.1R1 | 1 | 3 | 0.1 µM 20E | 2 | 2 | 35,43861 | 0 | 35,00 | 23,24 | 11,76 | 10,80 | 1,602679 | 0,96 | 0,51 |
| RR2h0.1R2 | 2 | 3 | 0.1 µM 20E | 2 | 2 | 34,95458 | 0 | 34,95 | 22,70 | 12,26 | 10,80 | 1,602679 | 1,46 | 0,36 |
| RR4h0.1R1 | 1 | 3 | 0.1 µM 20E | 2 | 4 | 34,13732 | 0 | 34,14 | 23,41 | 10,73 | 10,80 | 1,602679 | -0,07 | 1,05 |
| RR4h0.1R2 | 2 | 3 | 0.1 µM 20E | 2 | 4 | 36,29619 | 0 | 35,00 | 23,58 | 11,42 | 10,80 | 1,602679 | 0,62 | 0,65 |
| RR8h0.1R1 | 1 | 3 | 0.1 µM 20E | 2 | 8 | 34,91445 | 1,001845 | 34,91 | 22,76 | 12,16 | 10,80 | 1,602679 | 1,36 | 0,39 |
| RR8h0.1R2 | 2 | 3 | 0.1 µM 20E | 2 | 8 | 34,71439 | 0 | 34,71 | 22,54 | 12,17 | 10,80 | 1,602679 | 1,37 | 0,39 |
| 20ERC8h0.003R1 | 1 | 5 | 0.003 µM 20E | 10 | NA | 33,46764 | 1,189018 | 33,4676442 | 23,60 | 9,87 | 12,78 | 0,318721 | -2,91 | 7,53 |
| 20ERC8h0.003R2 | 2 | 5 | 0.003 µM 20E | 10 | NA | 33,64344 | 0,590311 | 33,6434383 | 23,18 | 10,47 | 12,78 | 0,318721 | -2,31 | 4,97 |
| 20ERC8h0.003R3 | 3 | 5 | 0.003 µM 20E | 10 | NA | 35,63268 | 0,591013 | 35 | 24,35 | 10,65 | 12,78 | 0,318721 | -2,13 | 4,38 |
| 20ERC8h0.01R1 | 1 | 5 | 0.01 µM 20E | 10 | NA | 33,5914 | 0,766674 | 33,59140235 | 22,42 | 11,17 | 12,78 | 0,318721 | -1,61 | 3,05 |
| 20ERC8h0.01R2 | 2 | 5 | 0.01 µM 20E | 10 | NA | 34,47849 | 0,475134 | 34,47848534 | 22,93 | 11,55 | 12,78 | 0,318721 | -1,23 | 2,34 |
| 20ERC8h0.01R3 | 3 | 5 | 0.01 µM 20E | 10 | NA | 34,60371 | 0,681851 | 34,60370803 | 22,95 | 11,65 | 12,78 | 0,318721 | -1,13 | 2,19 |
| 20ERC8h0.03R1 | 1 | 5 | 0.03 µM 20E | 10 | NA | 29,4859 | 0,056733 | 29,48590497 | 22,03 | 7,46 | 12,78 | 0,318721 | -5,32 | 40,07 |
| 20ERC8h0.03R2 | 2 | 5 | 0.03 µM 20E | 10 | NA | 30,45816 | 0,144335 | 30,45816239 | 22,17 | 8,29 | 12,78 | 0,318721 | -4,49 | 22,52 |
| 20ERC8h0.03R3 | 3 | 5 | 0.03 µM 20E | 10 | NA | 30,8643 | 0,212501 | 30,86429906 | 23,13 | 7,73 | 12,78 | 0,318721 | -5,05 | 33,05 |
| 20ERC8h0.1R1 | 1 | 5 | 0.1 µM 20E | 10 | NA | 28,20686 | 0,16155 | 28,20685767 | 22,27 | 5,94 | 12,78 | 0,318721 | -6,84 | 114,59 |
| 20ERC8h0.1R2 | 2 | 5 | 0.1 µM 20E | 10 | NA | 28,68756 | 0,347043 | 28,68756019 | 23,17 | 5,52 | 12,78 | 0,318721 | -7,26 | 153,46 |
| 20ERC8h0.1R3 | 3 | 5 | 0.1 µM 20E | 10 | NA | 29,2446 | 0,534108 | 29,24460259 | 23,08 | 6,16 | 12,78 | 0,318721 | -6,62 | 98,29 |
| 20ERC8h0.3R1 | 1 | 5 | 0.3 µM 20E | 10 | NA | 27,83317 | 0,427968 | 27,83317018 | 22,53 | 5,31 | 12,78 | 0,318721 | -7,47 | 177,77 |
| 20ERC8h0.3R2 | 2 | 5 | 0.3 µM 20E | 10 | NA | 27,97975 | 0,263436 | 27,97974609 | 22,47 | 5,51 | 12,78 | 0,318721 | -7,27 | 153,99 |
| 20ERC8h1R1 | 1 | 5 | 1 µM 20E | 10 | NA | 25,97797 | 0,067465 | 25,97797165 | 21,96 | 4,02 | 12,78 | 0,318721 | -8,76 | 434,85 |
| 20ERC8h1R2 | 2 | 5 | 1 µM 20E | 10 | NA | 28,36289 | 0,106433 | 28,36289064 | 25,05 | 3,31 | 12,78 | 0,318721 | -9,47 | 707,46 |
| 20ERC8h1R3 | 3 | 5 | 1 µM 20E | 10 | NA | 28,58316 | 0,485652 | 28,58315942 | 25,69 | 2,89 | 12,78 | 0,318721 | -9,89 | 947,69 |
| 20ERC8h3R1 | 1 | 5 | 3 µM 20E | 10 | NA | 25,985 | 0,077191 | 25,98499551 | 23,89 | 2,10 | 12,78 | 0,318721 | -10,68 | 1644,28 |
| 20ERC8h3R2 | 2 | 5 | 3 µM 20E | 10 | NA | 26,28697 | 0,01623 | 26,28697407 | 22,99 | 3,29 | 12,78 | 0,318721 | -9,49 | 716,66 |
| 20ERC8h3R3 | 3 | 5 | 3 µM 20E | 10 | NA | 26,26973 | 0,053553 | 26,26972926 | 23,01 | 3,26 | 12,78 | 0,318721 | -9,52 | 735,24 |
| 20ERR8h0.003R1 | 1 | 5 | 0.003 µM 20E | 2 | 8 | 35,26778 | 0,394863 | 35 | 22,12 | 12,88 | 12,78 | 0,318721 | 0,10 | 0,93 |
| 20ERR8h0.003R2 | 2 | 5 | 0.003 µM 20E | 2 | 8 | 35,56167 | 0,103062 | 35 | 21,83 | 13,17 | 12,78 | 0,318721 | 0,39 | 0,76 |
| 20ERR8h0.003R3 | 3 | 5 | 0.003 µM 20E | 2 | 8 | 34,53406 | 0,012167 | 35 | 22,43 | 12,57 | 12,78 | 0,318721 | -0,21 | 1,15 |
| 20ERR8h0.01R1 | 1 | 5 | 0.01 µM 20E | 2 | 8 | 35,82508 | 1,192376 | 35 | 22,58 | 12,42 | 12,78 | 0,318721 | -0,36 | 1,29 |
| 20ERR8h0.01R2 | 2 | 5 | 0.01 µM 20E | 2 | 8 | 34,16526 | 0,018937 | 34,16526129 | 22,19 | 11,97 | 12,78 | 0,318721 | -0,81 | 1,75 |
| 20ERR8h0.01R3 | 3 | 5 | 0.01 µM 20E | 2 | 8 | 37,45871 | 0 | 35 | 25,02 | 9,98 | 12,78 | 0,318721 | -2,80 | 6,97 |
| 20ERR8h0.03R1 | 1 | 5 | 0.03 µM 20E | 2 | 8 | 35,33055 | 0,791295 | 35 | 22,28 | 12,72 | 12,78 | 0,318721 | -0,06 | 1,04 |
| 20ERR8h0.03R2 | 2 | 5 | 0.03 µM 20E | 2 | 8 | 34,34499 | 1,760182 | 34,34499158 | 21,48 | 12,86 | 12,78 | 0,318721 | 0,08 | 0,94 |
| 20ERR8h0.03R3 | 3 | 5 | 0.03 µM 20E | 2 | 8 | 37,4981 | 0,898331 | 35 | 22,16 | 12,84 | 12,78 | 0,318721 | 0,06 | 0,96 |
| 20ERR8h0.1R1 | 1 | 5 | 0.1 µM 20E | 2 | 8 | 33,20206 | 0,083799 | 33,20206296 | 21,93 | 11,27 | 12,78 | 0,318721 | -1,51 | 2,85 |
| 20ERR8h0.1R2 | 2 | 5 | 0.1 µM 20E | 2 | 8 | 35,04338 | 0,009749 | 35 | 22,35 | 12,65 | 12,78 | 0,318721 | -0,13 | 1,09 |
| 20ERR8h0.1R3 | 3 | 5 | 0.1 µM 20E | 2 | 8 | 35,70224 | 2,023527 | 35 | 22,40 | 12,60 | 12,78 | 0,318721 | -0,18 | 1,14 |
| 20ERR8h0.3R1 | 1 | 5 | 0.3 µM 20E | 2 | 8 | 33,66928 | 0,179668 | 33,6692798 | 21,93 | 11,74 | 12,78 | 0,318721 | -1,04 | 2,05 |
| 20ERR8h0.3R2 | 2 | 5 | 0.3 µM 20E | 2 | 8 | 34,0599 | 1,375563 | 34,05990413 | 21,63 | 12,43 | 12,78 | 0,318721 | -0,35 | 1,27 |
| 20ERR8h0.3R3 | 3 | 5 | 0.3 µM 20E | 2 | 8 | 34,91435 | 1,569337 | 34,91434637 | 23,92 | 10,99 | 12,78 | 0,318721 | -1,79 | 3,46 |
| 20ERR8h1R1 | 1 | 5 | 1 µM 20E | 2 | 8 | 32,82922 | 0,071659 | 32,82922425 | 21,35 | 11,48 | 12,78 | 0,318721 | -1,30 | 2,46 |
| 20ERR8h1R2 | 2 | 5 | 1 µM 20E | 2 | 8 | 32,4447 | 0,046461 | 32,44469813 | 21,63 | 10,81 | 12,78 | 0,318721 | -1,97 | 3,91 |
| 20ERR8h1R3 | 3 | 5 | 1 µM 20E | 2 | 8 | 32,51005 | 1,101233 | 32,51004702 | 22,30 | 10,21 | 12,78 | 0,318721 | -2,57 | 5,93 |
| 20ERR8h3R1 | 1 | 5 | 3 µM 20E | 2 | 8 | 31,58148 | 0,028465 | 31,58147541 | 20,99 | 10,59 | 12,78 | 0,318721 | -2,19 | 4,57 |
| 20ERR8h3R2 | 2 | 5 | 3 µM 20E | 2 | 8 | 31,43612 | 0,104469 | 31,43612091 | 21,79 | 9,64 | 12,78 | 0,318721 | -3,14 | 8,81 |
| 20ERR8h3R3 | 3 | 5 | 3 µM 20E | 2 | 8 | 31,33827 | 0,157761 | 31,33827206 | 22,24 | 9,10 | 12,78 | 0,318721 | -3,68 | 12,86 |
| SC8hR1 | 1 | 5 | 0.5% DMSO | 10 | NA | 35,00828 | 0,591861 | 35 | 22,01 | 12,99 | 12,78 | 0,318721 |  |  |
| SC8hR2 | 2 | 5 | 0.5% DMSO | 10 | NA | 34,92558 | 0,182031 | 34,92558399 | 21,90 | 13,02 | 12,78 | 0,318721 |  |  |
| SC8hR3 | 3 | 5 | 0.5% DMSO | 10 | NA | 35,69946 | 0,489274 | 35 | 22,67 | 12,33 | 12,78 | 0,318721 |  |  |
| PR2h0.003R1 | 1 | 4 | 0.003 µM 20E | 2 | NA | 32,26424 | 0,32005 | 32,26424015 | 24,06650089 | 8,19773926 | 10,20 | 1,061061 | -2,00 | 4,00 |
| PR2h0.003R2 | 2 | 4 | 0.003 µM 20E | 2 | NA | 32,63952 | 0,308133 | 32,63951672 | 23,79508142 | 8,8444353 | 10,20 | 1,061061 | -1,36 | 2,56 |
| PR2h0.003R3 | 3 | 4 | 0.003 µM 20E | 2 | NA | 33,31441 | 0,155382 | 33,31441079 | 24,27232302 | 9,04208777 | 10,20 | 1,061061 | -1,16 | 2,23 |
| PR2h0.01R1 | 1 | 4 | 0.01 µM 20E | 2 | NA | 30,85579 | 0,402511 | 30,85578986 | 24,05947527 | 6,79631459 | 10,20 | 1,061061 | -3,40 | 10,58 |
| PR2h0.01R2 | 2 | 4 | 0.01 µM 20E | 2 | NA | 31,49178 | 0,13484 | 31,49178138 | 23,76239218 | 7,7293892 | 10,20 | 1,061061 | -2,47 | 5,54 |
| PR2h0.01R3 | 3 | 4 | 0.01 µM 20E | 2 | NA | 31,58262 | 0,042152 | 31,58261669 | 23,91807338 | 7,66454331 | 10,20 | 1,061061 | -2,53 | 5,80 |
| PR2h0.03R1 | 1 | 4 | 0.03 µM 20E | 2 | NA | 29,79994 | 0,013638 | 29,79994183 | 23,83681342 | 5,96312841 | 10,20 | 1,061061 | -4,24 | 18,85 |
| PR2h0.03R2 | 2 | 4 | 0.03 µM 20E | 2 | NA | 30,03572 | 0,047769 | 30,03572268 | 23,98816497 | 6,04755772 | 10,20 | 1,061061 | -4,15 | 17,78 |
| PR2h0.03R3 | 3 | 4 | 0.03 µM 20E | 2 | NA | 29,23701 | 0,019566 | 29,2370119 | 23,99708172 | 5,23993018 | 10,20 | 1,061061 | -4,96 | 31,12 |
| PR2h0.1R1 | 1 | 4 | 0.1 µM 20E | 2 | NA | 26,42226 | 0,083374 | 26,42225912 | 23,86297226 | 2,55928686 | 10,20 | 1,061061 | -7,64 | 199,49 |
| PR2h0.1R2 | 2 | 4 | 0.1 µM 20E | 2 | NA | 27,78711 | 0,246759 | 27,78710565 | 24,01000863 | 3,77709703 | 10,20 | 1,061061 | -6,42 | 85,77 |
| PR2h0.1R3 | 3 | 4 | 0.1 µM 20E | 2 | NA | 27,63176 | 0,078775 | 27,63175621 | 23,75794196 | 3,87381425 | 10,20 | 1,061061 | -6,33 | 80,21 |
| PR2h0.3R1 | 1 | 4 | 0.3 µM 20E | 2 | NA | 26,01712 | 0,200763 | 26,0171176 | 23,19081411 | 2,82630349 | 10,20 | 1,061061 | -7,37 | 165,79 |
| PR2h0.3R2 | 2 | 4 | 0.3 µM 20E | 2 | NA | 27,29161 | 0,061568 | 27,29160667 | 24,12390629 | 3,16770038 | 10,20 | 1,061061 | -7,03 | 130,85 |
| PR2h0.3R3 | 3 | 4 | 0.3 µM 20E | 2 | NA | 26,82159 | 0,138783 | 26,82158981 | 23,91917863 | 2,90241118 | 10,20 | 1,061061 | -7,30 | 157,27 |
| PR2h1R1 | 1 | 4 | 1 µM 20E | 2 | NA | 26,91158 | 0,078924 | 26,91157589 | 24,26299429 | 2,64858159 | 10,20 | 1,061061 | -7,55 | 187,52 |
| PR2h1R2 | 2 | 4 | 1 µM 20E | 2 | NA | 27,29106 | 0,139741 | 27,29105826 | 24,5015076 | 2,78955066 | 10,20 | 1,061061 | -7,41 | 170,06 |
| PR2h1R3 | 3 | 4 | 1 µM 20E | 2 | NA | 27,05217 | 0,020368 | 27,05216691 | 24,33668238 | 2,71548453 | 10,20 | 1,061061 | -7,48 | 179,02 |
| PR2h3R1 | 1 | 4 | 3 µM 20E | 2 | NA | 26,83404 | 0,121965 | 26,83404148 | 25,00208343 | 1,83195804 | 10,20 | 1,061061 | -8,37 | 330,28 |
| PR2h3R2 | 2 | 4 | 3 µM 20E | 2 | NA | 28,73193 | 0,010103 | 28,73193439 | 24,48061948 | 4,25131492 | 10,20 | 1,061061 | -5,95 | 61,74 |
| PR2h3R3 | 3 | 4 | 3 µM 20E | 2 | NA | 27,00896 | 0,215319 | 27,00896424 | 25,05831165 | 1,95065259 | 10,20 | 1,061061 | -8,25 | 304,19 |
| PR2hCSR1 | 1 | 4 | 0.5% DMSO | 2 | NA | 32,02062 | 1,156377 | 32,02062082 | 23,17631189 | 8,84430893 | 10,20 | 1,061061 |  |  |
| PR2hCSR2 | 2 | 4 | 0.5% DMSO | 2 | NA | 33,79566 | 0,420433 | 33,79565859 | 23,47662596 | 10,3190326 | 10,20 | 1,061061 |  |  |
| PR2hCSR3 | 3 | 4 | 0.5% DMSO | 2 | NA | 34,75847 | 1,180194 | 34,75846782 | 23,32336307 | 11,4351048 | 10,20 | 1,061061 |  |  |
| RC8h0.003R1 | 1 | 4 | 0.003 µM 20E | 10 | NA | 36,33656 | 0 | 35 | 25,33356426 | 9,66643574 | 10,86 | 0,44034 | -1,19 | 2,29 |
| RC8h0.003R2 | 2 | 4 | 0.003 µM 20E | 10 | NA | 35,02238 | 0,69174 | 35 | 24,1117651 | 10,8882349 | 10,86 | 0,44034 | 0,03 | 0,98 |
| RC8h0.003R3 | 3 | 4 | 0.003 µM 20E | 10 | NA | 37,09466 | 0,131232 | 35 | 24,03244502 | 10,967555 | 10,86 | 0,44034 | 0,11 | 0,93 |
| RC8h0.01R1 | 1 | 4 | 0.01 µM 20E | 10 | NA | 32,58275 | 0 | 32,58275325 | 24,72360096 | 7,85915229 | 10,86 | 0,44034 | -3,00 | 8,01 |
| RC8h0.01R2 | 2 | 4 | 0.01 µM 20E | 10 | NA | 33,5201 | 0,348381 | 33,52010446 | 24,09889467 | 9,42120979 | 10,86 | 0,44034 | -1,44 | 2,71 |
| RC8h0.01R3 | 3 | 4 | 0.01 µM 20E | 10 | NA | 33,67879 | 1,375567 | 33,67879388 | 23,83204493 | 9,84674895 | 10,86 | 0,44034 | -1,01 | 2,02 |
| RC8h0.03R1 | 1 | 4 | 0.03 µM 20E | 10 | NA | 31,64582 | 1,335903 | 31,64581965 | 24,08934449 | 7,55647516 | 10,86 | 0,44034 | -3,30 | 9,88 |
| RC8h0.03R2 | 2 | 4 | 0.03 µM 20E | 10 | NA | 28,25618 | 0,14532 | 28,25617583 | 23,0629046 | 5,19327122 | 10,86 | 0,44034 | -5,67 | 50,84 |
| RC8h0.03R3 | 3 | 4 | 0.03 µM 20E | 10 | NA | 31,22004 | 0,140654 | 31,22003837 | 23,9270167 | 7,29302167 | 10,86 | 0,44034 | -3,57 | 11,86 |
| RC8h0.1R1 | 1 | 4 | 0.1 µM 20E | 10 | NA | 30,51588 | 0,925108 | 30,51588436 | 25,60508325 | 4,9108011 | 10,86 | 0,44034 | -5,95 | 61,83 |
| RC8h0.1R2 | 2 | 4 | 0.1 µM 20E | 10 | NA | 29,68787 | 0,137025 | 29,68786974 | 24,75062075 | 4,93724899 | 10,86 | 0,44034 | -5,92 | 60,71 |
| RC8h0.1R3 | 3 | 4 | 0.1 µM 20E | 10 | NA | 29,47229 | 0,064841 | 29,47228772 | 24,38492629 | 5,08736143 | 10,86 | 0,44034 | -5,77 | 54,71 |
| RC8h0.3R1 | 1 | 4 | 0.3 µM 20E | 10 | NA | 28,95155 | 1,845977 | 28,95154867 | 25,03779239 | 3,91375628 | 10,86 | 0,44034 | -6,95 | 123,41 |
| RC8h0.3R2 | 2 | 4 | 0.3 µM 20E | 10 | NA | 28,30099 | 0,078146 | 28,30098666 | 24,99835833 | 3,30262833 | 10,86 | 0,44034 | -7,56 | 188,51 |
| RC8h0.3R3 | 3 | 4 | 0.3 µM 20E | 10 | NA | 27,92569 | 0,062332 | 27,92569408 | 25,15612733 | 2,76956675 | 10,86 | 0,44034 | -8,09 | 272,77 |
| RC8h1R1 | 1 | 4 | 1 µM 20E | 10 | NA | 27,41059 | 1,103698 | 27,41059259 | 25,66166746 | 1,74892513 | 10,86 | 0,44034 | -9,11 | 553,40 |
| RC8h1R2 | 2 | 4 | 1 µM 20E | 10 | NA | 25,97219 | 0,084988 | 25,97218751 | 25,14136453 | 0,83082298 | 10,86 | 0,44034 | -10,03 | 1045,72 |
| RC8h1R3 | 3 | 4 | 1 µM 20E | 10 | NA | 26,84234 | 0,25008 | 26,84234361 | 25,4551543 | 1,38718932 | 10,86 | 0,44034 | -9,47 | 711,10 |
| RC8h3R1 | 1 | 4 | 3 µM 20E | 10 | NA | 26,98608 | 0,071124 | 26,9860774 | 26,04497923 | 0,94109818 | 10,86 | 0,44034 | -9,92 | 968,77 |
| RC8h3R2 | 2 | 4 | 3 µM 20E | 10 | NA | 27,40426 | 0,154985 | 27,40425824 | 25,97859231 | 1,42566594 | 10,86 | 0,44034 | -9,44 | 692,39 |
| RC8h3R3 | 3 | 4 | 3 µM 20E | 10 | NA | 27,06338 | 0,180039 | 27,06337619 | 25,64677097 | 1,41660522 | 10,86 | 0,44034 | -9,44 | 696,75 |
| RC8hCSR1 | 1 | 4 | 0.5% DMSO | 10 | NA | 36,52372 | 0 | 35 | 24,55039242 | 10,4496076 | 10,86 | 0,44034 |  |  |
| RC8hCSR2 | 2 | 4 | 0.5% DMSO | 10 | NA | 35,21341 | 1,418009 | 35 | 24,86203113 | 10,1379689 | 10,86 | 0,44034 |  |  |
| RC8hCSR3 | 3 | 4 | 0.5% DMSO | 10 | NA | 35,49567 | 1,130368 | 35 | 24,16190411 | 10,8380959 | 10,86 | 0,44034 |  |  |
| RR8h0.003R1 | 1 | 4 | 0.003 µM 20E | 2 | 8 | 34,37067 | 0,061104 | 34,37067299 | 24,03374166 | 10,3369313 | 10,86 | 0,44034 | -0,52417 | 1,4381087 |
| RR8h0.003R2 | 2 | 4 | 0.003 µM 20E | 2 | 8 | 36,62032 | 2,128316 | 35 | 24,47529136 | 10,5247086 | 10,86 | 0,44034 | -0,3364 | 1,262598 |
| RR8h0.003R3 | 3 | 4 | 0.003 µM 20E | 2 | 8 | 37,05554 | 0,224824 | 35 | 24,18965883 | 10,8103412 | 10,86 | 0,44034 | -0,05076 | 1,0358125 |
| RR8h0.01R1 | 1 | 4 | 0.01 µM 20E | 2 | 8 | 34,58333 | 0,786315 | 34,5833281 | 24,2969812 | 10,2863469 | 10,86 | 0,44034 | -0,57476 | 1,4894267 |
| RR8h0.01R2 | 2 | 4 | 0.01 µM 20E | 2 | 8 | 35,64524 | 1,344005 | 35 | 24,3096332 | 10,6903668 | 10,86 | 0,44034 | -0,17074 | 1,1256335 |
| RR8h0.01R3 | 3 | 4 | 0.01 µM 20E | 2 | 8 | 35,43904 | 2,278132 | 35 | 23,74899753 | 11,2510025 | 10,86 | 0,44034 | 0,389898 | 0,7631833 |
| RR8h0.03R2 | 2 | 4 | 0.03 µM 20E | 2 | 8 | 36,38576 | 0,129652 | 35 | 24,76190518 | 10,2380948 | 10,86 | 0,44034 | -0,62301 | 1,5400842 |
| RR8h0.03R3 | 3 | 4 | 0.03 µM 20E | 2 | 8 | 34,34097 | 0,228665 | 34,34096815 | 23,63004873 | 10,7109194 | 10,86 | 0,44034 | -0,15018 | 1,1097115 |
| RR8h0.1R1 | 1 | 4 | 0.1 µM 20E | 2 | 8 | 34,63224 | 0,777966 | 34,63223561 | 23,91598832 | 10,7162473 | 10,86 | 0,44034 | -0,14486 | 1,1056209 |
| RR8h0.1R2 | 2 | 4 | 0.1 µM 20E | 2 | 8 | 32,50234 | 0,128897 | 32,50234495 | 23,83015246 | 8,67219249 | 10,86 | 0,44034 | -2,18891 | 4,5596135 |
| RR8h0.3R1 | 1 | 4 | 0.3 µM 20E | 2 | 8 | 34,03991 | 0,002122 | 34,03991293 | 24,69663993 | 9,34327299 | 10,86 | 0,44034 | -1,51783 | 2,8636021 |
| RR8h0.3R2 | 2 | 4 | 0.3 µM 20E | 2 | 8 | 33,20929 | 0,081673 | 33,20928843 | 23,93516496 | 9,27412347 | 10,86 | 0,44034 | -1,58698 | 3,0041994 |
| RR8h1R1 | 1 | 4 | 1 µM 20E | 2 | 8 | 33,77628 | 1,466613 | 33,77628364 | 24,5723365 | 9,20394714 | 10,86 | 0,44034 | -1,65716 | 3,1539437 |
| RR8h1R2 | 2 | 4 | 1 µM 20E | 2 | 8 | 33,25303 | 0,403437 | 33,25303384 | 23,92249298 | 9,33054086 | 10,86 | 0,44034 | -1,53056 | 2,8889859 |
| RR8h1R3 | 3 | 4 | 1 µM 20E | 2 | 8 | 35,01104 | 0,644448 | 35 | 24,3181916 | 10,6818084 | 10,86 | 0,44034 | -0,1793 | 1,1323309 |
| RR8h3R1 | 1 | 4 | 3 µM 20E | 2 | 8 | 31,86399 | 0,304416 | 31,86398695 | 25,5368918 | 6,32709514 | 10,86 | 0,44034 | -4,53401 | 23,167153 |
| RR8h3R2 | 2 | 4 | 3 µM 20E | 2 | 8 | 32,61197 | 0,126467 | 32,61196643 | 24,38143984 | 8,23052659 | 10,86 | 0,44034 | -2,63058 | 6,1927381 |
| RR8hCSR1 | 1 | 4 | 0.5% DMSO | 10 | NA | 34,89038 | 0,352255 | 34,89038056 | 23,6639561 | 11,2264245 | 10,86 | 0,44034 |  |  |
| RR8hCSR2 | 2 | 4 | 0.5% DMSO | 10 | NA | 34,53791 | 0,081926 | 34,53791418 | 23,17278357 | 11,3651306 | 10,86 | 0,44034 |  |  |
| RR8hCSR3 | 3 | 4 | 0.5% DMSO | 10 | NA | 35,02677 | 0,661594 | 35 | 23,85060323 | 11,1493968 | 10,86 | 0,44034 |  |  |
| 20ERC8h0.003R1 | 1 | 6 | 0.003 µM 20E | 10 | NA | 32,22128 | 0,248098 | 32,22128236 | 24,6740255 | 7,54725686 | 12,25 | 0,110228 | -4,70682 | 26,115214 |
| 20ERC8h0.003R2 | 2 | 6 | 0.003 µM 20E | 10 | NA | 33,03924 | 3,503841 | 33,03923552 | 25,05101999 | 7,98821553 | 12,25 | 0,110228 | -4,26586 | 19,237641 |
| 20ERC8h0.01R1 | 1 | 6 | 0.01 µM 20E | 10 | NA | 32,92331 | 0,457245 | 32,92331468 | 24,36954266 | 8,55377202 | 12,25 | 0,110228 | -3,7003 | 12,998772 |
| 20ERC8h0.01R2 | 2 | 6 | 0.01 µM 20E | 10 | NA | 33,3739 | 2,561288 | 33,37390044 | 23,18717505 | 10,1867254 | 12,25 | 0,110228 | -2,06735 | 4,1911615 |
| 20ERC8h0.01R3 | 3 | 6 | 0.01 µM 20E | 10 | NA | 32,90511 | 0,179893 | 32,90510928 | 24,46429689 | 8,44081239 | 12,25 | 0,110228 | -3,81326 | 14,057451 |
| 20ERC8h0.03R1 | 1 | 6 | 0.03 µM 20E | 10 | NA | 31,13459 | 0,044318 | 31,13459148 | 23,34814476 | 7,78644672 | 12,25 | 0,110228 | -4,46763 | 22,125356 |
| 20ERC8h0.03R3 | 3 | 6 | 0.03 µM 20E | 10 | NA | 31,47151 | 0,030734 | 31,47151178 | 24,10747574 | 7,36403604 | 12,25 | 0,110228 | -4,89004 | 29,651629 |
| 20ERC8h0.1R2 | 2 | 6 | 0.1 µM 20E | 10 | NA | 28,86829 | 1,94618 | 28,86828823 | 23,0638348 | 5,80445343 | 12,25 | 0,110228 | -6,44962 | 87,403677 |
| 20ERC8h0.1R3 | 3 | 6 | 0.1 µM 20E | 10 | NA | 28,05296 | 0,487687 | 28,05296048 | 24,10323852 | 3,94972196 | 12,25 | 0,110228 | -8,30435 | 316,12548 |
| 20ERC8h0.3R1 | 1 | 6 | 0.3 µM 20E | 10 | NA | 31,33939 | 0,42387 | 31,33939228 | 24,44131599 | 6,89807629 | 12,25 | 0,110228 | -5,356 | 40,955895 |
| 20ERC8h0.3R2 | 2 | 6 | 0.3 µM 20E | 10 | NA | 29,00922 | 1,700953 | 29,00921922 | 22,91674661 | 6,09247261 | 12,25 | 0,110228 | -6,1616 | 71,585867 |
| 20ERC8h0.3R3 | 3 | 6 | 0.3 µM 20E | 10 | NA | 28,10417 | 0,506213 | 28,10416925 | 23,73459404 | 4,36957521 | 12,25 | 0,110228 | -7,8845 | 236,30401 |
| 20ERC8h1R1 | 1 | 6 | 1 µM 20E | 10 | NA | 28,26558 | 0,128184 | 28,26558467 | 23,66687898 | 4,59870568 | 12,25 | 0,110228 | -7,65537 | 201,60251 |
| 20ERC8h1R2 | 2 | 6 | 1 µM 20E | 10 | NA | 28,38627 | 1,689386 | 28,38627403 | 24,9274558 | 3,45881823 | 12,25 | 0,110228 | -8,79526 | 444,25902 |
| 20ERC8h1R3 | 3 | 6 | 1 µM 20E | 10 | NA | 26,11015 | 0,286566 | 26,1101508 | 23,61815877 | 2,49199203 | 12,25 | 0,110228 | -9,76208 | 868,32028 |
| 20ERC8h3R1 | 1 | 6 | 3 µM 20E | 10 | NA | 28,15932 | 1,068499 | 28,15931661 | 23,39628549 | 4,76303112 | 12,25 | 0,110228 | -7,49104 | 179,89913 |
| 20ERC8h3R2 | 2 | 6 | 3 µM 20E | 10 | NA | 28,20488 | 1,367928 | 28,20488059 | 24,85654185 | 3,34833874 | 12,25 | 0,110228 | -8,90574 | 479,61626 |
| 20ERC8h3R3 | 3 | 6 | 3 µM 20E | 10 | NA | 26,10813 | 0,045229 | 26,10812668 | 24,26606522 | 1,84206146 | 12,25 | 0,110228 | -10,412 | 1362,475 |
| 20ERR8h0.01R2 | 2 | 6 | 0.01 µM 20E | 2 | 8 | 36,78074 | 3,753366 | 35 | 22,07952547 | 12,9204745 | 12,25 | 0,110228 | 0,666399 | 0,6300774 |
| 20ERR8h0.01R3 | 3 | 6 | 0.01 µM 20E | 2 | 8 | 37,9331 | 0,273253 | 35 | 22,81823793 | 12,1817621 | 12,25 | 0,110228 | -0,07231 | 1,0514013 |
| 20ERR8h0.03R3 | 3 | 6 | 0.03 µM 20E | 2 | 8 | 39,39397 | 0,307643 | 35 | 24,94826011 | 10,0517399 | 12,25 | 0,110228 | -2,20234 | 4,602238 |
| 20ERR8h0.1R1 | 1 | 6 | 0.1 µM 20E | 2 | 8 | 36,21719 | 1,671667 | 35 | 25,83004103 | 9,16995897 | 12,25 | 0,110228 | -3,08412 | 8,4803072 |
| 20ERR8h0.1R2 | 2 | 6 | 0.1 µM 20E | 2 | 8 | 35,8755 | 1,835101 | 35 | 22,46722676 | 12,5327732 | 12,25 | 0,110228 | 0,278698 | 0,8243348 |
| 20ERR8h0.1R3 | 3 | 6 | 0.1 µM 20E | 2 | 8 | 37,03879 | 1,193321 | 35 | 23,08837286 | 11,9116271 | 12,25 | 0,110228 | -0,34245 | 1,2679065 |
| 20ERR8h0.3R3 | 3 | 6 | 0.3 µM 20E | 2 | 8 | 35,95573 | 0,599631 | 35 | 22,69811513 | 12,3018849 | 12,25 | 0,110228 | 0,047809 | 0,9674041 |
| 20ERR8h1R2 | 2 | 6 | 1 µM 20E | 2 | 8 | 33,79873 | 1,504085 | 33,79873234 | 22,32129579 | 11,4774365 | 12,25 | 0,110228 | -0,77664 | 1,7131351 |
| 20ERR8h3R1 | 1 | 6 | 3 µM 20E | 2 | 8 | 32,13864 | 0,135309 | 32,13864101 | 23,40487294 | 8,73376806 | 12,25 | 0,110228 | -3,52031 | 11,474087 |
| 20ERR8h3R3 | 3 | 6 | 3 µM 20E | 2 | 8 | 34,52172 | 0,163864 | 34,52172481 | 22,96665096 | 11,5550739 | 12,25 | 0,110228 | -0,699 | 1,623381 |
| SC8hR2 | 2 | 6 | 0.5% DMSO | 10 | NA | 36,31546 | 3,074245 | 35 | 22,63569655 | 12,3643035 | 12,25 | 0,110228 |  |  |
| SC8hR3 | 3 | 6 | 0.5% DMSO | 10 | NA | 39,0282 | 0 | 35 | 22,85615246 | 12,1438475 | 12,25 | 0,110228 |  |  |

| sample id | replicate | experiment number | Treatment | exposure time/hours | recovery time/hours | Avg Cq | st.dev Cq | corrected Cq (max cq 35) | Refrence gene Cq | delta Cq | Avg. solvent control delta Cq | st.dev solvent control delta Cq | delta delta Cq | 2^ddCq |
| --- | --- | --- | --- | --- | --- | --- | --- | --- | --- | --- | --- | --- | --- | --- |
| PR1hA0.1R1 | 1 | 1 | 0.1 µM 20E | 1 | NA | 26,13 | 0,02089 | 34,73 | 23,08 | 11,65 | 11,88 | 0,5721929 | 0,96 | 0,51 |
| PR1hA0.1R2 | 2 | 1 | 0.1 µM 20E | 1 | NA | 28,26 | 0,00129 | 33,80 | 22,41 | 11,39 | 11,88 | 0,5721929 | 0,92 | 0,53 |
| PR1hA0.1R3 | 3 | 1 | 0.1 µM 20E | 1 | NA | 36,54 | 1,24381 | 35,23 | 23,03 | 12,21 | 11,88 | 0,5721929 | -0,59 | 1,51 |
| PR1hB0.1R1 | 1 | 1 | 0.1 µM 20E | 1 | NA | 29,57 | 0,14238 | 35,69 | 23,53 | 12,16 | 11,88 | 0,5721929 |  |  |
| PR1hB0.1R2 | 2 | 1 | 0.1 µM 20E | 1 | NA | 28,76 | 0,38295 | 32,35 | 22,44 | 9,91 | 11,88 | 0,5721929 |  |  |
| PR1hB0.1R3 | 3 | 1 | 0.1 µM 20E | 1 | NA | 36,57 | 0,16753 | 34,19 | 22,64 | 11,55 | 11,88 | 0,5721929 |  |  |
| PR1hCSR1 | 1 | 1 | 0.1 µM 20E | 1 | NA | 37,17 | 0,00000 | 35,00 | 23,32 | 11,68 | 11,88 | 0,5721929 |  |  |
| PR1hCSR2 | 2 | 1 | 0.1 µM 20E | 1 | NA | 35,34 | 1,63820 | 34,05 | 22,37 | 11,68 | 11,88 | 0,5721929 |  |  |
| PR1hCSR3 | 3 | 1 | 0.1 µM 20E | 1 | NA | 33,22 | 1,84902 | 34,26 | 22,81 | 11,45 | 11,88 | 0,5721929 |  |  |
| PR2hA0.1R1 | 1 | 1 | 0.1 µM 20E | 2 | NA | 26,03 | 0,09343 | 32,90 | 23,67 | 9,24 | 11,88 | 0,5721929 | -0,09 | 1,06 |
| PR2hA0.1R2 | 2 | 1 | 0.1 µM 20E | 2 | NA | 28,49 | 0,03464 | 30,91 | 22,77 | 8,14 | 11,88 | 0,5721929 | -1,35 | 2,55 |
| PR2hA0.1R3 | 3 | 1 | 0.1 µM 20E | 2 | NA | 29,40 | 0,30653 | 31,83 | 22,45 | 9,38 | 11,88 | 0,5721929 | -0,12 | 1,09 |
| PR2hB0.1R1 | 1 | 1 | 0.1 µM 20E | 2 | NA | 28,77 | 0,34167 | 34,06 | 23,98 | 10,08 | 11,88 | 0,5721929 |  |  |
| PR2hB0.1R2 | 2 | 1 | 0.1 µM 20E | 2 | NA | 28,28 | 0,31111 | 30,41 | 21,82 | 8,59 | 11,88 | 0,5721929 |  |  |
| PR2hB0.1R3 | 3 | 1 | 0.1 µM 20E | 2 | NA | 30,36 | 0,67995 | 31,70 | 22,32 | 9,39 | 11,88 | 0,5721929 |  |  |
| PR2hCSR1 | 1 | 1 | 0.5% DMSO | 2 | NA | 34,87 | 1,04142 | 35,00 | 23,19 | 11,81 | 11,88 | 0,5721929 |  |  |
| PR2hCSR2 | 2 | 1 | 0.5% DMSO | 2 | NA | 36,54 | 0,00000 | 34,77 | 22,41 | 12,36 | 11,88 | 0,5721929 |  |  |
| PR2hCSR3 | 3 | 1 | 0.5% DMSO | 2 | NA | 33,75 | 0,04970 | 35,22 | 23,14 | 12,08 | 11,88 | 0,5721929 |  |  |
| PR30minA0.1R1 | 1 | 1 | 0.1 µM 20E | 0,5 | NA | 26,71 | 0,26915 | 35,61 | 22,52 | 13,09 | 11,88 | 0,5721929 | -2,34 | 5,06 |
| PR30minA0.1R2 | 2 | 1 | 0.1 µM 20E | 0,5 | NA | 30,46 | 0,05462 | 35,29 | 22,37 | 12,92 | 11,88 | 0,5721929 | -3,63 | 12,40 |
| PR30minA0.1R3 | 3 | 1 | 0.1 µM 20E | 0,5 | NA | 36,15 | 0,00000 | 33,58 | 22,43 | 11,16 | 11,88 | 0,5721929 | -2,61 | 6,12 |
| PR30minB0.1R1 | 1 | 1 | 0.1 µM 20E | 0,5 | NA | 30,70 | 0,50500 | 35,25 | 22,42 | 12,83 | 11,88 | 0,5721929 |  |  |
| PR30minB0.1R2 | 2 | 1 | 0.1 µM 20E | 0,5 | NA | 30,88 | 0,16317 | 35,00 | 22,78 | 12,22 | 11,88 | 0,5721929 |  |  |
| PR30minB0.1R3 | 3 | 1 | 0.1 µM 20E | 0,5 | NA | 35,39 | 0,00000 | 35,44 | 22,98 | 12,46 | 11,88 | 0,5721929 |  |  |
| PR30minCSR1 | 1 | 1 | 0.5% DMSO | 0,5 | NA | 34,36 | 0,00000 | 35,44 | 23,12 | 12,32 | 11,88 | 0,5721929 |  |  |
| PR30minCSR2 | 2 | 1 | 0.5% DMSO | 0,5 | NA | 35,25 | 0,99541 | 35,30 | 23,05 | 12,24 | 11,88 | 0,5721929 |  |  |
| PR30minCSR3 | 3 | 1 | 0.5% DMSO | 0,5 | NA | 33,96 | 1,27104 | 35,00 | 22,64 | 12,36 | 11,88 | 0,5721929 |  |  |
| R16hCSR1 | 1 | 1 | 0.5% DMSO | 18 | NA | 34,34 | 0,00000 | 34,27 | 22,28 | 11,99 | 11,88 | 0,5721929 |  |  |
| R16hCSR2 | 2 | 1 | 0.5% DMSO | 18 | NA | 35,34 | 0,00000 | 35,22 | 23,12 | 12,10 | 11,88 | 0,5721929 |  |  |
| R16hCSR3 | 3 | 1 | 0.5% DMSO | 18 | NA | 32,14 | 0,09990 | 35,28 | 22,49 | 12,79 | 11,88 | 0,5721929 |  |  |
| R1hCSR1 | 1 | 1 | 0.5% DMSO | 3 | NA | 33,33 | 1,23050 | 35,00 | 23,00 | 12,00 | 11,88 | 0,5721929 |  |  |
| R1hCSR2 | 2 | 1 | 0.5% DMSO | 3 | NA | 35,51 | 0,00000 | 33,90 | 22,53 | 11,37 | 11,88 | 0,5721929 |  |  |
| R1hCSR3 | 3 | 1 | 0.5% DMSO | 3 | NA | 33,35 | 0,45068 | 35,42 | 23,79 | 11,63 | 11,88 | 0,5721929 |  |  |
| R2hCSR1 | 1 | 1 | 0.5% DMSO | 4 | NA | 33,85 | 0,24336 | 35,21 | 22,58 | 12,63 | 11,88 | 0,5721929 |  |  |
| R2hCSR2 | 2 | 1 | 0.5% DMSO | 4 | NA | 35,98 | 0,66578 | 34,39 | 22,43 | 11,96 | 11,88 | 0,5721929 |  |  |
| R2hCSR3 | 3 | 1 | 0.5% DMSO | 4 | NA | 33,08 | 1,03187 | 33,55 | 23,29 | 10,26 | 11,88 | 0,5721929 |  |  |
| R4hCSR1 | 1 | 1 | 0.5% DMSO | 6 | NA | 32,96 | 0,13681 | 34,22 | 22,95 | 11,27 | 11,88 | 0,5721929 |  |  |
| R4hCSR2 | 2 | 1 | 0.5% DMSO | 6 | NA | 36,70 | 0,17962 | 33,53 | 22,48 | 11,05 | 11,88 | 0,5721929 |  |  |
| R4hCSR3 | 3 | 1 | 0.5% DMSO | 6 | NA | 30,93 | 0,80456 | 34,75 | 23,30 | 11,45 | 11,88 | 0,5721929 |  |  |
| R8hCSR1 | 1 | 1 | 0.5% DMSO | 10 | NA | 34,46 | 0,81036 | 34,72 | 23,22 | 11,49 | 11,88 | 0,5721929 |  |  |
| R8hCSR2 | 2 | 1 | 0.5% DMSO | 10 | NA | 35,35 | 0,00000 | 35,30 | 22,55 | 12,74 | 11,88 | 0,5721929 |  |  |
| R8hCSR3 | 3 | 1 | 0.5% DMSO | 10 | NA | 35,12 | 2,98357 | 35,49 | 23,13 | 12,36 | 11,88 | 0,5721929 |  |  |
| RC16h0.1R1 | 1 | 1 | 0.1 µM 20E | 18 | NA | 29,08 | 0,14463 | 29,60 | 23,38 | 6,22 | 11,88 | 0,5721929 | -5,66 | 50,61 |
| RC16h0.1R2 | 2 | 1 | 0.1 µM 20E | 18 | NA | 30,76 | 0,39342 | 30,11 | 22,99 | 7,11 | 11,88 | 0,5721929 | -4,77 | 27,19 |
| RC16h0.1R3 | 3 | 1 | 0.1 µM 20E | 18 | NA | 28,04 | 0,03846 | 30,96 | 23,22 | 7,74 | 11,88 | 0,5721929 | -4,13 | 17,55 |
| RC1h0.1R1 | 1 | 1 | 0.1 µM 20E | 3 | NA | 29,25 | 0,09986 | 31,85 | 23,28 | 8,57 | 11,88 | 0,5721929 | -3,31 | 9,93 |
| RC1h0.1R2 | 2 | 1 | 0.1 µM 20E | 3 | NA | 30,06 | 0,33561 | 30,53 | 22,53 | 8,01 | 11,88 | 0,5721929 | -3,87 | 14,61 |
| RC1h0.1R3 | 3 | 1 | 0.1 µM 20E | 3 | NA | 31,64 | 0,66578 | 32,43 | 23,38 | 9,05 | 11,88 | 0,5721929 | -2,83 | 7,11 |
| RC2h0.1R1 | 1 | 1 | 0.1 µM 20E | 4 | NA | 28,92 | 0,60873 | 32,12 | 23,16 | 8,95 | 11,88 | 0,5721929 | -2,93 | 7,60 |
| RC2h0.1R2 | 2 | 1 | 0.1 µM 20E | 4 | NA | 29,12 | 0,06975 | 30,77 | 23,06 | 7,72 | 11,88 | 0,5721929 | -4,16 | 17,91 |
| RC2h0.1R3 | 3 | 1 | 0.1 µM 20E | 4 | NA | 28,84 | 0,65579 | 31,38 | 23,16 | 8,22 | 11,88 | 0,5721929 | -3,66 | 12,64 |
| RC4h0.1R1 | 1 | 1 | 0.1 µM 20E | 6 | NA | 28,53 | 0,64253 | 32,54 | 23,13 | 9,41 | 11,88 | 0,5721929 | -2,47 | 5,53 |
| RC4h0.1R2 | 2 | 1 | 0.1 µM 20E | 6 | NA | 29,73 | 0,00367 | 29,47 | 21,19 | 8,27 | 11,88 | 0,5721929 | -3,60 | 12,16 |
| RC4h0.1R3 | 3 | 1 | 0.1 µM 20E | 6 | NA | 30,99 | 0,65219 | 32,07 | 22,42 | 9,65 | 11,88 | 0,5721929 | -2,23 | 4,69 |
| RC8h0.1R1 | 1 | 1 | 0.1 µM 20E | 10 | NA | 29,12 | 0,49696 | 31,90 | 23,62 | 8,28 | 11,88 | 0,5721929 | -3,60 | 12,10 |
| RC8h0.1R2 | 2 | 1 | 0.1 µM 20E | 10 | NA | 27,61 | 0,01646 | 30,75 | 23,12 | 7,63 | 11,88 | 0,5721929 | -4,24 | 18,96 |
| RC8h0.1R3 | 3 | 1 | 0.1 µM 20E | 10 | NA | 31,02 | 0,91758 | 32,38 | 23,47 | 8,91 | 11,88 | 0,5721929 | -2,97 | 7,83 |
| RR16h0.1R1 | 1 | 1 | 0.1 µM 20E | 2 | 16 | 34,70 | 0,28067 | 34,32 | 21,89 | 12,43 | 11,88 | 0,5721929 | 0,55 | 0,68 |
| RR16h0.1R2 | 2 | 1 | 0.1 µM 20E | 2 | 16 | 36,68 | 1,40401 | 34,16 | 22,71 | 11,45 | 11,88 | 0,5721929 | -0,43 | 1,34 |
| RR16h0.1R3 | 3 | 1 | 0.1 µM 20E | 2 | 16 | 33,38 | 0,15175 | 35,06 | 23,39 | 11,67 | 11,88 | 0,5721929 | -0,21 | 1,15 |
| RR1h0.1R1 | 1 | 1 | 0.1 µM 20E | 2 | 1 | 29,93 | 0,14108 | 30,91 | 23,21 | 7,70 | 11,88 | 0,5721929 | -4,18 | 18,10 |
| RR1h0.1R2 | 2 | 1 | 0.1 µM 20E | 2 | 1 | 31,39 | 0,02609 | 30,39 | 22,62 | 7,77 | 11,88 | 0,5721929 | -4,11 | 17,25 |
| RR1h0.1R3 | 3 | 1 | 0.1 µM 20E | 2 | 1 | 33,06 | 0,00561 | 32,15 | 23,32 | 8,83 | 11,88 | 0,5721929 | -3,04 | 8,25 |
| RR2h0.1R1 | 1 | 1 | 0.1 µM 20E | 2 | 2 | 29,19 | 0,22069 | 33,45 | 23,53 | 9,93 | 11,88 | 0,5721929 | -1,95 | 3,86 |
| RR2h0.1R2 | 2 | 1 | 0.1 µM 20E | 2 | 2 | 32,43 | 0,06168 | 31,60 | 23,04 | 8,56 | 11,88 | 0,5721929 | -3,32 | 9,98 |
| RR2h0.1R3 | 3 | 1 | 0.1 µM 20E | 2 | 2 | 32,55 | 0,25455 | 32,31 | 22,77 | 9,54 | 11,88 | 0,5721929 | -2,34 | 5,05 |
| RR4h0.1R1 | 1 | 1 | 0.1 µM 20E | 2 | 4 | 30,83 | 0,52069 | 34,85 | 23,63 | 11,22 | 11,88 | 0,5721929 | -0,66 | 1,58 |
| RR4h0.1R2 | 2 | 1 | 0.1 µM 20E | 2 | 4 | 34,34 | 0,54199 | 32,58 | 22,32 | 10,27 | 11,88 | 0,5721929 | -1,61 | 3,05 |
| RR4h0.1R3 | 3 | 1 | 0.1 µM 20E | 2 | 4 | 34,31 | 1,14148 | 34,45 | 21,55 | 12,90 | 11,88 | 0,5721929 | 1,02 | 0,49 |
| RR8h0.1R1 | 1 | 1 | 0.1 µM 20E | 2 | 8 | 30,80 | 0,96806 | 34,93 | 23,33 | 11,60 | 11,88 | 0,5721929 | -0,28 | 1,21 |
| RR8h0.1R2 | 2 | 1 | 0.1 µM 20E | 2 | 8 | 33,88 | 0,36599 | 33,95 | 23,17 | 10,77 | 11,88 | 0,5721929 | -1,10 | 2,15 |
| RR8h0.1R3 | 3 | 1 | 0.1 µM 20E | 2 | 8 | 35,46 | 0,00000 | 35,27 | 21,18 | 14,09 | 11,88 | 0,5721929 | 2,21 | 0,22 |
| PR1h0.1R1 | 1 | 2 | 0.1 µM 20E | 1 | NA | 33,45258 | 0,108262 | 33,45 | 22,27 | 11,18 | 11,94 | 1,2424128 | -0,76 | 1,69 |
| PR1h0.1R2 | 2 | 2 | 0.1 µM 20E | 1 | NA | 32,45378 | 1,336066 | 32,45 | 22,09 | 10,37 | 11,94 | 1,2424128 | -1,57 | 2,97 |
| PR1hCSR1 | 1 | 2 | 0.1 µM 20E | 1 | NA | 34,55936 | 1,219662 | 34,56 | 22,01 | 12,54 | 11,94 | 1,2424128 |  |  |
| PR1hCSR2 | 2 | 2 | 0.1 µM 20E | 1 | NA | 36,78722 | 0 | 35,00 | 22,33 | 12,67 | 11,94 | 1,2424128 |  |  |
| PR2h0.1R1 | 1 | 2 | 0.1 µM 20E | 2 | NA | 30,29182 | 0,274309 | 30,29 | 21,82 | 8,47 | 11,94 | 1,2424128 | -3,47 | 11,05 |
| PR2h0.1R2 | 2 | 2 | 0.1 µM 20E | 2 | NA | 30,4211 | 0,10991 | 30,42 | 21,66 | 8,77 | 11,94 | 1,2424128 | -3,17 | 9,02 |
| PR2hCSR1 | 1 | 2 | 0.5% DMSO | 2 | NA | 37,50535 | 0,524069 | 35,00 | 22,09 | 12,91 | 11,94 | 1,2424128 |  |  |
| PR2hCSR2 | 2 | 2 | 0.5% DMSO | 2 | NA | 34,9399 | 0 | 34,94 | 22,06 | 12,88 | 11,94 | 1,2424128 |  |  |
| PR30min0.1R1 | 1 | 2 | 0.1 µM 20E | 0,5 | NA | 33,82334 | 1,449019 | 33,82 | 23,06 | 10,76 | 11,94 | 1,2424128 | -1,18 | 2,26 |
| PR30min0.1R2 | 2 | 2 | 0.1 µM 20E | 0,5 | NA | 33,56875 | 0,681356 | 33,57 | 22,21 | 11,36 | 11,94 | 1,2424128 | -0,58 | 1,50 |
| PR30minCSR1 | 1 | 2 | 0.5% DMSO | 0,5 | NA | 33,8221 | 0,79195 | 33,82 | 21,97 | 11,85 | 11,94 | 1,2424128 |  |  |
| PR30minCSR2 | 2 | 2 | 0.5% DMSO | 0,5 | NA | 34,64721 | 1,270184 | 34,65 | 22,57 | 12,08 | 11,94 | 1,2424128 |  |  |
| R16hCSR1 | 1 | 2 | 0.5% DMSO | 18 | NA | 34,72098 | 1,043274 | 34,72 | 22,90 | 11,82 | 11,94 | 1,2424128 |  |  |
| R16hCSR2 | 2 | 2 | 0.5% DMSO | 18 | NA | 35,30764 | 3,30215 | 35,00 | 22,21 | 12,79 | 11,94 | 1,2424128 |  |  |
| R1hCSR1 | 1 | 2 | 0.5% DMSO | 3 | NA | 33,07147 | 0,326787 | 33,07 | 23,14 | 9,93 | 11,94 | 1,2424128 |  |  |
| R1hCSR2 | 2 | 2 | 0.5% DMSO | 3 | NA | 33,26616 | 0 | 33,27 | 22,39 | 10,88 | 11,94 | 1,2424128 |  |  |
| R2hCSR1 | 1 | 2 | 0.5% DMSO | 4 | NA | 33,94622 | 0,813798 | 33,95 | 22,43 | 11,52 | 11,94 | 1,2424128 |  |  |
| R2hCSR2 | 2 | 2 | 0.5% DMSO | 4 | NA | 31,35557 | 0,010344 | 31,36 | 22,18 | 9,17 | 11,94 | 1,2424128 |  |  |
| R4hCSR1 | 1 | 2 | 0.5% DMSO | 6 | NA | 34,27507 | 0,731629 | 34,28 | 22,44 | 11,83 | 11,94 | 1,2424128 |  |  |
| R4hCSR2 | 2 | 2 | 0.5% DMSO | 6 | NA | 33,88232 | 0,743151 | 33,88 | 22,27 | 11,61 | 11,94 | 1,2424128 |  |  |
| R8hCSR1 | 1 | 2 | 0.5% DMSO | 10 | NA | 35,74838 | 0,701565 | 35,00 | 22,66 | 12,34 | 11,94 | 1,2424128 |  |  |
| R8hCSR2 | 2 | 2 | 0.5% DMSO | 10 | NA | 33,98432 | 0,237872 | 33,98 | 22,63 | 11,36 | 11,94 | 1,2424128 |  |  |
| RC16h0.1R1 | 1 | 2 | 0.1 µM 20E | 18 | NA | 29,12485 | 1,526131 | 29,12 | 22,14 | 6,99 | 11,94 | 1,2424128 | -4,95 | 31,00 |
| RC16h0.1R2 | 2 | 2 | 0.1 µM 20E | 18 | NA | 30,95045 | 0,228676 | 30,95 | 22,13 | 8,82 | 11,94 | 1,2424128 | -3,12 | 8,68 |
| RC1h0.1R1 | 1 | 2 | 0.1 µM 20E | 3 | NA | 29,37785 | 0,036447 | 29,38 | 22,31 | 7,07 | 11,94 | 1,2424128 | -4,87 | 29,24 |
| RC1h0.1R2 | 2 | 2 | 0.1 µM 20E | 3 | NA | 30,91023 | 0,350231 | 30,91 | 22,58 | 8,33 | 11,94 | 1,2424128 | -3,61 | 12,19 |
| RC2h0.1R1 | 1 | 2 | 0.1 µM 20E | 4 | NA | 29,25169 | 1,408535 | 29,25 | 22,23 | 7,02 | 11,94 | 1,2424128 | -4,92 | 30,28 |
| RC2h0.1R2 | 2 | 2 | 0.1 µM 20E | 4 | NA | 29,95722 | 0,421818 | 29,96 | 22,70 | 7,26 | 11,94 | 1,2424128 | -4,68 | 25,68 |
| RC4h0.1R1 | 1 | 2 | 0.1 µM 20E | 6 | NA | 29,85868 | 0,434636 | 29,86 | 22,21 | 7,65 | 11,94 | 1,2424128 | -4,29 | 19,51 |
| RC4h0.1R2 | 2 | 2 | 0.1 µM 20E | 6 | NA | 30,84995 | 0,586756 | 30,85 | 22,33 | 8,52 | 11,94 | 1,2424128 | -3,42 | 10,67 |
| RC8h0.1R1 | 1 | 2 | 0.1 µM 20E | 10 | NA | 30,77398 | 0,943214 | 30,77 | 22,22 | 8,55 | 11,94 | 1,2424128 | -3,39 | 10,48 |
| RC8h0.1R2 | 2 | 2 | 0.1 µM 20E | 10 | NA | 30,76829 | 0,377686 | 30,77 | 22,50 | 8,27 | 11,94 | 1,2424128 | -3,67 | 12,73 |
| RR16h0.1R1 | 1 | 2 | 0.1 µM 20E | 2 | 16 | 35,61315 | 2,228015 | 35,00 | 22,06 | 12,94 | 11,94 | 1,2424128 | 1,00 | 0,50 |
| RR16h0.1R2 | 2 | 2 | 0.1 µM 20E | 2 | 16 | 32,20835 | 0,192683 | 32,21 | 21,29 | 10,92 | 11,94 | 1,2424128 | -1,02 | 2,03 |
| RR1h0.1R1 | 1 | 2 | 0.1 µM 20E | 2 | 1 | 32,87312 | 2,473567 | 32,87 | 24,69 | 8,18 | 11,94 | 1,2424128 | -3,76 | 13,51 |
| RR1h0.1R2 | 2 | 2 | 0.1 µM 20E | 2 | 1 | 29,96081 | 0,166639 | 29,96 | 22,55 | 7,41 | 11,94 | 1,2424128 | -4,53 | 23,08 |
| RR2h0.1R1 | 1 | 2 | 0.1 µM 20E | 2 | 2 | 30,63701 | 0,565715 | 30,64 | 23,24 | 7,39 | 11,94 | 1,2424128 | -4,55 | 23,37 |
| RR2h0.1R2 | 2 | 2 | 0.1 µM 20E | 2 | 2 | 30,31499 | 0,459126 | 30,31 | 22,25 | 8,06 | 11,94 | 1,2424128 | -3,88 | 14,71 |
| RR4h0.1R1 | 1 | 2 | 0.1 µM 20E | 2 | 4 | 32,58862 | 0,30802 | 32,59 | 22,47 | 10,12 | 11,94 | 1,2424128 | -1,82 | 3,54 |
| RR4h0.1R2 | 2 | 2 | 0.1 µM 20E | 2 | 4 | 31,78765 | 0,650684 | 31,79 | 22,09 | 9,70 | 11,94 | 1,2424128 | -2,24 | 4,73 |
| RR8h0.1R1 | 1 | 2 | 0.1 µM 20E | 2 | 8 | 33,68643 | 0,800209 | 33,69 | 22,92 | 10,77 | 11,94 | 1,2424128 | -1,17 | 2,25 |
| RR8h0.1R2 | 2 | 2 | 0.1 µM 20E | 2 | 8 | 33,09137 | 1,480321 | 33,09 | 22,18 | 10,91 | 11,94 | 1,2424128 | -1,03 | 2,04 |
| PR1h0.1R1 | 1 | 3 | 0.1 µM 20E | 1 | NA | 32,19473 | 0,701136 | 32,19 | 21,66 | 10,54 | 11,62 | 1,5134185 | -1,08 | 2,12 |
| PR1h0.1R2 | 2 | 3 | 0.1 µM 20E | 1 | NA | 33,30109 | 0,390174 | 33,30 | 22,08 | 11,22 | 11,62 | 1,5134185 | -0,40 | 1,31 |
| PR1hCSR1 | 1 | 3 | 0.1 µM 20E | 1 | NA | 34,9223 | 1,196143 | 34,92 | 21,26 | 13,66 | 11,62 | 1,5134185 |  |  |
| PR1hCSR2 | 2 | 3 | 0.1 µM 20E | 1 | NA | 35,0368 | 1,40334 | 35,04 | 21,94 | 13,10 | 11,62 | 1,5134185 |  |  |
| PR2h0.1R1 | 1 | 3 | 0.1 µM 20E | 2 | NA | 31,75133 | 0,060638 | 31,75 | 22,27 | 9,48 | 11,62 | 1,5134185 | -2,13 | 4,39 |
| PR2h0.1R2 | 2 | 3 | 0.1 µM 20E | 2 | NA | 31,28039 | 0,133039 | 31,28 | 22,90 | 8,38 | 11,62 | 1,5134185 | -3,24 | 9,45 |
| PR2hCSR1 | 1 | 3 | 0.5% DMSO | 2 | NA |  | 0 | 35,00 | 21,14 | 13,86 | 11,62 | 1,5134185 |  |  |
| PR2hCSR2 | 2 | 3 | 0.5% DMSO | 2 | NA | 35,35792 | 0 | 35,36 | 22,37 | 12,98 | 11,62 | 1,5134185 |  |  |
| PR30min0.1R1 | 1 | 3 | 0.1 µM 20E | 0,5 | NA |  | 0 | 35,00 | 22,13 | 12,87 | 11,62 | 1,5134185 | 1,25 | 0,42 |
| PR30min0.1R2 | 2 | 3 | 0.1 µM 20E | 0,5 | NA |  | 0 | 35,00 | 22,43 | 12,57 | 11,62 | 1,5134185 | 0,95 | 0,52 |
| PR30minCSR1 | 1 | 3 | 0.5% DMSO | 0,5 | NA |  | 0 | 35,00 | 22,05 | 12,95 | 11,62 | 1,5134185 |  |  |
| PR30minCSR2 | 2 | 3 | 0.5% DMSO | 0,5 | NA | 34,90555 | 0 | 34,91 | 21,65 | 13,25 | 11,62 | 1,5134185 |  |  |
| R16hCSR1 | 1 | 3 | 0.5% DMSO | 18 | NA | 35,09497 | 0 | 35,09 | 24,48 | 10,61 | 11,62 | 1,5134185 |  |  |
| R16hCSR2 | 2 | 3 | 0.5% DMSO | 18 | NA | 35,00142 | 0 | 35,00 | 23,35 | 11,66 | 11,62 | 1,5134185 |  |  |
| R1hCSR1 | 1 | 3 | 0.5% DMSO | 3 | NA |  | 0 | 35,00 | 24,75 | 10,25 | 11,62 | 1,5134185 |  |  |
| R1hCSR2 | 2 | 3 | 0.5% DMSO | 3 | NA |  | 0 | 35,00 | 24,29 | 10,71 | 11,62 | 1,5134185 |  |  |
| R2hCSR1 | 1 | 3 | 0.5% DMSO | 4 | NA | 35,0176 | 0 | 35,02 | 25,73 | 9,29 | 11,62 | 1,5134185 |  |  |
| R2hCSR2 | 2 | 3 | 0.5% DMSO | 4 | NA | 36,26988 | 0 | 35,00 | 24,39 | 10,61 | 11,62 | 1,5134185 |  |  |
| R4hCSR1 | 1 | 3 | 0.5% DMSO | 6 | NA | 35,9433 | 1,185259 | 35,00 | 23,98 | 11,02 | 11,62 | 1,5134185 |  |  |
| R4hCSR2 | 2 | 3 | 0.5% DMSO | 6 | NA | 33,80919 | 1,766521 | 33,81 | 24,99 | 8,82 | 11,62 | 1,5134185 |  |  |
| R8hCSR1 | 1 | 3 | 0.5% DMSO | 10 | NA |  | 0 | 35,00 | 23,99 | 11,01 | 11,62 | 1,5134185 |  |  |
| R8hCSR2 | 2 | 3 | 0.5% DMSO | 10 | NA |  | 0 | 35,00 | 22,87 | 12,13 | 11,62 | 1,5134185 |  |  |
| RC16h0.1R1 | 1 | 3 | 0.1 µM 20E | 18 | NA | 31,82232 | 0,252156 | 31,82 | 22,76 | 9,07 | 11,62 | 1,5134185 | -2,55 | 5,87 |
| RC16h0.1R2 | 2 | 3 | 0.1 µM 20E | 18 | NA | 32,97282 | 1,655658 | 32,97 | 22,70 | 10,27 | 11,62 | 1,5134185 | -1,35 | 2,55 |
| RC1h0.1R1 | 1 | 3 | 0.1 µM 20E | 3 | NA | 33,13651 | 0 | 33,14 | 24,27 | 8,86 | 11,62 | 1,5134185 | -2,76 | 6,76 |
| RC1h0.1R2 | 2 | 3 | 0.1 µM 20E | 3 | NA | 33,24631 | 0,638401 | 33,25 | 24,45 | 8,80 | 11,62 | 1,5134185 | -2,82 | 7,07 |
| RC2h0.1R1 | 1 | 3 | 0.1 µM 20E | 4 | NA | 31,74863 | 0,588143 | 31,75 | 23,46 | 8,29 | 11,62 | 1,5134185 | -3,33 | 10,07 |
| RC2h0.1R2 | 2 | 3 | 0.1 µM 20E | 4 | NA | 32,27046 | 0,333191 | 32,27 | 23,47 | 8,80 | 11,62 | 1,5134185 | -2,82 | 7,07 |
| RC4h0.1R1 | 1 | 3 | 0.1 µM 20E | 6 | NA | 32,06277 | 0,160578 | 32,06 | 23,81 | 8,25 | 11,62 | 1,5134185 | -3,37 | 10,34 |
| RC4h0.1R2 | 2 | 3 | 0.1 µM 20E | 6 | NA | 32,32864 | 0,7131 | 32,33 | 24,12 | 8,21 | 11,62 | 1,5134185 | -3,41 | 10,64 |
| RC8h0.1R1 | 1 | 3 | 0.1 µM 20E | 10 | NA | 33,7488 | 1,186085 | 33,75 | 22,51 | 11,23 | 11,62 | 1,5134185 | -0,38 | 1,31 |
| RC8h0.1R2 | 2 | 3 | 0.1 µM 20E | 10 | NA | 32,58543 | 1,209468 | 32,59 | 23,33 | 9,25 | 11,62 | 1,5134185 | -2,37 | 5,15 |
| RR16h0.1R1 | 1 | 3 | 0.1 µM 20E | 2 | 16 |  | 0 | 35,00 | 22,20 | 12,80 | 11,62 | 1,5134185 | 1,18 | 0,44 |
| RR16h0.1R2 | 2 | 3 | 0.1 µM 20E | 2 | 16 | 34,24658 | 0,514135 | 34,25 | 21,39 | 12,85 | 11,62 | 1,5134185 | 1,24 | 0,42 |
| RR1h0.1R1 | 1 | 3 | 0.1 µM 20E | 2 | 1 | 33,75481 | 0,234771 | 33,75 | 23,37 | 10,39 | 11,62 | 1,5134185 | -1,23 | 2,35 |
| RR1h0.1R2 | 2 | 3 | 0.1 µM 20E | 2 | 1 | 32,32943 | 0,222877 | 32,33 | 23,64 | 8,69 | 11,62 | 1,5134185 | -2,93 | 7,62 |
| RR2h0.1R1 | 1 | 3 | 0.1 µM 20E | 2 | 2 | 33,29463 | 0,091837 | 33,29 | 23,24 | 10,05 | 11,62 | 1,5134185 | -1,57 | 2,96 |
| RR2h0.1R2 | 2 | 3 | 0.1 µM 20E | 2 | 2 | 34,06894 | 0,169812 | 34,07 | 22,70 | 11,37 | 11,62 | 1,5134185 | -0,25 | 1,19 |
| RR4h0.1R1 | 1 | 3 | 0.1 µM 20E | 2 | 4 | 36,2777 | 0 | 35,00 | 23,41 | 11,59 | 11,62 | 1,5134185 | -0,03 | 1,02 |
| RR4h0.1R2 | 2 | 3 | 0.1 µM 20E | 2 | 4 | 34,63747 | 0,567814 | 34,64 | 23,58 | 11,06 | 11,62 | 1,5134185 | -0,56 | 1,48 |
| RR8h0.1R1 | 1 | 3 | 0.1 µM 20E | 2 | 8 | 34,32614 | 1,698674 | 34,33 | 22,76 | 11,57 | 11,62 | 1,5134185 | -0,05 | 1,03 |
| RR8h0.1R2 | 2 | 3 | 0.1 µM 20E | 2 | 8 | 34,9017 | 0 | 34,90 | 22,54 | 12,36 | 11,62 | 1,5134185 | 0,74 | 0,60 |
| 20ERC8h0.003R1 | 1 | 5 | 0.003 µM 20E | 10 | NA | 33,68594 | 0 | 33,68594213 | 23,60101772 | 10,0849244 | 12,58 | 0,291709 | -2,50 | 5,64 |
| 20ERC8h0.003R2 | 2 | 5 | 0.003 µM 20E | 10 | NA | 34,81871 | 0 | 34,81870508 | 23,17600444 | 11,6427006 | 12,58 | 0,291709 | -0,94 | 1,91 |
| 20ERC8h0.003R3 | 3 | 5 | 0.003 µM 20E | 10 | NA | 35,65442 | 0 | 35 | 24,34962991 | 10,6503701 | 12,58 | 0,291709 | -1,93 | 3,81 |
| 20ERC8h0.01R1 | 1 | 5 | 0.01 µM 20E | 10 | NA | 32,90645 | 1,060598 | 32,90644719 | 22,4223394 | 10,4841078 | 12,58 | 0,291709 | -2,10 | 4,27 |
| 20ERC8h0.01R2 | 2 | 5 | 0.01 µM 20E | 10 | NA | 36,97824 | 3,119365 | 35 | 22,9271575 | 12,0728425 | 12,58 | 0,291709 | -0,51 | 1,42 |
| 20ERC8h0.01R3 | 3 | 5 | 0.01 µM 20E | 10 | NA | 35,82346 | 0 | 35 | 22,95352034 | 12,0464797 | 12,58 | 0,291709 | -0,53 | 1,45 |
| 20ERC8h0.03R1 | 1 | 5 | 0.03 µM 20E | 10 | NA | 30,36637 | 0,602861 | 30,36636781 | 22,03030862 | 8,3360592 | 12,58 | 0,291709 | -4,24 | 18,95 |
| 20ERC8h0.03R2 | 2 | 5 | 0.03 µM 20E | 10 | NA | 32,51214 | 0,500972 | 32,51214284 | 22,17130239 | 10,3408404 | 12,58 | 0,291709 | -2,24 | 4,72 |
| 20ERC8h0.03R3 | 3 | 5 | 0.03 µM 20E | 10 | NA | 33,78451 | 0,946103 | 33,78451015 | 23,1309832 | 10,653527 | 12,58 | 0,291709 | -1,93 | 3,80 |
| 20ERC8h0.1R1 | 1 | 5 | 0.1 µM 20E | 10 | NA | 29,3666 | 0,163949 | 29,36660069 | 22,26714499 | 7,0994557 | 12,58 | 0,291709 | -5,48 | 44,65 |
| 20ERC8h0.1R2 | 2 | 5 | 0.1 µM 20E | 10 | NA | 29,26703 | 0,152617 | 29,26703304 | 23,16932247 | 6,09771058 | 12,58 | 0,291709 | -6,48 | 89,41 |
| 20ERC8h0.1R3 | 3 | 5 | 0.1 µM 20E | 10 | NA | 30,86798 | 0,225141 | 30,86798484 | 23,08362347 | 7,78436137 | 12,58 | 0,291709 | -4,80 | 27,77 |
| 20ERC8h0.3R1 | 1 | 5 | 0.3 µM 20E | 10 | NA | 28,02799 | 0,676712 | 28,02799166 | 22,52706907 | 5,50092259 | 12,58 | 0,291709 | -7,08 | 135,21 |
| 20ERC8h0.3R2 | 2 | 5 | 0.3 µM 20E | 10 | NA | 28,43927 | 0,435538 | 28,43926735 | 22,46647162 | 5,97279574 | 12,58 | 0,291709 | -6,61 | 97,49 |
| 20ERC8h0.3R3 | 3 | 5 | 0.3 µM 20E | 10 | NA |  | 0 |  |  |  | 12,58 | 0,291709 |  |  |
| 20ERC8h1R1 | 1 | 5 | 1 µM 20E | 10 | NA | 27,07448 | 0,225489 | 27,07448242 | 21,96236616 | 5,11211625 | 12,58 | 0,291709 | -7,47 | 177,03 |
| 20ERC8h1R2 | 2 | 5 | 1 µM 20E | 10 | NA | 28,91261 | 0,146842 | 28,91261026 | 25,04940624 | 3,86320402 | 12,58 | 0,291709 | -8,72 | 420,74 |
| 20ERC8h1R3 | 3 | 5 | 1 µM 20E | 10 | NA | 29,28689 | 0,379397 | 29,28688845 | 25,69143208 | 3,59545637 | 12,58 | 0,291709 | -8,98 | 506,54 |
| 20ERC8h3R1 | 1 | 5 | 3 µM 20E | 10 | NA | 26,48633 | 0 | 26,48633438 | 23,88823867 | 2,59809571 | 12,58 | 0,291709 | -9,98 | 1011,24 |
| 20ERC8h3R2 | 2 | 5 | 3 µM 20E | 10 | NA | 26,58395 | 0,022653 | 26,58395193 | 22,99211511 | 3,59183682 | 12,58 | 0,291709 | -8,99 | 507,82 |
| 20ERC8h3R3 | 3 | 5 | 3 µM 20E | 10 | NA | 27,37758 | 0,00671 | 27,37758252 | 23,011798 | 4,36578452 | 12,58 | 0,291709 | -8,21 | 296,98 |
| 20ERR8h0.003R1 | 1 | 5 | 0.003 µM 20E | 2 | 8 |  | 0 | 35 | 22,11783064 | 12,8821694 | 12,58 | 0,291709 | 0,30 | 0,81 |
| 20ERR8h0.003R2 | 2 | 5 | 0.003 µM 20E | 2 | 8 | 34,25431 | 0,967925 | 34,2543059 | 21,82675467 | 12,4275512 | 12,58 | 0,291709 | -0,15 | 1,11 |
| 20ERR8h0.003R3 | 3 | 5 | 0.003 µM 20E | 2 | 8 |  | 0 | 35 | 22,42594108 | 12,5740589 | 12,58 | 0,291709 | -0,01 | 1,00 |
| 20ERR8h0.01R1 | 1 | 5 | 0.01 µM 20E | 2 | 8 | 34,89042 | 0 | 34,8904179 | 22,58267927 | 12,3077386 | 12,58 | 0,291709 | -0,27 | 1,21 |
| 20ERR8h0.01R2 | 2 | 5 | 0.01 µM 20E | 2 | 8 | 35,30482 | 0 | 35 | 22,19032537 | 12,8096746 | 12,58 | 0,291709 | 0,23 | 0,85 |
| 20ERR8h0.01R3 | 3 | 5 | 0.01 µM 20E | 2 | 8 |  | 0 |  | 25,02210576 | -25,022106 | 12,58 | 0,291709 |  |  |
| 20ERR8h0.03R1 | 1 | 5 | 0.03 µM 20E | 2 | 8 | 35,04835 | 0,372897 | 35 | 22,2753294 | 12,7246706 | 12,58 | 0,291709 | 0,14 | 0,90 |
| 20ERR8h0.03R2 | 2 | 5 | 0.03 µM 20E | 2 | 8 | 34,53295 | 1,256126 | 34,53295104 | 21,48217379 | 13,0507772 | 12,58 | 0,291709 | 0,47 | 0,72 |
| 20ERR8h0.03R3 | 3 | 5 | 0.03 µM 20E | 2 | 8 | 35,43142 | 0,237058 | 35 | 22,1588627 | 12,8411373 | 12,58 | 0,291709 | 0,26 | 0,83 |
| 20ERR8h0.1R1 | 1 | 5 | 0.1 µM 20E | 2 | 8 | 33,77285 | 0,402697 | 33,77285136 | 21,93443759 | 11,8384138 | 12,58 | 0,291709 | -0,74 | 1,67 |
| 20ERR8h0.1R2 | 2 | 5 | 0.1 µM 20E | 2 | 8 | 34,76025 | 0,124438 | 34,76024595 | 22,34611041 | 12,4141355 | 12,58 | 0,291709 | -0,17 | 1,12 |
| 20ERR8h0.1R3 | 3 | 5 | 0.1 µM 20E | 2 | 8 | 33,67819 | 1,011103 | 33,67818793 | 22,40353478 | 11,2746532 | 12,58 | 0,291709 | -1,31 | 2,47 |
| 20ERR8h0.3R1 | 1 | 5 | 0.3 µM 20E | 2 | 8 | 33,31929 | 0,273518 | 33,31929485 | 21,92529753 | 11,3939973 | 12,58 | 0,291709 | -1,19 | 2,28 |
| 20ERR8h0.3R2 | 2 | 5 | 0.3 µM 20E | 2 | 8 | 32,64571 | 0,213785 | 32,64571431 | 21,62678836 | 11,0189259 | 12,58 | 0,291709 | -1,56 | 2,95 |
| 20ERR8h0.3R3 | 3 | 5 | 0.3 µM 20E | 2 | 8 |  | 0 |  | 23,92318019 | -23,92318 | 12,58 | 0,291709 |  |  |
| 20ERR8h1R1 | 1 | 5 | 1 µM 20E | 2 | 8 | 31,48656 | 0,111835 | 31,48656385 | 21,349752 | 10,1368119 | 12,58 | 0,291709 | -2,44 | 5,44 |
| 20ERR8h1R2 | 2 | 5 | 1 µM 20E | 2 | 8 | 32,70387 | 0,343282 | 32,70386836 | 21,63102273 | 11,0728456 | 12,58 | 0,291709 | -1,51 | 2,84 |
| 20ERR8h1R3 | 3 | 5 | 1 µM 20E | 2 | 8 | 32,25263 | 1,285272 | 32,25263478 | 22,29880097 | 9,95383382 | 12,58 | 0,291709 | -2,63 | 6,17 |
| 20ERR8h3R1 | 1 | 5 | 3 µM 20E | 2 | 8 | 34,23137 | 0,015704 | 34,23136597 | 20,99323836 | 13,2381276 | 12,58 | 0,291709 | 0,66 | 0,63 |
| 20ERR8h3R2 | 2 | 5 | 3 µM 20E | 2 | 8 | 30,9326 | 0,390402 | 30,93259997 | 21,79499846 | 9,13760151 | 12,58 | 0,291709 | -3,44 | 10,87 |
| 20ERR8h3R3 | 3 | 5 | 3 µM 20E | 2 | 8 | 31,59003 | 0,66362 | 31,59002578 | 22,24283199 | 9,34719379 | 12,58 | 0,291709 | -3,23 | 9,40 |
| SC8hR1 | 1 | 5 | 0.5% DMSO | 10 | NA | 39,84559 | 0 | 35 | 22,01086538 | 12,9891346 | 12,58 | 0,291709 | -1,57 | 2,98 |
| SC8hR2 | 2 | 5 | 0.5% DMSO | 10 | NA | 34,32559 | 0 | 34,3255946 | 21,90436916 | 12,4212254 | 12,58 | 0,291709 | -0,53 | 1,44 |
| SC8hR3 | 3 | 5 | 0.5% DMSO | 10 | NA |  | 0 | 35 | 22,67036347 | 12,3296365 | 12,58 | 0,291709 | -0,87 | 1,82 |
| PR2h0.003R1 | 1 | 4 | 0.003 µM 20E | 2 | NA | 34,08622 | 0 | 34,08621817 | 24,06650089 | 10,0197173 | 11,59 | 0,063042 | -1,30 | 2,47 |
| PR2h0.003R2 | 2 | 4 | 0.003 µM 20E | 2 | NA | 34,86362 | 0,29745 | 34,86362269 | 23,79508142 | 11,0685413 | 11,59 | 0,063042 | -0,98 | 1,97 |
| PR2h0.003R3 | 3 | 4 | 0.003 µM 20E | 2 | NA | 35,43409 | 0 | 35 | 24,27232302 | 10,727677 | 11,59 | 0,063042 | -1,34 | 2,53 |
| PR2h0.01R1 | 1 | 4 | 0.01 µM 20E | 2 | NA | 34,35096 | 0 | 34,35095943 | 24,05947527 | 10,2914842 | 11,59 | 0,063042 | -2,71 | 6,52 |
| PR2h0.01R2 | 2 | 4 | 0.01 µM 20E | 2 | NA | 34,37682 | 1,383328 | 34,37681858 | 23,76239218 | 10,6144264 | 11,59 | 0,063042 | -3,23 | 9,36 |
| PR2h0.01R3 | 3 | 4 | 0.01 µM 20E | 2 | NA | 34,17139 | 1,557371 | 34,17139085 | 23,91807338 | 10,2533175 | 11,59 | 0,063042 | -3,17 | 8,98 |
| PR2h0.03R1 | 1 | 4 | 0.03 µM 20E | 2 | NA | 32,72606 | 0,79354 | 32,72605625 | 23,83681342 | 8,88924283 | 11,59 | 0,063042 | -6,93 | 121,66 |
| PR2h0.03R2 | 2 | 4 | 0.03 µM 20E | 2 | NA | 32,35639 | 0,032894 | 32,35639101 | 23,98816497 | 8,36822605 | 11,59 | 0,063042 | -5,78 | 54,95 |
| PR2h0.03R3 | 3 | 4 | 0.03 µM 20E | 2 | NA | 32,42461 | 0,586312 | 32,42460801 | 23,99708172 | 8,42752628 | 11,59 | 0,063042 | -5,63 | 49,44 |
| PR2h0.1R1 | 1 | 4 | 0.1 µM 20E | 2 | NA | 28,53082 | 0,030856 | 28,53082483 | 23,86297226 | 4,66785256 | 11,59 | 0,063042 | -6,16 | 71,52 |
| PR2h0.1R2 | 2 | 4 | 0.1 µM 20E | 2 | NA | 29,82461 | 0,158233 | 29,82461335 | 24,01000863 | 5,81460472 | 11,59 | 0,063042 | -6,44 | 86,61 |
| PR2h0.1R3 | 3 | 4 | 0.1 µM 20E | 2 | NA | 29,72503 | 0,329264 | 29,7250252 | 23,75794196 | 5,96708324 | 11,59 | 0,063042 | -6,70 | 104,02 |
| PR2h0.3R1 | 1 | 4 | 0.3 µM 20E | 2 | NA | 28,62509 | 0,392171 | 28,62509355 | 23,19081411 | 5,43427944 | 11,59 | 0,063042 | -6,74 | 106,53 |
| PR2h0.3R2 | 2 | 4 | 0.3 µM 20E | 2 | NA | 29,28197 | 0,026487 | 29,28196844 | 24,12390629 | 5,15806215 | 11,59 | 0,063042 | -6,02 | 65,08 |
| PR2h0.3R3 | 3 | 4 | 0.3 µM 20E | 2 | NA | 28,81295 | 0,066965 | 28,81295354 | 23,91917863 | 4,89377491 | 11,59 | 0,063042 | -6,87 | 116,92 |
| PR2h1R1 | 1 | 4 | 1 µM 20E | 2 | NA | 29,12237 | 0,2914 | 29,122373 | 24,26299429 | 4,8593787 | 11,59 | 0,063042 | -6,72 | 105,73 |
| PR2h1R2 | 2 | 4 | 1 µM 20E | 2 | NA | 30,07182 | 1,018601 | 30,07181969 | 24,5015076 | 5,57031208 | 11,59 | 0,063042 | -4,91 | 30,04 |
| PR2h1R3 | 3 | 4 | 1 µM 20E | 2 | NA | 29,06186 | 0,26517 | 29,06185543 | 24,33668238 | 4,72517305 | 11,59 | 0,063042 | -7,40 | 168,40 |
| PR2h3R1 | 1 | 4 | 3 µM 20E | 2 | NA | 29,87238 | 0,922489 | 29,87237756 | 25,00208343 | 4,87029412 | 11,59 | 0,063042 | -1,29 | 2,45 |
| PR2h3R2 | 2 | 4 | 3 µM 20E | 2 | NA | 31,16615 | 0,506332 | 31,16614935 | 24,48061948 | 6,68552987 | 11,59 | 0,063042 | -0,07 | 1,05 |
| PR2h3R3 | 3 | 4 | 3 µM 20E | 2 | NA | 29,25716 | 0,276841 | 29,25715833 | 25,05831165 | 4,19884668 | 11,59 | 0,063042 | 0,01 | 0,99 |
| PR2hCSR1 | 1 | 4 | 0.5% DMSO | 2 | NA | 34,75998 | 0,427583 | 34,75997547 | 23,17631189 | 11,5836636 | 11,59 | 0,063042 |  |  |
| PR2hCSR2 | 2 | 4 | 0.5% DMSO | 2 | NA | 35,20547 | 0,194911 | 35 | 23,47662596 | 11,523374 | 11,59 | 0,063042 |  |  |
| PR2hCSR3 | 3 | 4 | 0.5% DMSO | 2 | NA | 35,2466 | 0 | 35 | 23,32336307 | 11,6766369 | 11,59 | 0,063042 |  |  |
| RC8h0.003R1 | 1 | 4 | 0.003 µM 20E | 10 | NA | 36,31459 | 0 | 35 | 25,33356426 | 9,66643574 | 10,96 | 0,559816 | -1,29 | 2,45 |
| RC8h0.003R2 | 2 | 4 | 0.003 µM 20E | 10 | NA |  | 0 | 35 | 24,1117651 | 10,8882349 | 10,96 | 0,559816 | -0,07 | 1,05 |
| RC8h0.003R3 | 3 | 4 | 0.003 µM 20E | 10 | NA |  | 0 | 35 | 24,03244502 | 10,967555 | 10,96 | 0,559816 | 0,01 | 0,99 |
| RC8h0.01R1 | 1 | 4 | 0.01 µM 20E | 10 | NA | 34,3467 | 0 | 34,34670471 | 24,72360096 | 9,62310375 | 10,96 | 0,559816 | -1,33 | 2,52 |
| RC8h0.01R2 | 2 | 4 | 0.01 µM 20E | 10 | NA | 34,01547 | 0 | 34,01546923 | 24,09889467 | 9,91657456 | 10,96 | 0,559816 | -1,04 | 2,06 |
| RC8h0.01R3 | 3 | 4 | 0.01 µM 20E | 10 | NA | 35,74033 | 0 | 35 | 23,83204493 | 11,1679551 | 10,96 | 0,559816 | 0,21 | 0,86 |
| RC8h0.03R1 | 1 | 4 | 0.03 µM 20E | 10 | NA | 33,27633 | 0 | 33,27632758 | 24,08934449 | 9,1869831 | 10,96 | 0,559816 | -1,77 | 3,41 |
| RC8h0.03R2 | 2 | 4 | 0.03 µM 20E | 10 | NA | 33,38262 | 1,146908 | 33,38261511 | 23,0629046 | 10,3197105 | 10,96 | 0,559816 | -0,64 | 1,55 |
| RC8h0.03R3 | 3 | 4 | 0.03 µM 20E | 10 | NA | 33,51092 | 0,215652 | 33,51091696 | 23,9270167 | 9,58390026 | 10,96 | 0,559816 | -1,37 | 2,59 |
| RC8h0.1R1 | 1 | 4 | 0.1 µM 20E | 10 | NA | 32,05705 | 0,124886 | 32,05705177 | 25,60508325 | 6,45196851 | 10,96 | 0,559816 | -4,50 | 22,70 |
| RC8h0.1R2 | 2 | 4 | 0.1 µM 20E | 10 | NA | 31,38091 | 0,408141 | 31,380913 | 24,75062075 | 6,63029226 | 10,96 | 0,559816 | -4,33 | 20,06 |
| RC8h0.1R3 | 3 | 4 | 0.1 µM 20E | 10 | NA | 32,00507 | 0,297457 | 32,00507342 | 24,38492629 | 7,62014713 | 10,96 | 0,559816 | -3,34 | 10,10 |
| RC8h0.3R1 | 1 | 4 | 0.3 µM 20E | 10 | NA | 29,92225 | 0,951487 | 29,92224549 | 25,03779239 | 4,88445309 | 10,96 | 0,559816 | -6,07 | 67,27 |
| RC8h0.3R2 | 2 | 4 | 0.3 µM 20E | 10 | NA | 29,7331 | 0,479535 | 29,73310258 | 24,99835833 | 4,73474425 | 10,96 | 0,559816 | -6,22 | 74,63 |
| RC8h0.3R3 | 3 | 4 | 0.3 µM 20E | 10 | NA | 30,12647 | 0,157619 | 30,12646506 | 25,15612733 | 4,97033773 | 10,96 | 0,559816 | -5,99 | 63,38 |
| RC8h1R1 | 1 | 4 | 1 µM 20E | 10 | NA | 28,2997 | 0,119233 | 28,29969661 | 25,66166746 | 2,63802916 | 10,96 | 0,559816 | -8,32 | 319,21 |
| RC8h1R2 | 2 | 4 | 1 µM 20E | 10 | NA | 28,30039 | 0,040724 | 28,30039126 | 25,14136453 | 3,15902673 | 10,96 | 0,559816 | -7,80 | 222,45 |
| RC8h1R3 | 3 | 4 | 1 µM 20E | 10 | NA | 28,45753 | 0,362633 | 28,45753054 | 25,4551543 | 3,00237625 | 10,96 | 0,559816 | -7,95 | 247,97 |
| RC8h3R1 | 1 | 4 | 3 µM 20E | 10 | NA | 29,13632 | 0,039378 | 29,13632303 | 26,04497923 | 3,0913438 | 10,96 | 0,559816 | -7,87 | 233,14 |
| RC8h3R2 | 2 | 4 | 3 µM 20E | 10 | NA | 28,97719 | 0,410029 | 28,97718587 | 25,97859231 | 2,99859356 | 10,96 | 0,559816 | -7,96 | 248,62 |
| RC8h3R3 | 3 | 4 | 3 µM 20E | 10 | NA | 28,74042 | 0,432573 | 28,74041801 | 25,64677097 | 3,09364703 | 10,96 | 0,559816 | -7,86 | 232,77 |
| RC8hCSR1 | 1 | 4 | 0.5% DMSO | 10 | NA | 37,8404 | 0 | 35 | 24,55039242 | 10,4496076 | 10,96 | 0,559816 |  |  |
| RC8hCSR2 | 2 | 4 | 0.5% DMSO | 10 | NA |  | 0 | 35 | 24,86203113 | 10,1379689 | 10,96 | 0,559816 |  |  |
| RC8hCSR3 | 3 | 4 | 0.5% DMSO | 10 | NA |  | 0 | 35 | 24,16190411 | 10,8380959 | 10,96 | 0,559816 |  |  |
| RR8h0.003R1 | 1 | 4 | 0.003 µM 20E | 2 | 8 | 35,50763 | 0,436816 | 35 | 24,03374166 | 10,9662583 | 10,96 | 0,559816 | 0,01 | 0,99 |
| RR8h0.003R2 | 2 | 4 | 0.003 µM 20E | 2 | 8 | 33,65018 | 0 | 33,6501837 | 24,47529136 | 9,17489235 | 10,96 | 0,559816 | -1,78 | 3,44 |
| RR8h0.003R3 | 3 | 4 | 0.003 µM 20E | 2 | 8 | 35,25432 | 0 | 35 | 24,18965883 | 10,8103412 | 10,96 | 0,559816 | -0,15 | 1,11 |
| RR8h0.01R1 | 1 | 4 | 0.01 µM 20E | 2 | 8 |  | 0 | 35 | 24,2969812 | 10,7030188 | 10,96 | 0,559816 | -0,25 | 1,19 |
| RR8h0.01R2 | 2 | 4 | 0.01 µM 20E | 2 | 8 |  | 0 | 35 | 24,3096332 | 10,6903668 | 10,96 | 0,559816 | -0,27 | 1,20 |
| RR8h0.01R3 | 3 | 4 | 0.01 µM 20E | 2 | 8 | 34,2486 | 0 | 34,24859831 | 23,74899753 | 10,4996008 | 10,96 | 0,559816 | -0,46 | 1,37 |
| RR8h0.03R2 | 2 | 4 | 0.03 µM 20E | 2 | 8 | 34,88221 | 0,619158 | 34,88220554 | 24,76190518 | 10,1203004 | 10,96 | 0,559816 | -0,84 | 1,79 |
| RR8h0.03R3 | 3 | 4 | 0.03 µM 20E | 2 | 8 |  | 0 | 35 | 23,63004873 | 11,3699513 | 10,96 | 0,559816 | 0,41 | 0,75 |
| RR8h0.1R1 | 1 | 4 | 0.1 µM 20E | 2 | 8 | 34,30074 | 0,062648 | 34,30074072 | 23,91598832 | 10,3847524 | 10,96 | 0,559816 | -0,57 | 1,49 |
| RR8h0.1R2 | 2 | 4 | 0.1 µM 20E | 2 | 8 | 34,80467 | 0,768679 | 34,8046685 | 23,83015246 | 10,974516 | 10,96 | 0,559816 | 0,02 | 0,99 |
| RR8h0.3R1 | 1 | 4 | 0.3 µM 20E | 2 | 8 | 35,21594 | 0,107458 | 35 | 24,69663993 | 10,3033601 | 10,96 | 0,559816 | -0,65 | 1,57 |
| RR8h0.3R2 | 2 | 4 | 0.3 µM 20E | 2 | 8 | 34,00262 | 0,461829 | 34,00261512 | 23,93516496 | 10,0674502 | 10,96 | 0,559816 | -0,89 | 1,85 |
| RR8h1R1 | 1 | 4 | 1 µM 20E | 2 | 8 | 33,42578 | 0,445351 | 33,42578292 | 24,5723365 | 8,85344642 | 10,96 | 0,559816 | -2,10 | 4,30 |
| RR8h1R2 | 2 | 4 | 1 µM 20E | 2 | 8 | 35,37701 | 0 | 35 | 23,92249298 | 11,077507 | 10,96 | 0,559816 | 0,12 | 0,92 |
| RR8h1R3 | 3 | 4 | 1 µM 20E | 2 | 8 | 35,06311 | 0,480812 | 35 | 24,3181916 | 10,6818084 | 10,96 | 0,559816 | -0,27 | 1,21 |
| RR8h3R1 | 1 | 4 | 3 µM 20E | 2 | 8 | 33,7274 | 0,732656 | 33,72739591 | 25,5368918 | 8,19050411 | 10,96 | 0,559816 | -2,77 | 6,80 |
| RR8h3R2 | 2 | 4 | 3 µM 20E | 2 | 8 | 34,75814 | 1,174774 | 34,75814123 | 24,38143984 | 10,3767014 | 10,96 | 0,559816 | -0,58 | 1,49 |
| RR8hCSR1 | 1 | 4 | 0.5% DMSO | 10 | NA |  | 0 | 35 | 23,6639561 | 11,3360439 | 10,96 | 0,559816 |  |  |
| RR8hCSR2 | 2 | 4 | 0.5% DMSO | 10 | NA |  | 0 | 35 | 23,17278357 | 11,8272164 | 10,96 | 0,559816 |  |  |
| RR8hCSR3 | 3 | 4 | 0.5% DMSO | 10 | NA | 35,18823 | 0 | 35 | 23,85060323 | 11,1493968 | 10,96 | 0,559816 |  |  |
| 20ERC8h0.003R1 | 1 | 6 | 0.003 µM 20E | 10 | NA |  | 0 | 35 | 24,6740255 | 10,3259745 | 12,32 | 0,0424248 | -1,9959 | 3,9886599 |
| 20ERC8h0.003R2 | 2 | 6 | 0.003 µM 20E | 10 | NA | 37,00803 | 0 | 35 | 25,05101999 | 9,94898001 | 12,32 | 0,0424248 | -2,3729 | 5,179808 |
| 20ERC8h0.01R1 | 1 | 6 | 0.01 µM 20E | 10 | NA | 35,45137 | 0 | 35 | 24,36954266 | 10,6304573 | 12,32 | 0,0424248 | -1,69142 | 3,2297473 |
| 20ERC8h0.01R2 | 2 | 6 | 0.01 µM 20E | 10 | NA | 35,65326 | 0 | 35 | 23,18717505 | 11,8128249 | 12,32 | 0,0424248 | -0,50905 | 1,4231164 |
| 20ERC8h0.01R3 | 3 | 6 | 0.01 µM 20E | 10 | NA | 35,98877 | 0,570858 | 35 | 24,46429689 | 10,5357031 | 12,32 | 0,0424248 | -1,78618 | 3,4489937 |
| 20ERC8h0.03R1 | 1 | 6 | 0.03 µM 20E | 10 | NA | 32,80818 | 1,710991 | 32,80818351 | 23,34814476 | 9,46003875 | 12,32 | 0,0424248 | -2,86184 | 7,269418 |
| 20ERC8h0.03R3 | 3 | 6 | 0.03 µM 20E | 10 | NA | 36,22911 | 0 | 35 | 24,10747574 | 10,8925243 | 12,32 | 0,0424248 | -1,42935 | 2,6932616 |
| 20ERC8h0.1R2 | 2 | 6 | 0.1 µM 20E | 10 | NA | 30,7751 | 0,370101 | 30,77509721 | 23,0638348 | 7,71126241 | 12,32 | 0,0424248 | -4,61062 | 24,43058 |
| 20ERC8h0.1R3 | 3 | 6 | 0.1 µM 20E | 10 | NA | 30,42379 | 0,376178 | 30,42378744 | 24,10323852 | 6,32054892 | 12,32 | 0,0424248 | -6,00133 | 64,059015 |
| 20ERC8h0.3R1 | 1 | 6 | 0.3 µM 20E | 10 | NA | 32,04568 | 0,660517 | 32,04568457 | 24,44131599 | 7,60436858 | 12,32 | 0,0424248 | -4,71751 | 26,309466 |
| 20ERC8h0.3R2 | 2 | 6 | 0.3 µM 20E | 10 | NA | 30,34473 | 0,600232 | 30,34472941 | 22,91674661 | 7,42798281 | 12,32 | 0,0424248 | -4,8939 | 29,730995 |
| 20ERC8h0.3R3 | 3 | 6 | 0.3 µM 20E | 10 | NA | 30,29532 | 1,074316 | 30,29532337 | 23,73459404 |  | 12,32 | 0,0424248 | -5,76115 | 54,234888 |
| 20ERC8h1R1 | 1 | 6 | 1 µM 20E | 10 | NA | 28,16742 | 0,221164 | 28,16742411 | 23,66687898 | 4,50054513 | 12,32 | 0,0424248 | -7,82133 | 226,18093 |
| 20ERC8h1R2 | 2 | 6 | 1 µM 20E | 10 | NA | 31,71218 | 0,543196 | 31,71217667 | 24,9274558 | 6,78472087 | 12,32 | 0,0424248 | -5,53716 | 46,435548 |
| 20ERC8h1R3 | 3 | 6 | 1 µM 20E | 10 | NA | 28,17479 | 0,59366 | 28,1747913 | 23,61815877 | 4,55663253 | 12,32 | 0,0424248 | -7,76525 | 217,55647 |
| 20ERC8h3R1 | 1 | 6 | 3 µM 20E | 10 | NA | 28,87689 | 0,072249 | 28,87689386 | 23,39628549 | 5,48060837 | 12,32 | 0,0424248 | -6,84127 | 114,66412 |
| 20ERC8h3R2 | 2 | 6 | 3 µM 20E | 10 | NA | 29,61583 | 0,511443 | 29,6158293 | 24,85654185 | 4,75928745 | 12,32 | 0,0424248 | -7,56259 | 189,04569 |
| 20ERC8h3R3 | 3 | 6 | 3 µM 20E | 10 | NA | 28,2762 | 0,382491 | 28,27619552 | 24,26606522 | 4,0101303 | 12,32 | 0,0424248 | -8,31175 | 317,75 |
| 20ERR8h0.01R2 | 2 | 6 | 0.01 µM 20E | 2 | 8 |  | 0 | 35 | 22,07952547 | 12,9204745 | 12,32 | 0,0424248 | 0,598596 | 0,6603964 |
| 20ERR8h0.01R3 | 3 | 6 | 0.01 µM 20E | 2 | 8 | 33,48427 | 0 | 33,4842692 | 22,81823793 | 10,6660313 | 12,32 | 0,0424248 | -1,65585 | 3,1510821 |
| 20ERR8h0.03R3 | 3 | 6 | 0.03 µM 20E | 2 | 8 | 35,72898 | 0 | 35 | 24,94826011 | 10,0517399 | 12,32 | 0,0424248 | -2,27014 | 4,8236951 |
| 20ERR8h0.1R1 | 1 | 6 | 0.1 µM 20E | 2 | 8 | 35,16608 | 0 | 35 | 25,83004103 | 9,16995897 | 12,32 | 0,0424248 | -3,15192 | 8,8883748 |
| 20ERR8h0.1R2 | 2 | 6 | 0.1 µM 20E | 2 | 8 | 33,36178 | 0 | 33,36177902 | 22,46722676 | 10,8945523 | 12,32 | 0,0424248 | -1,42733 | 2,6894783 |
| 20ERR8h0.1R3 | 3 | 6 | 0.1 µM 20E | 2 | 8 | 34,06521 | 2,31498 | 34,0652138 | 23,08837286 | 10,9768409 | 12,32 | 0,0424248 | -1,34504 | 2,5403683 |
| 20ERR8h0.3R3 | 3 | 6 | 0.3 µM 20E | 2 | 8 | 33,60808 | 1,142975 | 33,6080842 | 22,69811513 | 10,9099691 | 12,32 | 0,0424248 | -1,41191 | 2,6608913 |
| 20ERR8h1R2 | 2 | 6 | 1 µM 20E | 2 | 8 | 32,79701 | 0,314299 | 32,79700836 | 22,32129579 | 10,4757126 | 12,32 | 0,0424248 | -1,84617 | 3,5954343 |
| 20ERR8h3R1 | 1 | 6 | 3 µM 20E | 2 | 8 | 33,58922 | 0 | 33,58921542 | 23,40487294 | 10,1843425 | 12,32 | 0,0424248 | -2,13754 | 4,4000995 |
| 20ERR8h3R3 | 3 | 6 | 3 µM 20E | 2 | 8 | 32,87802 | 0,070696 | 32,87801613 | 22,96665096 | 9,91136517 | 12,32 | 0,0424248 | -2,41051 | 5,3166351 |
| SC8hR2 | 2 | 6 | 0.5% DMSO | 10 | NA |  | 0 | 35 | 22,63569655 | 12,3643035 | 12,32 | 0,0424248 |  |  |
| SC8hR3 | 3 | 6 | 0.5% DMSO | 10 | NA | 35,13561 | 0 | 35,13560626 | 22,85615246 | 12,2794538 | 12,32 | 0,0424248 |  |  |

| sample id | replicate | experiment number | Treatment | exposure time/hours | recovery time/hours | Avg Cq | st.dev Cq | corrected Cq (max cq 35) | Refrence gene Cq | delta Cq | Avg. solvent control delta Cq | st.dev solvent control delta Cq | delta delta Cq | 2^ddCq |
| --- | --- | --- | --- | --- | --- | --- | --- | --- | --- | --- | --- | --- | --- | --- |
| PR1hA0.1R1 | 1 | 1 | 0.1 µM 20E | 1 | NA | 26,13 | 0,02089 | 29,49 | 24,11 | 5,38 | 5,36 | 0,28579415 | -0,14 | 1,10 |
| PR1hA0.1R2 | 2 | 1 | 0.1 µM 20E | 1 | NA | 28,26 | 0,00129 | 29,21 | 23,83 | 5,38 | 5,36 | 0,28579415 | -0,20 | 1,15 |
| PR1hA0.1R3 | 3 | 1 | 0.1 µM 20E | 1 | NA | 36,54 | 1,24381 | 30,17 | 25,01 | 5,15 | 5,36 | 0,28579415 | 0,09 | 0,94 |
| PR1hB0.1R1 | 4 | 1 | 0.1 µM 20E | 1 | NA | 29,57 | 0,14238 | 30,20 | 24,63 | 5,57 | 5,36 | 0,28579415 |  |  |
| PR1hB0.1R2 | 5 | 1 | 0.1 µM 20E | 1 | NA | 28,76 | 0,38295 | 29,88 | 24,38 | 5,50 | 5,36 | 0,28579415 |  |  |
| PR1hB0.1R3 | 6 | 1 | 0.1 µM 20E | 1 | NA | 36,57 | 0,16753 | 30,30 | 24,80 | 5,50 | 5,36 | 0,28579415 |  |  |
| PR1hCSR1 | 1 | 1 | 0.1 µM 20E | 1 | NA | 37,17 | 0,00000 | 30,53 | 25,19 | 5,34 | 5,36 | 0,28579415 |  |  |
| PR1hCSR2 | 2 | 1 | 0.1 µM 20E | 1 | NA | 35,34 | 1,63820 | 30,47 | 24,70 | 5,77 | 5,36 | 0,28579415 |  |  |
| PR1hCSR3 | 3 | 1 | 0.1 µM 20E | 1 | NA | 33,22 | 1,84902 | 30,53 | 25,38 | 5,15 | 5,36 | 0,28579415 |  |  |
| PR2hA0.1R1 | 1 | 1 | 0.1 µM 20E | 2 | NA | 26,03 | 0,09343 | 30,10 | 24,32 | 5,78 | 5,36 | 0,28579415 | 0,11 | 0,93 |
| PR2hA0.1R2 | 2 | 1 | 0.1 µM 20E | 2 | NA | 28,49 | 0,03464 | 31,44 | 26,35 | 5,09 | 5,36 | 0,28579415 | 0,07 | 0,95 |
| PR2hA0.1R3 | 3 | 1 | 0.1 µM 20E | 2 | NA | 29,40 | 0,30653 | 30,52 | 25,37 | 5,16 | 5,36 | 0,28579415 | -0,04 | 1,03 |
| PR2hB0.1R1 | 4 | 1 | 0.1 µM 20E | 2 | NA | 28,77 | 0,34167 | 29,98 | 24,39 | 5,59 | 5,36 | 0,28579415 |  |  |
| PR2hB0.1R2 | 5 | 1 | 0.1 µM 20E | 2 | NA | 28,28 | 0,31111 | 30,42 | 25,16 | 5,26 | 5,36 | 0,28579415 |  |  |
| PR2hB0.1R3 | 6 | 1 | 0.1 µM 20E | 2 | NA | 30,36 | 0,67995 | 30,82 | 25,10 | 5,72 | 5,36 | 0,28579415 |  |  |
| PR2hCSR1 | 1 | 1 | 0.5% DMSO | 2 | NA | 34,87 | 1,04142 | 30,90 | 25,36 | 5,54 | 5,36 | 0,28579415 |  |  |
| PR2hCSR2 | 2 | 1 | 0.5% DMSO | 2 | NA | 36,54 | 0,00000 | 29,98 | 24,75 | 5,22 | 5,36 | 0,28579415 |  |  |
| PR2hCSR3 | 3 | 1 | 0.5% DMSO | 2 | NA | 33,75 | 0,04970 | 30,57 | 25,37 | 5,20 | 5,36 | 0,28579415 |  |  |
| PR30minA0.1R1 | 1 | 1 | 0.1 µM 20E | 0,5 | NA | 26,71 | 0,26915 | 28,90 | 23,58 | 5,32 | 5,36 | 0,28579415 | 0,32 | 0,80 |
| PR30minA0.1R2 | 2 | 1 | 0.1 µM 20E | 0,5 | NA | 30,46 | 0,05462 | 29,50 | 24,13 | 5,37 | 5,36 | 0,28579415 | -0,19 | 1,14 |
| PR30minA0.1R3 | 3 | 1 | 0.1 µM 20E | 0,5 | NA | 36,15 | 0,00000 | 30,68 | 25,14 | 5,54 | 5,36 | 0,28579415 | 0,07 | 0,95 |
| PR30minB0.1R1 | 4 | 1 | 0.1 µM 20E | 0,5 | NA | 30,70 | 0,50500 | 29,54 | 24,40 | 5,14 | 5,36 | 0,28579415 |  |  |
| PR30minB0.1R2 | 5 | 1 | 0.1 µM 20E | 0,5 | NA | 30,88 | 0,16317 | 29,22 | 24,26 | 4,97 | 5,36 | 0,28579415 |  |  |
| PR30minB0.1R3 | 6 | 1 | 0.1 µM 20E | 0,5 | NA | 35,39 | 0,00000 | 29,86 | 24,62 | 5,24 | 5,36 | 0,28579415 |  |  |
| PR30minCSR1 | 1 | 1 | 0.5% DMSO | 0,5 | NA | 34,36 | 0,00000 | 30,01 | 24,73 | 5,29 | 5,36 | 0,28579415 |  |  |
| PR30minCSR2 | 2 | 1 | 0.5% DMSO | 0,5 | NA | 35,25 | 0,99541 | 30,27 | 25,01 | 5,26 | 5,36 | 0,28579415 |  |  |
| PR30minCSR3 | 3 | 1 | 0.5% DMSO | 0,5 | NA | 33,96 | 1,27104 | 30,92 | 24,83 | 6,10 | 5,36 | 0,28579415 |  |  |
| R16hCSR1 | 1 | 1 | 0.5% DMSO | 18 | NA | 34,34 | 0,00000 | 30,11 | 24,63 | 5,49 | 5,36 | 0,28579415 |  |  |
| R16hCSR2 | 2 | 1 | 0.5% DMSO | 18 | NA | 35,34 | 0,00000 | 31,01 | 25,48 | 5,53 | 5,36 | 0,28579415 |  |  |
| R16hCSR3 | 3 | 1 | 0.5% DMSO | 18 | NA | 32,14 | 0,09990 | 31,04 | 25,46 | 5,59 | 5,36 | 0,28579415 |  |  |
| R1hCSR1 | 1 | 1 | 0.5% DMSO | 3 | NA | 33,33 | 1,23050 | 29,34 | 24,66 | 4,68 | 5,36 | 0,28579415 |  |  |
| R1hCSR2 | 2 | 1 | 0.5% DMSO | 3 | NA | 35,51 | 0,00000 | 29,91 | 24,51 | 5,39 | 5,36 | 0,28579415 |  |  |
| R1hCSR3 | 3 | 1 | 0.5% DMSO | 3 | NA | 33,35 | 0,45068 | 31,15 | 25,77 | 5,38 | 5,36 | 0,28579415 |  |  |
| R2hCSR1 | 1 | 1 | 0.5% DMSO | 4 | NA | 33,85 | 0,24336 | 29,19 | 24,10 | 5,09 | 5,36 | 0,28579415 |  |  |
| R2hCSR2 | 2 | 1 | 0.5% DMSO | 4 | NA | 35,98 | 0,66578 | 28,96 | 23,91 | 5,04 | 5,36 | 0,28579415 |  |  |
| R2hCSR3 | 3 | 1 | 0.5% DMSO | 4 | NA | 33,08 | 1,03187 | 30,11 | 24,75 | 5,36 | 5,36 | 0,28579415 |  |  |
| R4hCSR1 | 1 | 1 | 0.5% DMSO | 6 | NA | 32,96 | 0,13681 | 29,09 | 24,03 | 5,06 | 5,36 | 0,28579415 |  |  |
| R4hCSR2 | 2 | 1 | 0.5% DMSO | 6 | NA | 36,70 | 0,17962 | 29,55 | 24,08 | 5,47 | 5,36 | 0,28579415 |  |  |
| R4hCSR3 | 3 | 1 | 0.5% DMSO | 6 | NA | 30,93 | 0,80456 | 30,33 | 24,72 | 5,62 | 5,36 | 0,28579415 |  |  |
| R8hCSR1 | 1 | 1 | 0.5% DMSO | 10 | NA | 34,46 | 0,81036 | 28,65 | 23,57 | 5,08 | 5,36 | 0,28579415 |  |  |
| R8hCSR2 | 2 | 1 | 0.5% DMSO | 10 | NA | 35,35 | 0,00000 | 29,27 | 23,90 | 5,37 | 5,36 | 0,28579415 |  |  |
| R8hCSR3 | 3 | 1 | 0.5% DMSO | 10 | NA | 35,12 | 2,98357 | 30,61 | 24,87 | 5,73 | 5,36 | 0,28579415 |  |  |
| RC16h0.1R1 | 1 | 1 | 0.1 µM 20E | 18 | NA | 29,08 | 0,14463 | 32,40 | 25,93 | 6,47 | 5,36 | 0,28579415 | 1,10 | 0,47 |
| RC16h0.1R2 | 2 | 1 | 0.1 µM 20E | 18 | NA | 30,76 | 0,39342 | 32,99 | 26,64 | 6,36 | 5,36 | 0,28579415 | 0,99 | 0,50 |
| RC16h0.1R3 | 3 | 1 | 0.1 µM 20E | 18 | NA | 28,04 | 0,03846 | 32,23 | 25,43 | 6,80 | 5,36 | 0,28579415 | 1,44 | 0,37 |
| RC1h0.1R1 | 1 | 1 | 0.1 µM 20E | 3 | NA | 29,25 | 0,09986 | 29,89 | 23,76 | 6,13 | 5,36 | 0,28579415 | 0,77 | 0,59 |
| RC1h0.1R2 | 2 | 1 | 0.1 µM 20E | 3 | NA | 30,06 | 0,33561 | 30,76 | 24,52 | 6,24 | 5,36 | 0,28579415 | 0,87 | 0,55 |
| RC1h0.1R3 | 3 | 1 | 0.1 µM 20E | 3 | NA | 31,64 | 0,66578 | 31,46 | 24,81 | 6,65 | 5,36 | 0,28579415 | 1,28 | 0,41 |
| RC2h0.1R1 | 1 | 1 | 0.1 µM 20E | 4 | NA | 28,92 | 0,60873 | 29,93 | 23,69 | 6,24 | 5,36 | 0,28579415 | 0,87 | 0,55 |
| RC2h0.1R2 | 2 | 1 | 0.1 µM 20E | 4 | NA | 29,12 | 0,06975 | 31,77 | 25,19 | 6,58 | 5,36 | 0,28579415 | 1,22 | 0,43 |
| RC2h0.1R3 | 3 | 1 | 0.1 µM 20E | 4 | NA | 28,84 | 0,65579 | 30,83 | 24,45 | 6,38 | 5,36 | 0,28579415 | 1,02 | 0,49 |
| RC4h0.1R1 | 1 | 1 | 0.1 µM 20E | 6 | NA | 28,53 | 0,64253 | 29,78 | 23,47 | 6,31 | 5,36 | 0,28579415 | 0,95 | 0,52 |
| RC4h0.1R2 | 2 | 1 | 0.1 µM 20E | 6 | NA | 29,73 | 0,00367 | 32,04 | 25,13 | 6,91 | 5,36 | 0,28579415 | 1,54 | 0,34 |
| RC4h0.1R3 | 3 | 1 | 0.1 µM 20E | 6 | NA | 30,99 | 0,65219 | 32,04 | 25,20 | 6,83 | 5,36 | 0,28579415 | 1,47 | 0,36 |
| RC8h0.1R1 | 1 | 1 | 0.1 µM 20E | 10 | NA | 29,12 | 0,49696 | 29,78 | 23,58 | 6,19 | 5,36 | 0,28579415 | 0,83 | 0,56 |
| RC8h0.1R2 | 2 | 1 | 0.1 µM 20E | 10 | NA | 27,61 | 0,01646 | 29,64 | 23,50 | 6,14 | 5,36 | 0,28579415 | 0,78 | 0,58 |
| RC8h0.1R3 | 3 | 1 | 0.1 µM 20E | 10 | NA | 31,02 | 0,91758 | 31,52 | 24,51 | 7,01 | 5,36 | 0,28579415 | 1,65 | 0,32 |
| RR16h0.1R1 | 1 | 1 | 0.1 µM 20E | 2 | 16 | 34,70 | 0,28067 | 29,38 | 24,55 | 4,83 | 5,36 | 0,28579415 | -0,54 | 1,45 |
| RR16h0.1R2 | 2 | 1 | 0.1 µM 20E | 2 | 16 | 36,68 | 1,40401 | 30,54 | 25,17 | 5,37 | 5,36 | 0,28579415 | 0,01 | 1,00 |
| RR16h0.1R3 | 3 | 1 | 0.1 µM 20E | 2 | 16 | 33,38 | 0,15175 | 30,15 | 25,06 | 5,08 | 5,36 | 0,28579415 | -0,28 | 1,21 |
| RR1h0.1R1 | 1 | 1 | 0.1 µM 20E | 2 | 1 | 29,93 | 0,14108 | 30,96 | 24,46 | 6,50 | 5,36 | 0,28579415 | 1,13 | 0,46 |
| RR1h0.1R2 | 2 | 1 | 0.1 µM 20E | 2 | 1 | 31,39 | 0,02609 | 30,61 | 24,85 | 5,75 | 5,36 | 0,28579415 | 0,39 | 0,76 |
| RR1h0.1R3 | 3 | 1 | 0.1 µM 20E | 2 | 1 | 33,06 | 0,00561 | 30,68 | 24,56 | 6,11 | 5,36 | 0,28579415 | 0,75 | 0,60 |
| RR2h0.1R1 | 1 | 1 | 0.1 µM 20E | 2 | 2 | 29,19 | 0,22069 | 29,50 | 23,37 | 6,14 | 5,36 | 0,28579415 | 0,77 | 0,59 |
| RR2h0.1R2 | 2 | 1 | 0.1 µM 20E | 2 | 2 | 32,43 | 0,06168 | 31,65 | 25,07 | 6,58 | 5,36 | 0,28579415 | 1,21 | 0,43 |
| RR2h0.1R3 | 3 | 1 | 0.1 µM 20E | 2 | 2 | 32,55 | 0,25455 | 30,50 | 24,45 | 6,05 | 5,36 | 0,28579415 | 0,69 | 0,62 |
| RR4h0.1R1 | 1 | 1 | 0.1 µM 20E | 2 | 4 | 30,83 | 0,52069 | 30,28 | 24,66 | 5,62 | 5,36 | 0,28579415 | 0,25 | 0,84 |
| RR4h0.1R2 | 2 | 1 | 0.1 µM 20E | 2 | 4 | 34,34 | 0,54199 | 30,10 | 24,39 | 5,71 | 5,36 | 0,28579415 | 0,35 | 0,79 |
| RR4h0.1R3 | 3 | 1 | 0.1 µM 20E | 2 | 4 | 34,31 | 1,14148 | 29,65 | 24,13 | 5,52 | 5,36 | 0,28579415 | 0,15 | 0,90 |
| RR8h0.1R1 | 1 | 1 | 0.1 µM 20E | 2 | 8 | 30,80 | 0,96806 | 29,31 | 25,97 | 3,35 | 5,36 | 0,28579415 | -2,02 | 4,05 |
| RR8h0.1R2 | 2 | 1 | 0.1 µM 20E | 2 | 8 | 33,88 | 0,36599 | 28,42 | 23,66 | 4,76 | 5,36 | 0,28579415 | -0,60 | 1,52 |
| RR8h0.1R3 | 3 | 1 | 0.1 µM 20E | 2 | 8 | 35,46 | 0,00000 | 29,33 | 24,17 | 5,16 | 5,36 | 0,28579415 | -0,20 | 1,15 |
| PR1h0.1R1 | 1 | 2 | 0.1 µM 20E | 1 | NA | 28,25336 | 0,076312 | 28,25 | 22,27 | 5,98 | 6,52 | 0,68649034 | -0,53 | 1,45 |
| PR1h0.1R2 | 2 | 2 | 0.1 µM 20E | 1 | NA | 27,28657 | 1,164076 | 27,29 | 22,09 | 5,20 | 6,52 | 0,68649034 | -1,32 | 2,49 |
| PR1hCSR1 | 1 | 2 | 0.1 µM 20E | 1 | NA | 28,76903 | 0,151103 | 28,77 | 22,01 | 6,75 | 6,52 | 0,68649034 |  |  |
| PR1hCSR2 | 2 | 2 | 0.1 µM 20E | 1 | NA | 28,89383 | 0,198941 | 28,89 | 22,33 | 6,56 | 6,52 | 0,68649034 |  |  |
| PR2h0.1R1 | 1 | 2 | 0.1 µM 20E | 2 | NA | 27,90765 | 0,356582 | 27,91 | 21,82 | 6,09 | 6,52 | 0,68649034 | -0,43 | 1,34 |
| PR2h0.1R2 | 2 | 2 | 0.1 µM 20E | 2 | NA | 28,68991 | 0,139264 | 28,69 | 21,66 | 7,03 | 6,52 | 0,68649034 | 0,52 | 0,70 |
| PR2hCSR1 | 1 | 2 | 0.5% DMSO | 2 | NA | 28,5251 | 0,396406 | 28,53 | 22,09 | 6,43 | 6,52 | 0,68649034 |  |  |
| PR2hCSR2 | 2 | 2 | 0.5% DMSO | 2 | NA | 28,64478 | 0,158461 | 28,64 | 22,06 | 6,59 | 6,52 | 0,68649034 |  |  |
| PR30min0.1R1 | 1 | 2 | 0.1 µM 20E | 0,5 | NA | 28,23563 | 0,097947 | 28,24 | 23,06 | 5,18 | 6,52 | 0,68649034 | -1,34 | 2,53 |
| PR30min0.1R2 | 2 | 2 | 0.1 µM 20E | 0,5 | NA | 27,89616 | 0,776471 | 27,90 | 22,21 | 5,69 | 6,52 | 0,68649034 | -0,83 | 1,78 |
| PR30minCSR1 | 1 | 2 | 0.5% DMSO | 0,5 | NA | 27,58977 | 0,123096 | 27,59 | 21,97 | 5,62 | 6,52 | 0,68649034 |  |  |
| PR30minCSR2 | 2 | 2 | 0.5% DMSO | 0,5 | NA | 29,49544 | 0,109348 | 29,50 | 22,57 | 6,93 | 6,52 | 0,68649034 |  |  |
| R16hCSR1 | 1 | 2 | 0.5% DMSO | 18 | NA | 31,13987 | 0,038143 | 31,14 | 22,90 | 8,24 | 6,52 | 0,68649034 |  |  |
| R16hCSR2 | 2 | 2 | 0.5% DMSO | 18 | NA | 30,06895 | 0,644179 | 30,07 | 22,21 | 7,86 | 6,52 | 0,68649034 |  |  |
| R1hCSR1 | 1 | 2 | 0.5% DMSO | 3 | NA | 29,20706 | 1,086697 | 29,21 | 23,14 | 6,06 | 6,52 | 0,68649034 |  |  |
| R1hCSR2 | 2 | 2 | 0.5% DMSO | 3 | NA | 28,68056 | 0,477684 | 28,68 | 22,39 | 6,29 | 6,52 | 0,68649034 |  |  |
| R2hCSR1 | 1 | 2 | 0.5% DMSO | 4 | NA | 28,33896 | 1,181373 | 28,34 | 22,43 | 5,91 | 6,52 | 0,68649034 |  |  |
| R2hCSR2 | 2 | 2 | 0.5% DMSO | 4 | NA | 27,90757 | 0,825889 | 27,91 | 22,18 | 5,72 | 6,52 | 0,68649034 |  |  |
| R4hCSR1 | 1 | 2 | 0.5% DMSO | 6 | NA | 28,59818 | 0,759954 | 28,60 | 22,44 | 6,16 | 6,52 | 0,68649034 |  |  |
| R4hCSR2 | 2 | 2 | 0.5% DMSO | 6 | NA | 28,72123 | 0,779481 | 28,72 | 22,27 | 6,45 | 6,52 | 0,68649034 |  |  |
| R8hCSR1 | 1 | 2 | 0.5% DMSO | 10 | NA | 29,36357 | 0,30683 | 29,36 | 22,66 | 6,71 | 6,52 | 0,68649034 |  |  |
| R8hCSR2 | 2 | 2 | 0.5% DMSO | 10 | NA | 28,60542 | 0,449607 | 28,61 | 22,63 | 5,98 | 6,52 | 0,68649034 |  |  |
| RC16h0.1R1 | 1 | 2 | 0.1 µM 20E | 18 | NA | 28,2591 | 0,770986 | 28,26 | 22,14 | 6,12 | 6,52 | 0,68649034 | -0,40 | 1,32 |
| RC16h0.1R2 | 2 | 2 | 0.1 µM 20E | 18 | NA | 29,41334 | 0,090481 | 29,41 | 22,13 | 7,28 | 6,52 | 0,68649034 | 0,77 | 0,59 |
| RC1h0.1R1 | 1 | 2 | 0.1 µM 20E | 3 | NA | 28,7508 | 0,152531 | 28,75 | 22,31 | 6,44 | 6,52 | 0,68649034 | -0,07 | 1,05 |
| RC1h0.1R2 | 2 | 2 | 0.1 µM 20E | 3 | NA | 29,38397 | 0,899734 | 29,38 | 22,58 | 6,81 | 6,52 | 0,68649034 | 0,29 | 0,82 |
| RC2h0.1R1 | 1 | 2 | 0.1 µM 20E | 4 | NA | 27,72443 | 0,326967 | 27,72 | 22,23 | 5,49 | 6,52 | 0,68649034 | -1,02 | 2,03 |
| RC2h0.1R2 | 2 | 2 | 0.1 µM 20E | 4 | NA | 29,38255 | 0,913246 | 29,38 | 22,70 | 6,68 | 6,52 | 0,68649034 | 0,17 | 0,89 |
| RC4h0.1R1 | 1 | 2 | 0.1 µM 20E | 6 | NA | 27,13743 | 0,777846 | 27,14 | 22,21 | 4,93 | 6,52 | 0,68649034 | -1,58 | 3,00 |
| RC4h0.1R2 | 2 | 2 | 0.1 µM 20E | 6 | NA | 29,4053 | 0,876738 | 29,41 | 22,33 | 7,08 | 6,52 | 0,68649034 | 0,56 | 0,68 |
| RC8h0.1R1 | 1 | 2 | 0.1 µM 20E | 10 | NA | 28,19256 | 0,21412 | 28,19 | 22,22 | 5,97 | 6,52 | 0,68649034 | -0,55 | 1,46 |
| RC8h0.1R2 | 2 | 2 | 0.1 µM 20E | 10 | NA | 28,58089 | 0,27909 | 28,58 | 22,50 | 6,08 | 6,52 | 0,68649034 | -0,44 | 1,35 |
| RR16h0.1R1 | 1 | 2 | 0.1 µM 20E | 2 | 16 | 29,07919 | 0,032915 | 29,08 | 22,06 | 7,01 | 6,52 | 0,68649034 | 0,50 | 0,71 |
| RR16h0.1R2 | 2 | 2 | 0.1 µM 20E | 2 | 16 | 27,33828 | 0,064717 | 27,34 | 21,29 | 6,05 | 6,52 | 0,68649034 | -0,47 | 1,39 |
| RR1h0.1R1 | 1 | 2 | 0.1 µM 20E | 2 | 1 | 30,41089 | 0 | 30,41 | 24,69 | 5,72 | 6,52 | 0,68649034 | -0,80 | 1,74 |
| RR1h0.1R2 | 2 | 2 | 0.1 µM 20E | 2 | 1 | 28,8014 | 0,115127 | 28,80 | 22,55 | 6,25 | 6,52 | 0,68649034 | -0,27 | 1,20 |
| RR2h0.1R1 | 1 | 2 | 0.1 µM 20E | 2 | 2 | 28,52838 | 0,482337 | 28,53 | 23,24 | 5,28 | 6,52 | 0,68649034 | -1,23 | 2,35 |
| RR2h0.1R2 | 2 | 2 | 0.1 µM 20E | 2 | 2 | 27,93793 | 0,177601 | 27,94 | 22,25 | 5,68 | 6,52 | 0,68649034 | -0,83 | 1,78 |
| RR4h0.1R1 | 1 | 2 | 0.1 µM 20E | 2 | 4 | 27,61166 | 0,364588 | 27,61 | 22,47 | 5,14 | 6,52 | 0,68649034 | -1,38 | 2,60 |
| RR4h0.1R2 | 2 | 2 | 0.1 µM 20E | 2 | 4 | 27,32694 | 0,097247 | 27,33 | 22,09 | 5,24 | 6,52 | 0,68649034 | -1,28 | 2,43 |
| RR8h0.1R1 | 1 | 2 | 0.1 µM 20E | 2 | 8 | 28,03949 | 0,216409 | 28,04 | 22,92 | 5,12 | 6,52 | 0,68649034 | -1,40 | 2,63 |
| RR8h0.1R2 | 2 | 2 | 0.1 µM 20E | 2 | 8 | 25,55498 | 0,989983 | 25,55 | 22,18 | 3,37 | 6,52 | 0,68649034 | -3,14 | 8,84 |
| PR1h0.1R1 | 1 | 3 | 0.1 µM 20E | 1 | NA | 29,5598 | 0,783777 | 29,56 | 21,66 | 7,90 | 7,46 | 1,66338785 | 0,44 | 0,74 |
| PR1h0.1R2 | 2 | 3 | 0.1 µM 20E | 1 | NA | 28,49451 | 0,010432 | 28,49 | 22,08 | 6,42 | 7,46 | 1,66338785 | -1,05 | 2,07 |
| PR1hCSR1 | 1 | 3 | 0.1 µM 20E | 1 | NA | 27,81325 | 0,188567 | 27,81 | 21,26 | 6,55 | 7,46 | 1,66338785 |  |  |
| PR1hCSR2 | 2 | 3 | 0.1 µM 20E | 1 | NA | 31,01603 | 1,195586 | 31,02 | 21,94 | 9,08 | 7,46 | 1,66338785 |  |  |
| PR2h0.1R1 | 1 | 3 | 0.1 µM 20E | 2 | NA | 30,91382 | 0,544474 | 30,91 | 22,27 | 8,65 | 7,46 | 1,66338785 | 1,18 | 0,44 |
| PR2h0.1R2 | 2 | 3 | 0.1 µM 20E | 2 | NA | 29,6327 | 0,373774 | 29,63 | 22,90 | 6,73 | 7,46 | 1,66338785 | -0,73 | 1,66 |
| PR2hCSR1 | 1 | 3 | 0.5% DMSO | 2 | NA | 30,73732 | 1,026148 | 30,74 | 21,14 | 9,60 | 7,46 | 1,66338785 |  |  |
| PR2hCSR2 | 2 | 3 | 0.5% DMSO | 2 | NA | 30,638 | 0,727561 | 30,64 | 22,37 | 8,27 | 7,46 | 1,66338785 |  |  |
| PR30min0.1R1 | 1 | 3 | 0.1 µM 20E | 0,5 | NA | 29,76527 | 0,468473 | 29,77 | 22,13 | 7,63 | 7,46 | 1,66338785 | 0,17 | 0,89 |
| PR30min0.1R2 | 2 | 3 | 0.1 µM 20E | 0,5 | NA | 29,82825 | 0,402277 | 29,83 | 22,43 | 7,40 | 7,46 | 1,66338785 | -0,07 | 1,05 |
| PR30minCSR1 | 1 | 3 | 0.5% DMSO | 0,5 | NA | 30,33407 | 0,792845 | 30,33 | 22,05 | 8,28 | 7,46 | 1,66338785 |  |  |
| PR30minCSR2 | 2 | 3 | 0.5% DMSO | 0,5 | NA | 32,38074 | 2,117175 | 32,38 | 21,65 | 10,73 | 7,46 | 1,66338785 |  |  |
| R16hCSR1 | 1 | 3 | 0.5% DMSO | 18 | NA | 33,04821 | 0,655105 | 33,05 | 24,97 | 8,08 | 7,46 | 1,66338785 |  |  |
| R16hCSR2 | 2 | 3 | 0.5% DMSO | 18 | NA | 34,71358 | 1,560616 | 34,71 | 24,64 | 10,07 | 7,46 | 1,66338785 |  |  |
| R1hCSR1 | 1 | 3 | 0.5% DMSO | 3 | NA | 31,59745 | 0,951058 | 31,60 | 26,16 | 5,44 | 7,46 | 1,66338785 |  |  |
| R1hCSR2 | 2 | 3 | 0.5% DMSO | 3 | NA | 30,90291 | 0,52082 | 30,90 | 24,71 | 6,19 | 7,46 | 1,66338785 |  |  |
| R2hCSR1 | 1 | 3 | 0.5% DMSO | 4 | NA | 32,52082 | 1,594147 | 32,52 | 25,86 | 6,66 | 7,46 | 1,66338785 |  |  |
| R2hCSR2 | 2 | 3 | 0.5% DMSO | 4 | NA | 30,49245 | 0,916009 | 30,49 | 25,37 | 5,13 | 7,46 | 1,66338785 |  |  |
| R4hCSR1 | 1 | 3 | 0.5% DMSO | 6 | NA | 32,70201 | 0,175609 | 32,70 | 26,51 | 6,20 | 7,46 | 1,66338785 |  |  |
| R4hCSR2 | 2 | 3 | 0.5% DMSO | 6 | NA | 29,6319 | 2,402079 | 29,63 | 23,63 | 6,00 | 7,46 | 1,66338785 |  |  |
| R8hCSR1 | 1 | 3 | 0.5% DMSO | 10 | NA | 32,31318 | 0,8597 | 32,31 | 25,82 | 6,49 | 7,46 | 1,66338785 |  |  |
| R8hCSR2 | 2 | 3 | 0.5% DMSO | 10 | NA | 30,20387 | 1,23134 | 30,20 | 23,53 | 6,67 | 7,46 | 1,66338785 |  |  |
| RC16h0.1R1 | 1 | 3 | 0.1 µM 20E | 18 | NA | 32,21373 | 0,079115 | 32,21 | 22,76 | 9,46 | 7,46 | 1,66338785 | 1,99 | 0,25 |
| RC16h0.1R2 | 2 | 3 | 0.1 µM 20E | 18 | NA | 33,62107 | 2,246655 | 33,62 | 22,70 | 10,92 | 7,46 | 1,66338785 | 3,45 | 0,09 |
| RC1h0.1R1 | 1 | 3 | 0.1 µM 20E | 3 | NA | 32,75028 | 0,805944 | 32,75 | 24,27 | 8,48 | 7,46 | 1,66338785 | 1,01 | 0,50 |
| RC1h0.1R2 | 2 | 3 | 0.1 µM 20E | 3 | NA | 33,60784 | 1,072881 | 33,61 | 24,45 | 9,16 | 7,46 | 1,66338785 | 1,69 | 0,31 |
| RC2h0.1R1 | 1 | 3 | 0.1 µM 20E | 4 | NA | 32,21849 | 2,408541 | 32,22 | 23,46 | 8,76 | 7,46 | 1,66338785 | 1,29 | 0,41 |
| RC2h0.1R2 | 2 | 3 | 0.1 µM 20E | 4 | NA | 34,38822 | 2,271237 | 34,39 | 23,47 | 10,91 | 7,46 | 1,66338785 | 3,45 | 0,09 |
| RC4h0.1R1 | 1 | 3 | 0.1 µM 20E | 6 | NA | 32,86361 | 1,966871 | 32,86 | 23,81 | 9,05 | 7,46 | 1,66338785 | 1,59 | 0,33 |
| RC4h0.1R2 | 2 | 3 | 0.1 µM 20E | 6 | NA | 34,387 | 2,283152 | 34,39 | 24,12 | 10,27 | 7,46 | 1,66338785 | 2,80 | 0,14 |
| RC8h0.1R1 | 1 | 3 | 0.1 µM 20E | 10 | NA | 32,60074 | 1,564978 | 32,60 | 22,51 | 10,09 | 7,46 | 1,66338785 | 2,62 | 0,16 |
| RC8h0.1R2 | 2 | 3 | 0.1 µM 20E | 10 | NA | 33,26797 | 1,76858 | 33,27 | 23,33 | 9,94 | 7,46 | 1,66338785 | 2,47 | 0,18 |
| RR16h0.1R1 | 1 | 3 | 0.1 µM 20E | 2 | 16 | 30,3458 | 0,351225 | 30,35 | 22,20 | 8,14 | 7,46 | 1,66338785 | 0,68 | 0,63 |
| RR16h0.1R2 | 2 | 3 | 0.1 µM 20E | 2 | 16 | 29,79585 | 0,813979 | 29,80 | 21,39 | 8,40 | 7,46 | 1,66338785 | 0,94 | 0,52 |
| RR1h0.1R1 | 1 | 3 | 0.1 µM 20E | 2 | 1 | 31,07468 | 0,749519 | 31,07 | 23,37 | 7,71 | 7,46 | 1,66338785 | 0,24 | 0,84 |
| RR1h0.1R2 | 2 | 3 | 0.1 µM 20E | 2 | 1 | 30,66692 | 0,448916 | 30,67 | 23,64 | 7,03 | 7,46 | 1,66338785 | -0,44 | 1,36 |
| RR2h0.1R1 | 1 | 3 | 0.1 µM 20E | 2 | 2 | 30,61929 | 0,443726 | 30,62 | 23,24 | 7,38 | 7,46 | 1,66338785 | -0,09 | 1,06 |
| RR2h0.1R2 | 2 | 3 | 0.1 µM 20E | 2 | 2 | 29,75299 | 0,614676 | 29,75 | 22,70 | 7,06 | 7,46 | 1,66338785 | -0,41 | 1,33 |
| RR4h0.1R1 | 1 | 3 | 0.1 µM 20E | 2 | 4 | 31,05816 | 0,325615 | 31,06 | 23,41 | 7,65 | 7,46 | 1,66338785 | 0,18 | 0,88 |
| RR4h0.1R2 | 2 | 3 | 0.1 µM 20E | 2 | 4 | 30,32844 | 1,315921 | 30,33 | 23,58 | 6,75 | 7,46 | 1,66338785 | -0,72 | 1,64 |
| RR8h0.1R1 | 1 | 3 | 0.1 µM 20E | 2 | 8 | 29,77167 | 0,495203 | 29,77 | 22,76 | 7,02 | 7,46 | 1,66338785 | -0,45 | 1,37 |
| RR8h0.1R2 | 2 | 3 | 0.1 µM 20E | 2 | 8 | 30,1859 | 0,735034 | 30,19 | 22,54 | 7,64 | 7,46 | 1,66338785 | 0,18 | 0,88 |
| 20ERC8h0.003R1 | 1 | 5 | 0.003 µM 20E | 10 | NA | 29,31274 | 0,397812 | 29,31 | 23,60 | 5,71 | 5,45 | 0,11670551 | 0,26 | 0,83 |
| 20ERC8h0.003R2 | 2 | 5 | 0.003 µM 20E | 10 | NA | 30,20364 | 0,519709 | 30,20 | 23,18 | 7,03 | 5,45 | 0,11670551 | 1,58 | 0,33 |
| 20ERC8h0.003R3 | 3 | 5 | 0.003 µM 20E | 10 | NA | 30,9248 | 0,250618 | 30,92 | 24,35 | 6,58 | 5,45 | 0,11670551 | 1,13 | 0,46 |
| 20ERC8h0.01R1 | 1 | 5 | 0.01 µM 20E | 10 | NA | 28,00296 | 0,292062 | 28,00 | 22,42 | 5,58 | 5,45 | 0,11670551 | 0,13 | 0,91 |
| 20ERC8h0.01R2 | 2 | 5 | 0.01 µM 20E | 10 | NA | 29,84422 | 0,189736 | 29,84 | 22,93 | 6,92 | 5,45 | 0,11670551 | 1,47 | 0,36 |
| 20ERC8h0.01R3 | 3 | 5 | 0.01 µM 20E | 10 | NA | 27,34368 | 0,010295 | 27,34 | 22,95 | 4,39 | 5,45 | 0,11670551 | -1,06 | 2,08 |
| 20ERC8h0.03R1 | 1 | 5 | 0.03 µM 20E | 10 | NA | 28,02606 | 0,110977 | 28,03 | 22,03 | 6,00 | 5,45 | 0,11670551 | 0,55 | 0,68 |
| 20ERC8h0.03R2 | 2 | 5 | 0.03 µM 20E | 10 | NA | 30,42876 | 0,504573 | 30,43 | 22,17 | 8,26 | 5,45 | 0,11670551 | 2,81 | 0,14 |
| 20ERC8h0.03R3 | 3 | 5 | 0.03 µM 20E | 10 | NA | 29,14137 | 0,015304 | 29,14 | 23,13 | 6,01 | 5,45 | 0,11670551 | 0,56 | 0,68 |
| 20ERC8h0.1R1 | 1 | 5 | 0.1 µM 20E | 10 | NA | 29,33077 | 0,466106 | 29,33 | 22,27 | 7,06 | 5,45 | 0,11670551 | 1,62 | 0,33 |
| 20ERC8h0.1R2 | 2 | 5 | 0.1 µM 20E | 10 | NA | 30,81285 | 0,158697 | 30,81 | 23,17 | 7,64 | 5,45 | 0,11670551 | 2,20 | 0,22 |
| 20ERC8h0.1R3 | 3 | 5 | 0.1 µM 20E | 10 | NA | 29,88373 | 0,306116 | 29,88 | 23,08 | 6,80 | 5,45 | 0,11670551 | 1,35 | 0,39 |
| 20ERC8h0.3R1 | 1 | 5 | 0.3 µM 20E | 10 | NA | 29,17116 | 0,579817 | 29,17 | 22,53 | 6,64 | 5,45 | 0,11670551 | 1,20 | 0,44 |
| 20ERC8h0.3R2 | 2 | 5 | 0.3 µM 20E | 10 | NA | 30,15738 | 0,813118 | 30,16 | 22,47 | 7,69 | 5,45 | 0,11670551 | 2,24 | 0,21 |
| 20ERC8h0.3R3 | 3 | 5 | 0.3 µM 20E | 10 | NA |  | 0 |  |  |  | 5,45 | 0,11670551 |  |  |
| 20ERC8h1R1 | 1 | 5 | 1 µM 20E | 10 | NA | 31,07123 | 0,673134 | 31,07 | 21,96 | 9,11 | 5,45 | 0,11670551 | 3,66 | 0,08 |
| 20ERC8h1R2 | 2 | 5 | 1 µM 20E | 10 | NA | 33,76314 | 0,546603 | 33,76 | 25,05 | 8,71 | 5,45 | 0,11670551 | 3,27 | 0,10 |
| 20ERC8h1R3 | 3 | 5 | 1 µM 20E | 10 | NA | 32,71295 | 0,737102 | 32,71 | 25,69 | 7,02 | 5,45 | 0,11670551 | 1,57 | 0,34 |
| 20ERC8h3R1 | 1 | 5 | 3 µM 20E | 10 | NA | 31,32898 | 0,653774 | 31,33 | 23,89 | 7,44 | 5,45 | 0,11670551 | 1,99 | 0,25 |
| 20ERC8h3R2 | 2 | 5 | 3 µM 20E | 10 | NA | 31,90103 | 0,024898 | 31,90 | 22,99 | 8,91 | 5,45 | 0,11670551 | 3,46 | 0,09 |
| 20ERC8h3R3 | 3 | 5 | 3 µM 20E | 10 | NA | 31,28007 | 0,284908 | 31,28 | 23,01 | 8,27 | 5,45 | 0,11670551 | 2,82 | 0,14 |
| 20ERR8h0.003R1 | 1 | 5 | 0.003 µM 20E | 2 | 8 | 27,36238 | 0,086277 | 27,36 | 22,12 | 5,24 | 5,45 | 0,11670551 | -0,20 | 1,15 |
| 20ERR8h0.003R2 | 2 | 5 | 0.003 µM 20E | 2 | 8 | 27,40183 | 0,122328 | 27,40 | 21,83 | 5,58 | 5,45 | 0,11670551 | 0,13 | 0,92 |
| 20ERR8h0.003R3 | 3 | 5 | 0.003 µM 20E | 2 | 8 | 27,31877 | 0,335323 | 27,32 | 22,43 | 4,89 | 5,45 | 0,11670551 | -0,56 | 1,47 |
| 20ERR8h0.01R1 | 1 | 5 | 0.01 µM 20E | 2 | 8 | 27,66937 | 0,054535 | 27,67 | 22,58 | 5,09 | 5,45 | 0,11670551 | -0,36 | 1,28 |
| 20ERR8h0.01R2 | 2 | 5 | 0.01 µM 20E | 2 | 8 | 27,71269 | 0,14244 | 27,71 | 22,19 | 5,52 | 5,45 | 0,11670551 | 0,07 | 0,95 |
| 20ERR8h0.01R3 | 3 | 5 | 0.01 µM 20E | 2 | 8 | 32,93124 | 0,026393 | 32,93 | 25,02 | 7,91 | 5,45 | 0,11670551 |  |  |
| 20ERR8h0.03R1 | 1 | 5 | 0.03 µM 20E | 2 | 8 | 27,44157 | 0,163286 | 27,44 | 22,28 | 5,17 | 5,45 | 0,11670551 | -0,28 | 1,22 |
| 20ERR8h0.03R2 | 2 | 5 | 0.03 µM 20E | 2 | 8 | 26,59155 | 0,029384 | 26,59 | 21,48 | 5,11 | 5,45 | 0,11670551 | -0,34 | 1,26 |
| 20ERR8h0.03R3 | 3 | 5 | 0.03 µM 20E | 2 | 8 | 28,02155 | 0,442274 | 28,02 | 22,16 | 5,86 | 5,45 | 0,11670551 | 0,41 | 0,75 |
| 20ERR8h0.1R1 | 1 | 5 | 0.1 µM 20E | 2 | 8 | 26,79892 | 0,101036 | 26,80 | 21,93 | 4,86 | 5,45 | 0,11670551 | -0,58 | 1,50 |
| 20ERR8h0.1R2 | 2 | 5 | 0.1 µM 20E | 2 | 8 | 27,85058 | 0,050759 | 27,85 | 22,35 | 5,50 | 5,45 | 0,11670551 | 0,06 | 0,96 |
| 20ERR8h0.1R3 | 3 | 5 | 0.1 µM 20E | 2 | 8 | 27,98204 | 0,730203 | 27,98 | 22,40 | 5,58 | 5,45 | 0,11670551 | 0,13 | 0,91 |
| 20ERR8h0.3R1 | 1 | 5 | 0.3 µM 20E | 2 | 8 | 26,83219 | 0,026763 | 26,83 | 21,93 | 4,91 | 5,45 | 0,11670551 | -0,54 | 1,46 |
| 20ERR8h0.3R2 | 2 | 5 | 0.3 µM 20E | 2 | 8 | 27,31172 | 0,041775 | 27,31 | 21,63 | 5,68 | 5,45 | 0,11670551 | 0,24 | 0,85 |
| 20ERR8h0.3R3 | 3 | 5 | 0.3 µM 20E | 2 | 8 | 31,57063 | 0,377989 | 31,57 | 23,92 | 7,65 | 5,45 | 0,11670551 |  |  |
| 20ERR8h1R1 | 1 | 5 | 1 µM 20E | 2 | 8 | 26,3122 | 0,130173 | 26,31 | 21,35 | 4,96 | 5,45 | 0,11670551 | -0,49 | 1,40 |
| 20ERR8h1R2 | 2 | 5 | 1 µM 20E | 2 | 8 | 27,11803 | 0,145634 | 27,12 | 21,63 | 5,49 | 5,45 | 0,11670551 | 0,04 | 0,97 |
| 20ERR8h1R3 | 3 | 5 | 1 µM 20E | 2 | 8 | 27,27363 | 0,238541 | 27,27 | 22,30 | 4,97 | 5,45 | 0,11670551 | -0,47 | 1,39 |
| 20ERR8h3R1 | 1 | 5 | 3 µM 20E | 2 | 8 | 29,08979 | 0,147109 | 29,09 | 20,99 | 8,10 | 5,45 | 0,11670551 | 2,65 | 0,16 |
| 20ERR8h3R2 | 2 | 5 | 3 µM 20E | 2 | 8 | 27,07229 | 0,04004 | 27,07 | 21,79 | 5,28 | 5,45 | 0,11670551 | -0,17 | 1,13 |
| 20ERR8h3R3 | 3 | 5 | 3 µM 20E | 2 | 8 | 28,00775 | 0,225425 | 28,01 | 22,24 | 5,76 | 5,45 | 0,11670551 | 0,32 | 0,80 |
| SC8hR1 | 1 | 5 | 0.5% DMSO | 10 | NA | 27,42007 | 0,099193 | 27,42 | 22,01 | 5,41 | 5,45 | 0,11670551 |  |  |
| SC8hR2 | 2 | 5 | 0.5% DMSO | 10 | NA | 27,23319 | 0,055088 | 27,23 | 21,90 | 5,33 | 5,45 | 0,11670551 |  |  |
| SC8hR3 | 3 | 5 | 0.5% DMSO | 10 | NA | 28,27695 | 0,260799 | 28,28 | 22,67 | 5,61 | 5,45 | 0,11670551 |  |  |
| PR2h0.003R1 | 1 | 4 | 0.003 µM 20E | 2 | NA | 27,84604 | 0,125736 | 27,85 | 24,07 | 3,78 | 4,33 | 0,09831838 | -0,54653 | 1,460569 |
| PR2h0.003R2 | 2 | 4 | 0.003 µM 20E | 2 | NA | 27,90449 | 0,133616 | 27,90 | 23,80 | 4,11 | 4,33 | 0,09831838 | -0,21666 | 1,162036 |
| PR2h0.003R3 | 3 | 4 | 0.003 µM 20E | 2 | NA | 28,48929 | 0,14658 | 28,49 | 24,27 | 4,22 | 4,33 | 0,09831838 | -0,1091 | 1,078554 |
| PR2h0.01R1 | 1 | 4 | 0.01 µM 20E | 2 | NA | 27,81282 | 0,270289 | 27,81 | 24,06 | 3,75 | 4,33 | 0,09831838 | -0,57272 | 1,487327 |
| PR2h0.01R2 | 2 | 4 | 0.01 µM 20E | 2 | NA | 27,94419 | 0,165708 | 27,94 | 23,76 | 4,18 | 4,33 | 0,09831838 | -0,14427 | 1,105172 |
| PR2h0.01R3 | 3 | 4 | 0.01 µM 20E | 2 | NA | 28,12098 | 0,203549 | 28,12 | 23,92 | 4,20 | 4,33 | 0,09831838 | -0,12316 | 1,089117 |
| PR2h0.03R1 | 1 | 4 | 0.03 µM 20E | 2 | NA | 29,01395 | 1,386332 | 29,01 | 23,84 | 5,18 | 4,33 | 0,09831838 | 0,85107 | 0,554373 |
| PR2h0.03R2 | 2 | 4 | 0.03 µM 20E | 2 | NA | 28,66025 | 0,115772 | 28,66 | 23,99 | 4,67 | 4,33 | 0,09831838 | 0,346015 | 0,786754 |
| PR2h0.03R3 | 3 | 4 | 0.03 µM 20E | 2 | NA | 28,13178 | 0,016809 | 28,13 | 24,00 | 4,13 | 4,33 | 0,09831838 | -0,19136 | 1,141842 |
| PR2h0.1R1 | 1 | 4 | 0.1 µM 20E | 2 | NA | 28,40814 | 0,142965 | 28,41 | 23,86 | 4,55 | 4,33 | 0,09831838 | 0,219106 | 0,859098 |
| PR2h0.1R2 | 2 | 4 | 0.1 µM 20E | 2 | NA | 28,33348 | 0,001427 | 28,33 | 24,01 | 4,32 | 4,33 | 0,09831838 | -0,00259 | 1,001799 |
| PR2h0.1R3 | 3 | 4 | 0.1 µM 20E | 2 | NA | 28,31657 | 0,139622 | 28,32 | 23,76 | 4,56 | 4,33 | 0,09831838 | 0,232565 | 0,85112 |
| PR2h0.3R1 | 1 | 4 | 0.3 µM 20E | 2 | NA | 28,0298 | 0,021359 | 28,03 | 23,19 | 4,84 | 4,33 | 0,09831838 | 0,512921 | 0,700802 |
| PR2h0.3R2 | 2 | 4 | 0.3 µM 20E | 2 | NA | 28,74853 | 0,180603 | 28,75 | 24,12 | 4,62 | 4,33 | 0,09831838 | 0,298556 | 0,813066 |
| PR2h0.3R3 | 3 | 4 | 0.3 µM 20E | 2 | NA | 28,51724 | 0,052405 | 28,52 | 23,92 | 4,60 | 4,33 | 0,09831838 | 0,272001 | 0,82817 |
| PR2h1R1 | 1 | 4 | 1 µM 20E | 2 | NA | 29,39184 | 0,149967 | 29,39 | 24,26 | 5,13 | 4,33 | 0,09831838 | 0,80278 | 0,573243 |
| PR2h1R2 | 2 | 4 | 1 µM 20E | 2 | NA | 29,18175 | 0,151223 | 29,18 | 24,50 | 4,68 | 4,33 | 0,09831838 | 0,354179 | 0,782314 |
| PR2h1R3 | 3 | 4 | 1 µM 20E | 2 | NA | 28,77127 | 0,106816 | 28,77 | 24,34 | 4,43 | 4,33 | 0,09831838 | 0,108519 | 0,92754 |
| PR2h3R1 | 1 | 4 | 3 µM 20E | 2 | NA | 29,28086 | 0,161874 | 29,28 | 25,00 | 4,28 | 4,33 | 0,09831838 | -0,04729 | 1,033319 |
| PR2h3R2 | 2 | 4 | 3 µM 20E | 2 | NA | 29,59924 | 0,27405 | 29,60 | 24,48 | 5,12 | 4,33 | 0,09831838 | 0,792557 | 0,57732 |
| PR2h3R3 | 3 | 4 | 3 µM 20E | 2 | NA | 29,19937 | 0,085899 | 29,20 | 25,06 | 4,14 | 4,33 | 0,09831838 | -0,18501 | 1,136824 |
| PR2hCSR1 | 1 | 4 | 0.5% DMSO | 2 | NA | 27,46372 | 0,125726 | 27,46 | 23,18 | 4,29 | 4,33 | 0,09831838 |  |  |
| PR2hCSR2 | 2 | 4 | 0.5% DMSO | 2 | NA | 27,93769 | 0,084793 | 27,94 | 23,48 | 4,46 | 4,33 | 0,09831838 |  |  |
| PR2hCSR3 | 3 | 4 | 0.5% DMSO | 2 | NA | 27,55309 | 0,312134 | 27,55 | 23,32 | 4,23 | 4,33 | 0,09831838 |  |  |
| RC8h0.003R1 | 1 | 4 | 0.003 µM 20E | 10 | NA | 28,72326 | 0,173351 | 28,72 | 25,33 | 3,39 | 4,58 | 0,370207 | -1,18721 | 2,277121 |
| RC8h0.003R2 | 2 | 4 | 0.003 µM 20E | 10 | NA | 28,55702 | 0,050097 | 28,56 | 24,11 | 4,45 | 4,58 | 0,370207 | -0,13166 | 1,09555 |
| RC8h0.003R3 | 3 | 4 | 0.003 µM 20E | 10 | NA | 29,56237 | 0,102413 | 29,56 | 24,03 | 5,53 | 4,58 | 0,370207 | 0,953015 | 0,516552 |
| RC8h0.01R1 | 1 | 4 | 0.01 µM 20E | 10 | NA | 29,0246 | 0,33181 | 29,02 | 24,72 | 4,30 | 4,58 | 0,370207 | -0,27591 | 1,210761 |
| RC8h0.01R2 | 2 | 4 | 0.01 µM 20E | 10 | NA | 29,0887 | 0,034544 | 29,09 | 24,10 | 4,99 | 4,58 | 0,370207 | 0,41289 | 0,751117 |
| RC8h0.01R3 | 3 | 4 | 0.01 µM 20E | 10 | NA | 28,4726 | 0,349417 | 28,47 | 23,83 | 4,64 | 4,58 | 0,370207 | 0,063645 | 0,956844 |
| RC8h0.03R1 | 1 | 4 | 0.03 µM 20E | 10 | NA | 29,24093 | 5,88E-07 | 29,24 | 24,09 | 5,15 | 4,58 | 0,370207 | 0,57468 | 0,671435 |
| RC8h0.03R2 | 2 | 4 | 0.03 µM 20E | 10 | NA | 28,94276 | 0,130951 | 28,94 | 23,06 | 5,88 | 4,58 | 0,370207 | 1,302942 | 0,405299 |
| RC8h0.03R3 | 3 | 4 | 0.03 µM 20E | 10 | NA | 29,51308 | 0,078473 | 29,51 | 23,93 | 5,59 | 4,58 | 0,370207 | 1,009153 | 0,496838 |
| RC8h0.1R1 | 1 | 4 | 0.1 µM 20E | 10 | NA | 30,67495 | 0,174802 | 30,67 | 25,61 | 5,07 | 4,58 | 0,370207 | 0,492954 | 0,710569 |
| RC8h0.1R2 | 2 | 4 | 0.1 µM 20E | 10 | NA | 30,35816 | 0,191961 | 30,36 | 24,75 | 5,61 | 4,58 | 0,370207 | 1,030632 | 0,489496 |
| RC8h0.1R3 | 3 | 4 | 0.1 µM 20E | 10 | NA | 29,93274 | 0,107321 | 29,93 | 24,38 | 5,55 | 4,58 | 0,370207 | 0,970907 | 0,510185 |
| RC8h0.3R1 | 1 | 4 | 0.3 µM 20E | 10 | NA | 30,08312 | 0,364646 | 30,08 | 25,04 | 5,05 | 4,58 | 0,370207 | 0,46842 | 0,722756 |
| RC8h0.3R2 | 2 | 4 | 0.3 µM 20E | 10 | NA | 30,62175 | 0,084617 | 30,62 | 25,00 | 5,62 | 4,58 | 0,370207 | 1,046482 | 0,484147 |
| RC8h0.3R3 | 3 | 4 | 0.3 µM 20E | 10 | NA | 30,37341 | 0,016791 | 30,37 | 25,16 | 5,22 | 4,58 | 0,370207 | 0,640375 | 0,641546 |
| RC8h1R1 | 1 | 4 | 1 µM 20E | 10 | NA | 30,79272 | 0,097509 | 30,79 | 25,66 | 5,13 | 4,58 | 0,370207 | 0,554141 | 0,681062 |
| RC8h1R2 | 2 | 4 | 1 µM 20E | 10 | NA | 30,63646 | 0,108019 | 30,64 | 25,14 | 5,50 | 4,58 | 0,370207 | 0,918184 | 0,529175 |
| RC8h1R3 | 3 | 4 | 1 µM 20E | 10 | NA | 31,01546 | 0,005534 | 31,02 | 25,46 | 5,56 | 4,58 | 0,370207 | 0,983399 | 0,505787 |
| RC8h3R1 | 1 | 4 | 3 µM 20E | 10 | NA | 32,1869 | 0,529595 | 32,19 | 26,04 | 6,14 | 4,58 | 0,370207 | 1,565012 | 0,337975 |
| RC8h3R2 | 2 | 4 | 3 µM 20E | 10 | NA | 32,05292 | 0,152346 | 32,05 | 25,98 | 6,07 | 4,58 | 0,370207 | 1,497413 | 0,354188 |
| RC8h3R3 | 3 | 4 | 3 µM 20E | 10 | NA | 32,4395 | 0,053782 | 32,44 | 25,65 | 6,79 | 4,58 | 0,370207 | 2,215815 | 0,215265 |
| RC8hCSR1 | 1 | 4 | 0.5% DMSO | 10 | NA | 28,54152 | 0,001396 | 28,54 | 24,55 | 3,99 | 4,58 | 0,370207 |  |  |
| RC8hCSR2 | 2 | 4 | 0.5% DMSO | 10 | NA | 29,24445 | 0,152118 | 29,24 | 24,86 | 4,38 | 4,58 | 0,370207 |  |  |
| RC8hCSR3 | 3 | 4 | 0.5% DMSO | 10 | NA | 29,23918 | 0,009903 | 29,24 | 24,16 | 5,08 | 4,58 | 0,370207 |  |  |
| RR8h0.003R1 | 1 | 4 | 0.003 µM 20E | 2 | 8 | 27,83652 | 0,154537 | 27,84 | 24,03 | 3,80 | 4,58 | 0,370207 | -0,77414 | 1,710165 |
| RR8h0.003R2 | 2 | 4 | 0.003 µM 20E | 2 | 8 | 28,29609 | 0,188134 | 28,30 | 24,48 | 3,82 | 4,58 | 0,370207 | -0,75611 | 1,688933 |
| RR8h0.003R3 | 3 | 4 | 0.003 µM 20E | 2 | 8 | 28,35794 | 0,119472 | 28,36 | 24,19 | 4,17 | 4,58 | 0,370207 | -0,40863 | 1,327422 |
| RR8h0.01R1 | 1 | 4 | 0.01 µM 20E | 2 | 8 | 28,27361 | 0,106222 | 28,27 | 24,30 | 3,98 | 4,58 | 0,370207 | -0,60028 | 1,516015 |
| RR8h0.01R2 | 2 | 4 | 0.01 µM 20E | 2 | 8 | 28,68498 | 0,172236 | 28,68 | 24,31 | 4,38 | 4,58 | 0,370207 | -0,20157 | 1,149948 |
| RR8h0.01R3 | 3 | 4 | 0.01 µM 20E | 2 | 8 | 28,14091 | 0,050178 | 28,14 | 23,75 | 4,39 | 4,58 | 0,370207 | -0,185 | 1,136818 |
| RR8h0.03R2 | 2 | 4 | 0.03 µM 20E | 2 | 8 | 28,54123 | 0,084396 | 28,54 | 24,76 | 3,78 | 4,58 | 0,370207 | -0,79759 | 1,73819 |
| RR8h0.03R3 | 3 | 4 | 0.03 µM 20E | 2 | 8 | 27,99231 | 0,00877 | 27,99 | 23,63 | 4,36 | 4,58 | 0,370207 | -0,21465 | 1,160423 |
| RR8h0.1R1 | 1 | 4 | 0.1 µM 20E | 2 | 8 | 27,32766 | 0,168411 | 27,33 | 23,92 | 3,41 | 4,58 | 0,370207 | -1,16524 | 2,242708 |
| RR8h0.1R2 | 2 | 4 | 0.1 µM 20E | 2 | 8 | 28,34335 | 0,177808 | 28,34 | 23,83 | 4,51 | 4,58 | 0,370207 | -0,06371 | 1,045152 |
| RR8h0.3R1 | 1 | 4 | 0.3 µM 20E | 2 | 8 | 27,83081 | 0,183785 | 27,83 | 24,70 | 3,13 | 4,58 | 0,370207 | -1,44274 | 2,718371 |
| RR8h0.3R2 | 2 | 4 | 0.3 µM 20E | 2 | 8 | 27,33212 | 0,102961 | 27,33 | 23,94 | 3,40 | 4,58 | 0,370207 | -1,17996 | 2,265703 |
| RR8h1R1 | 1 | 4 | 1 µM 20E | 2 | 8 | 27,68092 | 0,034842 | 27,68 | 24,57 | 3,11 | 4,58 | 0,370207 | -1,46833 | 2,767008 |
| RR8h1R2 | 2 | 4 | 1 µM 20E | 2 | 8 | 27,79821 | 0,335114 | 27,80 | 23,92 | 3,88 | 4,58 | 0,370207 | -0,70119 | 1,625847 |
| RR8h1R3 | 3 | 4 | 1 µM 20E | 2 | 8 | 28,13667 | 0,157989 | 28,14 | 24,32 | 3,82 | 4,58 | 0,370207 | -0,75843 | 1,691654 |
| RR8h3R1 | 1 | 4 | 3 µM 20E | 2 | 8 | 28,20349 | 0,003074 | 28,20 | 25,54 | 2,67 | 4,58 | 0,370207 | -1,91031 | 3,758908 |
| RR8h3R2 | 2 | 4 | 3 µM 20E | 2 | 8 | 28,70954 | 0,034497 | 28,71 | 24,38 | 4,33 | 4,58 | 0,370207 | -0,24881 | 1,188225 |
| RR8hCSR1 | 1 | 4 | 0.5% DMSO | 10 | NA | 28,10182 | 0,04571 | 28,10 | 23,66 | 4,44 | 4,58 | 0,370207 |  |  |
| RR8hCSR2 | 2 | 4 | 0.5% DMSO | 10 | NA | 28,16398 | 0,076145 | 28,16 | 23,17 | 4,99 | 4,58 | 0,370207 |  |  |
| RR8hCSR3 | 3 | 4 | 0.5% DMSO | 10 | NA | 28,43218 | 0,128484 | 28,43 | 23,85 | 4,58 | 4,58 | 0,370207 |  |  |
| 20ERC8h0.003R1 | 1 | 6 | 0.003 µM 20E | 10 | NA | 28,49043 | 0,033568 | 28,49 | 24,67 | 3,82 | 7,45 | 0,74181759 | -3,63769 | 12,4467 |
| 20ERC8h0.003R2 | 2 | 6 | 0.003 µM 20E | 10 | NA | 30,66686 | 1,218771 | 30,67 | 25,05 | 5,62 | 7,45 | 0,74181759 | -1,83826 | 3,575786 |
| 20ERC8h0.01R1 | 1 | 6 | 0.01 µM 20E | 10 | NA | 29,61235 | 0,228162 | 29,61 | 24,37 | 5,24 | 7,45 | 0,74181759 | -2,21129 | 4,630883 |
| 20ERC8h0.01R2 | 2 | 6 | 0.01 µM 20E | 10 | NA | 29,73448 | 1,02231 | 29,73 | 23,19 | 6,55 | 7,45 | 0,74181759 | -0,90679 | 1,87487 |
| 20ERC8h0.01R3 | 3 | 6 | 0.01 µM 20E | 10 | NA | 31,54744 | 4,02209 | 31,55 | 24,46 | 7,08 | 7,45 | 0,74181759 | -0,37095 | 1,293206 |
| 20ERC8h0.03R1 | 1 | 6 | 0.03 µM 20E | 10 | NA | 28,22279 | 0,06434 | 28,22 | 23,35 | 4,87 | 7,45 | 0,74181759 | -2,57945 | 5,977123 |
| 20ERC8h0.03R3 | 3 | 6 | 0.03 µM 20E | 10 | NA | 34,67037 | 4,832464 | 34,67 | 24,11 | 10,56 | 7,45 | 0,74181759 | 3,108795 | 0,11592 |
| 20ERC8h0.1R2 | 2 | 6 | 0.1 µM 20E | 10 | NA | 32,92538 | 1,086225 | 32,93 | 23,06 | 9,86 | 7,45 | 0,74181759 | 2,407445 | 0,188489 |
| 20ERC8h0.1R3 | 3 | 6 | 0.1 µM 20E | 10 | NA | 34,951 | 5,390559 | 34,95 | 24,10 | 10,85 | 7,45 | 0,74181759 | 3,393664 | 0,095149 |
| 20ERC8h0.3R1 | 1 | 6 | 0.3 µM 20E | 10 | NA | 32,26456 | 0,307037 | 32,26 | 24,44 | 7,82 | 7,45 | 0,74181759 | 0,369149 | 0,774239 |
| 20ERC8h0.3R2 | 2 | 6 | 0.3 µM 20E | 10 | NA | 30,57931 | 1,460876 | 30,58 | 22,92 | 7,66 | 7,45 | 0,74181759 | 0,20846 | 0,865461 |
| 20ERC8h0.3R3 | 3 | 6 | 0.3 µM 20E | 10 | NA | 32,89156 | 4,698236 | 32,89 | 23,73 | 9,16 | 7,45 | 0,74181759 | 1,702864 | 0,307176 |
| 20ERC8h1R1 | 1 | 6 | 1 µM 20E | 10 | NA | 30,65914 | 0,287216 | 30,66 | 23,67 | 6,99 | 7,45 | 0,74181759 | -0,46184 | 1,377294 |
| 20ERC8h1R2 | 2 | 6 | 1 µM 20E | 10 | NA | 35,13071 | 1,097489 | 35,00 | 24,93 | 10,07 | 7,45 | 0,74181759 | 2,618445 | 0,162843 |
| 20ERC8h1R3 | 3 | 6 | 1 µM 20E | 10 | NA | 32,60276 | 3,298914 | 32,60 | 23,62 | 8,98 | 7,45 | 0,74181759 | 1,530497 | 0,346158 |
| 20ERC8h3R1 | 1 | 6 | 3 µM 20E | 10 | NA | 31,56825 | 0,103712 | 31,57 | 23,40 | 8,17 | 7,45 | 0,74181759 | 0,717866 | 0,607996 |
| 20ERC8h3R2 | 2 | 6 | 3 µM 20E | 10 | NA | 33,50551 | 1,65807 | 33,51 | 24,86 | 8,65 | 7,45 | 0,74181759 | 1,194871 | 0,436825 |
| 20ERC8h3R3 | 3 | 6 | 3 µM 20E | 10 | NA | 32,66305 | 2,539891 | 32,66 | 24,27 | 8,40 | 7,45 | 0,74181759 | 0,942887 | 0,520191 |
| 20ERR8h0.01R2 | 2 | 6 | 0.01 µM 20E | 2 | 8 | 28,78142 | 0,65002 | 28,78 | 22,08 | 6,70 | 7,45 | 0,74181759 | -0,7522 | 1,684364 |
| 20ERR8h0.01R3 | 3 | 6 | 0.01 µM 20E | 2 | 8 | 28,81129 | 0,429073 | 28,81 | 22,82 | 5,99 | 7,45 | 0,74181759 | -1,46105 | 2,753086 |
| 20ERR8h0.03R3 | 3 | 6 | 0.03 µM 20E | 2 | 8 | 33,02854 | 0,029418 | 33,03 | 24,95 | 8,08 | 7,45 | 0,74181759 | 0,626181 | 0,647889 |
| 20ERR8h0.1R1 | 1 | 6 | 0.1 µM 20E | 2 | 8 | 29,6932 | 0,506002 | 29,69 | 25,83 | 3,86 | 7,45 | 0,74181759 | -3,59094 | 12,04981 |
| 20ERR8h0.1R2 | 2 | 6 | 0.1 µM 20E | 2 | 8 | 28,50715 | 0,984015 | 28,51 | 22,47 | 6,04 | 7,45 | 0,74181759 | -1,41417 | 2,66507 |
| 20ERR8h0.1R3 | 3 | 6 | 0.1 µM 20E | 2 | 8 | 27,99668 | 0,058354 | 28,00 | 23,09 | 4,91 | 7,45 | 0,74181759 | -2,5458 | 5,839301 |
| 20ERR8h0.3R3 | 3 | 6 | 0.3 µM 20E | 2 | 8 | 27,1713 | 0,237937 | 27,17 | 22,70 | 4,47 | 7,45 | 0,74181759 | -2,98091 | 7,894843 |
| 20ERR8h1R2 | 2 | 6 | 1 µM 20E | 2 | 8 | 28,14925 | 0,797872 | 28,15 | 22,32 | 5,83 | 7,45 | 0,74181759 | -1,62615 | 3,086871 |
| 20ERR8h3R1 | 1 | 6 | 3 µM 20E | 2 | 8 | 29,32744 | 0,992077 | 29,33 | 23,40 | 5,92 | 7,45 | 0,74181759 | -1,53153 | 2,890922 |
| 20ERR8h3R3 | 3 | 6 | 3 µM 20E | 2 | 8 | 27,99941 | 0,074196 | 28,00 | 22,97 | 5,03 | 7,45 | 0,74181759 | -2,42134 | 5,356695 |
| SC8hR2 | 2 | 6 | 0.5% DMSO | 10 | NA | 29,34798 | 0,357525 | 29,35 | 22,64 | 6,71 | 7,45 | 0,74181759 |  |  |
| SC8hR3 | 3 | 6 | 0.5% DMSO | 10 | NA | 31,05207 | 2,479312 | 31,05 | 22,86 | 8,20 | 7,45 | 0,74181759 |  |  |

| sample id | replicate | experiment number | Treatment | exposure time/hours | recovery time/hours | Avg Cq | st.dev Cq | corrected Cq (max cq 35) | Refrence gene Cq | delta Cq | Avg. solvent control delta Cq | st.dev solvent control delta Cq | delta delta Cq | 2^ddCq |
| --- | --- | --- | --- | --- | --- | --- | --- | --- | --- | --- | --- | --- | --- | --- |
| PR1hA0.1R1 | 1 | 1 | 0.1 µM 20E | 1 | NA | 26,13 | 0,02089 | 28,86 | 23,08 | 5,78 | 7,12 | 0,505007 | 0,38 | 0,77 |
| PR1hA0.1R2 | 2 | 1 | 0.1 µM 20E | 1 | NA | 28,26 | 0,00129 | 28,10 | 22,41 | 5,68 | 7,12 | 0,505007 | 0,01 | 0,99 |
| PR1hA0.1R3 | 3 | 1 | 0.1 µM 20E | 1 | NA | 36,54 | 1,24381 | 30,97 | 23,03 | 7,95 | 7,12 | 0,505007 | -0,21 | 1,16 |
| PR1hB0.1R1 | 1 | 1 | 0.1 µM 20E | 1 | NA | 29,57 | 0,14238 | 30,45 | 23,53 | 6,92 | 7,12 | 0,505007 |  |  |
| PR1hB0.1R2 | 2 | 1 | 0.1 µM 20E | 1 | NA | 28,76 | 0,38295 | 27,67 | 22,44 | 5,23 | 7,12 | 0,505007 |  |  |
| PR1hB0.1R3 | 3 | 1 | 0.1 µM 20E | 1 | NA | 36,57 | 0,16753 | 29,98 | 22,64 | 7,34 | 7,12 | 0,505007 |  |  |
| PR1hCSR1 | 1 | 1 | 0.1 µM 20E | 1 | NA | 37,17 | 0,00000 | 29,77 | 23,32 | 6,45 | 7,12 | 0,505007 |  |  |
| PR1hCSR2 | 2 | 1 | 0.1 µM 20E | 1 | NA | 35,34 | 1,63820 | 28,81 | 22,37 | 6,44 | 7,12 | 0,505007 |  |  |
| PR1hCSR3 | 3 | 1 | 0.1 µM 20E | 1 | NA | 33,22 | 1,84902 | 31,24 | 22,81 | 8,43 | 7,12 | 0,505007 |  |  |
| PR2hA0.1R1 | 1 | 1 | 0.1 µM 20E | 2 | NA | 26,03 | 0,09343 | 30,19 | 23,67 | 6,52 | 7,12 | 0,505007 | -0,77 | 1,70 |
| PR2hA0.1R2 | 2 | 1 | 0.1 µM 20E | 2 | NA | 28,49 | 0,03464 | 27,88 | 22,77 | 5,10 | 7,12 | 0,505007 | -1,66 | 3,16 |
| PR2hA0.1R3 | 3 | 1 | 0.1 µM 20E | 2 | NA | 29,40 | 0,30653 | 29,20 | 22,45 | 6,75 | 7,12 | 0,505007 | 0,53 | 0,69 |
| PR2hB0.1R1 | 1 | 1 | 0.1 µM 20E | 2 | NA | 28,77 | 0,34167 | 30,84 | 23,98 | 6,87 | 7,12 | 0,505007 |  |  |
| PR2hB0.1R2 | 2 | 1 | 0.1 µM 20E | 2 | NA | 28,28 | 0,31111 | 27,96 | 21,82 | 6,13 | 7,12 | 0,505007 |  |  |
| PR2hB0.1R3 | 3 | 1 | 0.1 µM 20E | 2 | NA | 30,36 | 0,67995 | 28,73 | 22,32 | 6,42 | 7,12 | 0,505007 |  |  |
| PR2hCSR1 | 1 | 1 | 0.5% DMSO | 2 | NA | 34,87 | 1,04142 | 30,64 | 23,19 | 7,45 | 7,12 | 0,505007 |  |  |
| PR2hCSR2 | 2 | 1 | 0.5% DMSO | 2 | NA | 36,54 | 0,00000 | 28,69 | 22,41 | 6,28 | 7,12 | 0,505007 |  |  |
| PR2hCSR3 | 3 | 1 | 0.5% DMSO | 2 | NA | 33,75 | 0,04970 | 30,66 | 23,14 | 7,52 | 7,12 | 0,505007 |  |  |
| PR30minA0.1R1 | 1 | 1 | 0.1 µM 20E | 0,5 | NA | 26,71 | 0,26915 | 29,65 | 22,52 | 7,14 | 7,12 | 0,505007 | -0,43 | 1,34 |
| PR30minA0.1R2 | 2 | 1 | 0.1 µM 20E | 0,5 | NA | 30,46 | 0,05462 | 29,16 | 22,37 | 6,79 | 7,12 | 0,505007 | -1,50 | 2,83 |
| PR30minA0.1R3 | 3 | 1 | 0.1 µM 20E | 0,5 | NA | 36,15 | 0,00000 | 30,47 | 22,43 | 8,04 | 7,12 | 0,505007 | -0,53 | 1,45 |
| PR30minB0.1R1 | 1 | 1 | 0.1 µM 20E | 0,5 | NA | 30,70 | 0,50500 | 30,27 | 22,42 | 7,85 | 7,12 | 0,505007 |  |  |
| PR30minB0.1R2 | 2 | 1 | 0.1 µM 20E | 0,5 | NA | 30,88 | 0,16317 | 30,24 | 22,78 | 7,47 | 7,12 | 0,505007 |  |  |
| PR30minB0.1R3 | 3 | 1 | 0.1 µM 20E | 0,5 | NA | 35,39 | 0,00000 | 30,68 | 22,98 | 7,71 | 7,12 | 0,505007 |  |  |
| PR30minCSR1 | 1 | 1 | 0.5% DMSO | 0,5 | NA | 34,36 | 0,00000 | 30,19 | 23,12 | 7,07 | 7,12 | 0,505007 |  |  |
| PR30minCSR2 | 2 | 1 | 0.5% DMSO | 0,5 | NA | 35,25 | 0,99541 | 29,58 | 23,05 | 6,53 | 7,12 | 0,505007 |  |  |
| PR30minCSR3 | 3 | 1 | 0.5% DMSO | 0,5 | NA | 33,96 | 1,27104 | 30,50 | 22,64 | 7,86 | 7,12 | 0,505007 |  |  |
| R16hCSR1 | 1 | 1 | 0.5% DMSO | 18 | NA | 34,34 | 0,00000 | 29,13 | 22,28 | 6,85 | 7,12 | 0,505007 |  |  |
| R16hCSR2 | 2 | 1 | 0.5% DMSO | 18 | NA | 35,34 | 0,00000 | 29,74 | 23,12 | 6,62 | 7,12 | 0,505007 |  |  |
| R16hCSR3 | 3 | 1 | 0.5% DMSO | 18 | NA | 32,14 | 0,09990 | 29,38 | 22,49 | 6,89 | 7,12 | 0,505007 |  |  |
| R1hCSR1 | 1 | 1 | 0.5% DMSO | 3 | NA | 33,33 | 1,23050 | 29,64 | 23,00 | 6,64 | 7,12 | 0,505007 |  |  |
| R1hCSR2 | 2 | 1 | 0.5% DMSO | 3 | NA | 35,51 | 0,00000 | 29,43 | 22,53 | 6,90 | 7,12 | 0,505007 |  |  |
| R1hCSR3 | 3 | 1 | 0.5% DMSO | 3 | NA | 33,35 | 0,45068 | 30,65 | 23,79 | 6,86 | 7,12 | 0,505007 |  |  |
| R2hCSR1 | 1 | 1 | 0.5% DMSO | 4 | NA | 33,85 | 0,24336 | 30,22 | 22,58 | 7,65 | 7,12 | 0,505007 |  |  |
| R2hCSR2 | 2 | 1 | 0.5% DMSO | 4 | NA | 35,98 | 0,66578 | 29,49 | 22,43 | 7,06 | 7,12 | 0,505007 |  |  |
| R2hCSR3 | 3 | 1 | 0.5% DMSO | 4 | NA | 33,08 | 1,03187 | 30,40 | 23,29 | 7,12 | 7,12 | 0,505007 |  |  |
| R4hCSR1 | 1 | 1 | 0.5% DMSO | 6 | NA | 32,96 | 0,13681 | 30,53 | 22,95 | 7,58 | 7,12 | 0,505007 |  |  |
| R4hCSR2 | 2 | 1 | 0.5% DMSO | 6 | NA | 36,70 | 0,17962 | 29,66 | 22,48 | 7,18 | 7,12 | 0,505007 |  |  |
| R4hCSR3 | 3 | 1 | 0.5% DMSO | 6 | NA | 30,93 | 0,80456 | 30,57 | 23,30 | 7,27 | 7,12 | 0,505007 |  |  |
| R8hCSR1 | 1 | 1 | 0.5% DMSO | 10 | NA | 34,46 | 0,81036 | 30,39 | 23,22 | 7,16 | 7,12 | 0,505007 |  |  |
| R8hCSR2 | 2 | 1 | 0.5% DMSO | 10 | NA | 35,35 | 0,00000 | 29,85 | 22,55 | 7,29 | 7,12 | 0,505007 |  |  |
| R8hCSR3 | 3 | 1 | 0.5% DMSO | 10 | NA | 35,12 | 2,98357 | 30,86 | 23,13 | 7,72 | 7,12 | 0,505007 |  |  |
| RC16h0.1R1 | 1 | 1 | 0.1 µM 20E | 18 | NA | 29,08 | 0,14463 | 28,63 | 23,38 | 5,25 | 7,12 | 0,505007 | -1,86 | 3,64 |
| RC16h0.1R2 | 2 | 1 | 0.1 µM 20E | 18 | NA | 30,76 | 0,39342 | 28,57 | 22,99 | 5,58 | 7,12 | 0,505007 | -1,54 | 2,91 |
| RC16h0.1R3 | 3 | 1 | 0.1 µM 20E | 18 | NA | 28,04 | 0,03846 | 29,34 | 23,22 | 6,12 | 7,12 | 0,505007 | -1,00 | 2,00 |
| RC1h0.1R1 | 1 | 1 | 0.1 µM 20E | 3 | NA | 29,25 | 0,09986 | 29,04 | 23,28 | 5,76 | 7,12 | 0,505007 | -1,36 | 2,57 |
| RC1h0.1R2 | 2 | 1 | 0.1 µM 20E | 3 | NA | 30,06 | 0,33561 | 28,37 | 22,53 | 5,84 | 7,12 | 0,505007 | -1,27 | 2,42 |
| RC1h0.1R3 | 3 | 1 | 0.1 µM 20E | 3 | NA | 31,64 | 0,66578 | 30,17 | 23,38 | 6,79 | 7,12 | 0,505007 | -0,33 | 1,26 |
| RC2h0.1R1 | 1 | 1 | 0.1 µM 20E | 4 | NA | 28,92 | 0,60873 | 29,87 | 23,16 | 6,70 | 7,12 | 0,505007 | -0,41 | 1,33 |
| RC2h0.1R2 | 2 | 1 | 0.1 µM 20E | 4 | NA | 29,12 | 0,06975 | 28,73 | 23,06 | 5,67 | 7,12 | 0,505007 | -1,45 | 2,72 |
| RC2h0.1R3 | 3 | 1 | 0.1 µM 20E | 4 | NA | 28,84 | 0,65579 | 29,11 | 23,16 | 5,95 | 7,12 | 0,505007 | -1,16 | 2,24 |
| RC4h0.1R1 | 1 | 1 | 0.1 µM 20E | 6 | NA | 28,53 | 0,64253 | 29,71 | 23,13 | 6,57 | 7,12 | 0,505007 | -0,54 | 1,46 |
| RC4h0.1R2 | 2 | 1 | 0.1 µM 20E | 6 | NA | 29,73 | 0,00367 | 27,91 | 21,19 | 6,72 | 7,12 | 0,505007 | -0,40 | 1,32 |
| RC4h0.1R3 | 3 | 1 | 0.1 µM 20E | 6 | NA | 30,99 | 0,65219 | 29,10 | 22,42 | 6,68 | 7,12 | 0,505007 | -0,43 | 1,35 |
| RC8h0.1R1 | 1 | 1 | 0.1 µM 20E | 10 | NA | 29,12 | 0,49696 | 29,66 | 23,62 | 6,04 | 7,12 | 0,505007 | -1,08 | 2,11 |
| RC8h0.1R2 | 2 | 1 | 0.1 µM 20E | 10 | NA | 27,61 | 0,01646 | 28,93 | 23,12 | 5,81 | 7,12 | 0,505007 | -1,30 | 2,47 |
| RC8h0.1R3 | 3 | 1 | 0.1 µM 20E | 10 | NA | 31,02 | 0,91758 | 29,80 | 23,47 | 6,33 | 7,12 | 0,505007 | -0,79 | 1,73 |
| RR16h0.1R1 | 1 | 1 | 0.1 µM 20E | 2 | 16 | 34,70 | 0,28067 | 28,34 | 21,89 | 6,45 | 7,12 | 0,505007 | -0,67 | 1,59 |
| RR16h0.1R2 | 2 | 1 | 0.1 µM 20E | 2 | 16 | 36,68 | 1,40401 | 29,78 | 22,71 | 7,07 | 7,12 | 0,505007 | -0,05 | 1,03 |
| RR16h0.1R3 | 3 | 1 | 0.1 µM 20E | 2 | 16 | 33,38 | 0,15175 | 30,59 | 23,39 | 7,20 | 7,12 | 0,505007 | 0,09 | 0,94 |
| RR1h0.1R1 | 1 | 1 | 0.1 µM 20E | 2 | 1 | 29,93 | 0,14108 | 29,00 | 23,21 | 5,79 | 7,12 | 0,505007 | -1,33 | 2,51 |
| RR1h0.1R2 | 2 | 1 | 0.1 µM 20E | 2 | 1 | 31,39 | 0,02609 | 28,10 | 22,62 | 5,47 | 7,12 | 0,505007 | -1,64 | 3,13 |
| RR1h0.1R3 | 3 | 1 | 0.1 µM 20E | 2 | 1 | 33,06 | 0,00561 | 29,80 | 23,32 | 6,48 | 7,12 | 0,505007 | -0,64 | 1,56 |
| RR2h0.1R1 | 1 | 1 | 0.1 µM 20E | 2 | 2 | 29,19 | 0,22069 | 31,00 | 23,53 | 7,47 | 7,12 | 0,505007 | 0,35 | 0,78 |
| RR2h0.1R2 | 2 | 1 | 0.1 µM 20E | 2 | 2 | 32,43 | 0,06168 | 29,84 | 23,04 | 6,79 | 7,12 | 0,505007 | -0,32 | 1,25 |
| RR2h0.1R3 | 3 | 1 | 0.1 µM 20E | 2 | 2 | 32,55 | 0,25455 | 30,50 | 22,77 | 7,73 | 7,12 | 0,505007 | 0,61 | 0,66 |
| RR4h0.1R1 | 1 | 1 | 0.1 µM 20E | 2 | 4 | 30,83 | 0,52069 | 31,54 | 23,63 | 7,91 | 7,12 | 0,505007 | 0,79 | 0,58 |
| RR4h0.1R2 | 2 | 1 | 0.1 µM 20E | 2 | 4 | 34,34 | 0,54199 | 29,15 | 22,32 | 6,84 | 7,12 | 0,505007 | -0,28 | 1,22 |
| RR4h0.1R3 | 3 | 1 | 0.1 µM 20E | 2 | 4 | 34,31 | 1,14148 | 30,38 | 21,55 | 8,82 | 7,12 | 0,505007 | 1,71 | 0,31 |
| RR8h0.1R1 | 1 | 1 | 0.1 µM 20E | 2 | 8 | 30,80 | 0,96806 | 30,83 | 23,33 | 7,50 | 7,12 | 0,505007 | 0,39 | 0,76 |
| RR8h0.1R2 | 2 | 1 | 0.1 µM 20E | 2 | 8 | 33,88 | 0,36599 | 30,14 | 23,17 | 6,96 | 7,12 | 0,505007 | -0,15 | 1,11 |
| RR8h0.1R3 | 3 | 1 | 0.1 µM 20E | 2 | 8 | 35,46 | 0,00000 | 31,14 | 21,18 | 9,96 | 7,12 | 0,505007 | 2,84 | 0,14 |
| PR1h0.1R1 | 1 | 2 | 0.1 µM 20E | 1 | NA | 28,49653 | 0,031826 | 28,50 | 22,27 | 6,23 | 7,39 | 0,663493 | -1,17 | 2,24 |
| PR1h0.1R2 | 2 | 2 | 0.1 µM 20E | 1 | NA | 27,73826 | 0,607422 | 27,74 | 22,09 | 5,65 | 7,39 | 0,663493 | -1,74 | 3,34 |
| PR1hCSR1 | 1 | 2 | 0.1 µM 20E | 1 | NA | 29,65516 | 0,126953 | 29,66 | 22,01 | 7,64 | 7,39 | 0,663493 |  |  |
| PR1hCSR2 | 2 | 2 | 0.1 µM 20E | 1 | NA | 30,2554 | 0,106873 | 30,26 | 22,33 | 7,92 | 7,39 | 0,663493 |  |  |
| PR2h0.1R1 | 1 | 2 | 0.1 µM 20E | 2 | NA | 27,34431 | 0,157515 | 27,34 | 21,82 | 5,53 | 7,39 | 0,663493 | -1,87 | 3,65 |
| PR2h0.1R2 | 2 | 2 | 0.1 µM 20E | 2 | NA | 27,94432 | 0,10311 | 27,94 | 21,66 | 6,29 | 7,39 | 0,663493 | -1,10 | 2,15 |
| PR2hCSR1 | 1 | 2 | 0.5% DMSO | 2 | NA | 29,2257 | 0,131632 | 29,23 | 22,09 | 7,13 | 7,39 | 0,663493 |  |  |
| PR2hCSR2 | 2 | 2 | 0.5% DMSO | 2 | NA | 29,99744 | 0,074905 | 30,00 | 22,06 | 7,94 | 7,39 | 0,663493 |  |  |
| PR30min0.1R1 | 1 | 2 | 0.1 µM 20E | 0,5 | NA | 28,97102 | 0,331973 | 28,97 | 23,06 | 5,91 | 7,39 | 0,663493 | -1,48 | 2,79 |
| PR30min0.1R2 | 2 | 2 | 0.1 µM 20E | 0,5 | NA | 28,60594 | 0,149343 | 28,61 | 22,21 | 6,40 | 7,39 | 0,663493 | -1,00 | 1,99 |
| PR30minCSR1 | 1 | 2 | 0.5% DMSO | 0,5 | NA | 28,9716 | 0,200959 | 28,97 | 21,97 | 7,00 | 7,39 | 0,663493 |  |  |
| PR30minCSR2 | 2 | 2 | 0.5% DMSO | 0,5 | NA | 30,88593 | 0,136763 | 30,89 | 22,57 | 8,32 | 7,39 | 0,663493 |  |  |
| R16hCSR1 | 1 | 2 | 0.5% DMSO | 18 | NA | 31,63858 | 0,003147 | 31,64 | 22,90 | 8,74 | 7,39 | 0,663493 |  |  |
| R16hCSR2 | 2 | 2 | 0.5% DMSO | 18 | NA | 30,60436 | 0 | 30,60 | 22,21 | 8,40 | 7,39 | 0,663493 |  |  |
| R1hCSR1 | 1 | 2 | 0.5% DMSO | 3 | NA | 29,47806 | 0 | 29,48 | 23,14 | 6,33 | 7,39 | 0,663493 |  |  |
| R1hCSR2 | 2 | 2 | 0.5% DMSO | 3 | NA | 29,23142 | 0,228146 | 29,23 | 22,39 | 6,84 | 7,39 | 0,663493 |  |  |
| R2hCSR1 | 1 | 2 | 0.5% DMSO | 4 | NA | 29,29899 | 0 | 29,30 | 22,43 | 6,87 | 7,39 | 0,663493 |  |  |
| R2hCSR2 | 2 | 2 | 0.5% DMSO | 4 | NA | 28,69864 | 0,194606 | 28,70 | 22,18 | 6,51 | 7,39 | 0,663493 |  |  |
| R4hCSR1 | 1 | 2 | 0.5% DMSO | 6 | NA | 29,60152 | 0 | 29,60 | 22,44 | 7,16 | 7,39 | 0,663493 |  |  |
| R4hCSR2 | 2 | 2 | 0.5% DMSO | 6 | NA | 29,09992 | 0 | 29,10 | 22,27 | 6,83 | 7,39 | 0,663493 |  |  |
| R8hCSR1 | 1 | 2 | 0.5% DMSO | 10 | NA | 30,18712 | 0,123963 | 30,19 | 22,66 | 7,53 | 7,39 | 0,663493 |  |  |
| R8hCSR2 | 2 | 2 | 0.5% DMSO | 10 | NA | 29,7339 | 0 | 29,73 | 22,63 | 7,11 | 7,39 | 0,663493 |  |  |
| RC16h0.1R1 | 1 | 2 | 0.1 µM 20E | 18 | NA | 27,52816 | 1,241906 | 27,53 | 22,14 | 5,39 | 7,39 | 0,663493 | -2,00 | 4,01 |
| RC16h0.1R2 | 2 | 2 | 0.1 µM 20E | 18 | NA | 28,06605 | 0,431801 | 28,07 | 22,13 | 5,94 | 7,39 | 0,663493 | -1,46 | 2,74 |
| RC1h0.1R1 | 1 | 2 | 0.1 µM 20E | 3 | NA | 27,49268 | 0,060869 | 27,49 | 22,31 | 5,18 | 7,39 | 0,663493 | -2,21 | 4,62 |
| RC1h0.1R2 | 2 | 2 | 0.1 µM 20E | 3 | NA | 27,92714 | 0,124123 | 27,93 | 22,58 | 5,35 | 7,39 | 0,663493 | -2,04 | 4,12 |
| RC2h0.1R1 | 1 | 2 | 0.1 µM 20E | 4 | NA | 26,88795 | 1,054205 | 26,89 | 22,23 | 4,66 | 7,39 | 0,663493 | -2,74 | 6,67 |
| RC2h0.1R2 | 2 | 2 | 0.1 µM 20E | 4 | NA | 27,42652 | 0,388816 | 27,43 | 22,70 | 4,73 | 7,39 | 0,663493 | -2,67 | 6,35 |
| RC4h0.1R1 | 1 | 2 | 0.1 µM 20E | 6 | NA | 26,71121 | 0,579694 | 26,71 | 22,21 | 4,51 | 7,39 | 0,663493 | -2,89 | 7,40 |
| RC4h0.1R2 | 2 | 2 | 0.1 µM 20E | 6 | NA | 27,64626 | 0,220466 | 27,65 | 22,33 | 5,32 | 7,39 | 0,663493 | -2,07 | 4,21 |
| RC8h0.1R1 | 1 | 2 | 0.1 µM 20E | 10 | NA | 26,83141 | 0,381816 | 26,83 | 22,22 | 4,61 | 7,39 | 0,663493 | -2,78 | 6,89 |
| RC8h0.1R2 | 2 | 2 | 0.1 µM 20E | 10 | NA | 27,00148 | 0,026664 | 27,00 | 22,50 | 4,50 | 7,39 | 0,663493 | -2,89 | 7,42 |
| RR16h0.1R1 | 1 | 2 | 0.1 µM 20E | 2 | 16 | 30,45512 | 0,077696 | 30,46 | 22,06 | 8,39 | 7,39 | 0,663493 | 1,00 | 0,50 |
| RR16h0.1R2 | 2 | 2 | 0.1 µM 20E | 2 | 16 | 28,51736 | 0,051508 | 28,52 | 21,29 | 7,22 | 7,39 | 0,663493 | -0,17 | 1,12 |
| RR1h0.1R1 | 1 | 2 | 0.1 µM 20E | 2 | 1 |  | 0 |  | 24,69 |  | 7,39 | 0,663493 |  |  |
| RR1h0.1R2 | 2 | 2 | 0.1 µM 20E | 2 | 1 | 27,89432 | 0,200559 | 27,89 | 22,55 | 5,34 | 7,39 | 0,663493 | -2,05 | 4,14 |
| RR2h0.1R1 | 1 | 2 | 0.1 µM 20E | 2 | 2 | 29,00082 | 0,209781 | 29,00 | 23,24 | 5,76 | 7,39 | 0,663493 | -1,64 | 3,11 |
| RR2h0.1R2 | 2 | 2 | 0.1 µM 20E | 2 | 2 | 28,44245 | 0,200977 | 28,44 | 22,25 | 6,19 | 7,39 | 0,663493 | -1,20 | 2,30 |
| RR4h0.1R1 | 1 | 2 | 0.1 µM 20E | 2 | 4 | 29,42856 | 0,17982 | 29,43 | 22,47 | 6,96 | 7,39 | 0,663493 | -0,44 | 1,35 |
| RR4h0.1R2 | 2 | 2 | 0.1 µM 20E | 2 | 4 | 28,54596 | 0,217867 | 28,55 | 22,09 | 6,46 | 7,39 | 0,663493 | -0,94 | 1,91 |
| RR8h0.1R1 | 1 | 2 | 0.1 µM 20E | 2 | 8 | 29,41399 | 0,143545 | 29,41 | 22,92 | 6,49 | 7,39 | 0,663493 | -0,90 | 1,86 |
| RR8h0.1R2 | 2 | 2 | 0.1 µM 20E | 2 | 8 | 27,97233 | 0,941291 | 27,97 | 22,18 | 5,79 | 7,39 | 0,663493 | -1,60 | 3,04 |
| PR1h0.1R1 | 1 | 3 | 0.1 µM 20E | 1 | NA | 28,73134 | 0,399167 | 28,73 | 21,66 | 7,07 | 7,43 | 1,237456 | -0,36 | 1,28 |
| PR1h0.1R2 | 2 | 3 | 0.1 µM 20E | 1 | NA | 28,3156 | 0,014839 | 28,32 | 22,08 | 6,24 | 7,43 | 1,237456 | -1,19 | 2,28 |
| PR1hCSR1 | 1 | 3 | 0.1 µM 20E | 1 | NA | 27,56954 | 0,423204 | 27,57 | 21,26 | 6,31 | 7,43 | 1,237456 |  |  |
| PR1hCSR2 | 2 | 3 | 0.1 µM 20E | 1 | NA | 29,88715 | 0,519252 | 29,89 | 21,94 | 7,95 | 7,43 | 1,237456 |  |  |
| PR2h0.1R1 | 1 | 3 | 0.1 µM 20E | 2 | NA | 30,28522 | 0,429442 | 30,29 | 22,27 | 8,02 | 7,43 | 1,237456 | 0,59 | 0,66 |
| PR2h0.1R2 | 2 | 3 | 0.1 µM 20E | 2 | NA | 29,49331 | 0,280718 | 29,49 | 22,90 | 6,59 | 7,43 | 1,237456 | -0,84 | 1,79 |
| PR2hCSR1 | 1 | 3 | 0.5% DMSO | 2 | NA | 30,71201 | 0,174622 | 30,71 | 21,14 | 9,57 | 7,43 | 1,237456 |  |  |
| PR2hCSR2 | 2 | 3 | 0.5% DMSO | 2 | NA | 30,47836 | 0,304836 | 30,48 | 22,37 | 8,11 | 7,43 | 1,237456 |  |  |
| PR30min0.1R1 | 1 | 3 | 0.1 µM 20E | 0,5 | NA | 29,47081 | 0,423953 | 29,47 | 22,13 | 7,34 | 7,43 | 1,237456 | -0,09 | 1,07 |
| PR30min0.1R2 | 2 | 3 | 0.1 µM 20E | 0,5 | NA | 30,72274 | 0,143232 | 30,72 | 22,43 | 8,29 | 7,43 | 1,237456 | 0,86 | 0,55 |
| PR30minCSR1 | 1 | 3 | 0.5% DMSO | 0,5 | NA | 30,73166 | 0,257581 | 30,73 | 22,05 | 8,68 | 7,43 | 1,237456 |  |  |
| PR30minCSR2 | 2 | 3 | 0.5% DMSO | 0,5 | NA | 30,73084 | 1,428994 | 30,73 | 21,65 | 9,08 | 7,43 | 1,237456 |  |  |
| R16hCSR1 | 1 | 3 | 0.5% DMSO | 18 | NA | 34,11978 | 2,27453 | 34,12 | 24,97 | 9,15 | 7,43 | 1,237456 |  |  |
| R16hCSR2 | 2 | 3 | 0.5% DMSO | 18 | NA | 32,73558 | 0,540654 | 32,74 | 24,64 | 8,09 | 7,43 | 1,237456 |  |  |
| R1hCSR1 | 1 | 3 | 0.5% DMSO | 3 | NA | 32,83443 | 0,745648 | 32,83 | 26,16 | 6,68 | 7,43 | 1,237456 |  |  |
| R1hCSR2 | 2 | 3 | 0.5% DMSO | 3 | NA | 30,157 | 0,237944 | 30,16 | 24,71 | 5,44 | 7,43 | 1,237456 |  |  |
| R2hCSR1 | 1 | 3 | 0.5% DMSO | 4 | NA | 32,20353 | 0,748715 | 32,20 | 25,86 | 6,35 | 7,43 | 1,237456 |  |  |
| R2hCSR2 | 2 | 3 | 0.5% DMSO | 4 | NA | 30,98266 | 0,477389 | 30,98 | 25,37 | 5,62 | 7,43 | 1,237456 |  |  |
| R4hCSR1 | 1 | 3 | 0.5% DMSO | 6 | NA | 33,72006 | 1,957294 | 33,72 | 26,51 | 7,21 | 7,43 | 1,237456 |  |  |
| R4hCSR2 | 2 | 3 | 0.5% DMSO | 6 | NA | 30,06169 | 0,155687 | 30,06 | 23,63 | 6,43 | 7,43 | 1,237456 |  |  |
| R8hCSR1 | 1 | 3 | 0.5% DMSO | 10 | NA | 32,87206 | 0,859258 | 32,87 | 25,82 | 7,05 | 7,43 | 1,237456 |  |  |
| R8hCSR2 | 2 | 3 | 0.5% DMSO | 10 | NA | 30,67591 | 0,531338 | 30,68 | 23,53 | 7,14 | 7,43 | 1,237456 |  |  |
| RC16h0.1R1 | 1 | 3 | 0.1 µM 20E | 18 | NA | 29,67451 | 0,595364 | 29,67 | 22,76 | 6,92 | 7,43 | 1,237456 | -0,51 | 1,42 |
| RC16h0.1R2 | 2 | 3 | 0.1 µM 20E | 18 | NA | 30,01181 | 0,615038 | 30,01 | 22,70 | 7,31 | 7,43 | 1,237456 | -0,12 | 1,09 |
| RC1h0.1R1 | 1 | 3 | 0.1 µM 20E | 3 | NA | 32,5558 | 0,586548 | 32,56 | 24,27 | 8,28 | 7,43 | 1,237456 | 0,85 | 0,55 |
| RC1h0.1R2 | 2 | 3 | 0.1 µM 20E | 3 | NA | 31,04044 | 0,830609 | 31,04 | 24,45 | 6,59 | 7,43 | 1,237456 | -0,84 | 1,79 |
| RC2h0.1R1 | 1 | 3 | 0.1 µM 20E | 4 | NA | 30,54729 | 1,336774 | 30,55 | 23,46 | 7,09 | 7,43 | 1,237456 | -0,34 | 1,27 |
| RC2h0.1R2 | 2 | 3 | 0.1 µM 20E | 4 | NA | 30,86899 | 0,870379 | 30,87 | 23,47 | 7,40 | 7,43 | 1,237456 | -0,03 | 1,02 |
| RC4h0.1R1 | 1 | 3 | 0.1 µM 20E | 6 | NA | 29,74282 | 0,327846 | 29,74 | 23,81 | 5,93 | 7,43 | 1,237456 | -1,50 | 2,83 |
| RC4h0.1R2 | 2 | 3 | 0.1 µM 20E | 6 | NA | 30,66656 | 0,91492 | 30,67 | 24,12 | 6,55 | 7,43 | 1,237456 | -0,88 | 1,84 |
| RC8h0.1R1 | 1 | 3 | 0.1 µM 20E | 10 | NA | 29,79022 | 0,975228 | 29,79 | 22,51 | 7,28 | 7,43 | 1,237456 | -0,15 | 1,11 |
| RC8h0.1R2 | 2 | 3 | 0.1 µM 20E | 10 | NA | 29,53232 | 0,885194 | 29,53 | 23,33 | 6,20 | 7,43 | 1,237456 | -1,23 | 2,34 |
| RR16h0.1R1 | 1 | 3 | 0.1 µM 20E | 2 | 16 | 30,43325 | 0,165092 | 30,43 | 22,20 | 8,23 | 7,43 | 1,237456 | 0,80 | 0,57 |
| RR16h0.1R2 | 2 | 3 | 0.1 µM 20E | 2 | 16 | 30,76443 | 0,357366 | 30,76 | 21,39 | 9,37 | 7,43 | 1,237456 | 1,94 | 0,26 |
| RR1h0.1R1 | 1 | 3 | 0.1 µM 20E | 2 | 1 | 30,78475 | 0,249023 | 30,78 | 23,37 | 7,42 | 7,43 | 1,237456 | -0,01 | 1,01 |
| RR1h0.1R2 | 2 | 3 | 0.1 µM 20E | 2 | 1 | 30,39132 | 0,074311 | 30,39 | 23,64 | 6,75 | 7,43 | 1,237456 | -0,68 | 1,60 |
| RR2h0.1R1 | 1 | 3 | 0.1 µM 20E | 2 | 2 | 31,3511 | 0,30925 | 31,35 | 23,24 | 8,11 | 7,43 | 1,237456 | 0,68 | 0,62 |
| RR2h0.1R2 | 2 | 3 | 0.1 µM 20E | 2 | 2 | 30,37757 | 0,136324 | 30,38 | 22,70 | 7,68 | 7,43 | 1,237456 | 0,25 | 0,84 |
| RR4h0.1R1 | 1 | 3 | 0.1 µM 20E | 2 | 4 | 31,56363 | 0,132794 | 31,56 | 23,41 | 8,16 | 7,43 | 1,237456 | 0,73 | 0,60 |
| RR4h0.1R2 | 2 | 3 | 0.1 µM 20E | 2 | 4 | 31,34439 | 0,139521 | 31,34 | 23,58 | 7,76 | 7,43 | 1,237456 | 0,33 | 0,79 |
| RR8h0.1R1 | 1 | 3 | 0.1 µM 20E | 2 | 8 | 30,26891 | 0,217073 | 30,27 | 22,76 | 7,51 | 7,43 | 1,237456 | 0,08 | 0,94 |
| RR8h0.1R2 | 2 | 3 | 0.1 µM 20E | 2 | 8 | 31,13284 | 0,566719 | 31,13 | 22,54 | 8,59 | 7,43 | 1,237456 | 1,16 | 0,45 |
| 20ERC8h0.003R1 | 1 | 5 | 0.003 µM 20E | 10 | NA | 29,56902 | 0,36288 | 29,57 | 23,60 | 5,97 | 7,54 | 0,162676 | -1,58 | 2,98 |
| 20ERC8h0.003R2 | 2 | 5 | 0.003 µM 20E | 10 | NA | 29,57854 | 0,012656 | 29,58 | 23,18 | 6,40 | 7,54 | 0,162676 | -1,14 | 2,21 |
| 20ERC8h0.003R3 | 3 | 5 | 0.003 µM 20E | 10 | NA | 31,81654 | 0,142302 | 31,82 | 24,35 | 7,47 | 7,54 | 0,162676 | -0,08 | 1,06 |
| 20ERC8h0.01R1 | 1 | 5 | 0.01 µM 20E | 10 | NA | 29,83733 | 0,25238 | 29,84 | 22,42 | 7,41 | 7,54 | 0,162676 | -0,13 | 1,09 |
| 20ERC8h0.01R2 | 2 | 5 | 0.01 µM 20E | 10 | NA | 30,10012 | 0,033828 | 30,10 | 22,93 | 7,17 | 7,54 | 0,162676 | -0,37 | 1,29 |
| 20ERC8h0.01R3 | 3 | 5 | 0.01 µM 20E | 10 | NA | 29,29629 | 0,006969 | 29,30 | 22,95 | 6,34 | 7,54 | 0,162676 | -1,20 | 2,30 |
| 20ERC8h0.03R1 | 1 | 5 | 0.03 µM 20E | 10 | NA | 27,30782 | 0,196682 | 27,31 | 22,03 | 5,28 | 7,54 | 0,162676 | -2,27 | 4,81 |
| 20ERC8h0.03R2 | 2 | 5 | 0.03 µM 20E | 10 | NA | 28,41371 | 0,015358 | 28,41 | 22,17 | 6,24 | 7,54 | 0,162676 | -1,30 | 2,47 |
| 20ERC8h0.03R3 | 3 | 5 | 0.03 µM 20E | 10 | NA | 28,78568 | 0,064636 | 28,79 | 23,13 | 5,65 | 7,54 | 0,162676 | -1,89 | 3,71 |
| 20ERC8h0.1R1 | 1 | 5 | 0.1 µM 20E | 10 | NA | 28,09415 | 0,092839 | 28,09 | 22,27 | 5,83 | 7,54 | 0,162676 | -1,72 | 3,29 |
| 20ERC8h0.1R2 | 2 | 5 | 0.1 µM 20E | 10 | NA | 28,43811 | 0,037711 | 28,44 | 23,17 | 5,27 | 7,54 | 0,162676 | -2,28 | 4,84 |
| 20ERC8h0.1R3 | 3 | 5 | 0.1 µM 20E | 10 | NA | 29,1691 | 0,09585 | 29,17 | 23,08 | 6,09 | 7,54 | 0,162676 | -1,46 | 2,75 |
| 20ERC8h0.3R1 | 1 | 5 | 0.3 µM 20E | 10 | NA | 28,18473 | 0,382847 | 28,18 | 22,53 | 5,66 | 7,54 | 0,162676 | -1,89 | 3,70 |
| 20ERC8h0.3R2 | 2 | 5 | 0.3 µM 20E | 10 | NA | 27,73985 | 0,240859 | 27,74 | 22,47 | 5,27 | 7,54 | 0,162676 | -2,27 | 4,83 |
| 20ERC8h0.3R3 | 3 | 5 | 0.3 µM 20E | 10 | NA |  | 0 |  |  |  | 7,54 | 0,162676 |  |  |
| 20ERC8h1R1 | 1 | 5 | 1 µM 20E | 10 | NA | 29,60791 | 0,232592 | 29,61 | 21,96 | 7,65 | 7,54 | 0,162676 | 0,10 | 0,93 |
| 20ERC8h1R2 | 2 | 5 | 1 µM 20E | 10 | NA | 30,78174 | 0,460775 | 30,78 | 25,05 | 5,73 | 7,54 | 0,162676 | -1,81 | 3,51 |
| 20ERC8h1R3 | 3 | 5 | 1 µM 20E | 10 | NA | 31,03348 | 0,764399 | 31,03 | 25,69 | 5,34 | 7,54 | 0,162676 | -2,20 | 4,60 |
| 20ERC8h3R1 | 1 | 5 | 3 µM 20E | 10 | NA | 29,09858 | 0,127007 | 29,10 | 23,89 | 5,21 | 7,54 | 0,162676 | -2,33 | 5,04 |
| 20ERC8h3R2 | 2 | 5 | 3 µM 20E | 10 | NA | 29,36087 | 0,054143 | 29,36 | 22,99 | 6,37 | 7,54 | 0,162676 | -1,18 | 2,26 |
| 20ERC8h3R3 | 3 | 5 | 3 µM 20E | 10 | NA | 29,57165 | 0,068629 | 29,57 | 23,01 | 6,56 | 7,54 | 0,162676 | -0,98 | 1,98 |
| 20ERR8h0.003R1 | 1 | 5 | 0.003 µM 20E | 2 | 8 | 29,42475 | 0,021164 | 29,42 | 22,12 | 7,31 | 7,54 | 0,162676 | -0,24 | 1,18 |
| 20ERR8h0.003R2 | 2 | 5 | 0.003 µM 20E | 2 | 8 | 29,86388 | 0,173294 | 29,86 | 21,83 | 8,04 | 7,54 | 0,162676 | 0,49 | 0,71 |
| 20ERR8h0.003R3 | 3 | 5 | 0.003 µM 20E | 2 | 8 | 29,47358 | 0,090583 | 29,47 | 22,43 | 7,05 | 7,54 | 0,162676 | -0,50 | 1,41 |
| 20ERR8h0.01R1 | 1 | 5 | 0.01 µM 20E | 2 | 8 | 30,05644 | 0,070881 | 30,06 | 22,58 | 7,47 | 7,54 | 0,162676 | -0,07 | 1,05 |
| 20ERR8h0.01R2 | 2 | 5 | 0.01 µM 20E | 2 | 8 | 30,24904 | 0,174251 | 30,25 | 22,19 | 8,06 | 7,54 | 0,162676 | 0,51 | 0,70 |
| 20ERR8h0.01R3 | 3 | 5 | 0.01 µM 20E | 2 | 8 | 36,25953 | 1,253798 | 35,00 | 25,02 | 9,98 | 7,54 | 0,162676 | -0,68 | 1,60 |
| 20ERR8h0.03R1 | 1 | 5 | 0.03 µM 20E | 2 | 8 | 29,42212 | 0,041583 | 29,42 | 22,28 | 7,15 | 7,54 | 0,162676 | -0,40 | 1,32 |
| 20ERR8h0.03R2 | 2 | 5 | 0.03 µM 20E | 2 | 8 | 28,20106 | 0,380981 | 28,20 | 21,48 | 6,72 | 7,54 | 0,162676 | -0,83 | 1,77 |
| 20ERR8h0.03R3 | 3 | 5 | 0.03 µM 20E | 2 | 8 | 29,55141 | 0,108878 | 29,55 | 22,16 | 7,39 | 7,54 | 0,162676 | -0,15 | 1,11 |
| 20ERR8h0.1R1 | 1 | 5 | 0.1 µM 20E | 2 | 8 | 28,98055 | 0,241378 | 28,98 | 21,93 | 7,05 | 7,54 | 0,162676 | -0,50 | 1,41 |
| 20ERR8h0.1R2 | 2 | 5 | 0.1 µM 20E | 2 | 8 | 29,7969 | 0,12963 | 29,80 | 22,35 | 7,45 | 7,54 | 0,162676 | -0,09 | 1,07 |
| 20ERR8h0.1R3 | 3 | 5 | 0.1 µM 20E | 2 | 8 | 29,26851 | 0,792834 | 29,27 | 22,40 | 6,86 | 7,54 | 0,162676 | -0,68 | 1,60 |
| 20ERR8h0.3R1 | 1 | 5 | 0.3 µM 20E | 2 | 8 | 29,26976 | 0,021422 | 29,27 | 21,93 | 7,34 | 7,54 | 0,162676 | -0,20 | 1,15 |
| 20ERR8h0.3R2 | 2 | 5 | 0.3 µM 20E | 2 | 8 | 29,24134 | 0,232188 | 29,24 | 21,63 | 7,61 | 7,54 | 0,162676 | 0,07 | 0,95 |
| 20ERR8h0.3R3 | 3 | 5 | 0.3 µM 20E | 2 | 8 | 33,5878 | 1,195005 | 33,59 | 23,92 | 9,66 | 7,54 | 0,162676 | 2,12 | 0,23 |
| 20ERR8h1R1 | 1 | 5 | 1 µM 20E | 2 | 8 | 28,23113 | 0,018511 | 28,23 | 21,35 | 6,88 | 7,54 | 0,162676 | -0,66 | 1,58 |
| 20ERR8h1R2 | 2 | 5 | 1 µM 20E | 2 | 8 | 28,22647 | 0,103816 | 28,23 | 21,63 | 6,60 | 7,54 | 0,162676 | -0,95 | 1,93 |
| 20ERR8h1R3 | 3 | 5 | 1 µM 20E | 2 | 8 | 28,22388 | 0,355474 | 28,22 | 22,30 | 5,93 | 7,54 | 0,162676 | -1,62 | 3,07 |
| 20ERR8h3R1 | 1 | 5 | 3 µM 20E | 2 | 8 | 30,97217 | 0,683076 | 30,97 | 20,99 | 9,98 | 7,54 | 0,162676 | 2,43 | 0,18 |
| 20ERR8h3R2 | 2 | 5 | 3 µM 20E | 2 | 8 | 27,79672 | 0,066201 | 27,80 | 21,79 | 6,00 | 7,54 | 0,162676 | -1,54 | 2,91 |
| 20ERR8h3R3 | 3 | 5 | 3 µM 20E | 2 | 8 | 28,35434 | 0,234837 | 28,35 | 22,24 | 6,11 | 7,54 | 0,162676 | -1,43 | 2,70 |
| SC8hR1 | 1 | 5 | 0.5% DMSO | 10 | NA | 29,45015 | 0,107871 | 29,45 | 22,01 | 7,44 | 7,54 | 0,162676 |  |  |
| SC8hR2 | 2 | 5 | 0.5% DMSO | 10 | NA | 29,32417 | 0,098501 | 29,32 | 21,90 | 7,42 | 7,54 | 0,162676 |  |  |
| SC8hR3 | 3 | 5 | 0.5% DMSO | 10 | NA | 30,44458 | 0,016024 | 30,44 | 22,67 | 7,77 | 7,54 | 0,162676 |  |  |

| sample id | replicate | experiment number | Treatment | exposure time/hours | recovery time/hours | Avg Cq | st.dev Cq | corrected Cq (max cq 35) | Refrence gene Cq | delta Cq | Avg. solvent control delta Cq | st.dev solvent control delta Cq | delta delta Cq | 2^ddCq |
| --- | --- | --- | --- | --- | --- | --- | --- | --- | --- | --- | --- | --- | --- | --- |
| PR1hA0.1R1 | 1 | 1 | 0.1 µM 20E | 1 | NA | 26,13 | 0,02089 | 35,00 | 23,90 | 11,10 | 10,64 | 0,55 | 0,50 | 0,71 |
| PR1hA0.1R2 | 2 | 1 | 0.1 µM 20E | 1 | NA | 28,26 | 0,00129 | 34,68 | 23,37 | 11,32 | 10,64 | 0,55 | -0,15 | 1,11 |
| PR1hA0.1R3 | 3 | 1 | 0.1 µM 20E | 1 | NA | 36,54 | 1,24381 | 35,00 | 24,43 | 10,57 | 10,64 | 0,55 | 0,17 | 0,89 |
| PR1hB0.1R1 | 1 | 1 | 0.1 µM 20E | 1 | NA | 29,57 | 0,14238 | 34,22 | 24,40 | 9,82 | 10,64 | 0,55 |  |  |
| PR1hB0.1R2 | 2 | 1 | 0.1 µM 20E | 1 | NA | 28,76 | 0,38295 | 34,01 | 23,69 | 10,32 | 10,64 | 0,55 |  |  |
| PR1hB0.1R3 | 3 | 1 | 0.1 µM 20E | 1 | NA | 36,57 | 0,16753 | 34,49 | 23,92 | 10,57 | 10,64 | 0,55 |  |  |
| PR1hCSR1 | 1 | 1 | 0.1 µM 20E | 1 | NA | 37,17 | 0,00000 | 34,56 | 24,60 | 9,97 | 10,64 | 0,55 |  |  |
| PR1hCSR2 | 2 | 1 | 0.1 µM 20E | 1 | NA | 35,34 | 1,63820 | 34,07 | 23,46 | 10,61 | 10,64 | 0,55 |  |  |
| PR1hCSR3 | 3 | 1 | 0.1 µM 20E | 1 | NA | 33,22 | 1,84902 | 35,00 | 23,93 | 11,07 | 10,64 | 0,55 |  |  |
| PR2hA0.1R1 | 1 | 1 | 0.1 µM 20E | 2 | NA | 26,03 | 0,09343 | 35,00 | 24,75 | 10,25 | 10,64 | 0,55 | -0,07 | 1,05 |
| PR2hA0.1R2 | 2 | 1 | 0.1 µM 20E | 2 | NA | 28,49 | 0,03464 | 33,26 | 23,95 | 9,31 | 10,64 | 0,55 | 0,29 | 0,82 |
| PR2hA0.1R3 | 3 | 1 | 0.1 µM 20E | 2 | NA | 29,40 | 0,30653 | 35,00 | 24,52 | 10,48 | 10,64 | 0,55 | 0,04 | 0,98 |
| PR2hB0.1R1 | 1 | 1 | 0.1 µM 20E | 2 | NA | 28,77 | 0,34167 | 35,00 | 24,87 | 10,13 | 10,64 | 0,55 |  |  |
| PR2hB0.1R2 | 2 | 1 | 0.1 µM 20E | 2 | NA | 28,28 | 0,31111 | 33,96 | 24,29 | 9,67 | 10,64 | 0,55 |  |  |
| PR2hB0.1R3 | 3 | 1 | 0.1 µM 20E | 2 | NA | 30,36 | 0,67995 | 34,95 | 24,21 | 10,74 | 10,64 | 0,55 |  |  |
| PR2hCSR1 | 1 | 1 | 0.5% DMSO | 2 | NA | 34,87 | 1,04142 | 34,37 | 24,18 | 10,19 | 10,64 | 0,55 |  |  |
| PR2hCSR2 | 2 | 1 | 0.5% DMSO | 2 | NA | 36,54 | 0,00000 | 34,95 | 24,11 | 10,85 | 10,64 | 0,55 |  |  |
| PR2hCSR3 | 3 | 1 | 0.5% DMSO | 2 | NA | 33,75 | 0,04970 | 35,00 | 24,17 | 10,83 | 10,64 | 0,55 |  |  |
| PR30minA0.1R1 | 1 | 1 | 0.1 µM 20E | 0,5 | NA | 26,71 | 0,26915 | 35,00 | 23,80 | 11,20 | 10,64 | 0,55 | -0,34 | 1,27 |
| PR30minA0.1R2 | 2 | 1 | 0.1 µM 20E | 0,5 | NA | 30,46 | 0,05462 | 34,08 | 23,53 | 10,55 | 10,64 | 0,55 | -1,04 | 2,05 |
| PR30minA0.1R3 | 3 | 1 | 0.1 µM 20E | 0,5 | NA | 36,15 | 0,00000 | 33,75 | 23,46 | 10,30 | 10,64 | 0,55 | 0,08 | 0,95 |
| PR30minB0.1R1 | 1 | 1 | 0.1 µM 20E | 0,5 | NA | 30,70 | 0,50500 | 34,37 | 23,51 | 10,86 | 10,64 | 0,55 |  |  |
| PR30minB0.1R2 | 2 | 1 | 0.1 µM 20E | 0,5 | NA | 30,88 | 0,16317 | 34,06 | 23,85 | 10,21 | 10,64 | 0,55 |  |  |
| PR30minB0.1R3 | 3 | 1 | 0.1 µM 20E | 0,5 | NA | 35,39 | 0,00000 | 35,00 | 24,05 | 10,95 | 10,64 | 0,55 |  |  |
| PR30minCSR1 | 1 | 1 | 0.5% DMSO | 0,5 | NA | 34,36 | 0,00000 | 34,44 | 23,97 | 10,47 | 10,64 | 0,55 |  |  |
| PR30minCSR2 | 2 | 1 | 0.5% DMSO | 0,5 | NA | 35,25 | 0,99541 | 34,08 | 23,90 | 10,17 | 10,64 | 0,55 |  |  |
| PR30minCSR3 | 3 | 1 | 0.5% DMSO | 0,5 | NA | 33,96 | 1,27104 | 35,00 | 23,77 | 11,23 | 10,64 | 0,55 |  |  |
| R16hCSR1 | 1 | 1 | 0.5% DMSO | 18 | NA | 34,34 | 0,00000 | 33,75 | 22,99 | 10,75 | 10,64 | 0,55 |  |  |
| R16hCSR2 | 2 | 1 | 0.5% DMSO | 18 | NA | 35,34 | 0,00000 | 34,34 | 23,58 | 10,76 | 10,64 | 0,55 |  |  |
| R16hCSR3 | 3 | 1 | 0.5% DMSO | 18 | NA | 32,14 | 0,09990 | 33,99 | 23,13 | 10,85 | 10,64 | 0,55 |  |  |
| R1hCSR1 | 1 | 1 | 0.5% DMSO | 3 | NA | 33,33 | 1,23050 | 34,42 | 24,12 | 10,30 | 10,64 | 0,55 |  |  |
| R1hCSR2 | 2 | 1 | 0.5% DMSO | 3 | NA | 35,51 | 0,00000 | 34,76 | 23,83 | 10,93 | 10,64 | 0,55 |  |  |
| R1hCSR3 | 3 | 1 | 0.5% DMSO | 3 | NA | 33,35 | 0,45068 | 35,00 | 25,13 | 9,87 | 10,64 | 0,55 |  |  |
| R2hCSR1 | 1 | 1 | 0.5% DMSO | 4 | NA | 33,85 | 0,24336 | 34,85 | 23,71 | 11,13 | 10,64 | 0,55 |  |  |
| R2hCSR2 | 2 | 1 | 0.5% DMSO | 4 | NA | 35,98 | 0,66578 | 33,47 | 23,98 | 9,49 | 10,64 | 0,55 |  |  |
| R2hCSR3 | 3 | 1 | 0.5% DMSO | 4 | NA | 33,08 | 1,03187 | 34,34 | 24,45 | 9,89 | 10,64 | 0,55 |  |  |
| R4hCSR1 | 1 | 1 | 0.5% DMSO | 6 | NA | 32,96 | 0,13681 | 33,80 | 24,16 | 9,63 | 10,64 | 0,55 |  |  |
| R4hCSR2 | 2 | 1 | 0.5% DMSO | 6 | NA | 36,70 | 0,17962 | 34,18 | 23,68 | 10,51 | 10,64 | 0,55 |  |  |
| R4hCSR3 | 3 | 1 | 0.5% DMSO | 6 | NA | 30,93 | 0,80456 | 35,00 | 24,23 | 10,77 | 10,64 | 0,55 |  |  |
| R8hCSR1 | 1 | 1 | 0.5% DMSO | 10 | NA | 34,46 | 0,81036 | 34,73 | 23,97 | 10,76 | 10,64 | 0,55 |  |  |
| R8hCSR2 | 2 | 1 | 0.5% DMSO | 10 | NA | 35,35 | 0,00000 | 35,00 | 23,75 | 11,25 | 10,64 | 0,55 |  |  |
| R8hCSR3 | 3 | 1 | 0.5% DMSO | 10 | NA | 35,12 | 2,98357 | 35,00 | 24,50 | 10,50 | 10,64 | 0,55 |  |  |
| RC16h0.1R1 | 1 | 1 | 0.1 µM 20E | 18 | NA | 29,08 | 0,14463 | 31,89 | 24,12 | 7,77 | 10,64 | 0,55 | -2,76 | 6,78 |
| RC16h0.1R2 | 2 | 1 | 0.1 µM 20E | 18 | NA | 30,76 | 0,39342 | 31,25 | 23,66 | 7,59 | 10,64 | 0,55 | -2,94 | 7,69 |
| RC16h0.1R3 | 3 | 1 | 0.1 µM 20E | 18 | NA | 28,04 | 0,03846 | 32,47 | 24,00 | 8,48 | 10,64 | 0,55 | -2,06 | 4,16 |
| RC1h0.1R1 | 1 | 1 | 0.1 µM 20E | 3 | NA | 29,25 | 0,09986 | 34,40 | 24,16 | 10,24 | 10,64 | 0,55 | -0,29 | 1,22 |
| RC1h0.1R2 | 2 | 1 | 0.1 µM 20E | 3 | NA | 30,06 | 0,33561 | 34,24 | 24,09 | 10,15 | 10,64 | 0,55 | -0,38 | 1,30 |
| RC1h0.1R3 | 3 | 1 | 0.1 µM 20E | 3 | NA | 31,64 | 0,66578 | 34,97 | 24,37 | 10,60 | 10,64 | 0,55 | 0,07 | 0,95 |
| RC2h0.1R1 | 1 | 1 | 0.1 µM 20E | 4 | NA | 28,92 | 0,60873 | 35,00 | 23,88 | 11,12 | 10,64 | 0,55 | 0,59 | 0,67 |
| RC2h0.1R2 | 2 | 1 | 0.1 µM 20E | 4 | NA | 29,12 | 0,06975 | 34,89 | 24,22 | 10,67 | 10,64 | 0,55 | 0,14 | 0,91 |
| RC2h0.1R3 | 3 | 1 | 0.1 µM 20E | 4 | NA | 28,84 | 0,65579 | 34,39 | 24,13 | 10,27 | 10,64 | 0,55 | -0,27 | 1,20 |
| RC4h0.1R1 | 1 | 1 | 0.1 µM 20E | 6 | NA | 28,53 | 0,64253 | 34,34 | 23,79 | 10,55 | 10,64 | 0,55 | 0,02 | 0,99 |
| RC4h0.1R2 | 2 | 1 | 0.1 µM 20E | 6 | NA | 29,73 | 0,00367 | 35,00 | 24,06 | 10,94 | 10,64 | 0,55 | 0,41 | 0,75 |
| RC4h0.1R3 | 3 | 1 | 0.1 µM 20E | 6 | NA | 30,99 | 0,65219 | 34,75 | 25,29 | 9,45 | 10,64 | 0,55 | -1,08 | 2,11 |
| RC8h0.1R1 | 1 | 1 | 0.1 µM 20E | 10 | NA | 29,12 | 0,49696 | 35,00 | 24,58 | 10,42 | 10,64 | 0,55 | -0,11 | 1,08 |
| RC8h0.1R2 | 2 | 1 | 0.1 µM 20E | 10 | NA | 27,61 | 0,01646 | 33,91 | 24,44 | 9,47 | 10,64 | 0,55 | -1,06 | 2,09 |
| RC8h0.1R3 | 3 | 1 | 0.1 µM 20E | 10 | NA | 31,02 | 0,91758 | 34,13 | 24,31 | 9,82 | 10,64 | 0,55 | -0,71 | 1,64 |
| RR16h0.1R1 | 1 | 1 | 0.1 µM 20E | 2 | 16 | 34,70 | 0,28067 | 33,17 | 22,38 | 10,79 | 10,64 | 0,55 | 0,26 | 0,84 |
| RR16h0.1R2 | 2 | 1 | 0.1 µM 20E | 2 | 16 | 36,68 | 1,40401 | 35,00 | 23,34 | 11,66 | 10,64 | 0,55 | 1,13 | 0,46 |
| RR16h0.1R3 | 3 | 1 | 0.1 µM 20E | 2 | 16 | 33,38 | 0,15175 | 35,00 | 24,14 | 10,86 | 10,64 | 0,55 | 0,33 | 0,80 |
| RR1h0.1R1 | 1 | 1 | 0.1 µM 20E | 2 | 1 | 29,93 | 0,14108 | 33,93 | 23,82 | 10,11 | 10,64 | 0,55 | -0,43 | 1,34 |
| RR1h0.1R2 | 2 | 1 | 0.1 µM 20E | 2 | 1 | 31,39 | 0,02609 | 33,35 | 23,82 | 9,53 | 10,64 | 0,55 | -1,00 | 2,00 |
| RR1h0.1R3 | 3 | 1 | 0.1 µM 20E | 2 | 1 | 33,06 | 0,00561 | 35,00 | 23,89 | 11,11 | 10,64 | 0,55 | 0,58 | 0,67 |
| RR2h0.1R1 | 1 | 1 | 0.1 µM 20E | 2 | 2 | 29,19 | 0,22069 | 34,70 | 24,20 | 10,50 | 10,64 | 0,55 | -0,03 | 1,02 |
| RR2h0.1R2 | 2 | 1 | 0.1 µM 20E | 2 | 2 | 32,43 | 0,06168 | 34,51 | 24,40 | 10,11 | 10,64 | 0,55 | -0,42 | 1,34 |
| RR2h0.1R3 | 3 | 1 | 0.1 µM 20E | 2 | 2 | 32,55 | 0,25455 | 35,00 | 24,71 | 10,29 | 10,64 | 0,55 | -0,24 | 1,18 |
| RR4h0.1R1 | 1 | 1 | 0.1 µM 20E | 2 | 4 | 30,83 | 0,52069 | 35,00 | 24,29 | 10,71 | 10,64 | 0,55 | 0,18 | 0,88 |
| RR4h0.1R2 | 2 | 1 | 0.1 µM 20E | 2 | 4 | 34,34 | 0,54199 | 34,59 | 23,49 | 11,10 | 10,64 | 0,55 | 0,57 | 0,68 |
| RR4h0.1R3 | 3 | 1 | 0.1 µM 20E | 2 | 4 | 34,31 | 1,14148 | 34,64 | 24,41 | 10,23 | 10,64 | 0,55 | -0,31 | 1,24 |
| RR8h0.1R1 | 1 | 1 | 0.1 µM 20E | 2 | 8 | 30,80 | 0,96806 | 35,00 | 23,73 | 11,27 | 10,64 | 0,55 | 0,74 | 0,60 |
| RR8h0.1R2 | 2 | 1 | 0.1 µM 20E | 2 | 8 | 33,88 | 0,36599 | 34,91 | 24,00 | 10,91 | 10,64 | 0,55 | 0,37 | 0,77 |
| RR8h0.1R3 | 3 | 1 | 0.1 µM 20E | 2 | 8 | 35,46 | 0,00000 | 35,00 | 25,12 | 9,88 | 10,64 | 0,55 | -0,66 | 1,58 |
| PR1h0.1R1 | 1 | 2 | 0.1 µM 20E | 1 | NA | 32,14227 | 0,122953 | 32,14 | 22,27 | 9,87 | 10,82 | 0,459332 | -0,95 | 1,93 |
| PR1h0.1R2 | 2 | 2 | 0.1 µM 20E | 1 | NA | 31,35242 | 0,781644 | 31,35 | 22,09 | 9,27 | 10,82 | 0,459332 | -1,55 | 2,94 |
| PR1hCSR1 | 1 | 2 | 0.1 µM 20E | 1 | NA | 32,83111 | 0,96061 | 32,83 | 22,01 | 10,82 | 10,82 | 0,459332 |  |  |
| PR1hCSR2 | 2 | 2 | 0.1 µM 20E | 1 | NA | 32,69109 | 0,044549 | 32,69 | 22,33 | 10,36 | 10,82 | 0,459332 |  |  |
| PR2h0.1R1 | 1 | 2 | 0.1 µM 20E | 2 | NA | 31,78958 | 0,484314 | 31,79 | 21,82 | 9,97 | 10,82 | 0,459332 | -0,85 | 1,80 |
| PR2h0.1R2 | 2 | 2 | 0.1 µM 20E | 2 | NA | 32,01326 | 0,333092 | 32,01 | 21,66 | 10,36 | 10,82 | 0,459332 | -0,46 | 1,38 |
| PR2hCSR1 | 1 | 2 | 0.5% DMSO | 2 | NA | 32,62979 | 0,527027 | 32,63 | 22,09 | 10,53 | 10,82 | 0,459332 |  |  |
| PR2hCSR2 | 2 | 2 | 0.5% DMSO | 2 | NA | 32,63335 | 0,864576 | 32,63 | 22,06 | 10,57 | 10,82 | 0,459332 |  |  |
| PR30min0.1R1 | 1 | 2 | 0.1 µM 20E | 0,5 | NA | 32,98442 | 0,526921 | 32,98 | 23,06 | 9,93 | 10,82 | 0,459332 | -0,89 | 1,86 |
| PR30min0.1R2 | 2 | 2 | 0.1 µM 20E | 0,5 | NA | 31,57967 | 0,250976 | 31,58 | 22,21 | 9,37 | 10,82 | 0,459332 | -1,45 | 2,73 |
| PR30minCSR1 | 1 | 2 | 0.5% DMSO | 0,5 | NA | 31,94231 | 0,171447 | 31,94 | 21,97 | 9,97 | 10,82 | 0,459332 |  |  |
| PR30minCSR2 | 2 | 2 | 0.5% DMSO | 0,5 | NA | 33,48057 | 0,156332 | 33,48 | 22,57 | 10,91 | 10,82 | 0,459332 |  |  |
| R16hCSR1 | 1 | 2 | 0.5% DMSO | 18 | NA | 33,89625 | 0,065932 | 33,90 | 22,90 | 11,00 | 10,82 | 0,459332 |  |  |
| R16hCSR2 | 2 | 2 | 0.5% DMSO | 18 | NA | 33,9256 | 0,225707 | 33,93 | 22,21 | 11,72 | 10,82 | 0,459332 |  |  |
| R1hCSR1 | 1 | 2 | 0.5% DMSO | 3 | NA | 33,91248 | 0,394075 | 33,91 | 23,14 | 10,77 | 10,82 | 0,459332 |  |  |
| R1hCSR2 | 2 | 2 | 0.5% DMSO | 3 | NA | 33,01047 | 0,470338 | 33,01 | 22,39 | 10,62 | 10,82 | 0,459332 |  |  |
| R2hCSR1 | 1 | 2 | 0.5% DMSO | 4 | NA | 32,83511 | 0,27141 | 32,84 | 22,43 | 10,41 | 10,82 | 0,459332 |  |  |
| R2hCSR2 | 2 | 2 | 0.5% DMSO | 4 | NA | 32,83668 | 0,507569 | 32,84 | 22,18 | 10,65 | 10,82 | 0,459332 |  |  |
| R4hCSR1 | 1 | 2 | 0.5% DMSO | 6 | NA | 33,39205 | 0,582911 | 33,39 | 22,44 | 10,95 | 10,82 | 0,459332 |  |  |
| R4hCSR2 | 2 | 2 | 0.5% DMSO | 6 | NA | 33,18389 | 0,008356 | 33,18 | 22,27 | 10,91 | 10,82 | 0,459332 |  |  |
| R8hCSR1 | 1 | 2 | 0.5% DMSO | 10 | NA | 34,55 | 0,375798 | 34,55 | 22,66 | 11,89 | 10,82 | 0,459332 |  |  |
| R8hCSR2 | 2 | 2 | 0.5% DMSO | 10 | NA | 33,64927 | 0,057262 | 33,65 | 22,63 | 11,02 | 10,82 | 0,459332 |  |  |
| RC16h0.1R1 | 1 | 2 | 0.1 µM 20E | 18 | NA | 30,03328 | 1,067073 | 30,03 | 22,14 | 7,89 | 10,82 | 0,459332 | -2,93 | 7,60 |
| RC16h0.1R2 | 2 | 2 | 0.1 µM 20E | 18 | NA | 30,55537 | 0,568184 | 30,56 | 22,13 | 8,43 | 10,82 | 0,459332 | -2,39 | 5,26 |
| RC1h0.1R1 | 1 | 2 | 0.1 µM 20E | 3 | NA | 31,86272 | 0,269832 | 31,86 | 22,31 | 9,55 | 10,82 | 0,459332 | -1,27 | 2,40 |
| RC1h0.1R2 | 2 | 2 | 0.1 µM 20E | 3 | NA | 32,02209 | 0,28274 | 32,02 | 22,58 | 9,44 | 10,82 | 0,459332 | -1,38 | 2,59 |
| RC2h0.1R1 | 1 | 2 | 0.1 µM 20E | 4 | NA | 31,08089 | 1,071561 | 31,08 | 22,23 | 8,85 | 10,82 | 0,459332 | -1,97 | 3,92 |
| RC2h0.1R2 | 2 | 2 | 0.1 µM 20E | 4 | NA | 31,91591 | 0,277169 | 31,92 | 22,70 | 9,22 | 10,82 | 0,459332 | -1,60 | 3,04 |
| RC4h0.1R1 | 1 | 2 | 0.1 µM 20E | 6 | NA | 30,73411 | 0,817835 | 30,73 | 22,21 | 8,53 | 10,82 | 0,459332 | -2,29 | 4,89 |
| RC4h0.1R2 | 2 | 2 | 0.1 µM 20E | 6 | NA | 31,88814 | 0,215875 | 31,89 | 22,33 | 9,56 | 10,82 | 0,459332 | -1,26 | 2,39 |
| RC8h0.1R1 | 1 | 2 | 0.1 µM 20E | 10 | NA | 30,72097 | 0,423492 | 30,72 | 22,22 | 8,50 | 10,82 | 0,459332 | -2,32 | 5,00 |
| RC8h0.1R2 | 2 | 2 | 0.1 µM 20E | 10 | NA | 30,83242 | 0,050932 | 30,83 | 22,50 | 8,33 | 10,82 | 0,459332 | -2,49 | 5,61 |
| RR16h0.1R1 | 1 | 2 | 0.1 µM 20E | 2 | 16 | 32,63515 | 0,358875 | 32,64 | 22,06 | 10,57 | 10,82 | 0,459332 | -0,25 | 1,19 |
| RR16h0.1R2 | 2 | 2 | 0.1 µM 20E | 2 | 16 | 31,56489 | 0,281721 | 31,56 | 21,29 | 10,27 | 10,82 | 0,459332 | -0,55 | 1,46 |
| RR1h0.1R1 | 1 | 2 | 0.1 µM 20E | 2 | 1 |  | 0 |  | 24,69 |  | 10,82 | 0,459332 |  |  |
| RR1h0.1R2 | 2 | 2 | 0.1 µM 20E | 2 | 1 | 32,27981 | 0,524043 | 32,28 | 22,55 | 9,73 | 10,82 | 0,459332 | -1,09 | 2,13 |
| RR2h0.1R1 | 1 | 2 | 0.1 µM 20E | 2 | 2 | 31,95222 | 0,084941 | 31,95 | 23,24 | 8,71 | 10,82 | 0,459332 | -2,11 | 4,32 |
| RR2h0.1R2 | 2 | 2 | 0.1 µM 20E | 2 | 2 | 31,30502 | 0,284128 | 31,31 | 22,25 | 9,05 | 10,82 | 0,459332 | -1,77 | 3,41 |
| RR4h0.1R1 | 1 | 2 | 0.1 µM 20E | 2 | 4 | 32,64942 | 0,078095 | 32,65 | 22,47 | 10,18 | 10,82 | 0,459332 | -0,64 | 1,56 |
| RR4h0.1R2 | 2 | 2 | 0.1 µM 20E | 2 | 4 | 31,9675 | 1,354869 | 31,97 | 22,09 | 9,88 | 10,82 | 0,459332 | -0,94 | 1,92 |
| RR8h0.1R1 | 1 | 2 | 0.1 µM 20E | 2 | 8 | 32,07941 | 0,781029 | 32,08 | 22,92 | 9,16 | 10,82 | 0,459332 | -1,66 | 3,16 |
| RR8h0.1R2 | 2 | 2 | 0.1 µM 20E | 2 | 8 | 30,56535 | 0,857237 | 30,57 | 22,18 | 8,38 | 10,82 | 0,459332 | -2,44 | 5,42 |
| PR1h0.1R1 | 1 | 3 | 0.1 µM 20E | 1 | NA | 31,54363 | 0,074331 | 31,54 | 21,66 | 9,89 | 9,14 | 1,090219 | 0,75 | 0,59 |
| PR1h0.1R2 | 2 | 3 | 0.1 µM 20E | 1 | NA | 32,05713 | 0,317787 | 32,06 | 22,08 | 9,98 | 9,14 | 1,090219 | 0,84 | 0,56 |
| PR1hCSR1 | 1 | 3 | 0.1 µM 20E | 1 | NA | 31,02039 | 0,11472 | 31,02 | 21,26 | 9,76 | 9,14 | 1,090219 |  |  |
| PR1hCSR2 | 2 | 3 | 0.1 µM 20E | 1 | NA | 32,35286 | 0,974667 | 32,35 | 21,94 | 10,41 | 9,14 | 1,090219 |  |  |
| PR2h0.1R1 | 1 | 3 | 0.1 µM 20E | 2 | NA | 32,89918 | 1,039133 | 32,90 | 22,27 | 10,63 | 9,14 | 1,090219 | 1,50 | 0,35 |
| PR2h0.1R2 | 2 | 3 | 0.1 µM 20E | 2 | NA | 31,88734 | 0,405348 | 31,89 | 22,90 | 8,99 | 9,14 | 1,090219 | -0,15 | 1,11 |
| PR2hCSR1 | 1 | 3 | 0.5% DMSO | 2 | NA | 31,52417 | 0,734483 | 31,52 | 21,14 | 10,38 | 9,14 | 1,090219 |  |  |
| PR2hCSR2 | 2 | 3 | 0.5% DMSO | 2 | NA | 31,87316 | 0,626718 | 31,87 | 22,37 | 9,50 | 9,14 | 1,090219 |  |  |
| PR30min0.1R1 | 1 | 3 | 0.1 µM 20E | 0,5 | NA | 32,58154 | 0,268435 | 32,58 | 22,13 | 10,45 | 9,14 | 1,090219 | 1,31 | 0,40 |
| PR30min0.1R2 | 2 | 3 | 0.1 µM 20E | 0,5 | NA | 32,3351 | 0,838066 | 32,34 | 22,43 | 9,90 | 9,14 | 1,090219 | 0,77 | 0,59 |
| PR30minCSR1 | 1 | 3 | 0.5% DMSO | 0,5 | NA | 32,17287 | 0,146091 | 32,17 | 22,05 | 10,12 | 9,14 | 1,090219 |  |  |
| PR30minCSR2 | 2 | 3 | 0.5% DMSO | 0,5 | NA | 32,96866 | 1,400907 | 32,97 | 21,65 | 11,32 | 9,14 | 1,090219 |  |  |
| R16hCSR1 | 1 | 3 | 0.5% DMSO | 18 | NA | 34,10741 | 0 | 34,11 | 24,97 | 9,14 | 9,14 | 1,090219 |  |  |
| R16hCSR2 | 2 | 3 | 0.5% DMSO | 18 | NA | 34,12598 | 0,615011 | 34,13 | 24,64 | 9,48 | 9,14 | 1,090219 |  |  |
| R1hCSR1 | 1 | 3 | 0.5% DMSO | 3 | NA | 33,93972 | 0,848008 | 33,94 | 26,16 | 7,78 | 9,14 | 1,090219 |  |  |
| R1hCSR2 | 2 | 3 | 0.5% DMSO | 3 | NA | 32,34911 | 0,455369 | 32,35 | 24,71 | 7,63 | 9,14 | 1,090219 |  |  |
| R2hCSR1 | 1 | 3 | 0.5% DMSO | 4 | NA | 33,39501 | 1,009859 | 33,40 | 25,86 | 7,54 | 9,14 | 1,090219 |  |  |
| R2hCSR2 | 2 | 3 | 0.5% DMSO | 4 | NA | 32,99076 | 0,281235 | 32,99 | 25,37 | 7,62 | 9,14 | 1,090219 |  |  |
| R4hCSR1 | 1 | 3 | 0.5% DMSO | 6 | NA | 35,61965 | 0,477051 | 35,00 | 26,51 | 8,49 | 9,14 | 1,090219 |  |  |
| R4hCSR2 | 2 | 3 | 0.5% DMSO | 6 | NA | 32,43348 | 0,335882 | 32,43 | 23,63 | 8,81 | 9,14 | 1,090219 |  |  |
| R8hCSR1 | 1 | 3 | 0.5% DMSO | 10 | NA | 36,08849 | 0 | 35,00 | 25,82 | 9,18 | 9,14 | 1,090219 |  |  |
| R8hCSR2 | 2 | 3 | 0.5% DMSO | 10 | NA | 32,52505 | 0,427954 | 32,53 | 23,53 | 8,99 | 9,14 | 1,090219 |  |  |
| RC16h0.1R1 | 1 | 3 | 0.1 µM 20E | 18 | NA | 32,22948 | 1,532375 | 32,23 | 22,76 | 9,47 | 9,14 | 1,090219 | 0,34 | 0,79 |
| RC16h0.1R2 | 2 | 3 | 0.1 µM 20E | 18 | NA | 31,53151 | 0,737989 | 31,53 | 22,70 | 8,83 | 9,14 | 1,090219 | -0,31 | 1,24 |
| RC1h0.1R1 | 1 | 3 | 0.1 µM 20E | 3 | NA | 33,75694 | 0,794381 | 33,76 | 24,27 | 9,48 | 9,14 | 1,090219 | 0,35 | 0,79 |
| RC1h0.1R2 | 2 | 3 | 0.1 µM 20E | 3 | NA | 33,66122 | 0,474575 | 33,66 | 24,45 | 9,21 | 9,14 | 1,090219 | 0,08 | 0,95 |
| RC2h0.1R1 | 1 | 3 | 0.1 µM 20E | 4 | NA | 32,77417 | 0,848985 | 32,77 | 23,46 | 9,31 | 9,14 | 1,090219 | 0,18 | 0,88 |
| RC2h0.1R2 | 2 | 3 | 0.1 µM 20E | 4 | NA | 33,19353 | 0,290643 | 33,19 | 23,47 | 9,72 | 9,14 | 1,090219 | 0,58 | 0,67 |
| RC4h0.1R1 | 1 | 3 | 0.1 µM 20E | 6 | NA | 32,54019 | 0 | 32,54 | 23,81 | 8,73 | 9,14 | 1,090219 | -0,41 | 1,33 |
| RC4h0.1R2 | 2 | 3 | 0.1 µM 20E | 6 | NA | 33,69383 | 0,160131 | 33,69 | 24,12 | 9,57 | 9,14 | 1,090219 | 0,44 | 0,74 |
| RC8h0.1R1 | 1 | 3 | 0.1 µM 20E | 10 | NA | 32,27196 | 1,407923 | 32,27 | 22,51 | 9,76 | 9,14 | 1,090219 | 0,62 | 0,65 |
| RC8h0.1R2 | 2 | 3 | 0.1 µM 20E | 10 | NA | 32,50981 | 0,601856 | 32,51 | 23,33 | 9,18 | 9,14 | 1,090219 | 0,04 | 0,97 |
| RR16h0.1R1 | 1 | 3 | 0.1 µM 20E | 2 | 16 | 32,5564 | 0,184088 | 32,56 | 22,20 | 10,35 | 9,14 | 1,090219 | 1,22 | 0,43 |
| RR16h0.1R2 | 2 | 3 | 0.1 µM 20E | 2 | 16 | 32,56892 | 0,328463 | 32,57 | 21,39 | 11,18 | 9,14 | 1,090219 | 2,04 | 0,24 |
| RR1h0.1R1 | 1 | 3 | 0.1 µM 20E | 2 | 1 | 32,97287 | 0,358813 | 32,97 | 23,37 | 9,61 | 9,14 | 1,090219 | 0,47 | 0,72 |
| RR1h0.1R2 | 2 | 3 | 0.1 µM 20E | 2 | 1 | 33,75107 | 0,762911 | 33,75 | 23,64 | 10,11 | 9,14 | 1,090219 | 0,97 | 0,51 |
| RR2h0.1R1 | 1 | 3 | 0.1 µM 20E | 2 | 2 | 33,49836 | 0,163349 | 33,50 | 23,24 | 10,26 | 9,14 | 1,090219 | 1,12 | 0,46 |
| RR2h0.1R2 | 2 | 3 | 0.1 µM 20E | 2 | 2 | 31,99926 | 0,128091 | 32,00 | 22,70 | 9,30 | 9,14 | 1,090219 | 0,17 | 0,89 |
| RR4h0.1R1 | 1 | 3 | 0.1 µM 20E | 2 | 4 | 33,71015 | 1,419082 | 33,71 | 23,41 | 10,30 | 9,14 | 1,090219 | 1,17 | 0,45 |
| RR4h0.1R2 | 2 | 3 | 0.1 µM 20E | 2 | 4 | 33,38902 | 0,932925 | 33,39 | 23,58 | 9,81 | 9,14 | 1,090219 | 0,67 | 0,63 |
| RR8h0.1R1 | 1 | 3 | 0.1 µM 20E | 2 | 8 | 33,31476 | 0,347733 | 33,31 | 22,76 | 10,56 | 9,14 | 1,090219 | 1,42 | 0,37 |
| RR8h0.1R2 | 2 | 3 | 0.1 µM 20E | 2 | 8 | 34,39361 | 0,011431 | 34,39 | 22,54 | 11,85 | 9,14 | 1,090219 | 2,72 | 0,15 |

| sample id | replicate | experiment number | Treatment | exposure time/hours | recovery time/hours | Avg Cq | st.dev Cq | corrected Cq (max cq 35) | Refrence gene Cq | delta Cq | Avg. solvent control delta Cq | st.dev solvent control delta Cq | delta delta Cq | 2^ddCq |
| --- | --- | --- | --- | --- | --- | --- | --- | --- | --- | --- | --- | --- | --- | --- |
| PR1hA0.1R1 | 1 | 1 | 0.1 µM 20E | 1 | NA | 26,13 | 0,02089 | 27,78 | 23,08 | 4,70 | 9,05 | 0,56 | -2,45 | 5,47 |
| PR1hA0.1R2 | 2 | 1 | 0.1 µM 20E | 1 | NA | 28,26 | 0,00129 | 27,35 | 22,41 | 4,94 | 9,05 | 0,56 | -2,54 | 5,82 |
| PR1hA0.1R3 | 3 | 1 | 0.1 µM 20E | 1 | NA | 36,54 | 1,24381 | 32,31 | 23,03 | 9,28 | 9,05 | 0,56 | -1,94 | 3,83 |
| PR1hB0.1R1 | 1 | 1 | 0.1 µM 20E | 1 | NA | 29,57 | 0,14238 | 29,38 | 23,53 | 5,85 | 9,05 | 0,56 |  |  |
| PR1hB0.1R2 | 2 | 1 | 0.1 µM 20E | 1 | NA | 28,76 | 0,38295 | 27,02 | 22,44 | 4,58 | 9,05 | 0,56 |  |  |
| PR1hB0.1R3 | 3 | 1 | 0.1 µM 20E | 1 | NA | 36,57 | 0,16753 | 31,49 | 22,64 | 8,84 | 9,05 | 0,56 |  |  |
| PR1hCSR1 | 1 | 1 | 0.1 µM 20E | 1 | NA | 37,17 | 0,00000 | 31,49 | 23,32 | 8,18 | 9,05 | 0,56 |  |  |
| PR1hCSR2 | 2 | 1 | 0.1 µM 20E | 1 | NA | 35,34 | 1,63820 | 31,23 | 22,37 | 8,86 | 9,05 | 0,56 |  |  |
| PR1hCSR3 | 3 | 1 | 0.1 µM 20E | 1 | NA | 33,22 | 1,84902 | 32,49 | 22,81 | 9,67 | 9,05 | 0,56 |  |  |
| PR2hA0.1R1 | 1 | 1 | 0.1 µM 20E | 2 | NA | 26,03 | 0,09343 | 28,36 | 23,67 | 4,69 | 9,05 | 0,56 | -3,77 | 13,67 |
| PR2hA0.1R2 | 2 | 1 | 0.1 µM 20E | 2 | NA | 28,49 | 0,03464 | 26,93 | 22,77 | 4,15 | 9,05 | 0,56 | -4,29 | 19,55 |
| PR2hA0.1R3 | 3 | 1 | 0.1 µM 20E | 2 | NA | 29,40 | 0,30653 | 27,88 | 22,45 | 5,43 | 9,05 | 0,56 | 0,01 | 0,99 |
| PR2hB0.1R1 | 1 | 1 | 0.1 µM 20E | 2 | NA | 28,77 | 0,34167 | 29,17 | 23,98 | 5,19 | 9,05 | 0,56 |  |  |
| PR2hB0.1R2 | 2 | 1 | 0.1 µM 20E | 2 | NA | 28,28 | 0,31111 | 26,55 | 21,82 | 4,73 | 9,05 | 0,56 |  |  |
| PR2hB0.1R3 | 3 | 1 | 0.1 µM 20E | 2 | NA | 30,36 | 0,67995 | 27,30 | 22,32 | 4,98 | 9,05 | 0,56 |  |  |
| PR2hCSR1 | 1 | 1 | 0.5% DMSO | 2 | NA | 34,87 | 1,04142 | 32,77 | 23,19 | 9,57 | 9,05 | 0,56 |  |  |
| PR2hCSR2 | 2 | 1 | 0.5% DMSO | 2 | NA | 36,54 | 0,00000 | 30,92 | 22,41 | 8,51 | 9,05 | 0,56 |  |  |
| PR2hCSR3 | 3 | 1 | 0.5% DMSO | 2 | NA | 33,75 | 0,04970 | 33,88 | 23,14 | 10,74 | 9,05 | 0,56 |  |  |
| PR30minA0.1R1 | 1 | 1 | 0.1 µM 20E | 0,5 | NA | 26,71 | 0,26915 | 28,26 | 22,52 | 5,75 | 9,05 | 0,56 | -4,11 | 17,25 |
| PR30minA0.1R2 | 2 | 1 | 0.1 µM 20E | 0,5 | NA | 30,46 | 0,05462 | 28,37 | 22,37 | 6,01 | 9,05 | 0,56 | -4,61 | 24,41 |
| PR30minA0.1R3 | 3 | 1 | 0.1 µM 20E | 0,5 | NA | 36,15 | 0,00000 | 31,95 | 22,43 | 9,52 | 9,05 | 0,56 | -3,84 | 14,35 |
| PR30minB0.1R1 | 1 | 1 | 0.1 µM 20E | 0,5 | NA | 30,70 | 0,50500 | 29,87 | 22,42 | 7,45 | 9,05 | 0,56 |  |  |
| PR30minB0.1R2 | 2 | 1 | 0.1 µM 20E | 0,5 | NA | 30,88 | 0,16317 | 29,79 | 22,78 | 7,01 | 9,05 | 0,56 |  |  |
| PR30minB0.1R3 | 3 | 1 | 0.1 µM 20E | 0,5 | NA | 35,39 | 0,00000 | 32,79 | 22,98 | 9,81 | 9,05 | 0,56 |  |  |
| PR30minCSR1 | 1 | 1 | 0.5% DMSO | 0,5 | NA | 34,36 | 0,00000 | 32,19 | 23,12 | 9,07 | 9,05 | 0,56 |  |  |
| PR30minCSR2 | 2 | 1 | 0.5% DMSO | 0,5 | NA | 35,25 | 0,99541 | 32,05 | 23,05 | 9,00 | 9,05 | 0,56 |  |  |
| PR30minCSR3 | 3 | 1 | 0.5% DMSO | 0,5 | NA | 33,96 | 1,27104 | 32,08 | 22,64 | 9,44 | 9,05 | 0,56 |  |  |
| R16hCSR1 | 1 | 1 | 0.5% DMSO | 18 | NA | 34,34 | 0,00000 | 31,50 | 22,28 | 9,22 | 9,05 | 0,56 |  |  |
| R16hCSR2 | 2 | 1 | 0.5% DMSO | 18 | NA | 35,34 | 0,00000 | 32,09 | 23,12 | 8,97 | 9,05 | 0,56 |  |  |
| R16hCSR3 | 3 | 1 | 0.5% DMSO | 18 | NA | 32,14 | 0,09990 | 31,43 | 22,49 | 8,94 | 9,05 | 0,56 |  |  |
| R1hCSR1 | 1 | 1 | 0.5% DMSO | 3 | NA | 33,33 | 1,23050 | 31,25 | 23,00 | 8,25 | 9,05 | 0,56 |  |  |
| R1hCSR2 | 2 | 1 | 0.5% DMSO | 3 | NA | 35,51 | 0,00000 | 31,36 | 22,53 | 8,83 | 9,05 | 0,56 |  |  |
| R1hCSR3 | 3 | 1 | 0.5% DMSO | 3 | NA | 33,35 | 0,45068 | 32,51 | 23,79 | 8,72 | 9,05 | 0,56 |  |  |
| R2hCSR1 | 1 | 1 | 0.5% DMSO | 4 | NA | 33,85 | 0,24336 | 32,20 | 22,58 | 9,63 | 9,05 | 0,56 |  |  |
| R2hCSR2 | 2 | 1 | 0.5% DMSO | 4 | NA | 35,98 | 0,66578 | 31,30 | 22,43 | 8,87 | 9,05 | 0,56 |  |  |
| R2hCSR3 | 3 | 1 | 0.5% DMSO | 4 | NA | 33,08 | 1,03187 | 31,38 | 23,29 | 8,09 | 9,05 | 0,56 |  |  |
| R4hCSR1 | 1 | 1 | 0.5% DMSO | 6 | NA | 32,96 | 0,13681 | 31,94 | 22,95 | 8,99 | 9,05 | 0,56 |  |  |
| R4hCSR2 | 2 | 1 | 0.5% DMSO | 6 | NA | 36,70 | 0,17962 | 31,66 | 22,48 | 9,18 | 9,05 | 0,56 |  |  |
| R4hCSR3 | 3 | 1 | 0.5% DMSO | 6 | NA | 30,93 | 0,80456 | 33,03 | 23,30 | 9,73 | 9,05 | 0,56 |  |  |
| R8hCSR1 | 1 | 1 | 0.5% DMSO | 10 | NA | 34,46 | 0,81036 | 32,24 | 23,22 | 9,01 | 9,05 | 0,56 |  |  |
| R8hCSR2 | 2 | 1 | 0.5% DMSO | 10 | NA | 35,35 | 0,00000 | 31,75 | 22,55 | 9,19 | 9,05 | 0,56 |  |  |
| R8hCSR3 | 3 | 1 | 0.5% DMSO | 10 | NA | 35,12 | 2,98357 | 31,68 | 23,13 | 8,55 | 9,05 | 0,56 |  |  |
| RC16h0.1R1 | 1 | 1 | 0.1 µM 20E | 18 | NA | 29,08 | 0,14463 | 27,75 | 23,38 | 4,37 | 9,05 | 0,56 | -4,68 | 25,61 |
| RC16h0.1R2 | 2 | 1 | 0.1 µM 20E | 18 | NA | 30,76 | 0,39342 | 27,79 | 22,99 | 4,80 | 9,05 | 0,56 | -4,25 | 19,07 |
| RC16h0.1R3 | 3 | 1 | 0.1 µM 20E | 18 | NA | 28,04 | 0,03846 | 28,72 | 23,22 | 5,50 | 9,05 | 0,56 | -3,55 | 11,71 |
| RC1h0.1R1 | 1 | 1 | 0.1 µM 20E | 3 | NA | 29,25 | 0,09986 | 28,36 | 23,28 | 5,08 | 9,05 | 0,56 | -3,97 | 15,72 |
| RC1h0.1R2 | 2 | 1 | 0.1 µM 20E | 3 | NA | 30,06 | 0,33561 | 27,70 | 22,53 | 5,18 | 9,05 | 0,56 | -3,87 | 14,64 |
| RC1h0.1R3 | 3 | 1 | 0.1 µM 20E | 3 | NA | 31,64 | 0,66578 | 29,14 | 23,38 | 5,75 | 9,05 | 0,56 | -3,30 | 9,82 |
| RC2h0.1R1 | 1 | 1 | 0.1 µM 20E | 4 | NA | 28,92 | 0,60873 | 28,92 | 23,16 | 5,75 | 9,05 | 0,56 | -3,30 | 9,82 |
| RC2h0.1R2 | 2 | 1 | 0.1 µM 20E | 4 | NA | 29,12 | 0,06975 | 27,57 | 23,06 | 4,51 | 9,05 | 0,56 | -4,54 | 23,25 |
| RC2h0.1R3 | 3 | 1 | 0.1 µM 20E | 4 | NA | 28,84 | 0,65579 | 28,19 | 23,16 | 5,03 | 9,05 | 0,56 | -4,02 | 16,28 |
| RC4h0.1R1 | 1 | 1 | 0.1 µM 20E | 6 | NA | 28,53 | 0,64253 | 28,81 | 23,13 | 5,68 | 9,05 | 0,56 | -3,37 | 10,34 |
| RC4h0.1R2 | 2 | 1 | 0.1 µM 20E | 6 | NA | 29,73 | 0,00367 | 26,88 | 21,19 | 5,69 | 9,05 | 0,56 | -3,36 | 10,25 |
| RC4h0.1R3 | 3 | 1 | 0.1 µM 20E | 6 | NA | 30,99 | 0,65219 | 27,85 | 22,42 | 5,43 | 9,05 | 0,56 | -3,62 | 12,27 |
| RC8h0.1R1 | 1 | 1 | 0.1 µM 20E | 10 | NA | 29,12 | 0,49696 | 28,94 | 23,62 | 5,32 | 9,05 | 0,56 | -3,73 | 13,26 |
| RC8h0.1R2 | 2 | 1 | 0.1 µM 20E | 10 | NA | 27,61 | 0,01646 | 27,98 | 23,12 | 4,86 | 9,05 | 0,56 | -4,19 | 18,21 |
| RC8h0.1R3 | 3 | 1 | 0.1 µM 20E | 10 | NA | 31,02 | 0,91758 | 28,40 | 23,47 | 4,92 | 9,05 | 0,56 | -4,13 | 17,45 |
| RR16h0.1R1 | 1 | 1 | 0.1 µM 20E | 2 | 16 | 34,70 | 0,28067 | 31,05 | 21,89 | 9,16 | 9,05 | 0,56 | 0,11 | 0,93 |
| RR16h0.1R2 | 2 | 1 | 0.1 µM 20E | 2 | 16 | 36,68 | 1,40401 | 31,78 | 22,71 | 9,07 | 9,05 | 0,56 | 0,02 | 0,99 |
| RR16h0.1R3 | 3 | 1 | 0.1 µM 20E | 2 | 16 | 33,38 | 0,15175 | 33,08 | 23,39 | 9,69 | 9,05 | 0,56 | 0,64 | 0,64 |
| RR1h0.1R1 | 1 | 1 | 0.1 µM 20E | 2 | 1 | 29,93 | 0,14108 | 29,13 | 23,21 | 5,92 | 9,05 | 0,56 | -3,13 | 8,77 |
| RR1h0.1R2 | 2 | 1 | 0.1 µM 20E | 2 | 1 | 31,39 | 0,02609 | 28,37 | 22,62 | 5,75 | 9,05 | 0,56 | -3,30 | 9,88 |
| RR1h0.1R3 | 3 | 1 | 0.1 µM 20E | 2 | 1 | 33,06 | 0,00561 | 30,13 | 23,32 | 6,81 | 9,05 | 0,56 | -2,24 | 4,71 |
| RR2h0.1R1 | 1 | 1 | 0.1 µM 20E | 2 | 2 | 29,19 | 0,22069 | 31,08 | 23,53 | 7,56 | 9,05 | 0,56 | -1,49 | 2,81 |
| RR2h0.1R2 | 2 | 1 | 0.1 µM 20E | 2 | 2 | 32,43 | 0,06168 | 30,08 | 23,04 | 7,04 | 9,05 | 0,56 | -2,01 | 4,03 |
| RR2h0.1R3 | 3 | 1 | 0.1 µM 20E | 2 | 2 | 32,55 | 0,25455 | 30,20 | 22,77 | 7,43 | 9,05 | 0,56 | -1,62 | 3,08 |
| RR4h0.1R1 | 1 | 1 | 0.1 µM 20E | 2 | 4 | 30,83 | 0,52069 | 32,43 | 23,63 | 8,80 | 9,05 | 0,56 | -0,25 | 1,19 |
| RR4h0.1R2 | 2 | 1 | 0.1 µM 20E | 2 | 4 | 34,34 | 0,54199 | 30,03 | 22,32 | 7,72 | 9,05 | 0,56 | -1,33 | 2,52 |
| RR4h0.1R3 | 3 | 1 | 0.1 µM 20E | 2 | 4 | 34,31 | 1,14148 | 31,73 | 21,55 | 10,18 | 9,05 | 0,56 | 1,13 | 0,46 |
| RR8h0.1R1 | 1 | 1 | 0.1 µM 20E | 2 | 8 | 30,80 | 0,96806 | 31,77 | 23,33 | 8,44 | 9,05 | 0,56 | -0,61 | 1,52 |
| RR8h0.1R2 | 2 | 1 | 0.1 µM 20E | 2 | 8 | 33,88 | 0,36599 | 32,39 | 23,17 | 9,21 | 9,05 | 0,56 | 0,16 | 0,89 |
| RR8h0.1R3 | 3 | 1 | 0.1 µM 20E | 2 | 8 | 35,46 | 0,00000 | 32,17 | 21,18 | 10,99 | 9,05 | 0,56 | 1,94 | 0,26 |
| PR1h0.1R1 | 1 | 2 | 0.1 µM 20E | 1 | NA | 29,35 | 0,073459 | 29,35 | 22,96 | 6,40 | 9,35 | 0,54 | -2,95 | 7,74 |
| PR1h0.1R2 | 2 | 2 | 0.1 µM 20E | 1 | NA | 28,63 | 0,186851 | 28,63 | 22,52 | 6,12 | 9,35 | 0,54 | -3,23 | 9,41 |
| PR1hCSR1 | 1 | 2 | 0.1 µM 20E | 1 | NA | 32,94 | 0,066974 | 32,94 | 22,69 | 10,25 | 9,35 | 0,54 |  |  |
| PR1hCSR2 | 2 | 2 | 0.1 µM 20E | 1 | NA | 33,86 | 0,712965 | 33,86 | 24,71 | 9,15 | 9,35 | 0,54 |  |  |
| PR2h0.1R1 | 1 | 2 | 0.1 µM 20E | 2 | NA | 28,65 | 0,050238 | 28,65 | 22,57 | 6,08 | 9,35 | 0,54 | -3,27 | 9,64 |
| PR2h0.1R2 | 2 | 2 | 0.1 µM 20E | 2 | NA | 28,79 | 0,11129 | 28,79 | 22,66 | 6,14 | 9,35 | 0,54 | -3,21 | 9,28 |
| PR2hCSR1 | 1 | 2 | 0.5% DMSO | 2 | NA | 33,21 | 0,299186 | 33,21 | 23,09 | 10,12 | 9,35 | 0,54 |  |  |
| PR2hCSR2 | 2 | 2 | 0.5% DMSO | 2 | NA | 32,90 | 0,666843 | 32,90 | 23,49 | 9,41 | 9,35 | 0,54 |  |  |
| PR30min0.1R1 | 1 | 2 | 0.1 µM 20E | 0,5 | NA | 30,57 | 0,069946 | 30,57 | 22,85 | 7,72 | 9,35 | 0,54 | -1,63 | 3,09 |
| PR30min0.1R2 | 2 | 2 | 0.1 µM 20E | 0,5 | NA | 30,23 | 0,255692 | 30,23 | 23,39 | 6,84 | 9,35 | 0,54 | -2,51 | 5,71 |
| PR30minCSR1 | 1 | 2 | 0.5% DMSO | 0,5 | NA | 32,89 | 0,267333 | 32,89 | 22,62 | 10,27 | 9,35 | 0,54 |  |  |
| PR30minCSR2 | 2 | 2 | 0.5% DMSO | 0,5 | NA | 34,09 | 0 | 34,09 | 24,49 | 9,60 | 9,35 | 0,54 |  |  |
| R16hCSR1 | 1 | 2 | 0.5% DMSO | 18 | NA | 32,64 | 0,117936 | 32,64 | 23,74 | 8,90 | 9,35 | 0,54 |  |  |
| R16hCSR2 | 2 | 2 | 0.5% DMSO | 18 | NA | 32,56 | 0,127274 | 32,56 | 23,99 | 8,57 | 9,35 | 0,54 |  |  |
| R1hCSR1 | 1 | 2 | 0.5% DMSO | 3 | NA | 33,13 | 0,239894 | 33,13 | 24,40 | 8,73 | 9,35 | 0,54 |  |  |
| R1hCSR2 | 2 | 2 | 0.5% DMSO | 3 | NA | 32,84 | 0,824693 | 32,84 | 23,77 | 9,07 | 9,35 | 0,54 |  |  |
| R2hCSR1 | 1 | 2 | 0.5% DMSO | 4 | NA | 33,34 | 0,428317 | 33,34 | 23,78 | 9,57 | 9,35 | 0,54 |  |  |
| R2hCSR2 | 2 | 2 | 0.5% DMSO | 4 | NA | 33,64 | 1,214619 | 33,64 | 24,22 | 9,42 | 9,35 | 0,54 |  |  |
| R4hCSR1 | 1 | 2 | 0.5% DMSO | 6 | NA | 33,24 | 0,046233 | 33,24 | 23,48 | 9,76 | 9,35 | 0,54 |  |  |
| R4hCSR2 | 2 | 2 | 0.5% DMSO | 6 | NA | 33,50 | 0,16116 | 33,50 | 24,38 | 9,12 | 9,35 | 0,54 |  |  |
| R8hCSR1 | 1 | 2 | 0.5% DMSO | 10 | NA | 33,39 | 0,446916 | 33,39 | 24,87 | 8,52 | 9,35 | 0,54 |  |  |
| R8hCSR2 | 2 | 2 | 0.5% DMSO | 10 | NA | 33,84 | 0,89637 | 33,84 | 24,70 | 9,14 | 9,35 | 0,54 |  |  |
| RC16h0.1R1 | 1 | 2 | 0.1 µM 20E | 18 | NA | 28,06 | 0,004555 | 28,06 | 22,16 | 5,90 | 9,35 | 0,54 | -3,45 | 10,95 |
| RC16h0.1R2 | 2 | 2 | 0.1 µM 20E | 18 | NA | 29,04 | 0,219138 | 29,04 | 23,62 | 5,42 | 9,35 | 0,54 | -3,93 | 15,26 |
| RC1h0.1R1 | 1 | 2 | 0.1 µM 20E | 3 | NA | 27,24 | 0,075657 | 27,24 | 22,19 | 5,05 | 9,35 | 0,54 | -4,30 | 19,63 |
| RC1h0.1R2 | 2 | 2 | 0.1 µM 20E | 3 | NA | 27,86 | 0,06139 | 27,86 | 23,12 | 4,74 | 9,35 | 0,54 | -4,61 | 24,38 |
| RC2h0.1R1 | 1 | 2 | 0.1 µM 20E | 4 | NA | 26,05 | 0,071124 | 26,05 | 21,56 | 4,49 | 9,35 | 0,54 | -4,86 | 28,96 |
| RC2h0.1R2 | 2 | 2 | 0.1 µM 20E | 4 | NA | 27,59 | 0,073724 | 27,59 | 22,78 | 4,81 | 9,35 | 0,54 | -4,54 | 23,33 |
| RC4h0.1R1 | 1 | 2 | 0.1 µM 20E | 6 | NA | 26,78 | 0,096691 | 26,78 | 22,27 | 4,51 | 9,35 | 0,54 | -4,84 | 28,57 |
| RC4h0.1R2 | 2 | 2 | 0.1 µM 20E | 6 | NA | 28,17 | 0,072284 | 28,17 | 23,32 | 4,84 | 9,35 | 0,54 | -4,51 | 22,76 |
| RC8h0.1R1 | 1 | 2 | 0.1 µM 20E | 10 | NA | 27,25 | 0,236398 | 27,25 | 22,35 | 4,90 | 9,35 | 0,54 | -4,45 | 21,87 |
| RC8h0.1R2 | 2 | 2 | 0.1 µM 20E | 10 | NA | 28,02 | 0,042607 | 28,02 | 23,23 | 4,79 | 9,35 | 0,54 | -4,56 | 23,59 |
| RR16h0.1R1 | 1 | 2 | 0.1 µM 20E | 2 | 16 | 33,15 | 0,958764 | 33,15 | 22,12 | 11,03 | 9,35 | 0,54 | 1,68 | 0,31 |
| RR16h0.1R2 | 2 | 2 | 0.1 µM 20E | 2 | 16 | 32,15 | 0,131955 | 32,15 | 22,14 | 10,01 | 9,35 | 0,54 | 0,66 | 0,63 |
| RR1h0.1R1 | 1 | 2 | 0.1 µM 20E | 2 | 1 | 28,72 | 0,240093 | 28,72 | 22,71 | 6,01 | 9,35 | 0,54 | -3,34 | 10,14 |
| RR1h0.1R2 | 2 | 2 | 0.1 µM 20E | 2 | 1 | 29,03 | 0,32486 | 29,03 | 23,30 | 5,73 | 9,35 | 0,54 | -3,62 | 12,30 |
| RR2h0.1R1 | 1 | 2 | 0.1 µM 20E | 2 | 2 | 31,34 | 0,061069 | 31,34 | 22,39 | 8,94 | 9,35 | 0,54 | -0,41 | 1,33 |
| RR2h0.1R2 | 2 | 2 | 0.1 µM 20E | 2 | 2 | 31,86 | 0,348743 | 31,86 | 22,18 | 9,68 | 9,35 | 0,54 | 0,33 | 0,79 |
| RR4h0.1R1 | 1 | 2 | 0.1 µM 20E | 2 | 4 | 33,43 | 0,361227 | 33,43 | 22,29 | 11,14 | 9,35 | 0,54 | 1,79 | 0,29 |
| RR4h0.1R2 | 2 | 2 | 0.1 µM 20E | 2 | 4 | 32,97 | 0,026708 | 32,97 | 22,68 | 10,29 | 9,35 | 0,54 | 0,94 | 0,52 |
| RR8h0.1R1 | 1 | 2 | 0.1 µM 20E | 2 | 8 | 31,94 | 0,605561 | 31,94 | 22,19 | 9,75 | 9,35 | 0,54 | 0,40 | 0,76 |
| RR8h0.1R2 | 2 | 2 | 0.1 µM 20E | 2 | 8 | 31,59 | 0,648923 | 31,59 | 22,25 | 9,35 | 9,35 | 0,54 | 0,00 | 1,00 |
| PR1h0.1R1 | 1 | 3 | 0.1 µM 20E | 1 | NA | 27,47405 | 0,161336 | 27,47 | 21,97 | 5,50 | 9,44 | 0,815604 | -3,94 | 15,32 |
| PR1h0.1R2 | 2 | 3 | 0.1 µM 20E | 1 | NA | 27,46005 | 0,192787 | 27,46 | 22,33 | 5,13 | 9,44 | 0,815604 | -4,31 | 19,85 |
| PR1hCSR2 | 2 | 3 | 0.1 µM 20E | 1 | NA | 32,63119 | 0,108145 | 32,63 | 23,56 | 9,07 | 9,44 | 0,815604 |  |  |
| PR2h0.1R1 | 1 | 3 | 0.1 µM 20E | 2 | NA | 27,60183 | 0,052781 | 27,60 | 22,26 | 5,35 | 9,44 | 0,815604 | -4,09 | 17,06 |
| PR2h0.1R2 | 2 | 3 | 0.1 µM 20E | 2 | NA | 30,14479 | 0,06819 | 30,14 | 25,74 | 4,41 | 9,44 | 0,815604 | -5,03 | 32,68 |
| PR2hCSR1 | 1 | 3 | 0.5% DMSO | 2 | NA | 31,75633 | 0,420493 | 31,76 | 22,07 | 9,68 | 9,44 | 0,815604 |  |  |
| PR2hCSR2 | 2 | 3 | 0.5% DMSO | 2 | NA | 32,02156 | 0,345699 | 32,02 | 23,31 | 8,71 | 9,44 | 0,815604 |  |  |
| PR30min0.1R1 | 1 | 3 | 0.1 µM 20E | 0,5 | NA | 29,32433 | 0,135419 | 29,32 | 22,76 | 6,56 | 9,44 | 0,815604 | -2,88 | 7,36 |
| PR30min0.1R2 | 2 | 3 | 0.1 µM 20E | 0,5 | NA | 29,53718 | 0,215747 | 29,54 | 22,46 | 7,08 | 9,44 | 0,815604 | -2,36 | 5,13 |
| PR30minCSR1 | 1 | 3 | 0.5% DMSO | 0,5 | NA | 32,54051 | 0,624387 | 32,54 | 22,46 | 10,08 | 9,44 | 0,815604 |  |  |
| PR30minCSR2 | 2 | 3 | 0.5% DMSO | 0,5 | NA | 32,51592 | 0,176303 | 32,52 | 22,90 | 9,61 | 9,44 | 0,815604 |  |  |
| R16hCSR1 | 1 | 3 | 0.5% DMSO | 18 | NA | 35,9885 | 0,73754 | 35,00 | 24,75 | 10,25 | 9,44 | 0,815604 |  |  |
| R16hCSR2 | 2 | 3 | 0.5% DMSO | 18 | NA | 35,38612 | 1,042506 | 35,00 | 24,29 | 10,71 | 9,44 | 0,815604 |  |  |
| R1hCSR1 | 1 | 3 | 0.5% DMSO | 3 | NA | 32,8376 | 0,147428 | 32,84 | 25,73 | 7,11 | 9,44 | 0,815604 |  |  |
| R1hCSR2 | 2 | 3 | 0.5% DMSO | 3 | NA | 33,10885 | 0,348881 | 33,11 | 24,39 | 8,72 | 9,44 | 0,815604 |  |  |
| R2hCSR1 | 1 | 3 | 0.5% DMSO | 4 | NA | 33,97212 | 1,002197 | 33,97 | 23,98 | 9,99 | 9,44 | 0,815604 |  |  |
| R2hCSR2 | 2 | 3 | 0.5% DMSO | 4 | NA | 34,21597 | 0,064268 | 34,22 | 24,99 | 9,23 | 9,44 | 0,815604 |  |  |
| R4hCSR1 | 1 | 3 | 0.5% DMSO | 6 | NA | 33,62453 | 0,419295 | 33,62 | 23,99 | 9,63 | 9,44 | 0,815604 |  |  |
| R4hCSR2 | 2 | 3 | 0.5% DMSO | 6 | NA | 32,72761 | 0,534812 | 32,73 | 22,87 | 9,86 | 9,44 | 0,815604 |  |  |
| R8hCSR1 | 1 | 3 | 0.5% DMSO | 10 | NA | 33,82583 | 1,16366 | 33,83 | 24,48 | 9,34 | 9,44 | 0,815604 |  |  |
| R8hCSR2 | 2 | 3 | 0.5% DMSO | 10 | NA | 32,91838 | 0,490009 | 32,92 | 23,35 | 9,57 | 9,44 | 0,815604 |  |  |
| RC16h0.1R1 | 1 | 3 | 0.1 µM 20E | 18 | NA | 29,31669 | 0,146952 | 29,32 | 23,33 | 5,98 | 9,44 | 0,815604 | -3,46 | 10,98 |
| RC16h0.1R2 | 2 | 3 | 0.1 µM 20E | 18 | NA | 29,55932 | 0,075103 | 29,56 | 23,62 | 5,94 | 9,44 | 0,815604 | -3,49 | 11,27 |
| RC1h0.1R1 | 1 | 3 | 0.1 µM 20E | 3 | NA | 29,61592 | 0,407856 | 29,62 | 24,39 | 5,22 | 9,44 | 0,815604 | -4,21 | 18,56 |
| RC1h0.1R2 | 2 | 3 | 0.1 µM 20E | 3 | NA | 28,57615 | 0,206044 | 28,58 | 24,50 | 4,07 | 9,44 | 0,815604 | -5,37 | 41,22 |
| RC2h0.1R1 | 1 | 3 | 0.1 µM 20E | 4 | NA | 29,0548 | 0,388251 | 29,05 | 23,75 | 5,30 | 9,44 | 0,815604 | -4,14 | 17,59 |
| RC2h0.1R2 | 2 | 3 | 0.1 µM 20E | 4 | NA | 29,43321 | 0,021199 | 29,43 | 24,14 | 5,30 | 9,44 | 0,815604 | -4,14 | 17,64 |
| RC4h0.1R1 | 1 | 3 | 0.1 µM 20E | 6 | NA | 28,58917 | 0,404187 | 28,59 | 23,95 | 4,64 | 9,44 | 0,815604 | -4,80 | 27,81 |
| RC4h0.1R2 | 2 | 3 | 0.1 µM 20E | 6 | NA | 28,97107 | 0,036681 | 28,97 | 24,73 | 4,24 | 9,44 | 0,815604 | -5,20 | 36,69 |
| RC8h0.1R1 | 1 | 3 | 0.1 µM 20E | 10 | NA | 28,85996 | 0,133858 | 28,86 | 23,34 | 5,52 | 9,44 | 0,815604 | -3,92 | 15,09 |
| RC8h0.1R2 | 2 | 3 | 0.1 µM 20E | 10 | NA | 29,40354 | 0,001236 | 29,40 | 24,74 | 4,66 | 9,44 | 0,815604 | -4,78 | 27,45 |
| RR16h0.1R1 | 1 | 3 | 0.1 µM 20E | 2 | 16 | 33,68323 | 0,530194 | 33,68 | 23,20 | 10,48 | 9,44 | 0,815604 | 1,04 | 0,49 |
| RR16h0.1R2 | 2 | 3 | 0.1 µM 20E | 2 | 16 | 33,41678 | 0,395478 | 33,42 | 22,12 | 11,29 | 9,44 | 0,815604 | 1,86 | 0,28 |
| RR1h0.1R1 | 1 | 3 | 0.1 µM 20E | 2 | 1 | 29,94814 | 0,200843 | 29,95 | 22,63 | 7,31 | 9,44 | 0,815604 | -2,12 | 4,36 |
| RR1h0.1R2 | 2 | 3 | 0.1 µM 20E | 2 | 1 | 30,25144 | 0,068708 | 30,25 | 23,47 | 6,78 | 9,44 | 0,815604 | -2,66 | 6,31 |
| RR2h0.1R1 | 1 | 3 | 0.1 µM 20E | 2 | 2 | 33,22715 | 0,16049 | 33,23 | 23,17 | 10,06 | 9,44 | 0,815604 | 0,62 | 0,65 |
| RR2h0.1R2 | 2 | 3 | 0.1 µM 20E | 2 | 2 | 33,69361 | 0,892622 | 33,69 | 23,35 | 10,35 | 9,44 | 0,815604 | 0,91 | 0,53 |
| RR4h0.1R1 | 1 | 3 | 0.1 µM 20E | 2 | 4 | 34,66827 | 0,493123 | 34,67 | 23,54 | 11,12 | 9,44 | 0,815604 | 1,69 | 0,31 |
| RR4h0.1R2 | 2 | 3 | 0.1 µM 20E | 2 | 4 | 34,09393 | 0,799685 | 34,09 | 23,57 | 10,53 | 9,44 | 0,815604 | 1,09 | 0,47 |
| RR8h0.1R1 | 1 | 3 | 0.1 µM 20E | 2 | 8 | 34,01123 | 0,289881 | 34,01 | 23,14 | 10,87 | 9,44 | 0,815604 | 1,44 | 0,37 |
| RR8h0.1R2 | 2 | 3 | 0.1 µM 20E | 2 | 8 | 32,87639 | 0,420687 | 32,88 | 22,81 | 10,06 | 9,44 | 0,815604 | 0,62 | 0,65 |

| sample id | replicate | experiment number | Treatment | exposure time/hours | recovery time/hours | Avg Cq | st.dev Cq | corrected Cq (max cq 35) | Refrence gene Cq | delta Cq | Avg. solvent control delta Cq | st.dev solvent control delta Cq | delta delta Cq | 2^ddCq |
| --- | --- | --- | --- | --- | --- | --- | --- | --- | --- | --- | --- | --- | --- | --- |
| PR1hA0.1R1 | 1 | 1 | 0.1 µM 20E | 1 | NA | 26,13 | 0,02089 | 28,92 | 22,93 | 5,99 | 9,30 | 0,53 | -1,36 | 2,57 |
| PR1hA0.1R2 | 2 | 1 | 0.1 µM 20E | 1 | NA | 28,26 | 0,00129 | 28,43 | 22,22 | 6,20 | 9,30 | 0,53 | -1,04 | 2,06 |
| PR1hA0.1R3 | 3 | 1 | 0.1 µM 20E | 1 | NA | 36,54 | 1,24381 | 32,74 | 23,32 | 9,41 | 9,30 | 0,53 | -1,81 | 3,50 |
| PR1hB0.1R1 | 1 | 1 | 0.1 µM 20E | 1 | NA | 29,57 | 0,14238 | 30,16 | 23,19 | 6,97 | 9,30 | 0,53 |  |  |
| PR1hB0.1R2 | 2 | 1 | 0.1 µM 20E | 1 | NA | 28,76 | 0,38295 | 27,73 | 21,68 | 6,05 | 9,30 | 0,53 |  |  |
| PR1hB0.1R3 | 3 | 1 | 0.1 µM 20E | 1 | NA | 36,57 | 0,16753 | 32,64 | 23,52 | 9,11 | 9,30 | 0,53 |  |  |
| PR1hCSR1 | 1 | 1 | 0.1 µM 20E | 1 | NA | 37,17 | 0,00000 | 31,93 | 23,11 | 8,82 | 9,30 | 0,53 |  |  |
| PR1hCSR2 | 2 | 1 | 0.1 µM 20E | 1 | NA | 35,34 | 1,63820 | 31,58 | 22,75 | 8,83 | 9,30 | 0,53 |  |  |
| PR1hCSR3 | 3 | 1 | 0.1 µM 20E | 1 | NA | 33,22 | 1,84902 | 33,82 | 24,51 | 9,31 | 9,30 | 0,53 |  |  |
| PR2hA0.1R1 | 1 | 1 | 0.1 µM 20E | 2 | NA | 26,03 | 0,09343 | 29,58 | 23,76 | 5,82 | 9,30 | 0,53 | -2,82 | 7,04 |
| PR2hA0.1R2 | 2 | 1 | 0.1 µM 20E | 2 | NA | 28,49 | 0,03464 | 27,83 | 22,98 | 4,85 | 9,30 | 0,53 | -3,17 | 9,00 |
| PR2hA0.1R3 | 3 | 1 | 0.1 µM 20E | 2 | NA | 29,40 | 0,30653 | 28,66 | 23,04 | 5,62 | 9,30 | 0,53 | -0,03 | 1,02 |
| PR2hB0.1R1 | 1 | 1 | 0.1 µM 20E | 2 | NA | 28,77 | 0,34167 | 29,65 | 23,57 | 6,08 | 9,30 | 0,53 |  |  |
| PR2hB0.1R2 | 2 | 1 | 0.1 µM 20E | 2 | NA | 28,28 | 0,31111 | 27,06 | 22,08 | 4,98 | 9,30 | 0,53 |  |  |
| PR2hB0.1R3 | 3 | 1 | 0.1 µM 20E | 2 | NA | 30,36 | 0,67995 | 28,76 | 22,55 | 6,21 | 9,30 | 0,53 |  |  |
| PR2hCSR1 | 1 | 1 | 0.5% DMSO | 2 | NA | 34,87 | 1,04142 | 32,54 | 22,61 | 9,93 | 9,30 | 0,53 |  |  |
| PR2hCSR2 | 2 | 1 | 0.5% DMSO | 2 | NA | 36,54 | 0,00000 | 31,34 | 23,15 | 8,19 | 9,30 | 0,53 |  |  |
| PR2hCSR3 | 3 | 1 | 0.5% DMSO | 2 | NA | 33,75 | 0,04970 | 33,39 | 24,44 | 8,95 | 9,30 | 0,53 |  |  |
| PR30minA0.1R1 | 1 | 1 | 0.1 µM 20E | 0,5 | NA | 26,71 | 0,26915 | 30,01 | 22,99 | 7,03 | 9,30 | 0,53 | -3,34 | 10,16 |
| PR30minA0.1R2 | 2 | 1 | 0.1 µM 20E | 0,5 | NA | 30,46 | 0,05462 | 30,18 | 22,42 | 7,76 | 9,30 | 0,53 | -4,38 | 20,86 |
| PR30minA0.1R3 | 3 | 1 | 0.1 µM 20E | 0,5 | NA | 36,15 | 0,00000 | 32,39 | 23,41 | 8,98 | 9,30 | 0,53 | -3,38 | 10,42 |
| PR30minB0.1R1 | 1 | 1 | 0.1 µM 20E | 0,5 | NA | 30,70 | 0,50500 | 31,02 | 22,18 | 8,84 | 9,30 | 0,53 |  |  |
| PR30minB0.1R2 | 2 | 1 | 0.1 µM 20E | 0,5 | NA | 30,88 | 0,16317 | 30,92 | 22,18 | 8,74 | 9,30 | 0,53 |  |  |
| PR30minB0.1R3 | 3 | 1 | 0.1 µM 20E | 0,5 | NA | 35,39 | 0,00000 | 32,21 | 22,87 | 9,34 | 9,30 | 0,53 |  |  |
| PR30minCSR1 | 1 | 1 | 0.5% DMSO | 0,5 | NA | 34,36 | 0,00000 | 31,97 | 22,91 | 9,06 | 9,30 | 0,53 |  |  |
| PR30minCSR2 | 2 | 1 | 0.5% DMSO | 0,5 | NA | 35,25 | 0,99541 | 32,75 | 23,39 | 9,35 | 9,30 | 0,53 |  |  |
| PR30minCSR3 | 3 | 1 | 0.5% DMSO | 0,5 | NA | 33,96 | 1,27104 | 33,05 | 24,51 | 8,54 | 9,30 | 0,53 |  |  |
| R16hCSR1 | 1 | 1 | 0.5% DMSO | 18 | NA | 34,34 | 0,00000 | 32,39 | 22,81 | 9,58 | 9,30 | 0,53 |  |  |
| R16hCSR2 | 2 | 1 | 0.5% DMSO | 18 | NA | 35,34 | 0,00000 | 32,06 | 23,00 | 9,07 | 9,30 | 0,53 |  |  |
| R16hCSR3 | 3 | 1 | 0.5% DMSO | 18 | NA | 32,14 | 0,09990 | 32,63 | 23,16 | 9,48 | 9,30 | 0,53 |  |  |
| R1hCSR1 | 1 | 1 | 0.5% DMSO | 3 | NA | 33,33 | 1,23050 | 32,35 | 22,76 | 9,60 | 9,30 | 0,53 |  |  |
| R1hCSR2 | 2 | 1 | 0.5% DMSO | 3 | NA | 35,51 | 0,00000 | 31,95 | 22,89 | 9,06 | 9,30 | 0,53 |  |  |
| R1hCSR3 | 3 | 1 | 0.5% DMSO | 3 | NA | 33,35 | 0,45068 | 33,50 | 24,28 | 9,22 | 9,30 | 0,53 |  |  |
| R2hCSR1 | 1 | 1 | 0.5% DMSO | 4 | NA | 33,85 | 0,24336 | 31,93 | 22,67 | 9,26 | 9,30 | 0,53 |  |  |
| R2hCSR2 | 2 | 1 | 0.5% DMSO | 4 | NA | 35,98 | 0,66578 | 31,64 | 23,06 | 8,58 | 9,30 | 0,53 |  |  |
| R2hCSR3 | 3 | 1 | 0.5% DMSO | 4 | NA | 33,08 | 1,03187 | 32,99 | 23,76 | 9,23 | 9,30 | 0,53 |  |  |
| R4hCSR1 | 1 | 1 | 0.5% DMSO | 6 | NA | 32,96 | 0,13681 | 33,71 | 23,19 | 10,52 | 9,30 | 0,53 |  |  |
| R4hCSR2 | 2 | 1 | 0.5% DMSO | 6 | NA | 36,70 | 0,17962 | 32,26 | 22,47 | 9,79 | 9,30 | 0,53 |  |  |
| R4hCSR3 | 3 | 1 | 0.5% DMSO | 6 | NA | 30,93 | 0,80456 | 32,91 | 23,67 | 9,24 | 9,30 | 0,53 |  |  |
| R8hCSR1 | 1 | 1 | 0.5% DMSO | 10 | NA | 34,46 | 0,81036 | 32,52 | 23,18 | 9,34 | 9,30 | 0,53 |  |  |
| R8hCSR2 | 2 | 1 | 0.5% DMSO | 10 | NA | 35,35 | 0,00000 | 32,84 | 22,63 | 10,22 | 9,30 | 0,53 |  |  |
| R8hCSR3 | 3 | 1 | 0.5% DMSO | 10 | NA | 35,12 | 2,98357 | 33,34 | 23,41 | 9,92 | 9,30 | 0,53 |  |  |
| RC16h0.1R1 | 1 | 1 | 0.1 µM 20E | 18 | NA | 29,08 | 0,14463 | 27,72 | 23,33 | 4,40 | 9,30 | 0,53 | -4,90 | 29,84 |
| RC16h0.1R2 | 2 | 1 | 0.1 µM 20E | 18 | NA | 30,76 | 0,39342 | 27,57 | 22,77 | 4,80 | 9,30 | 0,53 | -4,49 | 22,50 |
| RC16h0.1R3 | 3 | 1 | 0.1 µM 20E | 18 | NA | 28,04 | 0,03846 | 28,60 | 22,51 | 6,08 | 9,30 | 0,53 | -3,21 | 9,27 |
| RC1h0.1R1 | 1 | 1 | 0.1 µM 20E | 3 | NA | 29,25 | 0,09986 | 28,54 | 23,01 | 5,52 | 9,30 | 0,53 | -3,77 | 13,68 |
| RC1h0.1R2 | 2 | 1 | 0.1 µM 20E | 3 | NA | 30,06 | 0,33561 | 28,02 | 22,05 | 5,97 | 9,30 | 0,53 | -3,33 | 10,02 |
| RC1h0.1R3 | 3 | 1 | 0.1 µM 20E | 3 | NA | 31,64 | 0,66578 | 29,44 | 23,25 | 6,18 | 9,30 | 0,53 | -3,11 | 8,64 |
| RC2h0.1R1 | 1 | 1 | 0.1 µM 20E | 4 | NA | 28,92 | 0,60873 | 29,06 | 22,91 | 6,14 | 9,30 | 0,53 | -3,15 | 8,88 |
| RC2h0.1R2 | 2 | 1 | 0.1 µM 20E | 4 | NA | 29,12 | 0,06975 | 28,06 | 22,41 | 5,65 | 9,30 | 0,53 | -3,64 | 12,49 |
| RC2h0.1R3 | 3 | 1 | 0.1 µM 20E | 4 | NA | 28,84 | 0,65579 | 28,90 | 22,50 | 6,40 | 9,30 | 0,53 | -2,89 | 7,43 |
| RC4h0.1R1 | 1 | 1 | 0.1 µM 20E | 6 | NA | 28,53 | 0,64253 | 29,24 | 23,01 | 6,23 | 9,30 | 0,53 | -3,07 | 8,39 |
| RC4h0.1R2 | 2 | 1 | 0.1 µM 20E | 6 | NA | 29,73 | 0,00367 | 27,88 | 22,16 | 5,72 | 9,30 | 0,53 | -3,58 | 11,96 |
| RC4h0.1R3 | 3 | 1 | 0.1 µM 20E | 6 | NA | 30,99 | 0,65219 | 28,68 | 22,34 | 6,34 | 9,30 | 0,53 | -2,95 | 7,73 |
| RC8h0.1R1 | 1 | 1 | 0.1 µM 20E | 10 | NA | 29,12 | 0,49696 | 29,22 | 23,67 | 5,55 | 9,30 | 0,53 | -3,75 | 13,41 |
| RC8h0.1R2 | 2 | 1 | 0.1 µM 20E | 10 | NA | 27,61 | 0,01646 | 28,12 | 22,44 | 5,68 | 9,30 | 0,53 | -3,62 | 12,26 |
| RC8h0.1R3 | 3 | 1 | 0.1 µM 20E | 10 | NA | 31,02 | 0,91758 | 29,18 | 22,64 | 6,54 | 9,30 | 0,53 | -2,75 | 6,74 |
| RR16h0.1R1 | 1 | 1 | 0.1 µM 20E | 2 | 16 | 34,70 | 0,28067 | 31,47 | 22,23 | 9,24 | 9,30 | 0,53 | -0,05 | 1,04 |
| RR16h0.1R2 | 2 | 1 | 0.1 µM 20E | 2 | 16 | 36,68 | 1,40401 | 32,25 | 23,09 | 9,16 | 9,30 | 0,53 | -0,13 | 1,10 |
| RR16h0.1R3 | 3 | 1 | 0.1 µM 20E | 2 | 16 | 33,38 | 0,15175 | 33,55 | 23,50 | 10,05 | 9,30 | 0,53 | 0,75 | 0,59 |
| RR1h0.1R1 | 1 | 1 | 0.1 µM 20E | 2 | 1 | 29,93 | 0,14108 | 29,66 | 23,40 | 6,26 | 9,30 | 0,53 | -3,03 | 8,19 |
| RR1h0.1R2 | 2 | 1 | 0.1 µM 20E | 2 | 1 | 31,39 | 0,02609 | 28,87 | 23,29 | 5,58 | 9,30 | 0,53 | -3,71 | 13,13 |
| RR1h0.1R3 | 3 | 1 | 0.1 µM 20E | 2 | 1 | 33,06 | 0,00561 | 31,13 | 24,07 | 7,05 | 9,30 | 0,53 | -2,24 | 4,73 |
| RR2h0.1R1 | 1 | 1 | 0.1 µM 20E | 2 | 2 | 29,19 | 0,22069 | 30,89 | 23,44 | 7,45 | 9,30 | 0,53 | -1,84 | 3,59 |
| RR2h0.1R2 | 2 | 1 | 0.1 µM 20E | 2 | 2 | 32,43 | 0,06168 | 29,46 | 22,61 | 6,85 | 9,30 | 0,53 | -2,44 | 5,44 |
| RR2h0.1R3 | 3 | 1 | 0.1 µM 20E | 2 | 2 | 32,55 | 0,25455 | 30,91 | 23,38 | 7,53 | 9,30 | 0,53 | -1,76 | 3,39 |
| RR4h0.1R1 | 1 | 1 | 0.1 µM 20E | 2 | 4 | 30,83 | 0,52069 | 32,12 | 23,54 | 8,58 | 9,30 | 0,53 | -0,71 | 1,64 |
| RR4h0.1R2 | 2 | 1 | 0.1 µM 20E | 2 | 4 | 34,34 | 0,54199 | 30,94 | 23,13 | 7,81 | 9,30 | 0,53 | -1,48 | 2,79 |
| RR4h0.1R3 | 3 | 1 | 0.1 µM 20E | 2 | 4 | 34,31 | 1,14148 | 31,50 | 23,16 | 8,34 | 9,30 | 0,53 | -0,95 | 1,94 |
| RR8h0.1R1 | 1 | 1 | 0.1 µM 20E | 2 | 8 | 30,80 | 0,96806 | 33,04 | 23,18 | 9,86 | 9,30 | 0,53 | 0,56 | 0,68 |
| RR8h0.1R2 | 2 | 1 | 0.1 µM 20E | 2 | 8 | 33,88 | 0,36599 | 32,96 | 23,62 | 9,34 | 9,30 | 0,53 | 0,05 | 0,97 |
| RR8h0.1R3 | 3 | 1 | 0.1 µM 20E | 2 | 8 | 35,46 | 0,00000 | 33,91 | 23,41 | 10,51 | 9,30 | 0,53 | 1,21 | 0,43 |
| PR1h0.1R1 | 1 | 2 | 0.1 µM 20E | 1 | NA | 30,12 | 0,0230 | 30,12 | 22,96 | 7,16 | 10,23 | 0,72015 | -3,07 | 8,40 |
| PR1h0.1R2 | 2 | 2 | 0.1 µM 20E | 1 | NA | 30,03 | 0,0643 | 30,03 | 22,52 | 7,52 | 10,23 | 0,72015 | -2,71 | 6,56 |
| PR1hCSR1 | 1 | 2 | 0.1 µM 20E | 1 | NA | 34,00 | 0,0025 | 34,00 | 22,69 | 11,32 | 10,23 | 0,72015 |  |  |
| PR1hCSR2 | 2 | 2 | 0.1 µM 20E | 1 | NA | 35,46 | 1,1007 | 35,00 | 24,71 | 10,29 | 10,23 | 0,72015 |  |  |
| PR2h0.1R1 | 1 | 2 | 0.1 µM 20E | 2 | NA | 28,89 | 0,1766 | 28,89 | 22,57 | 6,32 | 10,23 | 0,72015 | -3,91 | 15,07 |
| PR2h0.1R2 | 2 | 2 | 0.1 µM 20E | 2 | NA | 29,23 | 0,0555 | 29,23 | 22,66 | 6,57 | 10,23 | 0,72015 | -3,66 | 12,61 |
| PR2hCSR1 | 1 | 2 | 0.5% DMSO | 2 | NA | 33,70 | 0,1223 | 33,70 | 23,09 | 10,61 | 10,23 | 0,72015 |  |  |
| PR2hCSR2 | 2 | 2 | 0.5% DMSO | 2 | NA | 34,45 | 0,0898 | 34,45 | 23,49 | 10,96 | 10,23 | 0,72015 |  |  |
| PR30min0.1R1 | 1 | 2 | 0.1 µM 20E | 0,5 | NA | 32,55 | 0,2841 | 32,55 | 22,85 | 9,70 | 10,23 | 0,72015 | -0,53 | 1,44 |
| PR30min0.1R2 | 2 | 2 | 0.1 µM 20E | 0,5 | NA | 32,57 | 0,6949 | 32,57 | 23,39 | 9,18 | 10,23 | 0,72015 | -1,05 | 2,06 |
| PR30minCSR1 | 1 | 2 | 0.5% DMSO | 0,5 | NA | 33,80 | 0,4108 | 33,80 | 22,62 | 11,17 | 10,23 | 0,72015 |  |  |
| PR30minCSR2 | 2 | 2 | 0.5% DMSO | 0,5 | NA | 34,42 | 0,0000 | 34,42 | 24,49 | 9,93 | 10,23 | 0,72015 |  |  |
| R16hCSR1 | 1 | 2 | 0.5% DMSO | 18 | NA | 34,26 | 0,1071 | 34,26 | 23,74 | 10,52 | 10,23 | 0,72015 |  |  |
| R16hCSR2 | 2 | 2 | 0.5% DMSO | 18 | NA | 34,57 | 0,3996 | 34,57 | 23,99 | 10,58 | 10,23 | 0,72015 |  |  |
| R1hCSR1 | 1 | 2 | 0.5% DMSO | 3 | NA | 33,57 | 0,8439 | 33,57 | 24,40 | 9,17 | 10,23 | 0,72015 |  |  |
| R1hCSR2 | 2 | 2 | 0.5% DMSO | 3 | NA | 35,68 | 0,6238 | 35,00 | 23,77 | 11,23 | 10,23 | 0,72015 |  |  |
| R2hCSR1 | 1 | 2 | 0.5% DMSO | 4 | NA | 33,78 | 0,0973 | 33,78 | 23,78 | 10,01 | 10,23 | 0,72015 |  |  |
| R2hCSR2 | 2 | 2 | 0.5% DMSO | 4 | NA | 33,84 | 0,6684 | 33,84 | 24,22 | 9,62 | 10,23 | 0,72015 |  |  |
| R4hCSR1 | 1 | 2 | 0.5% DMSO | 6 | NA | 33,67 | 0,1685 | 33,67 | 23,48 | 10,19 | 10,23 | 0,72015 |  |  |
| R4hCSR2 | 2 | 2 | 0.5% DMSO | 6 | NA | 34,22 | 0,0000 | 34,22 | 24,38 | 9,84 | 10,23 | 0,72015 |  |  |
| R8hCSR1 | 1 | 2 | 0.5% DMSO | 10 | NA | 33,80 | 0,5268 | 33,80 | 24,87 | 8,92 | 10,23 | 0,72015 |  |  |
| R8hCSR2 | 2 | 2 | 0.5% DMSO | 10 | NA | 34,02 | 0,3651 | 34,02 | 24,70 | 9,32 | 10,23 | 0,72015 |  |  |
| RC16h0.1R1 | 1 | 2 | 0.1 µM 20E | 18 | NA | 29,17 | 0,3555 | 29,17 | 22,16 | 7,01 | 10,23 | 0,72015 | -3,22 | 9,32 |
| RC16h0.1R2 | 2 | 2 | 0.1 µM 20E | 18 | NA | 30,53 | 0,2896 | 30,53 | 23,62 | 6,91 | 10,23 | 0,72015 | -3,32 | 9,96 |
| RC1h0.1R1 | 1 | 2 | 0.1 µM 20E | 3 | NA | 28,64 | 0,4001 | 28,64 | 22,19 | 6,45 | 10,23 | 0,72015 | -3,78 | 13,70 |
| RC1h0.1R2 | 2 | 2 | 0.1 µM 20E | 3 | NA | 29,36 | 0,1725 | 29,36 | 23,12 | 6,24 | 10,23 | 0,72015 | -3,99 | 15,92 |
| RC2h0.1R1 | 1 | 2 | 0.1 µM 20E | 4 | NA | 27,86 | 0,1197 | 27,86 | 21,56 | 6,30 | 10,23 | 0,72015 | -3,93 | 15,29 |
| RC2h0.1R2 | 2 | 2 | 0.1 µM 20E | 4 | NA | 29,32 | 0,1608 | 29,32 | 22,78 | 6,54 | 10,23 | 0,72015 | -3,69 | 12,94 |
| RC4h0.1R1 | 1 | 2 | 0.1 µM 20E | 6 | NA | 29,07 | 0,0260 | 29,07 | 22,27 | 6,81 | 10,23 | 0,72015 | -3,42 | 10,73 |
| RC4h0.1R2 | 2 | 2 | 0.1 µM 20E | 6 | NA | 29,84 | 0,0543 | 29,84 | 23,32 | 6,51 | 10,23 | 0,72015 | -3,72 | 13,15 |
| RC8h0.1R1 | 1 | 2 | 0.1 µM 20E | 10 | NA | 28,94 | 0,1090 | 28,94 | 22,35 | 6,58 | 10,23 | 0,72015 | -3,64 | 12,51 |
| RC8h0.1R2 | 2 | 2 | 0.1 µM 20E | 10 | NA | 30,01 | 0,3487 | 30,01 | 23,23 | 6,78 | 10,23 | 0,72015 | -3,45 | 10,92 |
| RR16h0.1R1 | 1 | 2 | 0.1 µM 20E | 2 | 16 | 34,35 | 0,5800 | 34,35 | 22,12 | 12,23 | 10,23 | 0,72015 | 2,00 | 0,25 |
| RR16h0.1R2 | 2 | 2 | 0.1 µM 20E | 2 | 16 | 34,21 | 1,1157 | 34,21 | 22,14 | 12,07 | 10,23 | 0,72015 | 1,84 | 0,28 |
| RR1h0.1R1 | 1 | 2 | 0.1 µM 20E | 2 | 1 | 29,63 | 0,2270 | 29,63 | 22,71 | 6,92 | 10,23 | 0,72015 | -3,31 | 9,92 |
| RR1h0.1R2 | 2 | 2 | 0.1 µM 20E | 2 | 1 | 30,32 | 0,3348 | 30,32 | 23,30 | 7,02 | 10,23 | 0,72015 | -3,21 | 9,27 |
| RR2h0.1R1 | 1 | 2 | 0.1 µM 20E | 2 | 2 | 30,64 | 0,1769 | 30,64 | 22,39 | 8,24 | 10,23 | 0,72015 | -1,99 | 3,96 |
| RR2h0.1R2 | 2 | 2 | 0.1 µM 20E | 2 | 2 | 30,61 | 0,0763 | 30,61 | 22,18 | 8,44 | 10,23 | 0,72015 | -1,79 | 3,47 |
| RR4h0.1R1 | 1 | 2 | 0.1 µM 20E | 2 | 4 | 33,23 | 1,1222 | 33,23 | 22,29 | 10,94 | 10,23 | 0,72015 | 0,71 | 0,61 |
| RR4h0.1R2 | 2 | 2 | 0.1 µM 20E | 2 | 4 | 33,36 | 0,9097 | 33,36 | 22,68 | 10,68 | 10,23 | 0,72015 | 0,45 | 0,73 |
| RR8h0.1R1 | 1 | 2 | 0.1 µM 20E | 2 | 8 | 33,40 | 0,9870 | 33,40 | 22,19 | 11,22 | 10,23 | 0,72015 | 0,99 | 0,50 |
| RR8h0.1R2 | 2 | 2 | 0.1 µM 20E | 2 | 8 | 33,94 | 0,7281 | 33,94 | 22,25 | 11,69 | 10,23 | 0,72015 | 1,46 | 0,36 |
| PR1h0.1R1 | 1 | 3 | 0.1 µM 20E | 1 | NA | 28,57161 | 0,297925 | 28,57 | 21,97 | 6,60 | 9,78 | 1E+00 | -3,18 | 9,09 |
| PR1h0.1R2 | 2 | 3 | 0.1 µM 20E | 1 | NA | 28,5714 | 0,082951 | 28,57 | 22,33 | 6,24 | 9,78 | 1E+00 | -3,55 | 11,67 |
| PR1hCSR2 | 2 | 3 | 0.1 µM 20E | 1 | NA | 32,43106 | 0,522628 | 32,43 | 23,56 | 8,87 | 9,78 | 1E+00 |  |  |
| PR2h0.1R1 | 1 | 3 | 0.1 µM 20E | 2 | NA | 28,07043 | 0,141378 | 28,07 | 22,26 | 5,81 | 9,78 | 1E+00 | -3,97 | 15,66 |
| PR2h0.1R2 | 2 | 3 | 0.1 µM 20E | 2 | NA | 30,30173 | 0,235915 | 30,30 | 25,74 | 4,57 | 9,78 | 1E+00 | -5,22 | 37,23 |
| PR2hCSR1 | 1 | 3 | 0.5% DMSO | 2 | NA | 33,15026 | 0,835679 | 33,15 | 22,07 | 11,08 | 9,78 | 1E+00 |  |  |
| PR2hCSR2 | 2 | 3 | 0.5% DMSO | 2 | NA | 32,13316 | 0,14068 | 32,13 | 23,31 | 8,82 | 9,78 | 1E+00 |  |  |
| PR30min0.1R1 | 1 | 3 | 0.1 µM 20E | 0,5 | NA | 30,25681 | 0,490997 | 30,26 | 22,76 | 7,49 | 9,78 | 1E+00 | -2,29 | 4,90 |
| PR30min0.1R2 | 2 | 3 | 0.1 µM 20E | 0,5 | NA | 30,68498 | 0,410326 | 30,68 | 22,46 | 8,23 | 9,78 | 1E+00 | -1,56 | 2,94 |
| PR30minCSR1 | 1 | 3 | 0.5% DMSO | 0,5 | NA | 33,63097 | 0,055064 | 33,63 | 22,46 | 11,17 | 9,78 | 1E+00 |  |  |
| PR30minCSR2 | 2 | 3 | 0.5% DMSO | 0,5 | NA | 32,86906 | 0,315588 | 32,87 | 22,90 | 9,97 | 9,78 | 1E+00 |  |  |
| R16hCSR1 | 1 | 3 | 0.5% DMSO | 18 | NA | 35,34696 | 1,405709 | 35,00 | 24,75 | 10,25 | 9,78 | 1E+00 |  |  |
| R16hCSR2 | 2 | 3 | 0.5% DMSO | 18 | NA |  | 0 | 35,00 | 24,29 | 10,71 | 9,78 | 1E+00 |  |  |
| R1hCSR1 | 1 | 3 | 0.5% DMSO | 3 | NA | 33,14162 | 0,834801 | 33,14 | 25,73 | 7,41 | 9,78 | 1E+00 |  |  |
| R1hCSR2 | 2 | 3 | 0.5% DMSO | 3 | NA | 32,54784 | 0 | 32,55 | 24,39 | 8,16 | 9,78 | 1E+00 |  |  |
| R2hCSR1 | 1 | 3 | 0.5% DMSO | 4 | NA | 32,78069 | 0,543518 | 32,78 | 23,98 | 8,80 | 9,78 | 1E+00 |  |  |
| R2hCSR2 | 2 | 3 | 0.5% DMSO | 4 | NA | 33,87288 | 0,172624 | 33,87 | 24,99 | 8,89 | 9,78 | 1E+00 |  |  |
| R4hCSR1 | 1 | 3 | 0.5% DMSO | 6 | NA | 35,12117 | 0 | 35,00 | 23,99 | 11,01 | 9,78 | 1E+00 |  |  |
| R4hCSR2 | 2 | 3 | 0.5% DMSO | 6 | NA | 33,18797 | 0,575219 | 33,19 | 22,87 | 10,32 | 9,78 | 1E+00 |  |  |
| R8hCSR1 | 1 | 3 | 0.5% DMSO | 10 | NA | 35,05814 | 2,283652 | 35,00 | 24,48 | 10,52 | 9,78 | 1E+00 |  |  |
| R8hCSR2 | 2 | 3 | 0.5% DMSO | 10 | NA | 34,12972 | 0,413333 | 34,13 | 23,35 | 10,78 | 9,78 | 1E+00 |  |  |
| RC16h0.1R1 | 1 | 3 | 0.1 µM 20E | 18 | NA | 30,06093 | 0,002521 | 30,06 | 23,33 | 6,73 | 9,78 | 1E+00 | -3,06 | 8,32 |
| RC16h0.1R2 | 2 | 3 | 0.1 µM 20E | 18 | NA | 30,02389 | 0,199576 | 30,02 | 23,62 | 6,41 | 9,78 | 1E+00 | -3,38 | 10,38 |
| RC1h0.1R1 | 1 | 3 | 0.1 µM 20E | 3 | NA | 29,2919 | 0,003439 | 29,29 | 24,39 | 4,90 | 9,78 | 1E+00 | -4,88 | 29,51 |
| RC1h0.1R2 | 2 | 3 | 0.1 µM 20E | 3 | NA | 28,67171 | 0,11774 | 28,67 | 24,50 | 4,17 | 9,78 | 1E+00 | -5,61 | 49,01 |
| RC2h0.1R1 | 1 | 3 | 0.1 µM 20E | 4 | NA | 29,43142 | 0,192494 | 29,43 | 23,75 | 5,68 | 9,78 | 1E+00 | -4,11 | 17,21 |
| RC2h0.1R2 | 2 | 3 | 0.1 µM 20E | 4 | NA | 29,2819 | 0,32445 | 29,28 | 24,14 | 5,15 | 9,78 | 1E+00 | -4,64 | 24,89 |
| RC4h0.1R1 | 1 | 3 | 0.1 µM 20E | 6 | NA | 29,52341 | 0,293434 | 29,52 | 23,95 | 5,58 | 9,78 | 1E+00 | -4,21 | 18,49 |
| RC4h0.1R2 | 2 | 3 | 0.1 µM 20E | 6 | NA | 29,96328 | 0,578434 | 29,96 | 24,73 | 5,23 | 9,78 | 1E+00 | -4,55 | 23,43 |
| RC8h0.1R1 | 1 | 3 | 0.1 µM 20E | 10 | NA | 29,7369 | 0,159884 | 29,74 | 23,34 | 6,40 | 9,78 | 1E+00 | -3,38 | 10,44 |
| RC8h0.1R2 | 2 | 3 | 0.1 µM 20E | 10 | NA | 30,23756 | 0,311388 | 30,24 | 24,74 | 5,49 | 9,78 | 1E+00 | -4,29 | 19,56 |
| RR16h0.1R1 | 1 | 3 | 0.1 µM 20E | 2 | 16 | 35,28403 | 0,857025 | 35,00 | 23,20 | 11,80 | 9,78 | 1E+00 | 2,01 | 0,25 |
| RR16h0.1R2 | 2 | 3 | 0.1 µM 20E | 2 | 16 | 33,93746 | 0,78569 | 33,94 | 22,12 | 11,81 | 9,78 | 1E+00 | 2,03 | 0,24 |
| RR1h0.1R1 | 1 | 3 | 0.1 µM 20E | 2 | 1 | 29,65804 | 0,447243 | 29,66 | 22,63 | 7,02 | 9,78 | 1E+00 | -2,76 | 6,78 |
| RR1h0.1R2 | 2 | 3 | 0.1 µM 20E | 2 | 1 | 29,36862 | 0,249339 | 29,37 | 23,47 | 5,90 | 9,78 | 1E+00 | -3,89 | 14,78 |
| RR2h0.1R1 | 1 | 3 | 0.1 µM 20E | 2 | 2 | 30,90377 | 0,212546 | 30,90 | 23,17 | 7,74 | 9,78 | 1E+00 | -2,05 | 4,14 |
| RR2h0.1R2 | 2 | 3 | 0.1 µM 20E | 2 | 2 | 30,87161 | 0,19248 | 30,87 | 23,35 | 7,53 | 9,78 | 1E+00 | -2,26 | 4,78 |
| RR4h0.1R1 | 1 | 3 | 0.1 µM 20E | 2 | 4 | 34,2086 | 1,202596 | 34,21 | 23,54 | 10,66 | 9,78 | 1E+00 | 0,88 | 0,54 |
| RR4h0.1R2 | 2 | 3 | 0.1 µM 20E | 2 | 4 | 32,71961 | 0,286276 | 32,72 | 23,57 | 9,15 | 9,78 | 1E+00 | -0,63 | 1,55 |
| RR8h0.1R1 | 1 | 3 | 0.1 µM 20E | 2 | 8 | 33,47275 | 0,404405 | 33,47 | 23,14 | 10,34 | 9,78 | 1E+00 | 0,55 | 0,68 |
| RR8h0.1R2 | 2 | 3 | 0.1 µM 20E | 2 | 8 | 33,82555 | 0,690663 | 33,83 | 22,81 | 11,01 | 9,78 | 1E+00 | 1,23 | 0,43 |

### S4: Calibration curve performance per PCR run

| Experiment number | PCR run id | Target | Efficiency % | Slope | Y-Intercept | R^2 |
| --- | --- | --- | --- | --- | --- | --- |
| 1 | A | DHR3 | 110,79 | -3,088 | 46,834 | 0,991 |
| 1 | A | GAPDH | 107,33 | -3,158 | 37,681 | 0,982 |
| 1 | A | E75A | 99,79 | -3,327 | 44,607 | 0,992 |
| 1 | A | EcRB1 | 113,49 | -3,036 | 43,868 | 0,974 |
| 1 | B | Ftz-f1 | 102,96 | -3,253 | 45,154 | 0,990 |
| 1 | B | GAPDH | 111,93 | -3,066 | 38,790 | 0,992 |
| 1 | C | a-tub | 115,57 | -2,998 | 35,148 | 0,999 |
| 1 | C | GAPDH | 112,14 | -3,062 | 37,110 | 0,995 |
| 1 | C | E75b | 106,42 | -3,177 | 44,273 | 0,992 |
| 1 | C | BrC | 115,57 | -2,998 | 43,552 | 0,979 |
| 1 | C | GAPDH | 109,67 | -3,110 | 36,370 | 0,994 |
| 2 | D | DHR3 | 98,60 | -3,356 | 47,697 | 0,987 |
| 2 | D | E75b | 97,60 | -3,381 | 46,987 | 0,992 |
| 2 | D | GAPDH | 101,50 | -3,287 | 38,537 | 0,994 |
| 2 | D | EcRA | 116,01 | -2,990 | 46,097 | 0,937 |
| 2 | D | EcRB1 | 108,38 | -3,136 | 43,781 | 0,984 |
| 2 | D | Ftz-f1 | 95,42 | -3,437 | 44,864 | 0,999 |
| 2 | E | BrC | 108,88 | -3,126 | 44,188 | 0,941 |
| 2 | E | E75A | 106,44 | -3,177 | 43,163 | 0,999 |
| 2 | E | GAPDH | 106,05 | -3,185 | 37,023 | 0,998 |
| 3 | E | BrC | 99,56 | -3,333 | 45,317 | 0,999 |
| 3 | E | E75A | 96,84 | -3,400 | 44,864 | 0,991 |
| 3 | E | GAPDH | 105,05 | -3,206 | 37,140 | 0,997 |
| 3 | F | DHR3 | 115,68 | -2,996 | 46,660 | 0,980 |
| 3 | F | E75b | 94,77 | -3,454 | 47,938 | 0,954 |
| 3 | F | GAPDH | 108,50 | -3,134 | 37,757 | 0,986 |
| 3 | F | EcRA | 101,51 | -3,286 | 47,679 | 0,982 |
| 3 | F | EcRB1 | 99,12 | -3,343 | 44,951 | 0,974 |
| 3 | F | Ftz-f1 | 95,45 | -3,436 | 44,079 | 0,993 |
| 4 | G | Ftz-f1 | 106,19 | -3,182 | 46,170 | 0,883 |
| 4 | G | DHR3 | 100,52 | -3,310 | 43,905 | 0,991 |
| 4 | G | E75b | 105,53 | -3,196 | 43,068 | 0,980 |
| 4 | G | GAPDH | 99,80 | -3,327 | 38,155 | 0,985 |
| 5 | H | DHR3 | 107,26 | -3,159 | 44,549 | 0,928 |
| 5 | H | E75b | 102,01 | -3,275 | 43,772 | 0,998 |
| 5 | H | GAPDH | 103,41 | -3,243 | 38,527 | 0,994 |
| 5 | H | EcRB1 | 98,18 | -3,366 | 44,827 | 0,999 |
| 5 | H | Ftz-f1 | 94,40 | -3,464 | 45,364 | 0,981 |
| 5 | H | rp49 | 105,81 | -3,190 | 33,681 | 0,998 |
| 6 | I | DHR3 | 100,06 | -3,321 | 47,834 | 0,953 |
| 6 | I | E75b | 91,08 | -3,556 | 48,723 | 0,976 |
| 6 | I | GAPDH | 105,37 | -3,200 | 38,826 | 0,977 |
| 6 | I | EcRB1 | 94,69 | -3,456 | 47,626 | 0,986 |
| 6 | I | Ftz-f1 | 93,99 | -3,475 | 45,510 | 0,992 |
| 6 | I | rp49 | 102,93 | -3,254 | 35,052 | 0,998 |
